# Metagenomic-Metabolomic Mining of *Kinema*, a Naturally Fermented Soybean Food of the Eastern Himalayas

**DOI:** 10.3389/fmicb.2022.868383

**Published:** 2022-04-29

**Authors:** Pynhunlang Kharnaior, Jyoti Prakash Tamang

**Affiliations:** Department of Microbiology, School of Life Sciences, Sikkim University, Gangtok, India

**Keywords:** fermented soybean, metagenomics, metabolomics, immunodomulators, metataxonomic

## Abstract

*Kinema* is a popular sticky fermented soybean food of the Eastern Himalayan regions of North East India, east Nepal, and south Bhutan. We hypothesized that some dominant bacteria in *kinema* may contribute to the formation of targeted and non-targeted metabolites for health benefits; hence, we studied the microbiome–metabolite mining of *kinema*. A total of 1,394,094,912 bp with an average of 464,698,304 ± 120,720,392 bp was generated from *kinema* metagenome, which resulted in the identification of 47 phyla, 331 families, 709 genera, and 1,560 species. Bacteria (97.78%) were the most abundant domain with the remaining domains of viruses, eukaryote, and archaea. *Firmicutes* (93.36%) was the most abundant phylum with 280 species of *Bacillus*, among which *Bacillus subtilis* was the most dominant species in *kinema* followed by *B. glycinifermentans*, *B. cereus*, *B. licheniformis*, *B. thermoamylovorans*, *B. coagulans*, *B. circulans*, *B. paralicheniformis*, and *Brevibacillus borstelensis*. Predictive metabolic pathways revealed the abundance of genes associated with metabolism (60.66%), resulting in 216 sub-pathways. A total of 361 metabolites were identified by metabolomic analysis (liquid chromatography-mass spectrophotometry, LC-MS). The presence of metabolites, such as chrysin, swainsonine, and 3-hydroxy-L-kynurenine (anticancer activity) and benzimidazole (antimicrobial, anticancer, and anti-HIV activities), and compounds with immunomodulatory effects in *kinema* supports its therapeutic potential. The correlation between the abundant species of *Bacillus* and primary and secondary metabolites was constructed with a bivariate result. This study proves that *Bacillus* spp. contribute to the formation of many targeted and untargeted metabolites in *kinema* for health-promoting benefits.

## Introduction

Fermented soybean products are one of the oldest food items of the Oriental Asians (Shurtleff and Aoyagi, 2017), which are either fungal-fermented products used as condiments and taste makers, such as *shoyu* of Japan, *doenjang* of Korea, and *douchi* of China, and also eaten as foods such as *miso* of Japan, *tempe* of Indonesia, and *sufu/furu* of China (Kadar et al., 2020; Takeshita, 2020), or bacteria-fermented foods such as *natto*, *kinema*, *cheonggukjang*, *thua-nao*, etc. (Tamang et al., 2016, 2020; Kwon et al., 2019). Historically and anthropologically, spontaneously fermented sticky soybean foods with umami flavor (Hartley et al., 2019) are confined to few Asian countries, namely, Japan (*natto*), Korea (*cheonggukjang*), Laos (*sieng*), northern Thailand (*thua nao*), Myanmar (*pe poke*), eastern Nepal and southern Bhutan (*kinema*), and North East India (*kinema*, *hawaijar*, *bekang*, *tungrymbai*, *peha*, *peron-namsing*, *peruyaan*, and *aakhonii*) (Kwon et al., 2019; Tamang et al., 2021a). Sticky fermented soybean foods are still produced traditionally by natural fermentation (Tamang, 2015), except Japanese *natto*, which is now produced by the commercial starter of *Bacillus subtilis* variety *natto* strain (Ju et al., 2019). Colossal microbial community structures with inter–intra species diversity have been observed in some fermented soybean foods of Asia (Kharnaior and Tamang, 2021; Ren et al., 2021), which are also the major sources of biological active compounds (Cao et al., 2019).

Shotgun metagenome sequence analysis is one of the most advance sequence-based taxonomic tools, which profiles the microbial domains up to species level (Durazzi et al., 2021), including culturable and unculturable bacteria, yeasts, fungi, viruses, and archaea in food samples (Leech et al., 2020). Metabolomics approach, based on liquid chromatography-mass spectrophotometry (LC-MS)-based chromatographic analysis, has been extensively applied in fermented soybean foods (Liu et al., 2021; Song et al., 2021) to profile targeted and untargeted metabolites (Eudy et al., 2020) with multiple applications in nutrition (González-Peña and Brennan, 2019). Among the untargeted metabolites, immunomodulators (Cambeiro-Pérez et al., 2018) are considered as the vital biomolecules for health-promoting benefits (Shahbazi et al., 2021). Integrated metagenomics–metabolomics approaches have been reported in few fermented foods such as Chinese pu-erh tea (Zhao et al., 2019), kombucha (Villarreal-Soto et al., 2020), kefir (Blasche et al., 2021), and doenjang and ganjang of Korea (Song et al., 2021).

**FIGURE 1:**
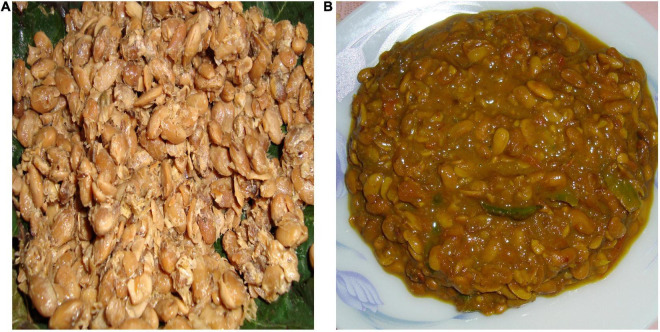
**(A)** Freshly prepared *kinema*; **(B)** *Kinema* curry.

*Kinema* (Figure 1A) is a sticky and umami-flavored solid fermented soybean food, which is conventionally prepared by natural fermentation, and is consumed as curry (Figure 1B) by the Nepali/Gorkha community of the Eastern Himalayan regions of North East India, east Nepal, and south Bhutan (Nikkuni et al., 1995; Tamang, 2021) for more than 2,000 years (Tamang and Samuel, 2010). During *kinema* preparation, local varieties of soybean seeds are soaked overnight, boiled, and the excess water is discarded; cooked beans are wrapped in fern fronds or paddy straw inside bamboo-made baskets and placed above earthen kitchen for natural fermentation for 1–3 days (Tamang, 2015). During the natural fermentation, soybean grits are covered with whitish viscous sticky materials, presumably poly-glutamic acid (Chettri et al., 2016), with umami taste and is commonly eaten as a side dish with steamed rice (Tamang, 2015). Microbiological investigation of *kinema* samples by culture method showed *Bacillus subtilis* as the dominant bacterium during natural fermentation (Sarkar et al., 1994; Tamang and Nikkuni, 1996; Tamang, 2003). Targeted high-throughput sequencing was applied to study the metataxonomic of *kinema* samples collected from India, Nepal, and Bhutan, which revealed the abundance of *B. subtilis*, few other non-*Bacillus* bacteria, yeasts, and molds detected in low abundances (Kharnaior and Tamang, 2021). However, metagenome–metabolite mining of fermented foods may help to understand the roles of beneficial microorganisms for production of primary and secondary metabolites. We hypothesized that some dominant bacteria present in *kinema* may contribute to the formation of targeted and non-targeted metabolites. Hence, this paper is aimed to decipher the integrative metagenomic and metabolomic approach to profile the abundant domains in *kinema* samples from three different countries, namely, India, Nepal, and Bhutan, and to determine both targeted and non-targeted metabolites by LC-MS. Functional profiles of metagenomes were also predicted using the SqueezeMeta pipeline and Kyoto Encyclopedia of Genes and Genomes (KEGG) database and were validated with the metabolomics profiles of the samples to find the relationship between predominant species and metabolite formation in *kinema*.

## Materials and Methods

### Sample Collection

Eighteen sun-dried samples of *kinema* were aseptically collected from different places of India, Nepal, and Bhutan (Supplementary Table 1) in a pre-sterile container, sealed, labeled, and brought to laboratory for further analysis.

### Genomic DNA Extraction

Ten grams of each sample were homogenized with 90 ml sterile 0.1 M phosphate buffer (pH 6.4) and kept undisturbed for 5 min to settle down the sample debris. Homogenate was collected, and genomic DNA was extracted using Nucleospin Food kit (Macherey-Nagel GmbH & Co., KG, Düren, Germany) following the manufacturer’s instructions. Quantification of DNA concentration was checked using a spectrophotometer (Eppendorf, United States), and quality of DNA was observed in 0.8% agarose gel electrophoresis followed by visualization under Gel Doc EZ imager (Bio-Rad, United States). Following the method described by Ray et al. (2019), the DNA from 18 samples of three countries were pooled in equal quantity (pooled from six initial samples of each country i.e., India, Nepal, and Bhutan), and the mixture was considered as one sample from each country and were used for metagenomic analysis.

### MinION Library Preparation and Metagenomics Sequencing

The MinION gDNA sequencing Ligation Kit SQK-LSK109 (Oxford Nanopore Technologies, Oxford, United Kingdom) was used to prepare the metagenome libraries (Sevim et al., 2019) according to the manufacturer’s instruction. Genomic DNA (10 μg) was sheared using the Covaris g-tubes to produce the fragments of the size > 10 kb (Covaris Inc., MA, United States). The sheared genomic DNA with appropriate size was then selected using the BluePippin instrument (Sage Science, Beverly, MA, United States). End-repairing was performed on the sheared DNA using the NEBNext FFPE DNA Repair kit following the manufacturer’s protocol (New England BioLabs, Ipswich, MA, United States). Qubit HS DNA kit was used to quantify the gDNA. Ligation Sequencing Kit SQK-LSK109 was used for the clean-up and adapter ligation. The ligated sample was purified and eluted using the AMPure XP beads (Beckman Coulter Inc., United States) along with wash buffer and kit. Libraries were then sequenced (1D sequencing) on MinION MK1 device using R9 flow cell chemistry. The device was controlled through the MinKNOW software version 1.0.5 (Oxford Nanopore Technologies, Oxford, United Kingdom); Metrichor platform of ONT (Oxford Nanopore Technologies) was used to perform the 1D base calling.

### Bioinformatics Analysis

#### Metataxonomic

The generated fast5 data from MinION ONT was processed by converting into fastq using poretools software version 0.6.0 for *kinema* metagenome (Loman and Quinlan, 2014). Furthermore, the sequence quality was subjected and examined using NanoPlot version 1.30.1 (De Coster et al., 2018) and was assembled using canu-assembler (Koren et al., 2017). Taxonomy classification of the assembled quality sequences were assigned using the Kaiju taxonomic pipeline (Menzel et al., 2016), mapped against the GenBank-derived database (NCBI nr + euk) containing millions of protein sequences from bacteria, viruses, eukaryotes, and archaea (Chen et al., 2017). Default “greedy” algorithm was preferred to map the sequences against the database (Zhang et al., 2000). A minimum required match length, minimum match score, and *E*-value of *m* = 11, *s* = 80, and *E* = 0.05, respectively, was filtered to avoid mismatches, chimera, low-complexity regions, and false positive taxon assigned (Menzel et al., 2016). Additionally, amino acid substitution was performed in amino acid sequence, and multiple matches were ranked, and taxon was classified from the database. After translation of open reading frames (ORFs) into a set of amino acid fragments, the same fragments were ranked by BLOSUM62 (BLOcks SUbstitution Matrix) score and the database search with the highest scoring was started (Lazar et al., 2019). Burrows–Wheeler transform (BWT) algorithm was used to search the fragments backward against the database (Pokrzywa and Polanski, 2010). The fragments with higher score were used for classifying and outputting the taxon identifier (Menzel et al., 2016).

#### Predictive Functional Features

Predictive metabolic pathways of *kinema* metagenome were derived using SqueezeMeta v1.3.0 (Tamames and Puente-Sánchez, 2019). Imported metagenomic data with contigs of < 500 bp were removed using prinseq (Schmieder and Edwards, 2011). Prediction of gene was performed using Prodigal v2.6.2 (Hyatt et al., 2012), and the homologies of predicted genes were searched against the functional database using DIAMOND, a computational tool for the alignment of sequencing reads against a protein reference database, which aligned the sequence against a protein database (Buchfink et al., 2015). After running the DIAMOND, the method assigned as functions to each ORF was carried out using the fun3 method (fun3 method produced functional assignments to compare gene sequences against the functional database) for clusters of orthologous groups/non-supervised orthologous groups (COGs/NOGs) using evolutionary genealogy of genes: non-supervised orthologous groups (eggNOG) database (Huerta-Cepas et al., 2016) and KEGG database (Kanehisa and Goto, 2000). The highest-scoring ORFs exceeding 30% (default) were considered for annotation (Tamames and Puente-Sánchez, 2019). Annotations were processed for predictive pathways and enzyme classification (Ye and Doak, 2009). Additionally, high-level function (level 1), lower-level function (level 2), and the sub-pathways (level 3) were categorized (Scala et al., 2019), and relative abundance of > 1% was visualized by bar plot using STAMP v2.1.3 (Parks et al., 2014). The inter distribution of predictive functional profiles among samples was performed using Mann–Whitney test (Fong and Huang, 2019) considering the sub-pathways (level 3).

### Metabolite Profiling by Liquid Chromatography-Mass Spectrophotometry Analysis

LC-MS coupled to a Q-Exactive Orbitrap (Dionex Ultimate 3000, Thermo Fisher Scientific, United States) was used for the metabolite profiling of *kinema* samples (three samples each from India, Nepal, and Bhutan) following the method of Ramakrishnan et al. (2016). A 200-mg amount of each powdered sample was mixed with 1 ml of 80% methanol and sonicated in ice, the mixture was centrifuged at 14,000 rpm for 5 min at 4°C, and the supernatant was filtered using a 0.2-μm PTFE filter. Then, 10 μl of each sample was injected into the LC-MS system coupled to a Q-Exactive Orbitrap, and chromatography was performed on a hydrophilic interaction liquid chromatography (HILIC) (5 μ, 150 mm × 4.6 mm, Phenomenex Luna) with a flow rate of 0.4 ml/min at 40°C. The mobile phase A contained 10 mM ammonium acetate with acetonitrile in the ratio of 1:1 (0.1% FA), and phase B contained acetonitrile (0.1% FA) for positive mode. Whereas, for the negative mode, the mobile phase A contained 5 mM ammonium acetate in water, and the phase B contained 5 mM ammonium acetate in water with acetonitrile in the ratio of 1:9. The following gradient gave optimal resolution and was used in both ionization modes (0–2 min: 100% B, 2–15 min: 100–90% B, 15–25 min: 90–80% B, 25–30 min: 80–75% B, 30–35 min: 75–20% B, 35–40 min: 20–0% B, 40–45 min: 0% B, 45–45.1: 0–100% B, and 45.1–55 min: 100% B) at a 400-μl/min flow rate. The Q-Exactive Orbitrap (Thermo Fisher Scientific, United States) was set up for data acquisition in the full scan/data-dependent scan (FS/DDS) mode in a mass range of 70–1,050 *m*/*z*, alternating between MS and MS/MS scans. The MS operating conditions for all samples were as follows: spray voltage, 4,000 V (2,500 V for negative); vaporizer temperature, 320 °C; sheath gas flow rate of 30 arbitrary units (40 for negative); and auxiliary gas flow rate of 10 arbitrary units. Injector settings were as follows: 0–2 min: waste, 2–45 min: load, and 45–55 min: waste. The spiked reserpine and taurocholate were used as internal standards and later used for intensity normalization of data. The raw data files were imported into SIEVE 2.2 for the generation of peak list and component extraction (Hao et al., 2018), and the Human Metabolome DataBase (HMDB), KEGG (Zhang B. et al., 2018), and PlantCyc (Zhang et al., 2010) were used for possible identification of the compounds. The coefficient of variation (CV) was calculated from the pooled quality control sample data, and the data with CV_QC > 20% were removed.

### Statistical Analysis

#### Pooled Sequences

Nucleotide diversity (pi) analysis and different indices of neutrality test, based on sequence polymorphism in DnaSP software version 6 (Rozas et al., 2017), were performed to justify the pooling of DNA from six samples from each country (India, Nepal, and Bhutan) for pooled sequence in terms of intra-sample (within the sample) diversity. For each sample, the intra-species diversity was calculated by one-sample *t*-test using IBM SPSS v20.0 (Statistical Package for the Social Sciences) to check the significant differences within the sample. Statistical relations among the samples were performed using Mann–Whitney test (Fong and Huang, 2019).

#### Diversity Indices

Non-parametric Shannon index and Simpson’s index of diversity (1-D) were calculated for beta diversity indices using PAST software version 4.0 (Martino et al., 2019). Bray–Curtis index of beta diversity was also calculated using PAST version 4.0 and visualized *via* principal coordinates analysis (PCoA) plot (Martino et al., 2019). The clustering pattern of microbial population was calculated using the unweighted pair group method with arithmetic mean (UPGMA) (Parks and Beiko, 2012). Fisher’s exact test (non-parametric) was performed through the Statistical Analysis of Metagenomic Profiles (STAMP) software version 2.1.3 (Parks et al., 2014) to check the significance in the distribution of different microbial taxa among the samples.

#### Metabolite Profiles

The significant differences of metabolites were calculated by Student *t*-test (two-tailed) (Kwon et al., 2019). Metabolites were log transformed (log *x*_*i*_ + 1) and analyzed by PCoA using Bray–Curtis dissimilarity (Djeni et al., 2020). The hierarchical clustering of metabolites was calculated using UPGMA (Parks and Beiko, 2012). The correlation between the significant differentiated metabolites of more than fourfold and predominant species was measured by a non-parametric Spearman’s rank correlation using IBM SPSS v20.0. The network-based correlation was visualized using MetScape v3.1.3 in Cytoscape v3.8.2. Metabolites identified by LC-MS analysis were imported in MetaboAnalyst (v5.0) and further carried out to describe the association of metabolites with KEGG pathways referring to HMDB (Pang et al., 2021). Additionally, metabolites associated with predictive pathways were analyzed by network-functional analysis modules and visualized using KEGG global metabolic network (Pang et al., 2021). The variation of metabolites among the samples was analyzed based on the fold change (FC) ratio. The outcome was transformed into log value (log_2_ FC) (Hyeon et al., 2020), and significant differences were tested using analysis of variance (ANOVA) (Song et al., 2021).

## Results

### Pooled Sequence Statistics

Nucleotide diversity (Pi) per site of pooled samples was 0.72668 (India), 0.7243 (Nepal), and 0.72687 (Bhutan), respectively. The neutrality indices were performed using two tests: Tajima’ *D* with values of 1.363 (India), 1.347 (Nepal), and 1.364 (Bhutan), respectively, and Fu’s Fs statistic representing the values of -46.669, -31.869, and -43.373 of *kinema* from India, Nepal, and Bhutan, respectively. Pooled DNA samples were not significantly (*p* > 0.1) different in terms of sequence variation within the sample.

### Metagenomic Sequencing and Microbial Community

**FIGURE 2:**
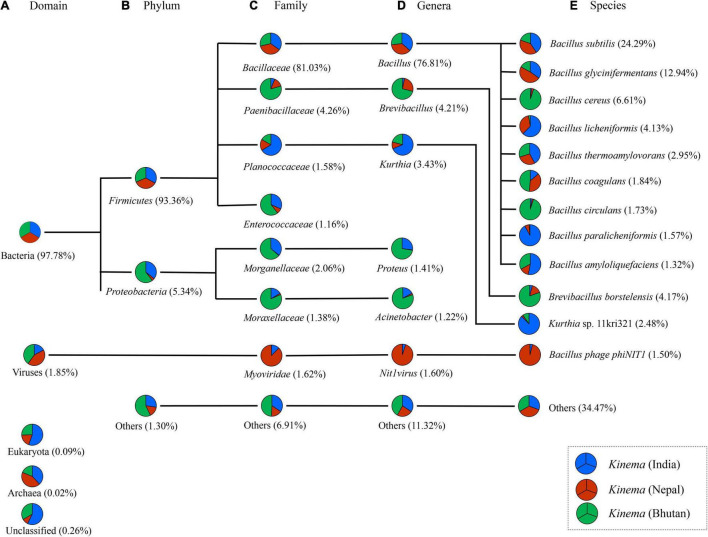
Differences in relative abundance (%) of predominant species in *kinema* metagenome at different taxonomic levels **(A)** domain, **(B)** phylum, **(C)** family, **(D)** genus, and **(E)** species levels. The minor species with a relative percentage of less than 1% were grouped and represented as others.

A total of 1,394,094,912 bp with an average of 464,698,304 ± 120,720,392 bp was generated from *kinema* metagenome. In comparison, among the samples, *kinema* from Nepal has the longest contig of 533,203 bp, whereas *kinema* from Bhutan has the shortest contig of 1,001 bp (Table 1). Raw reads from the taxonomic classification analysis were then normalized as relative percentages for visualization, and taxonomic abundances with > 1% and those with < 1% abundances were grouped as “others.” The overall taxonomy classification resulted in the identification of 47 phyla, 331 families, 709 genera, and 1,560 species. In the organismal diversity, the most abundant domain was bacteria (97.78%) in *kinema* metagenome, and the remaining domains were viruses, eukaryote, and archaea (Figure 2A). *Firmicutes* (93.36%) was the most abundant bacterial phylum (Figure 2B and Supplementary Table 2). Further classification led to the identification of abundant families represented by *Bacillaceae* (81.03%), followed by *Paenibacillaceae*, *Planococcaceae*, and *Enterococcaceae* (Figure 2C and Supplementary Table 3). *Bacillus* was the most abundant genus followed by *Brevibacillus*, *Kurthia*, *Nit1virus* (virus), *Proteus*, *Acinetobacter*, and other genera (Figure 2D and Supplementary Table 4). At the species level, *B. subtilis* was detected as the most abundant species in *kinema*, followed by *B. glycinifermentans*, *B. cereus*, *Brevibacillus borstelensis*, *B. licheniformis*, *B. thermoamylovorans*, *Kurthia* sp. 11kri321, *B. coagulans*, *B. circulans*, *B. paralicheniformis*, and others (Figure 2E and Supplementary Table 5). No eukaryote and archaea species were found with > 1% relative abundance in *kinema* metagenomes.

**TABLE 1:** General summary and details of metagenomic sequences and assembly statistics.

Sequence summary	*Kinema*		
	
	India	Nepal	Bhutan
Mean read length	682.6	721.2	543.4
Number of reads	480,129	708,244	1,022,385
Total bases	327,718,808	510,807,788	555,568,316
Longest contig	317,256	533,203	120,895
Shortest contig	1,014	1,011	1,001
COG ORFs	12,490	16,022	14,988
KEGG ORFs	9,600	12,383	11,379

**FIGURE 3:**
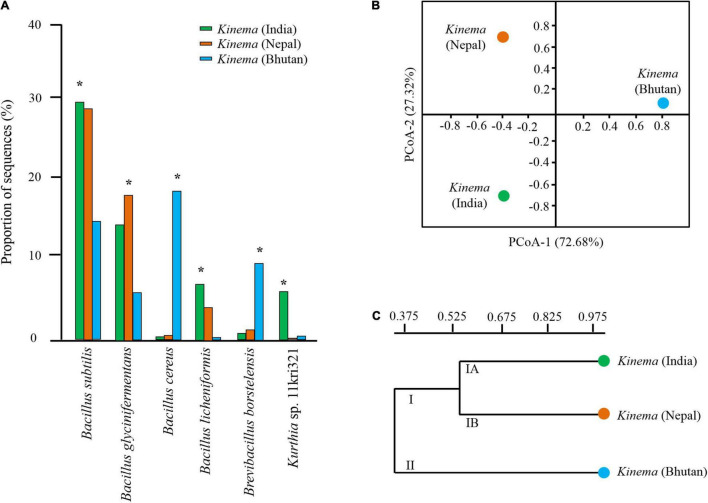
**(A)** Significant differences* of species abundance with a relative abundance of more than 1% were calculated among samples by a non-parametric Fisher’s exact statistical test. **(B)** Principal coordinates analysis (PCoA) plot using Bray–Curtis dissimilarity. **(C)** Unweighted pair group method with arithmetic mean (UPGMA) hierarchical cluster constructed based on species level to observe the differences in the overall microbial structure and diversity in *kinema* samples collected from different geographical locations.

The result showed that *B. subtilis* was predominant in *kinema* samples of India and Nepal (Figure 3A). Contrastingly, *B. cereus* and *B. borstelensis* were significantly (*p* < 0.05) abundant in *kinema* samples of Bhutan (Figure 3A). In the overall distribution of species of all domains, bacterial species were the dominant domain in which 280 species of *Bacillus* (Supplementary Table 6) and 121 species of lactic acid bacteria (LAB) (Supplementary Table 7) were detected with < 1% abundances in *kinema* metagenomes. Apart from bacteria, the second abundant domain was viruses with a total of 42 phage(s), out of which 26 were *Bacillus* phage and 16 were others (Supplementary Table 8). *Myoviridae* was the predominant viral family, among which *Bacillus* phage phiNIT1 was the abundant phage observed in *kinema*. Among eukaryotes, a total number of 56 species were identified; 17 species each were classified as molds and yeasts, respectively, and 22 species were grouped as other microbial eukaryotes (Supplementary Table 9). Additionally, 11 species were identified under the domain archaea (Supplementary Table 10).

### Diversity Indices and Shared and Unique Species

Good’s coverage ranging from 0.98 to 0.99 was observed in all samples. The alpha diversity indices revealed the diversity in the following order: Bhutan (0.92) > India (0.71) > Nepal (0.57) (data not shown). Similarly, a non-parametric Shannon index also followed the same pattern, Bhutan (5.45) > India (3.92) > Nepal (2.89). Intra-diversity showed no significant differences in pooled samples of *kinema* from India (*p* = 0.54), Nepal (*p* = 0.55), and Bhutan (*p* = 0.48). Based on the species distribution and its relative abundance of all domains, the inter-diversity, calculated by PCoA, was distinctly different among samples (Figure 3B). The hierarchical clustering UPGMA revealed two clusters: cluster I (*kinema* from India and Nepal) and cluster II (*kinema* from Bhutan). Cluster I was observed with two subclusters, cluster IA and cluster IB of *kinema* from India and Nepal, respectively (Figure 3C). Statistical variation in the distribution of species among samples showed that *kinema* from India was significantly different from Nepal (*p* = 0.01945) and Bhutan (*p* = 0.000127), respectively. Similarly, *kinema* from Nepal was also significantly (*p* = 0.00007398) different from Bhutan.

**FIGURE 4:**
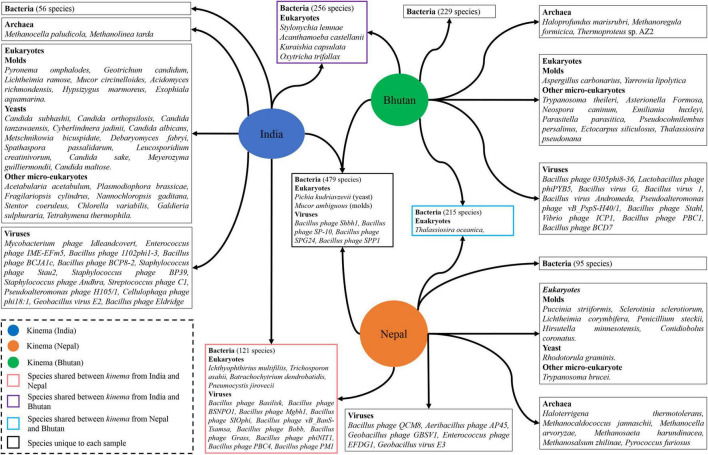
Representation of shared and unique species identified from different domains in *kinema* samples.

It was observed that 485 overall species including bacteria, viruses, eukaryotes, and archaea were common in all samples. We observed that 479 bacterial species were shared in all samples, whereas 56 species were unique to India, 95 species to Nepal, and 229 species to Bhutan, respectively (Figure 4 and Supplementary Table 11). Among viruses, four species were found common such as *Bacillus* phage Shbh1, *Bacillus* phage SP-10, *Bacillus* phage SPG24, and *Bacillus* phage SPP1, whereas 13, 5, and 10 viral species were unique to *kinema* from India, Nepal, and Bhutan, respectively (Figure 4 and Supplementary Table 12). Among eukaryotes, two species (*Pichia kudriavzevii* and *Mucor ambiguousi*) were the common species, whereas 27, 8, and 10 species were unique species to India, Nepal, and Bhutan, respectively (Figure 4 and Supplementary Table 13). No common and shared archaea species were detected in *kinema* samples, whereas two, six, and three species of archaea were found unique to India, Nepal, and Bhutan, respectively (Figure 4 and Supplementary Table 14).

### Predictive Functional Profiles

**FIGURE 5:**
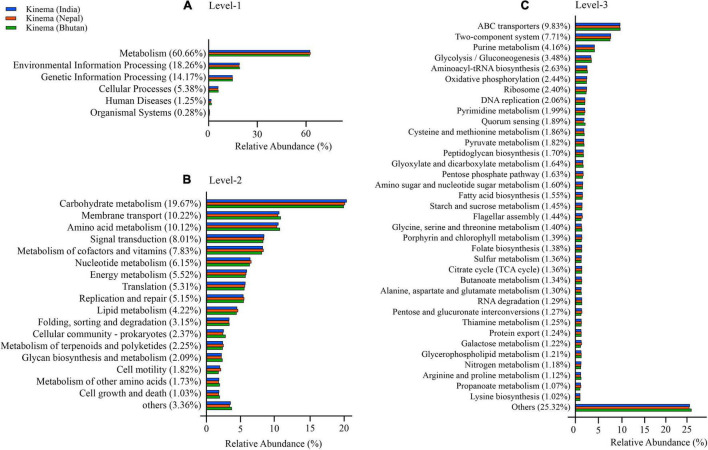
Predictive functional features mapped against Kyoto Encyclopedia of Genes and Genomes (KEGG) database were represented by relative abundance (%) at three different categories: **(A)** Level 1, **(B)** level 2, and **(C)** level 3. The functional features with less than 1% were grouped as others.

**FIGURE 6:**
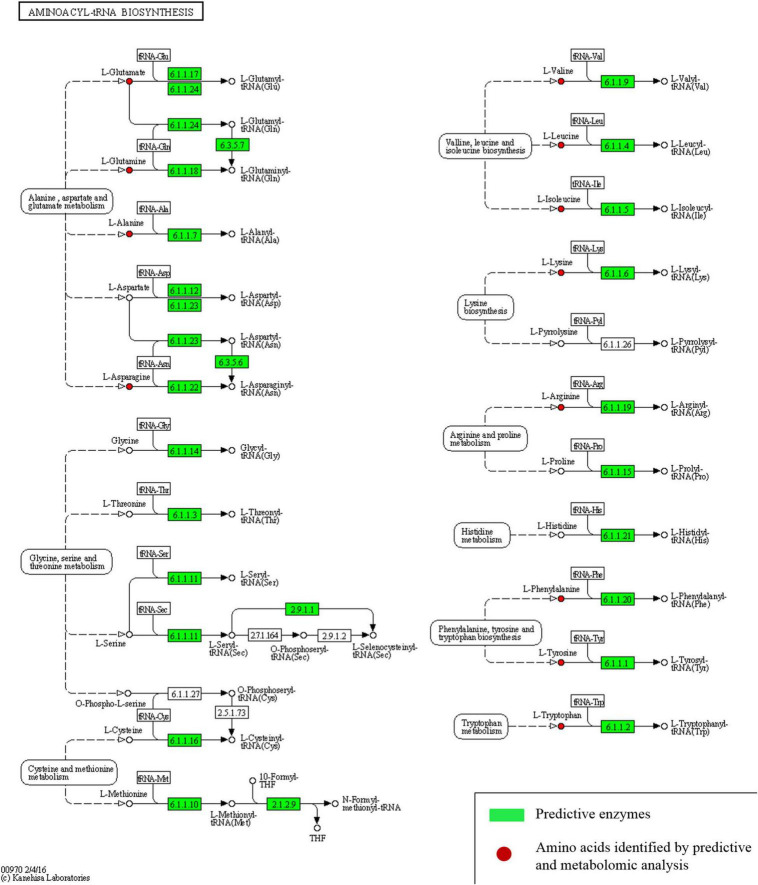
Predictive enzymes involved in aminoacyl-tRNA biosynthesis of different amino acids.

**FIGURE 7:**
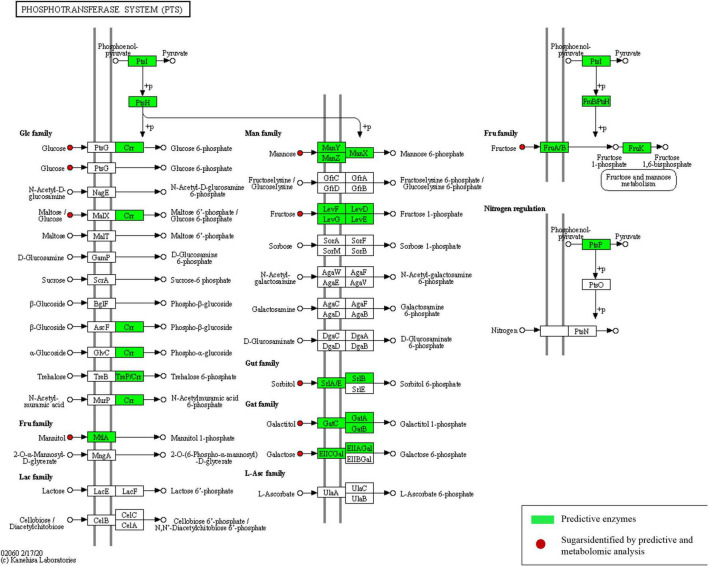
Predictive enzymes involved in phosphotransferase system (PTS).

Different enhanced functional pathways were observed after mapping metagenomic ORFs against eggNOG and KEGG databases. Around 56.41–56.84% of the total mapped ORFs were assigned to COG genes, and 43.16–43.59% were assigned as KEGG pathway genes. Metagenome ORFs were aligned with genes associated to 216 KEGG metabolic pathways and categorized into three different levels. In level 1, metabolism was the most abundant category followed by environmental information processing, genetic information processing, cellular processes, human diseases, and organismal systems (Figure 5A). At level 2, there were 17 super-pathways with > 1% abundance and minor super-pathways with a relative abundance < 1% (Figure 5B), and at level 3, there were 36 super-pathways with > 1% abundance (Figure 5C) with 180 minor super-pathways with a relative abundance of < 1% (Supplementary Table 15). The distribution of the functional features in *kinema* metagenome was compared, and significant differences among the functional features of *kinema* of India, Nepal (*p* = 0.04182), and Bhutan (*p* = 0.02698) were found. Similarly, significant differences between *kinema* from Nepal and Bhutan (0.00007833) were observed. Exploring the enzyme classification by predictive analysis revealed the presence of protease, glucosidase, galactosidase, amylase, lipase, and γ-PGA proteins and enzymes involved in its biosynthesis (Supplementary Table 16). Additionally, further analysis was carried out on enzymes involved in aminoacyl-tRNA biosynthesis (Figure 6 and Supplementary Table 17) and phosphotransferase system (PTS) (Figure 7 and Supplementary Table 18), which revealed the enzymes responsible in the biosynthesis of amino acids and sugar components present in *kinema*.

### Metabolites Associated With Predictive Kyoto Encyclopedia of Genes and Genomes Pathways

**FIGURE 8:**
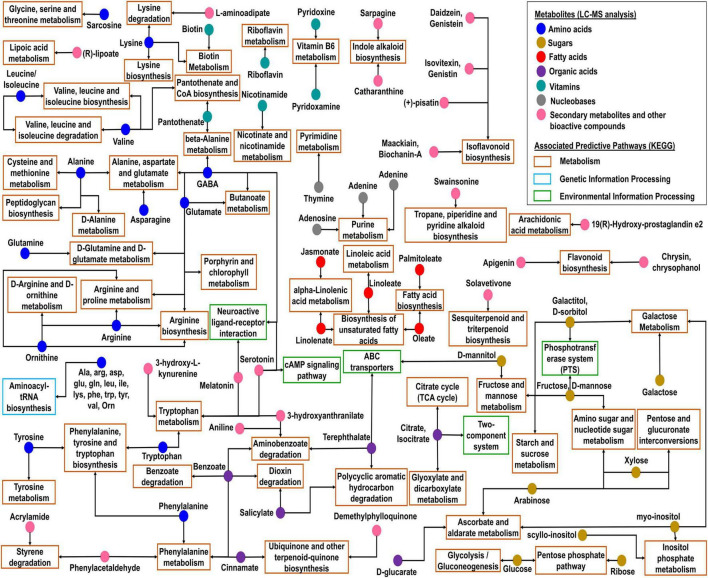
Network and functional analysis modules of metabolites associated with predictive KEGG pathways represented *via* KEGG global metabolic network using MetaboAnalyst.

The analysis of metabolites associated with predictive functional pathways using MetaboAnalyst v5.0 network revealed that many compounds were found associated with different metabolic pathways (Figure 8 and Supplementary Table 19). Amino acids such as glutamine, asparagine, valine, alanine/sarcosine, arginine, lysine, phenylalanine, tyrosine, glutamate, leucine/isoleucine, tryptophan, ornithine, and 4-aminobutanoate (γ-aminobutyric acid, GABA) were associated with predictive pathways related to amino acid metabolism. Fatty acids were associated with fatty acid biosynthesis, biosynthesis of unsaturated fatty acids, and alpha-linolenic acid metabolism (Figure 8). Sugars were mostly found to be associated with carbohydrate metabolism. Whereas, in citrate cycle (TCA cycle) pathways of the carbohydrate metabolism, organic acids such as citrate and isocitrate were involved, which were also found to be associated with glyoxylate and dicarboxylate metabolism. Among metabolites, daidzein, genistein, isovitexin, genistin, maackiain, biochanin-A, (+)-pisatin, apigenin, chrysin, and chrysophanol were found to be associated with flavonoid and isoflavonoid biosynthesis. Biotin, nicotinamide, pyridoxamine, pyridoxine, pantothenate, and riboflavin were found to be associated with metabolism of vitamins and cofactors. (R)-lipoate was associated with lipoic acid metabolism. Additionally, sarpagine and catharanthine were associated with indole alkaloid biosynthesis; swainsonine with tropane, piperidine, and pyridine alkaloid biosynthesis; and solavetivone with sesquiterpenoid and triterpenoid biosynthesis (Supplementary Table 19).

### Targeted and Untargeted Metabolites

**FIGURE 9:**
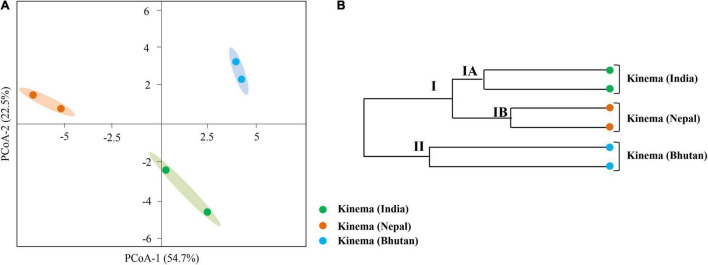
Comparison of metabolite formation in *kinema* samples from India, Nepal, and Bhutan. **(A)** PCoA using Bray–Curtis dissimilarity. **(B)** UPGMA hierarchical clustering was plotted for the distribution of metabolites analyzed by liquid chromatography-mass spectrophotometry (LC-MS).

**FIGURE 10:**
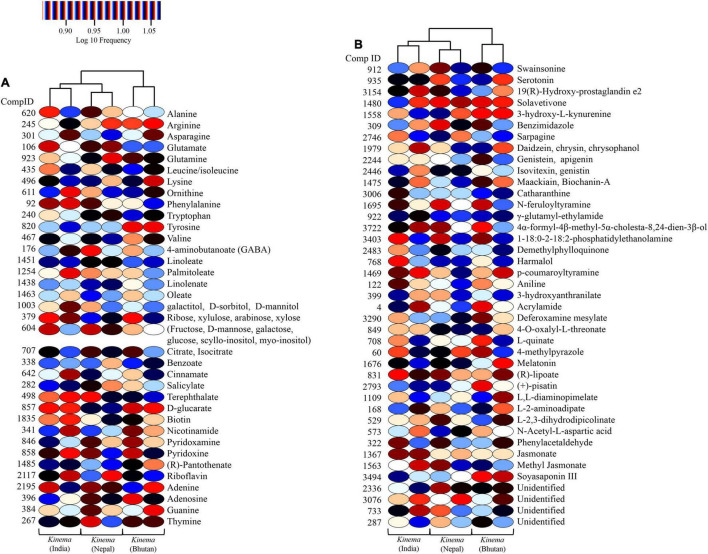
Heatmap representation of significant metabolites (*p* < 0.05) in *kinema* samples. **(A)** Significant primary metabolites include amino acids, fatty acids, sugars, organic acids, vitamins, and nucleobases. **(B)** Significant secondary metabolites such as isoflavones, soyasaponin, flavoring agents, and other bioactive compounds with potential therapeutic properties.

**FIGURE 11:**
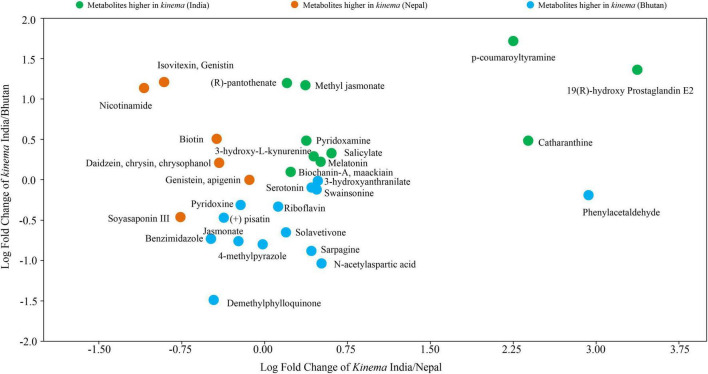
The abundance of secondary metabolites, vitamins, flavoring agents, and other bioactive compounds of *kinema* based on the log-transformed fold change, and the metabolites were found significantly different (*p* < 0.05) among samples.

A total of 361 metabolites were identified using the PlantCyc database. PCoA scores were plotted with a total cumulative variance of 77.1% (PCoA-1, 54.7%; PCoA-2, 22.5%) with distinct formation of metabolites (Figure 9A). The hierarchical clustering analysis of significant metabolites revealed the formation of two clusters (Figure 9B), which followed the same pattern as shown in the microbial distribution. Cluster I constituted *kinema* from India and Nepal and was further sub-divided into two sub-clusters, clusters IA and IB, respectively. Cluster II constituted *kinema* from Bhutan and was observed to be distinctly different from the other samples. In metabolomic analysis, 36 primary metabolites (Figure 10A and Supplementary Table 20A) and 37 secondary metabolites were identified (Figure 10B and Supplementary Table 20B). The presence of isoflavones and various bioactive compounds associated with immunomodulatory effects, immuno-stimulatory effects, phytoalexins, and anti-viral properties was detected (Table 2). The abundance of secondary metabolites and other bioactive compounds was compared among *kinema* samples based on log-transformed fold change (Figure 11). Among isoflavones identified in *kinema*, daidzein, chrysin, and chrysophanol were significantly (*p* = 0.014651) higher in Nepal than in India and Bhutan. Other than isoflavones, compounds such as p-coumaroyltyramine, melatonin, 3-hydroxy-L-kynurenine, and catharanthine were higher in India compared to Bhutan and Nepal (*p* < 0.05). Serotonin, solavetivone, swainsonine, N-acetylaspartic acid, and sarpagine were higher (*p* < 0.05) in Bhutan than in India and Nepal, and soyasaponin III was found to be higher in Nepal than Bhutan and India (*p* = 0.0000396). The metabolites with at least fourfold differences among samples from different locations were detected (Figure 11 and Supplementary Table 21).

**TABLE 2:** Metabolites with potential immunomodulatory effects, other bioactive compounds, soyasaponin, and vitamins (*p* < 0.05).

Sl. No.	Metabolites	Average log (*x*_*i*_ + 1) abundance with standard deviation (avg ± SD)		
		
		*Kinema* (India)	*Kinema* (Nepal)	*Kinema* (Bhutan)
**Potential immunomodulator**				
1	Daidzein, chrysin, and chrysophanol	9.32 ± 0.06	9.44 ± 0.10	9.26 ± 0.10
2	(+)-Pisatin	6.39 ± 0.03	6.49 ± 0.11	6.53 ± 0.05
3	Genistein and apigenin	8.72 ± 0.0008	8.76 ± 0.03	8.71 ± 0.15
4	Isovitexin and genistin	8.17 ± 0.10	8.28 ± 0.003	8.16 ± 0.02
5	Maackiain and biochanin-A	8.64 ± 0.07	8.51 ± 0.05	8.66 ± 0.05
6	Swainsonine	7.73 ± 0.13	7.59 ± 0.14	7.84 ± 0.13
7	Serotonin	8.00 ± 0.01	7.87 ± 0.09	8.02 ± 0.08
8	19 (R)-Hydroxy-prostaglandin e2	7.22 ± 0.03	6.20 ± 0.04	6.80 ± 0.05
9	Solavetivone	6.68 ± 0.10	6.62 ± 0.16	6.87 ± 0.18
10	3-Hydroxy-L-kynurenine	7.60 ± 0.08	7.47 ± 0.08	7.52 ± 0.001
11	Benzimidazole	6.56 ± 0.05	6.71 ± 0.04	6.78 ± 0.10
12	Sarpagine	7.07 ± 0.08	6.96 ± 0.08	7.24 ± 0.15
13	Catharanthine	7.54 ± 0.11	6.83 ± 0.0007	7.40 ± 0.02
**Other bioactive compounds**				
14	4-Methylpyrazole	7.75 ± 0.08	7.76 ± 0.05	7.99 ± 0.09
15	Melatonin	7.70 ± 0.04	7.44 ± 0.01	7.56 ± 0.06
16	(R)-Lipoate	8.95 ± 0.05	8.98 ± 0.06	8.99 ± 0.05
17	3-Hydroxyanthranilate	7.62 ± 0.09	7.48 ± 0.10	7.63 ± 0.05
18	Harmalol	8.44 ± 0.07	8.28 ± 0.08	8.35 ± 0.17
19	p-Coumaroyltyramine	7.78 ± 0.02	7.09 ± 0.09	7.26 ± 0.03
**Soyasaponin**				
20	Soyasaponin III	7.37 ± 0.06	7.60 ± 0.02	7.51 ± 0.01
**Vitamins**				
21	Biotin (vitamin B7)	8.38 ± 0.01	8.50 ± 0.10	8.22 ± 0.12
22	Nicotinamide (vitamin B3)	7.86 ± 0.11	8.19 ± 0.03	7.52 ± 0.05
23	Pyridoxamine (vitamin B6)	8.37 ± 0.15	8.36 ± 0.06	8.23 ± 0.14
24	Pyridoxine (vitamin B6)	7.36 ± 0.15	7.44 ± 0.07	7.47 ± 0.05
25	(R)-Pantothenate (vitamin B5)	8.87 ± 0.02	8.81 ± 0.02	8.50 ± 0.14
26	Riboflavin (vitamin B2)	7.48 ± 0.04	7.44 ± 0.10	7.55 ± 0.23
27	Demethylphylloquinone (vitamin K)	6.57 ± 0.04	6.63 ± 0.06	7.25 ± 0.12

### Correlation of Metabolites With Abundant Metagenomes

**FIGURE 12:**
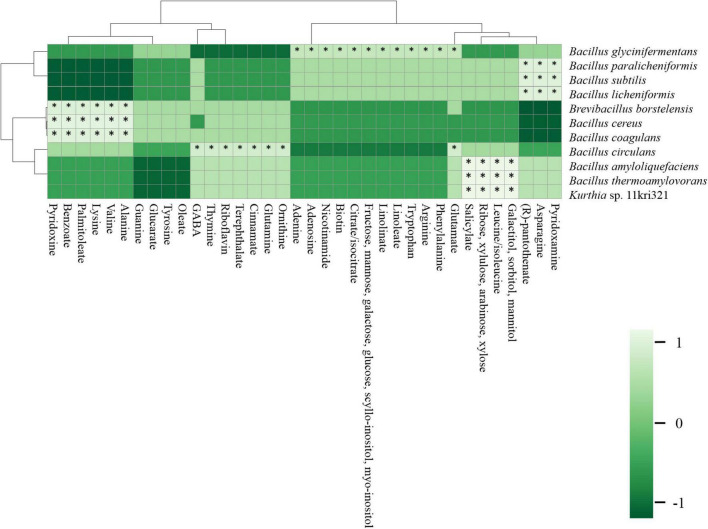
A correlation analysis between abundant species (> 1%) and primary metabolites (amino acids, fatty acids, organic acids, sugars, vitamins, and nucleobases) using Spearman’s correlation constructed by Clustvis.

The correlation between the abundant species and primary metabolites (amino acids, fatty acids, sugars, organic acids, vitamins, and nucleobases) was constructed with a bivariate result obtained from Spearman’s correlation (Figure 12). *B. subtilis*, *B. paralicheniformis*, and *B. licheniformis* were positively correlated (*p* < 0.01) with arginine, phenylalanine, glutamate, leucine, isoleucine, tryptophan, and 4-aminobutanoate (GABA) among amino acids; linoleate and linolenate among fatty acids; galactitol, sorbitol, mannitol, ribose, xylulose, arabinose, xylose, fructose, mannose, galactose, glucose, L-gulose, scyllo-inositol, and myo-inositol among sugars; and citrate, isocitrate, and salicylate among organic acids. *B. borstelensis*, *B. cereus*, and *B. coagulans* were positively correlated (*p* < 0.01) with alanine, valine, lysine, palmitoleate, benzoate, and pyridoxine. Furthermore, the correlation analysis observed the formation of galactitol, sorbitol, mannitol, ribose, xylulose, arabinose, xylose, leucine/isoleucine, and salicylate with *B. amyloliquefaciens*, *B. thermoamylovorans*, and *Kurthia* sp. 11kri321 (*p* < 0.01). *B. circulans* was found to be significant and positively correlated (*p* < 0.01) with ornithine, glutamine, cinnamate, terephthalate, riboflavin, thymine, GABA, and glutamate. *B. glycinifermentans* was the only species found to be positively correlated (*p* < 0.01) with the compounds glutamate, phenylalanine, arginine, tryptophan, linoleate, linolinate, fructose, mannose, galactose, glucose, scyllo-inositol, myo-inositol, citrate/isocitrate, biotin, nicotinamide, adenosine, and adenine.

**FIGURE 13:**
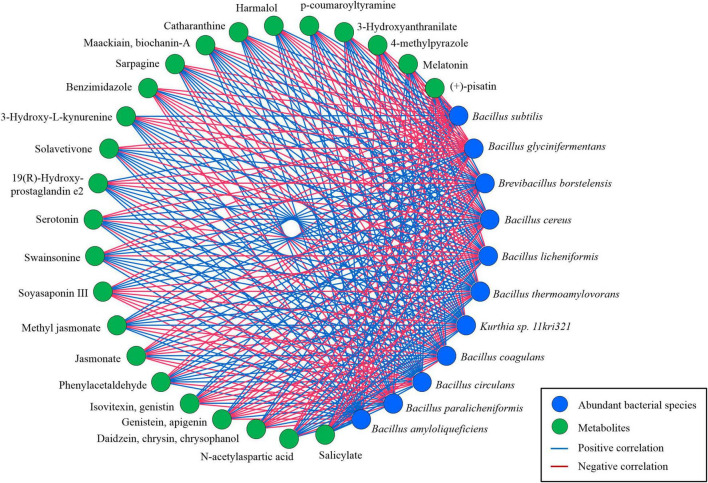
The correlation-based network between predominant microbial species (> 1%) and significant secondary metabolites based on a non-parametric Spearman’s correlation data constructed using MetScape v3.1.3 (Cytoscape v3.8).

Similarly, the relationship of abundant species identified in *kinema* with secondary metabolites and other bioactive compounds was explored using a bivariate analysis results obtained from Spearman’s correlation (Figure 13). The correlation of *B. glycinifermentans* with the formation of daidzein, chrysin, chrysophanol, genistein, apigenin, isovitexin, and genistin was positively significant (*p* < 0.01). *B. borstelensis*, *B. cereus*, and *B. coagulans* were positively correlated (*p* < 0.01) with the formation of jasmonate, benzimidazole, demethylphylloquinone, 4-methylpyrazole, and (+)-pisatin. Phenylacetaldehyde, swainsonine, serotonin, solavetivone, sarpagine, maackiain, biochanin-A, 3-hydroxyanthranilate, and N-acetyl-L-aspartic acid showed a positive correlation (*p* < 0.01) with *B. circulans*, whereas 19 (R)-hydroxy-prostaglandin e2, 3-hydroxy-L-kynurenine, catharanthine, harmalol, p-coumaroyltyramine, melatonin, and salicylate showed a positive correlation (*p* < 0.01) with *B. thermoamylovorans*, *Kurthia* sp. 11kri321, and *B. amyloliquefaciens*. Among other metabolites, methyl jasmonate was the only metabolite showing a significant correlation (*p* < 0.01) with *B. subtilis*, *B. licheniformis*, and *B. paralicheniformis*.

## Discussion

### Microbial Structure

Complex microbial community of major or minor domains exists or co-exists during uncontrolled natural fermentation of plant or animal substrates into fermented foods (Capozzi et al., 2017; Tamang et al., 2020). *Kinema* is also a fermented product of uncontrolled natural fermentation of soybean with existence of diverse microbial communities. The amplicon sequence of OTUs of *kinema* samples were earlier analyzed targeting the 16S rRNA gene for bacteria and ITS gene for fungi and yeasts (Kharnaior and Tamang, 2021). However, the profile of the microbial domains that appeared during *kinema* fermentation is important to know the entire microbial community structure of naturally fermented *kinema*. Hence, the shotgun metagenomic analysis was performed to explore the microbial community structure of *kinema* samples collected from India, Nepal, and Bhutan. DNA from the samples of the three countries were pooled in equal quantity and were used for metagenomic analysis. Pooled DNA samples were not significantly (*p* > 0.1) different in terms of sequence variation within the sample, since the pooled sequence could represent an actual sequence from each initial DNA sample (Anand et al., 2016).

In the organismal diversity, classified bacteria were the most abundant domain in *kinema* metagenomes, which was also observed in other naturally fermented soybean foods such as *pe poke* of Myanmar (Tamang et al., 2021b) and *cheonggukjang* of Korea (Tamang et al., 2022). In terms of relative abundance and occurrence, a colossal diversity of species of *Bacillus* was detected in *kinema* samples with 280 species including those detected at < 1% abundances. Previously, by amplicon sequence analysis, only 27 species of *Bacillus* were reported in *kinema* samples (Kharnaior and Tamang, 2021). Depth learning of genome mining of *kinema* has depicted huge microbial ecology, which is possible only by metagenomics using random shotgun sequence of DNA to highlight the higher resolution on taxonomic analyses at low abundance genera or species level (Tovo et al., 2020). Intra/inter-species diversity of *Bacillus*, both culturable and unculturable, in *kinema* exists and co-exists for the survival and competition among different species. The dominance of *Bacillus* in fermented soybean foods is probably due to the alkalinity of the product during fermentation (Gopikrishna et al., 2021) and also its proteolytic activity (Nguyen and Nguyen, 2020). Predominant *Bacillus* with different species has been reported in other Asian fermented soybean foods such as *cheonggukjang* (Nam et al., 2012; Tamang et al., 2022), *natto* (Kubo et al., 2011), *pe poke* (Tamang et al., 2021b), *douchi* (Hu et al., 2019), and *thua nao* (Pakwan et al., 2020). *B. subtilis* does not cause any health risk to consumers; hence, it is a safe and beneficial bacterium (Su et al., 2020). Besides *B. subtilis*, the next abundant species of *Bacillus* detected in *kinema* metagenome was *B. glycinifermentans*, which is known to produce many bioactive compounds (Stadermann et al., 2017). Some other major beneficial species of *Bacillus*, namely, *B. thermoamylovorans*, a thermostable enzyme producer (Yamada et al., 2017), *B. coagulans*, a probiotic bacterium (Cao et al., 2020), and *B. licheniformis*, functional bacterium (Muras et al., 2021), were also detected in *kinema* samples. However, the abundant species of *Bacillus* in samples of Bhutan was *B. cereus*, which was also detected by amplicon sequence analysis of *kinema* samples (Kharnaior and Tamang, 2021). *B. cereus* might have existed in fermenting soybeans during the uncontrolled spontaneous fermentation. Though *B. cereus* is a food-borne pathogen, its pathogenicity varies from harmless to lethal (Kavanaugh et al., 2022). *B. borstelensis* was also an abundant rod-shaped and Gram-positive bacterium in *kinema* samples, which is mostly thermoresistant, enzyme producing, and alkaline tolerant (Cotta et al., 2021). About 121 species of LAB (<1% relative abundance) were also detected from *kinema* samples, showing their co-existence with *Bacillus* spp. Although their abundance is low (<1%), their existence in naturally fermented soybean food may have fermentative or other functional roles, which may be investigated further.

Abundance of viruses, mostly bacteriophages, was only 1.85% in *kinema* metagenomes; their appearance might suppress the growth of some pathogenic bacterial species, which were detected in low abundance, probably combating their multiplication for food safety to extend the shelf life of the product (Endersen and Coffey, 2020). Few archaeal species were also detected in *kinema*; though their abundance was very low, their contributions to health and disease remain unknown (Adam et al., 2017).

Microbial communities in *kinema* samples of Bhutan showed the highest diversity index compared to those of India and Nepal, probably due to environmental factors (Lee S. et al., 2017), seasonal changes (Kumar et al., 2019), geographical locations (Lee et al., 2019), and inconsistency in traditional preparation practices adopted by the local people. We observed that a set of species was common and unique to each country, and the reason could be the unusual associations of microbial communities and environmental conditions including temperature and humidity. However, the predominance of beneficial microbes with various antimicrobial properties could be the reason for the reduction of the abundance of unwanted microorganisms.

### Predictive Functional Profiles

The predictive functional analysis of *kinema* metagenomes by KEGG annotation revealed the abundance of several metabolic pathways, where carbohydrate metabolism was predominant. The abundance of genes related to galactose metabolism could involve in the degradation of complex sugars in soybeans during fermentation (Chun et al., 2021). In amino acid metabolism, the abundance of genes associated to sub-pathways alanine, aspartate, and glutamate metabolism in *kinema* metagenome could enhance the aroma and flavor of the product (Yi and Hong, 2021). Glutamate is known as the precursor for the biosynthesis of GABA (Dhakal et al., 2012), which has several bio-functional properties (Diez-Gutiérrez et al., 2020). The presence of genes related to branched-chain amino acids was identified, which could be involved in the metabolism of carbon or nitrogen by *B. subtilis* (Belitsky, 2015). β-glucosidase detected in *kinema* could be involved in the biosynthesis of isoflavones and hydrolysis of oligosaccharides (Khosravi and Razavi, 2021).

### Metabolite Profiles

Metabolite profiled by LC-MS analysis revealed the presence of several bioactive compounds indicating *kinema* has several bio-functional properties, which may impart health-promoting benefits to the consumers. Although raw soybeans have various functional compounds, the presence of microorganisms can enhance the levels of production of amino acids and secondary metabolites (Hyeon et al., 2020). The abundance of *B. subtilis* during the natural fermentation processes of *kinema* could enhance the nutrient content because of the ability to release hydrolase enzymes that breaks down macromolecules into small molecules and increases the content of metabolites, as also observed in *cheonggukjang* (Lee et al., 2017; Ali et al., 2018). Some metabolites such as amino acid, namely, alanine, leucine, isoleucine, phenylalanine, and valine, and phenylacetaldehyde were identified in metabolomic analysis of *kinema*, which are known to contribute to taste and flavor development in the product (Lioe et al., 2018). Different types of isoflavones such as daidzein and genistein were detected in *kinema*, which are mostly present in soybeans (Choi et al., 2020a,b) and are associated with many health-promoting or immune-stimulating properties (Kim, 2021). The content of isoflavones in *kinema* could be indirectly influenced by the presence of microbes in the rhizosphere of soybean plants that secretes various secondary metabolites, since soybean is known to produce specialized metabolites such as isoflavones and soyasaponins (Sugiyama, 2019). Group B soyasaponin (soyasaponin III) was also detected in *kinema* samples, which has an antitumor effect, anti-inflammatory activity, and antimicrobial and cardiovascular-protective activities (Omar et al., 2020). Metabolomics result revealed the presence of vitamin B complex such as biotin, riboflavin, nicotinamide, pyridoxamine, pyridoxine, and (R)-pantothenate in *kinema*, which are the sources of micronutrients required for bio-functional activities including host immunity (Yoshii et al., 2019). Vitamin K, also known as demethylphylloquinone, was also detected in *kinema*, which has an anti-inflammatory effect (Harshman and Shea, 2016). The presence of metabolites such as lipoate, 3-hydroxyanthranilate, and melatonin in *kinema* may be related to antioxidant activities (Galano et al., 2016), and detection of chrysophanol in *kinema* may have immuno-stimulating effects, anti-inflammatory effects, and anticancer activities (Wen et al., 2018; Prateeksha et al., 2019). Also, p-coumaroyltyramine and 4-methylpyrazole were detected in *kinema*, and these bio-active compounds are used as anti-diabetic and anti-acetylcholinesterase (Masi et al., 2021). Benzimidazole, as an anti-inflammatory agent (Hashem and Bakri, 2021), is also detected in *kinema* samples. Among the samples, *kinema* samples of Bhutan depicted a higher diversity and secondary metabolites comparable to those of India and Bhutan. Therefore, we believe that microorganisms play a crucial role in secreting and changing the chemical diversity of various metabolites under certain environmental conditions including temperature and pH. We also observed that the metabolite abundance differs among *kinema* from different locations, which suggested that the differences in cultivars or soybean seeds may vary the composition of metabolites (Wang et al., 2019), and also, the formation of metabolites could enhance or affect the accumulation of secondary metabolites by various environmental factors such as regional variation, climates, and methods of preparation (Iqbal et al., 2018).

### Validation of Metabolomics With Metagenomics

Predictive functionality generated by KEGG annotation is a hypothetical claim, and prediction of genes is associated with functional profiles. Validation with real-time experiment may verify the predictive metabolic pathways to claim the presence or absence of some metabolites in the samples. Hence, we compared the KEGG database generated by metabolomics analysis with that of predictive metabolic pathways of the abundant metagenomes in *kinema* samples of India, Nepal, and Bhutan. Stickiness in *kinema* is due to γ-polyglutamic acid (γ-PGA) (Chettri et al., 2016), which determines the quality of the product preferred by the consumers. *B. subtilis* and *B. licheniformis* were both correlated with glutamate production in *kinema*, probably associated with γ-PGA production (Cai et al., 2017; Zhang C. et al., 2018). The positive correlation between *B. amyloliquefaciens* and many amino acids in *kinema* may enhance the antioxidant activity and formation of bioactive compounds and increase the levels of isoflavones (Shahzad et al., 2020). *B. coagulans* showed a positive correlation with tryptophan, tyrosine, and genistin in this study, which may improve the amino acid absorption (Stecker et al., 2020). The correlation between amino acids and *B. borstelensis* observed in *kinema* metagenomes might be the ability of thermostable D-amino acid amidase to produce amino acids at higher temperature (Baek, 2003). Unsaturated fatty acids (UFAs) such as oleate, linoleate, and linolenate were detected in *kinema*, the biosynthetic pathway of which were also predicted in *kinema* metagenomes. UFAs are important untargeted metabolites in fermented soybean foods (Park and Kim, 2021), which play an important role in biotic and abiotic stresses (He et al., 2020). The co-existence of *B. subtilis* with some fungi in *kinema* might be correlated with increase of unsaturated fatty acids in fermented soybeans (Kanghae et al., 2017). Pantothenate and pyridoxamine were positively correlated with *B. subtilis* strain, which may enhance the biosynthesis of vitamins and cofactors in *kinema*, imparting health-promoting benefits to consumers. Serotonin, a neurotransmitter (Sahu et al., 2018), was found to be correlated with the neuroactive ligand–receptor interaction *via* predictive function, and it could control the signaling pathways either intracellularly by serotonylation or extracellularly *via* membrane receptors (Bockaert et al., 2021). Swainsonine predicted in KEGG database was also detected in metabolomics analysis in *kinema* samples, which is involved in the biosynthesis of tropane, piperidine, and pyridine alkaloid (Li and Lu, 2019). A positive correlation between the predominant *Bacillus* species and the secondary metabolites such as aglycones and glycosides was observed, with the potential ability of *Bacillus* species in producing secondary metabolites that mediate antibiosis and exhibit as antimicrobial compounds (Caulier et al., 2019). A variation in microbial and metabolite formation in naturally fermented *kinema* samples of three different countries has been observed, and this may be due to different cultivars of soybean seeds used for fermentation, soil types, geographical and ago-climatic variation, and community- or region-specific conventional methods of preparation. However, the results obtained from the metagenome–metabolite interaction by correlation analysis may provide a better understanding in the selection of strains or targeting metabolites of interest for further studies.

## Conclusion

The novel outcome of this study is the metabolomics-driven metagenome mining of *kinema*, a naturally fermented low-cost plant high protein food in local diet of India, Nepal, and Bhutan, with more than 1,560 species of microbial communities, and the detection of several targeted and untargeted metabolites. The presence of metabolites such as chrysin, swainsonine, and 3-hydroxy-L-kynurenine (anticancer activity); benzimidazole (antimicrobial and anticancer); and compounds with immunomodulatory effects in *kinema* supports its therapeutic potential. Therefore, this study proves that *kinema*, a unique Himalayan fermented soybean food, is a rich source of several bioactive compounds, immunomodulators, and vitamins for nutritional supplements as well as for therapeutic uses.

## Data Availability Statement

The metagenomic sequences of *kinema* were submitted and available in the National Center for Biotechnology Information (NCBI) under the Bio-project ID PRJNA695113 (https://www.ncbi.nlm.nih.gov/bioproject/PRJNA695113) with the Sequence Read Archive (SRA) numbers SRR13573809 *kinema* (India) (https://www.ncbi.nlm.nih.gov/sra/SRR13573809), SRR13573808 *kinema* (Bhutan) (https://www.ncbi.nlm.nih.gov/sra/SRR13573808), and SRR13573807 *kinema* (Nepal) (https://www.ncbi.nlm.nih.gov/sra/SRR13573807).

## Author Contributions

PK: methodology, software, investigation, data curation, and writing—original draft preparation. JT: conceptualization, visualization, supervision, validation, and writing—reviewing and editing. Both authors contributed to the article and approved the submitted version.

## Conflict of Interest

The authors declare that the research was conducted in the absence of any commercial or financial relationships that could be construed as a potential conflict of interest.

## Publisher’s Note

All claims expressed in this article are solely those of the authors and do not necessarily represent those of their affiliated organizations, or those of the publisher, the editors and the reviewers. Any product that may be evaluated in this article, or claim that may be made by its manufacturer, is not guaranteed or endorsed by the publisher.
