# Supplementary material for: Metagenomic-Metabolomic Mining of Kinema, a Naturally Fermented Soybean Food of the Eastern Himalayas
Source: Front Microbiol. 2022 Apr 29;13:868383. doi: 10.3389/fmicb.2022.868383 (PMC9106393; doi:10.3389/fmicb.2022.868383)
Supplement: Supplementary file 1 [file Table_1.DOCX]

| **Supplementary Table 1: Geographical details on sample collection sites from the Eastern Himalayan regions** | | | | | |
| --- | --- | --- | --- | --- | --- |
| **Country** | **State/ Province** | **Collection Site (No. of samples)** | **Altitude (meter)** | **Latitude** | **Longitude** |
| India | Sikkim | Gangtok (2) | 1650 | 27.3314° N | 88.6138° E |
| India | West Bengal | Darjeeling (2) | 2042 | 27.0410° N | 88.2663° E |
| India | West Bengal | Kalimpong (2) | 1247 | 27.0594° N | 88.4695° E |
| Nepal | Sunsari | Dharan (3) | 371 | 26.8065° N | 87.2846° E |
| Nepal | Ilam | Ilam (3) | 1205 | 26.9112° N | 87.9237° E |
| Bhutan | Samtse | Samtse (6) | 417 | 26.9131° N | 89.0836° E |

| **Supplementary Table 2\| Minor phyla with a relative abundance of less than 1%.** | | | | | |
| --- | --- | --- | --- | --- | --- |
| Sl. No. | Phylum | Relative Abundance (%) | | | Domain |
|  |  | *Kinema*  (India) | *Kinema*  (Nepal) | *Kinema*  (Bhutan) |  |
| 1 | *Bacteroidetes* | 0.217803 | 0.141717 | 1.19815 | Bacteria |
| 2 | *Actinobacteria* | 0.383393 | 0.107312 | 0.727146 | Bacteria |
| 3 | *Ascomycota* | 0.116361 | 0.033586 | 0.045036 | Eukaryota |
| 4 | *Chloroflexi* | 0.028344 | 0.027033 | 0.016889 | Bacteria |
| 5 | *Acidobacteria* | 0.01641 | 0.027033 | 0.017827 | Bacteria |
| 6 | *Verrucomicrobia* | 0.023869 | 0.01966 | 0.009383 | Bacteria |
| 7 | *Planctomycetes* | 0.01641 | 0.018022 | 0.015012 | Bacteria |
| 8 | *Nitrospirae* | 0.020885 | 0.017203 | 0.010321 | Bacteria |
| 9 | *Cyanobacteria* | 0.004475 | 0.016384 | 0.011259 | Bacteria |
| 10 | *Mucoromycota* | 0.008951 | 0.00983 | 0.011259 | Eukaryota |
| 11 | *Euryarchaeota* | 0.007459 | 0.015564 | 0.006568 | Archaea |
| 12 | *Gemmatimonadetes* | 0.013426 | 0.007373 | 0.003753 | Bacteria |
| 13 | *Tenericutes* | 0.004475 | 0.002458 | 0.015012 | Bacteria |
| 14 | *Spirochaetes* | 0.004475 | 0.007373 | 0.008444 | Bacteria |
| 15 | *Ciliophora* | 0.010443 | 0.002458 | 0.00563 | Eukaryota |
| 16 | *Chlamydiae* | 0.005967 | 0.004915 | 0.00563 | Bacteria |
| 17 | *Basidiomycota* | 0.005967 | 0.004096 | 0.001877 | Eukaryota |
| 18 | *Thaumarchaeota* | 0.005967 | 0.003277 | 0.001877 | Archaea |
| 19 | *Fusobacteria* | 0.004475 | 0.001638 | 0.004691 | Bacteria |
| 20 | *Armatimonadetes* | 0.004475 | 0.004096 | 0.000938 | Bacteria |
| 21 | *Lentisphaerae* | 0.007459 | 0.001638 | 0 | Bacteria |
| 22 | *Elusimicrobia* | 0.002984 | 0.004096 | 0.001877 | Bacteria |
| 23 | *Thermotogae* | 0.004475 | 0.001638 | 0.001877 | Bacteria |
| 24 | *Ignavibacteriae* | 0.001492 | 0.003277 | 0.002815 | Bacteria |
| 25 | *Deinococcus-Thermus* | 0.002984 | 0.000819 | 0.003753 | Bacteria |
| 26 | *Synergistetes* | 0.002984 | 0.001638 | 0.002815 | Bacteria |
| 27 | *Bacillariophyta* | 0.001492 | 0.000819 | 0.003753 | Eukaryota |
| 28 | *Chlorobi* | 0.001492 | 0 | 0.002815 | Bacteria |
| 29 | *Chlorophyta* | 0.002984 | 0 | 0.000938 | Eukaryota |
| 30 | *Chytridiomycota* | 0.001492 | 0.001638 | 0 | Eukaryota |
| 31 | *Oomycota* | 0.002984 | 0 | 0 | Eukaryota |
| 32 | *Apicomplexa* | 0 | 0 | 0.002815 | Eukaryota |
| 33 | *Amoebozoa* | 0.001492 | 0 | 0.000938 | Eukaryota |
| 34 | *Calditrichaeota* | 0 | 0.000819 | 0.000938 | Bacteria |
| 35 | *Kiritimatiellaeota* | 0.001492 | 0 | 0 | Bacteria |
| 36 | *Thermodesulfobacteria* | 0.001492 | 0 | 0 | Bacteria |
| 37 | *Cercozoa* | 0.001492 | 0 | 0 | Eukaryota |
| 38 | *Ochrophyta* | 0.001492 | 0 | 0 | Eukaryota |
| 39 | *Rhodophyta* | 0.001492 | 0 | 0 | Eukaryota |
| 40 | *Aquificae* | 0 | 0 | 0.000938 | Bacteria |
| 41 | *Nitrospinae* | 0 | 0 | 0.000938 | Bacteria |
| 42 | *Crenarchaeota* | 0 | 0 | 0.000938 | Archaea |
| 43 | *Cryptomycota* | 0 | 0 | 0.000938 | Eukaryota |
| 44 | *Euglenozoa* | 0 | 0 | 0.000938 | Eukaryota |
| 45 | *Zoopagomycota* | 0 | 0.000819 | 0 | Eukaryota |
| 46 | unclassified bacterial phyla | 0.108902 | 0.1196 | 0.074122 | Bacteria |
| 47 | unclassified archaea phyla | 0.001492 | 0.001638 | 0 | Archaea |
| 48 | unclassified eukaryotic phyla | 0.001492 | 0.001638 | 0.002815 | Eukaryota |
