# Supplementary material for: Metagenomic-Metabolomic Mining of Kinema, a Naturally Fermented Soybean Food of the Eastern Himalayas
Source: Front Microbiol. 2022 Apr 29;13:868383. doi: 10.3389/fmicb.2022.868383 (PMC9106393; doi:10.3389/fmicb.2022.868383)
Supplement: Supplementary file 2 [file Table_2.DOCX]

| **Supplementary Table 3\| Minor families with a relative abundance of less than 1%.** | | | | | |
| --- | --- | --- | --- | --- | --- |
| Sl. No. | Family | Relative Abundance (%) | | | Domain |
|  |  | *Kinema* (India) | *Kinema* (Nepal) | *Kinema* (Bhutan) |  |
| 1 | *Staphylococcaceae* | 1.989976 | 0.221027 | 0.286849 | Bacteria |
| 2 | *Alcaligenaceae* | 0.319379 | 0.017002 | 1.963813 | Bacteria |
| 3 | *Lactobacillaceae* | 0.429114 | 0.149439 | 1.226572 | Bacteria |
| 4 | *Flavobacteriaceae* | 0.055687 | 0.030425 | 1.440735 | Bacteria |
| 5 | *Enterobacteriaceae* | 0.289898 | 0.041163 | 0.711282 | Bacteria |
| 6 | *Clostridiaceae* | 0.342309 | 0.23445 | 0.310212 | Bacteria |
| 7 | *Corynebacteriaceae* | 0.258779 | 0.014318 | 0.594466 | Bacteria |
| 8 | *Thermoactinomycetaceae* | 0.055687 | 0.716772 | 0.040237 | Bacteria |
| 9 | *Streptococcaceae* | 0.119562 | 0.121699 | 0.530865 | Bacteria |
| 10 | *Siphoviridae* | 0.445493 | 0.089485 | 0.007788 | Viruses |
| 11 | *Carnobacteriaceae* | 0.152319 | 0.104697 | 0.163543 | Bacteria |
| 12 | *Listeriaceae* | 0.113011 | 0.051006 | 0.11422 | Bacteria |
| 13 | *Comamonadaceae* | 0.062238 | 0.043847 | 0.170033 | Bacteria |
| 14 | *Mycobacteriaceae* | 0.026205 | 0.024161 | 0.183012 | Bacteria |
| 15 | *Lachnospiraceae* | 0.093357 | 0.063534 | 0.044131 | Bacteria |
| 16 | *Sporolactobacillaceae* | 0.050773 | 0.085905 | 0.051918 | Bacteria |
| 17 | *Leuconostocaceae* | 0.099908 | 0.061744 | 0.014278 | Bacteria |
| 18 | *Pseudomonadaceae* | 0.039308 | 0.011633 | 0.099943 | Bacteria |
| 19 | *Burkholderiaceae* | 0.034395 | 0.016107 | 0.061004 | Bacteria |
| 20 | *Rhodocyclaceae* | 0.03767 | 0.026845 | 0.045429 | Bacteria |
| 21 | *Pneumocystidaceae* | 0.080254 | 0.02774 | 0 | Eukaryota |
| 22 | *Ruminococcaceae* | 0.040946 | 0.024161 | 0.040237 | Bacteria |
| 23 | *Rhodobacteraceae* | 0.044222 | 0.010738 | 0.038939 | Bacteria |
| 24 | *Nocardiaceae* | 0.003276 | 0.005369 | 0.080473 | Bacteria |
| 25 | *Vibrionaceae* | 0.034395 | 0.00179 | 0.049322 | Bacteria |
| 26 | *Cyclobacteriaceae* | 0.040946 | 0.028635 | 0.011682 | Bacteria |
| 27 | *Aerococcaceae* | 0.052411 | 0.00179 | 0.022065 | Bacteria |
| 28 | *Peptostreptococcaceae* | 0.021292 | 0.010738 | 0.040237 | Bacteria |
| 29 | *Erysipelotrichaceae* | 0.021292 | 0.006264 | 0.041535 | Bacteria |
| 30 | *Yersiniaceae* | 0.034395 | 0.003579 | 0.028555 | Bacteria |
| 31 | *Pichiaceae* | 0.008189 | 0.003579 | 0.053216 | Eukaryota |
| 32 | *Sphingobacteriaceae* | 0.013103 | 0.003579 | 0.048025 | Bacteria |
| 33 | *Oceanospirillaceae* | 0.021292 | 0.003579 | 0.032449 | Bacteria |
| 34 | *Rhodospirillaceae* | 0.02293 | 0.011633 | 0.022065 | Bacteria |
| 35 | *Peptococcaceae* | 0.009827 | 0.025056 | 0.020767 | Bacteria |
| 36 | *Micrococcaceae* | 0.029481 | 0.009843 | 0.015576 | Bacteria |
| 37 | *Oxalobacteraceae* | 0.011465 | 0.008054 | 0.031151 | Bacteria |
| 38 | *Erwiniaceae* | 0.016378 | 0.005369 | 0.028555 | Bacteria |
| 39 | *Thiotrichaceae* | 0.019654 | 0.016107 | 0.014278 | Bacteria |
| 40 | *Pasteurellaceae* | 0.024568 | 0 | 0.023363 | Bacteria |
| 41 | *Alicyclobacillaceae* | 0.014741 | 0.013423 | 0.018171 | Bacteria |
| 42 | *Pectobacteriaceae* | 0.018016 | 0.005369 | 0.022065 | Bacteria |
| 43 | *Nitrospiraceae* | 0.019654 | 0.016107 | 0.009086 | Bacteria |
| 44 | *Planctomycetaceae* | 0.014741 | 0.014318 | 0.01298 | Bacteria |
| 45 | *Peptoniphilaceae* | 0.013103 | 0.008948 | 0.018171 | Bacteria |
| 46 | *Chitinophagaceae* | 0.009827 | 0.011633 | 0.018171 | Bacteria |
| 47 | *Xanthomonadaceae* | 0.014741 | 0.003579 | 0.020767 | Bacteria |
| 48 | *Eubacteriaceae* | 0.011465 | 0.005369 | 0.022065 | Bacteria |
| 49 | *Neisseriaceae* | 0.014741 | 0.00179 | 0.022065 | Bacteria |
| 50 | *Rhizobiaceae* | 0.014741 | 0.005369 | 0.018171 | Bacteria |
| 51 | *Acetobacteraceae* | 0.014741 | 0.004474 | 0.016873 | Bacteria |
| 52 | *Streptomycetaceae* | 0.008189 | 0.014318 | 0.01298 | Bacteria |
| 53 | *Microbacteriaceae* | 0.009827 | 0 | 0.023363 | Bacteria |
| 54 | *Sphingomonadaceae* | 0.006551 | 0.008054 | 0.018171 | Bacteria |
| 55 | *Bacteroidaceae* | 0.019654 | 0.008948 | 0.003894 | Bacteria |
| 56 | *Podoviridae* | 0.031119 | 0 | 0.001298 | Viruses |
| 57 | *Mucoraceae* | 0.008189 | 0.008054 | 0.015576 | Eukaryota |
| 58 | *Acidaminococcaceae* | 0.013103 | 0.002685 | 0.015576 | Bacteria |
| 59 | *Heliobacteriaceae* | 0.006551 | 0.011633 | 0.01298 | Bacteria |
| 60 | *Brucellaceae* | 0 | 0.000895 | 0.029853 | Bacteria |
| 61 | *Halomonadaceae* | 0.013103 | 0.00179 | 0.015576 | Bacteria |
| 62 | *Porphyromonadaceae* | 0.013103 | 0.008054 | 0.009086 | Bacteria |
| 63 | *Alteromonadaceae* | 0.006551 | 0.007159 | 0.014278 | Bacteria |
| 64 | *Chromatiaceae* | 0.011465 | 0.004474 | 0.011682 | Bacteria |
| 65 | *Cytophagaceae* | 0.009827 | 0.004474 | 0.011682 | Bacteria |
| 66 | *Verrucomicrobiaceae* | 0.013103 | 0.009843 | 0.002596 | Bacteria |
| 67 | *Phyllobacteriaceae* | 0.011465 | 0.00179 | 0.010384 | Bacteria |
| 68 | *Sporomusaceae* | 0.003276 | 0.013423 | 0.00649 | Bacteria |
| 69 | *Aeromonadaceae* | 0.008189 | 0.002685 | 0.011682 | Bacteria |
| 70 | *Chromobacteriaceae* | 0.008189 | 0.002685 | 0.011682 | Bacteria |
| 71 | *Selenomonadaceae* | 0.008189 | 0.008948 | 0.005192 | Bacteria |
| 72 | *Erythrobacteraceae* | 0.009827 | 0.004474 | 0.007788 | Bacteria |
| 73 | *Bradyrhizobiaceae* | 0.003276 | 0.010738 | 0.007788 | Bacteria |
| 74 | *Methylococcaceae* | 0.011465 | 0.002685 | 0.00649 | Bacteria |
| 75 | *Legionellaceae* | 0.008189 | 0.00179 | 0.010384 | Bacteria |
| 76 | *Geobacteraceae* | 0.009827 | 0.006264 | 0.003894 | Bacteria |
| 77 | *Opitutaceae* | 0.008189 | 0.004474 | 0.00649 | Bacteria |
| 78 | *Anaerolineaceae* | 0.004914 | 0.006264 | 0.007788 | Bacteria |
| 79 | *Pseudonocardiaceae* | 0.004914 | 0.003579 | 0.010384 | Bacteria |
| 80 | *Prevotellaceae* | 0.008189 | 0.005369 | 0.005192 | Bacteria |
| 81 | *Orbaceae* | 0.008189 | 0 | 0.009086 | Bacteria |
| 82 | *Actinomycetaceae* | 0.008189 | 0.000895 | 0.007788 | Bacteria |
| 83 | *Gemmatimonadaceae* | 0.011465 | 0.005369 | 0 | Bacteria |
| 84 | *Hyphomicrobiaceae* | 0.011465 | 0.002685 | 0.002596 | Bacteria |
| 85 | *Nitrosomonadaceae* | 0.003276 | 0.005369 | 0.007788 | Bacteria |
| 86 | *Debaryomycetaceae* | 0.016378 | 0 | 0 | Eukaryota |
| 87 | *Spirochaetaceae* | 0.001638 | 0.004474 | 0.009086 | Bacteria |
| 88 | *Rhodanobacteraceae* | 0.008189 | 0.00179 | 0.005192 | Bacteria |
| 89 | *Ectothiorhodospiraceae* | 0.004914 | 0.00179 | 0.007788 | Bacteria |
| 90 | *Caulobacteraceae* | 0.008189 | 0.006264 | 0 | Bacteria |
| 91 | *Desulfobacteraceae* | 0.006551 | 0.002685 | 0.005192 | Bacteria |
| 92 | *Chlamydiaceae* | 0.003276 | 0.004474 | 0.00649 | Bacteria |
| 93 | *Hymenobacteraceae* | 0.001638 | 0.004474 | 0.007788 | Bacteria |
| 94 | *Flammeovirgaceae* | 0.008189 | 0.002685 | 0.002596 | Bacteria |
| 95 | *Defluviitaleaceae* | 0.004914 | 0.007159 | 0.001298 | Bacteria |
| 96 | *Tissierellaceae* | 0.003276 | 0.00179 | 0.007788 | Bacteria |
| 97 | *Sinobacteraceae* | 0 | 0.008948 | 0.003894 | Bacteria |
| 98 | *Oxytrichidae* | 0.006551 | 0.000895 | 0.005192 | Eukaryota |
| 99 | *Methylophilaceae* | 0.004914 | 0.003579 | 0.003894 | Bacteria |
| 100 | *Acidimicrobiaceae* | 0.004914 | 0.004474 | 0.002596 | Bacteria |
| 101 | *Cellvibrionaceae* | 0.004914 | 0.002685 | 0.003894 | Bacteria |
| 102 | *Bifidobacteriaceae* | 0.006551 | 0.003579 | 0.001298 | Bacteria |
| 103 | *Campylobacteraceae* | 0.008189 | 0.00179 | 0.001298 | Bacteria |
| 104 | *Hyphomonadaceae* | 0.004914 | 0.003579 | 0.002596 | Bacteria |
| 105 | *Hafniaceae* | 0.003276 | 0 | 0.007788 | Bacteria |
| 106 | *Desulfovibrionaceae* | 0.006551 | 0.00179 | 0.002596 | Bacteria |
| 107 | *Fusobacteriaceae* | 0.004914 | 0.00179 | 0.003894 | Bacteria |
| 108 | *Succinivibrionaceae* | 0.006551 | 0 | 0.003894 | Bacteria |
| 109 | *Desulfuromonadaceae* | 0.004914 | 0.005369 | 0 | Bacteria |
| 110 | *Brevibacteriaceae* | 0.006551 | 0.000895 | 0.002596 | Bacteria |
| 111 | *Piscirickettsiaceae* | 0.006551 | 0.000895 | 0.002596 | Bacteria |
| 112 | *Coriobacteriaceae* | 0.003276 | 0.002685 | 0.003894 | Bacteria |
| 113 | *Pseudoalteromonadaceae* | 0.003276 | 0.002685 | 0.003894 | Bacteria |
| 114 | *Dermabacteraceae* | 0.009827 | 0 | 0 | Bacteria |
| 115 | *Shewanellaceae* | 0.003276 | 0 | 0.00649 | Bacteria |
| 116 | *Holophagaceae* | 0.003276 | 0.003579 | 0.002596 | Bacteria |
| 117 | *Desulfobulbaceae* | 0.003276 | 0.000895 | 0.005192 | Bacteria |
| 118 | *Hydrogenophilaceae* | 0.003276 | 0.000895 | 0.005192 | Bacteria |
| 119 | *Veillonellaceae* | 0.004914 | 0.00179 | 0.002596 | Bacteria |
| 120 | *Acholeplasmataceae* | 0 | 0.002685 | 0.00649 | Bacteria |
| 121 | *Syntrophomonadaceae* | 0 | 0.002685 | 0.00649 | Bacteria |
| 122 | *Idiomarinaceae* | 0.006551 | 0 | 0.002596 | Bacteria |
| 123 | *Alcanivoracaceae* | 0 | 0 | 0.009086 | Bacteria |
| 124 | *Methanocaldococcaceae* | 0 | 0.008948 | 0 | Archaea |
| 125 | *Thermoanaerobacteraceae* | 0 | 0.003579 | 0.005192 | Bacteria |
| 126 | *Propionibacteriaceae* | 0 | 0.000895 | 0.007788 | Bacteria |
| 127 | *Microbulbiferaceae* | 0.004914 | 0.000895 | 0.002596 | Bacteria |
| 128 | *Intrasporangiaceae* | 0.001638 | 0.002685 | 0.003894 | Bacteria |
| 129 | *Prolixibacteraceae* | 0.003276 | 0.003579 | 0.001298 | Bacteria |
| 130 | *Synergistaceae* | 0.003276 | 0.000895 | 0.003894 | Bacteria |
| 131 | *Hahellaceae* | 0.006551 | 0 | 0.001298 | Bacteria |
| 132 | *Acidobacteriaceae* | 0.001638 | 0.003579 | 0.002596 | Bacteria |
| 133 | *Bartonellaceae* | 0.001638 | 0.000895 | 0.005192 | Bacteria |
| 134 | *Polyangiaceae* | 0.003276 | 0.00179 | 0.002596 | Bacteria |
| 135 | *Methylocystaceae* | 0.004914 | 0 | 0.002596 | Bacteria |
| 136 | *Frankiaceae* | 0.001638 | 0.00179 | 0.003894 | Bacteria |
| 137 | *Methylobacteriaceae* | 0.003276 | 0 | 0.003894 | Bacteria |
| 138 | *Caldicoprobacteraceae* | 0 | 0.004474 | 0.002596 | Bacteria |
| 139 | *Deinococcaceae* | 0.003276 | 0.000895 | 0.002596 | Bacteria |
| 140 | *Helicobacteraceae* | 0.003276 | 0.000895 | 0.002596 | Bacteria |
| 141 | *Halobacteroidaceae* | 0.001638 | 0.000895 | 0.003894 | Bacteria |
| 142 | *Sutterellaceae* | 0.003276 | 0.00179 | 0.001298 | Bacteria |
| 143 | *Methanosarcinaceae* | 0.003276 | 0.00179 | 0.001298 | Archaea |
| 144 | *Dipodascaceae* | 0.004914 | 0 | 0.001298 | Eukaryota |
| 145 | *Leptolyngbyaceae* | 0 | 0.003579 | 0.002596 | Bacteria |
| 146 | *Trichosporonaceae* | 0.003276 | 0.002685 | 0 | Eukaryota |
| 147 | *Bdellovibrionaceae* | 0.003276 | 0 | 0.002596 | Bacteria |
| 148 | *Marinilabiliaceae* | 0.003276 | 0 | 0.002596 | Bacteria |
| 149 | *Micromonosporaceae* | 0.003276 | 0 | 0.002596 | Bacteria |
| 150 | *Francisellaceae* | 0 | 0.004474 | 0.001298 | Bacteria |
| 151 | *Beijerinckiaceae* | 0 | 0.00179 | 0.003894 | Bacteria |
| 152 | *Chlorobiaceae* | 0.001638 | 0 | 0.003894 | Bacteria |
| 153 | *Anaeromyxobacteraceae* | 0.003276 | 0.000895 | 0.001298 | Bacteria |
| 154 | *Coxiellaceae* | 0.003276 | 0.000895 | 0.001298 | Bacteria |
| 155 | *Desulfurellaceae* | 0.003276 | 0.000895 | 0.001298 | Bacteria |
| 156 | *Halothiobacillaceae* | 0 | 0 | 0.005192 | Bacteria |
| 157 | *Akkermansiaceae* | 0.001638 | 0.000895 | 0.002596 | Bacteria |
| 158 | *Eggerthellaceae* | 0.001638 | 0.000895 | 0.002596 | Bacteria |
| 159 | *Nocardioidaceae* | 0.001638 | 0.000895 | 0.002596 | Bacteria |
| 160 | *Saprospiraceae* | 0.001638 | 0.000895 | 0.002596 | Bacteria |
| 161 | *Chthoniobacteraceae* | 0.003276 | 0.00179 | 0 | Bacteria |
| 162 | *Saccharomycetaceae* | 0.004914 | 0 | 0 | Eukaryota |
| 163 | *Aurantimonadaceae* | 0 | 0.000895 | 0.003894 | Bacteria |
| 164 | *Gemmataceae* | 0 | 0.000895 | 0.003894 | Bacteria |
| 165 | *Oleiphilaceae* | 0.001638 | 0.00179 | 0.001298 | Bacteria |
| 166 | *Solibacteraceae* | 0.001638 | 0.00179 | 0.001298 | Bacteria |
| 167 | *Aspergillaceae* | 0.001638 | 0.00179 | 0.001298 | Eukaryota |
| 168 | *Christensenellaceae* | 0.003276 | 0 | 0.001298 | Bacteria |
| 169 | *Spongiibacteraceae* | 0.003276 | 0 | 0.001298 | Bacteria |
| 170 | *Crocinitomicaceae* | 0 | 0.00179 | 0.002596 | Bacteria |
| 171 | *Acaryochloridaceae* | 0.001638 | 0.002685 | 0 | Bacteria |
| 172 | *Acidiferrobacteraceae* | 0.001638 | 0 | 0.002596 | Bacteria |
| 173 | *Atopobiaceae* | 0.001638 | 0 | 0.002596 | Bacteria |
| 174 | *Geodermatophilaceae* | 0.001638 | 0 | 0.002596 | Bacteria |
| 175 | *Gordoniaceae* | 0.001638 | 0 | 0.002596 | Bacteria |
| 176 | *Leptospiraceae* | 0.001638 | 0 | 0.002596 | Bacteria |
| 177 | *Mycoplasmataceae* | 0.001638 | 0 | 0.002596 | Bacteria |
| 178 | *Rikenellaceae* | 0.001638 | 0 | 0.002596 | Bacteria |
| 179 | *Ktedonobacteraceae* | 0.003276 | 0.000895 | 0 | Bacteria |
| 180 | *Rickettsiaceae* | 0.003276 | 0.000895 | 0 | Bacteria |
| 181 | *Archangiaceae* | 0 | 0.002685 | 0.001298 | Bacteria |
| 182 | *Halieaceae* | 0 | 0.002685 | 0.001298 | Bacteria |
| 183 | *Nostocaceae* | 0 | 0.002685 | 0.001298 | Bacteria |
| 184 | *Desulfonatronaceae* | 0 | 0 | 0.003894 | Bacteria |
| 185 | *Sulfuricellaceae* | 0.001638 | 0.000895 | 0.001298 | Bacteria |
| 186 | *Thalassiosiraceae* | 0 | 0.000895 | 0.002596 | Eukaryota |
| 187 | *Cryomorphaceae* | 0.001638 | 0.00179 | 0 | Bacteria |
| 188 | *Dietziaceae* | 0.001638 | 0.00179 | 0 | Bacteria |
| 189 | *Lichtheimiaceae* | 0.001638 | 0.00179 | 0 | Eukaryota |
| 190 | *Myxococcaceae* | 0 | 0.00179 | 0.001298 | Bacteria |
| 191 | *Colwelliaceae* | 0.001638 | 0 | 0.001298 | Bacteria |
| 192 | *Ferrimonadaceae* | 0.001638 | 0 | 0.001298 | Bacteria |
| 193 | *Ferrovaceae* | 0.001638 | 0 | 0.001298 | Bacteria |
| 194 | *Kosmotogaceae* | 0.001638 | 0 | 0.001298 | Bacteria |
| 195 | *Pseudobacteriovoracaceae* | 0.001638 | 0 | 0.001298 | Bacteria |
| 196 | *Thorselliaceae* | 0.001638 | 0 | 0.001298 | Bacteria |
| 197 | *Methanoregulaceae* | 0.001638 | 0 | 0.001298 | Archaea |
| 198 | *Acanthamoebidae* | 0.001638 | 0 | 0.001298 | Eukaryota |
| 199 | *Microcystaceae* | 0 | 0.002685 | 0 | Bacteria |
| 200 | *Algiphilaceae* | 0 | 0 | 0.002596 | Bacteria |
| 201 | *Immundisolibacteraceae* | 0 | 0 | 0.002596 | Bacteria |
| 202 | *Leptotrichiaceae* | 0 | 0 | 0.002596 | Bacteria |
| 203 | *Promicromonosporaceae* | 0 | 0 | 0.002596 | Bacteria |
| 204 | *Synechococcaceae* | 0 | 0 | 0.002596 | Bacteria |
| 205 | *Syntrophaceae* | 0 | 0 | 0.002596 | Bacteria |
| 206 | *Thermaceae* | 0 | 0 | 0.002596 | Bacteria |
| 207 | *Sarcocystidae* | 0 | 0 | 0.002596 | Eukaryota |
| 208 | *Chloroflexaceae* | 0.001638 | 0.000895 | 0 | Bacteria |
| 209 | *Magnetococcaceae* | 0.001638 | 0.000895 | 0 | Bacteria |
| 210 | *Nocardiopsaceae* | 0.001638 | 0.000895 | 0 | Bacteria |
| 211 | *Petrotogaceae* | 0.001638 | 0.000895 | 0 | Bacteria |
| 212 | *Methanocellaceae* | 0.001638 | 0.000895 | 0 | Archaea |
| 213 | *Nitrososphaeraceae* | 0.001638 | 0.000895 | 0 | Archaea |
| 214 | *Calditrichaceae* | 0 | 0.000895 | 0.001298 | Bacteria |
| 215 | *Catalimonadaceae* | 0 | 0.000895 | 0.001298 | Bacteria |
| 216 | *Fervidobacteriaceae* | 0 | 0.000895 | 0.001298 | Bacteria |
| 217 | *Oscillatoriaceae* | 0 | 0.000895 | 0.001298 | Bacteria |
| 218 | *Tolypothrichaceae* | 0 | 0.000895 | 0.001298 | Bacteria |
| 219 | *Borreliaceae* | 0 | 0.00179 | 0 | Bacteria |
| 220 | *Bryobacteraceae* | 0 | 0.00179 | 0 | Bacteria |
| 221 | *Chthonomonadaceae* | 0 | 0.00179 | 0 | Bacteria |
| 222 | *Lewinellaceae* | 0 | 0.00179 | 0 | Bacteria |
| 223 | *Oscillospiraceae* | 0 | 0.00179 | 0 | Bacteria |
| 224 | *Rubrobacteraceae* | 0 | 0.00179 | 0 | Bacteria |
| 225 | *Sandaracinaceae* | 0 | 0.00179 | 0 | Bacteria |
| 226 | *Xanthobacteraceae* | 0 | 0.00179 | 0 | Bacteria |
| 227 | *Acidithiobacillaceae* | 0.001638 | 0 | 0 | Bacteria |
| 228 | *Brachyspiraceae* | 0.001638 | 0 | 0 | Bacteria |
| 229 | *Cardiobacteriaceae* | 0.001638 | 0 | 0 | Bacteria |
| 230 | *Competibacteraceae* | 0.001638 | 0 | 0 | Bacteria |
| 231 | *Holosporaceae* | 0.001638 | 0 | 0 | Bacteria |
| 232 | *Kangiellaceae* | 0.001638 | 0 | 0 | Bacteria |
| 233 | *Kiritimatiellaceae* | 0.001638 | 0 | 0 | Bacteria |
| 234 | *Parvularculaceae* | 0.001638 | 0 | 0 | Bacteria |
| 235 | *Patulibacteraceae* | 0.001638 | 0 | 0 | Bacteria |
| 236 | *Pelagibacteraceae* | 0.001638 | 0 | 0 | Bacteria |
| 237 | *Psychromonadaceae* | 0.001638 | 0 | 0 | Bacteria |
| 238 | *Sneathiellaceae* | 0.001638 | 0 | 0 | Bacteria |
| 239 | *Sporichthyaceae* | 0.001638 | 0 | 0 | Bacteria |
| 240 | *Thermomicrobiaceae* | 0.001638 | 0 | 0 | Bacteria |
| 241 | *Thermomonosporaceae* | 0.001638 | 0 | 0 | Bacteria |
| 242 | *Thermotogaceae* | 0.001638 | 0 | 0 | Bacteria |
| 243 | *Waddliaceae* | 0.001638 | 0 | 0 | Bacteria |
| 244 | *Albuginaceae* | 0.001638 | 0 | 0 | Eukaryota |
| 245 | *Bacillariaceae* | 0.001638 | 0 | 0 | Eukaryota |
| 246 | *Chlorellaceae* | 0.001638 | 0 | 0 | Eukaryota |
| 247 | *Cyanidiaceae* | 0.001638 | 0 | 0 | Eukaryota |
| 248 | *Herpotrichiellaceae* | 0.001638 | 0 | 0 | Eukaryota |
| 249 | *Herpotrichiellaceae* | 0.001638 | 0 | 0 | Eukaryota |
| 250 | *Leucosporidiaceae* | 0.001638 | 0 | 0 | Eukaryota |
| 251 | *Lyophyllaceae* | 0.001638 | 0 | 0 | Eukaryota |
| 252 | *Metschnikowiaceae* | 0.001638 | 0 | 0 | Eukaryota |
| 253 | *Monodopsidaceae* | 0.001638 | 0 | 0 | Eukaryota |
| 254 | *Phaffomycetaceae* | 0.001638 | 0 | 0 | Eukaryota |
| 255 | *Plasmodiophoridae* | 0.001638 | 0 | 0 | Eukaryota |
| 256 | *Polyphysaceae* | 0.001638 | 0 | 0 | Eukaryota |
| 257 | *Pyronemataceae* | 0.001638 | 0 | 0 | Eukaryota |
| 258 | *Saprolegniaceae* | 0.001638 | 0 | 0 | Eukaryota |
| 259 | *Stentoridae* | 0.001638 | 0 | 0 | Eukaryota |
| 260 | *Tetrahymenidae* | 0.001638 | 0 | 0 | Eukaryota |
| 261 | *Amoebophilaceae* | 0 | 0 | 0.001298 | Bacteria |
| 262 | *Bacteriovoracaceae* | 0 | 0 | 0.001298 | Bacteria |
| 263 | *Blattabacteriaceae* | 0 | 0 | 0.001298 | Bacteria |
| 264 | *Budviciaceae* | 0 | 0 | 0.001298 | Bacteria |
| 265 | *Caldilineaceae* | 0 | 0 | 0.001298 | Bacteria |
| 266 | *Campylobacteraceae* | 0 | 0 | 0.001298 | Bacteria |
| 267 | *Cardiobacteriaceae* | 0 | 0 | 0.001298 | Bacteria |
| 268 | *Dermabacteraceae* | 0 | 0 | 0.001298 | Bacteria |
| 269 | *Gallionellaceae* | 0 | 0 | 0.001298 | Bacteria |
| 270 | *Gracilibacteraceae* | 0 | 0 | 0.001298 | Bacteria |
| 271 | *Halanaerobiaceae* | 0 | 0 | 0.001298 | Bacteria |
| 272 | *Haliscomenobacteraceae* | 0 | 0 | 0.001298 | Bacteria |
| 273 | *Hapalosiphonaceae* | 0 | 0 | 0.001298 | Bacteria |
| 274 | *Hydrogenothermaceae* | 0 | 0 | 0.001298 | Bacteria |
| 275 | *Kiloniellaceae* | 0 | 0 | 0.001298 | Bacteria |
| 276 | *Limnochordaceae* | 0 | 0 | 0.001298 | Bacteria |
| 277 | *Marinifilaceae* | 0 | 0 | 0.001298 | Bacteria |
| 278 | *Melioribacteraceae* | 0 | 0 | 0.001298 | Bacteria |
| 279 | *Micropepsaceae* | 0 | 0 | 0.001298 | Bacteria |
| 280 | *Microscillaceae* | 0 | 0 | 0.001298 | Bacteria |
| 281 | *Porticoccaceae* | 0 | 0 | 0.001298 | Bacteria |
| 282 | *Rhodothermaceae* | 0 | 0 | 0.001298 | Bacteria |
| 283 | *Rivulariaceae* | 0 | 0 | 0.001298 | Bacteria |
| 284 | *Salinisphaeraceae* | 0 | 0 | 0.001298 | Bacteria |
| 285 | *Streptosporangiaceae* | 0 | 0 | 0.001298 | Bacteria |
| 286 | *Thermodesulfobiaceae* | 0 | 0 | 0.001298 | Bacteria |
| 287 | *Haloferacaceae* | 0 | 0 | 0.001298 | Archaea |
| 288 | *Thermoproteaceae* | 0 | 0 | 0.001298 | Archaea |
| 289 | *Dacrymycetaceae* | 0 | 0 | 0.001298 | Eukaryota |
| 290 | *Ectocarpaceae* | 0 | 0 | 0.001298 | Eukaryota |
| 291 | *Eimeriidae* | 0 | 0 | 0.001298 | Eukaryota |
| 292 | *Fragilariaceae* | 0 | 0 | 0.001298 | Eukaryota |
| 293 | *Noelaerhabdaceae* | 0 | 0 | 0.001298 | Eukaryota |
| 294 | *Ostreobiaceae* | 0 | 0 | 0.001298 | Eukaryota |
| 295 | *Plectosphaerellaceae* | 0 | 0 | 0.001298 | Eukaryota |
| 296 | *Pseudocohnilembidae* | 0 | 0 | 0.001298 | Eukaryota |
| 297 | *Sordariaceae* | 0 | 0 | 0.001298 | Eukaryota |
| 298 | *Ustilaginaceae* | 0 | 0 | 0.001298 | Eukaryota |
| 299 | *Mimiviridae* | 0 | 0 | 0.001298 | Viruses |
| 300 | *Actinopolysporaceae* | 0 | 0.000895 | 0 | Bacteria |
| 301 | *Ardenticatenaceae* | 0 | 0.000895 | 0 | Bacteria |
| 302 | *Cellulomonadaceae* | 0 | 0.000895 | 0 | Bacteria |
| 303 | *Chlorogloeopsidaceae* | 0 | 0.000895 | 0 | Bacteria |
| 304 | *Coleofasciculaceae* | 0 | 0.000895 | 0 | Bacteria |
| 305 | *Dehalococcoidaceae* | 0 | 0.000895 | 0 | Bacteria |
| 306 | *Glycomycetaceae* | 0 | 0.000895 | 0 | Bacteria |
| 307 | *Lentimicrobiaceae* | 0 | 0.000895 | 0 | Bacteria |
| 308 | *Methylothermaceae* | 0 | 0.000895 | 0 | Bacteria |
| 309 | *Microcoleaceae* | 0 | 0.000895 | 0 | Bacteria |
| 310 | *Proteinivoraceae* | 0 | 0.000895 | 0 | Bacteria |
| 311 | *Rhodobiaceae* | 0 | 0.000895 | 0 | Bacteria |
| 312 | *Syntrophobacteraceae* | 0 | 0.000895 | 0 | Bacteria |
| 313 | *Thermodesulfobacteriaceae* | 0 | 0.000895 | 0 | Bacteria |
| 314 | *Thermoleophilaceae* | 0 | 0.000895 | 0 | Bacteria |
| 315 | *Methanosaetaceae* | 0 | 0.000895 | 0 | Archaea |
| 316 | *Natrialbaceae* | 0 | 0.000895 | 0 | Archaea |
| 317 | *Thermococcaceae* | 0 | 0.000895 | 0 | Archaea |
| 318 | *Ancylistaceae* | 0 | 0.000895 | 0 | Eukaryota |
| 319 | *Ophiocordycipitaceae* | 0 | 0.000895 | 0 | Eukaryota |
| 320 | *Pucciniaceae* | 0 | 0.000895 | 0 | Eukaryota |
| 321 | *Sclerotiniaceae* | 0 | 0.000895 | 0 | Eukaryota |
| 322 | *Sporidiobolaceae* | 0 | 0.000895 | 0 | Eukaryota |
| 323 | *Hytrosaviridae* | 0 | 0.000895 | 0 | Viruses |
| 324 | *Phycodnaviridae* | 0 | 0.000895 | 0 | Viruses |
| 325 | unclassified bacterial families | 0.214374 | 1.217448 | 0.340065 | Bacteria |
| 326 | unclassified archaeal families | 0.006551 | 0.005369 | 0.005192 | Archaea |
| 327 | unclassified eukaryotic families | 0.009827 | 0.009843 | 0.011682 | Eukaryota |
| 328 | unclassified viral families | 0 | 0.00179 | 0.001298 | Viruses |

| **Supplementary Table 4\| Minor genera with a relative abundance of less than 1%.** | | | | | |
| --- | --- | --- | --- | --- | --- |
| Sl. No. | Genera | Relative Abundance (%) | | | Domain |
|  |  | *Kinema* (India) | *Kinema* (Nepal) | *Kinema* (Bhutan) |  |
| 1 | *Enterococcus* | 0.819197 | 0.278588 | 1.293163 | Bacteria |
| 2 | *Staphylococcus* | 1.791896 | 0.212706 | 0.236792 | Bacteria |
| 3 | *Lysinibacillus* | 0.540388 | 0.249412 | 1.058669 | Bacteria |
| 4 | *Oceanobacillus* | 0.176997 | 0.217412 | 1.178215 | Bacteria |
| 5 | *Lactobacillus* | 0.29134 | 0.054588 | 0.99085 | Bacteria |
| 6 | *Ignatzschineria* | 0.858356 | 0.005647 | 0.354039 | Bacteria |
| 7 | *Myroides* | 0.031327 | 0.004706 | 1.114994 | Bacteria |
| 8 | *Paenibacillus* | 0.285074 | 0.316235 | 0.448296 | Bacteria |
| 9 | *Providencia* | 0.825462 | 0.005647 | 0.218401 | Bacteria |
| 10 | *Geobacillus* | 0.219288 | 0.581647 | 0.102304 | Bacteria |
| 11 | *Anoxybacillus* | 0.073618 | 0.677647 | 0.026438 | Bacteria |
| 12 | *Corynebacterium* | 0.247482 | 0.015059 | 0.514966 | Bacteria |
| 13 | *Kerstersia* | 0.009398 | 0.005647 | 0.760955 | Bacteria |
| 14 | *Clostridium* | 0.286641 | 0.229647 | 0.243689 | Bacteria |
| 15 | *Vagococcus* | 0.205191 | 0.006588 | 0.533358 | Bacteria |
| 16 | *Ureibacillus* | 0.028194 | 0.663529 | 0.019541 | Bacteria |
| 17 | *Thermoactinomyces* | 0.009398 | 0.679529 | 0.018392 | Bacteria |
| 18 | *Streptococcus* | 0.100246 | 0.122353 | 0.452894 | Bacteria |
| 19 | *Aneurinibacillus* | 0.062654 | 0.522353 | 0.078165 | Bacteria |
| 20 | *Virgibacillus* | 0.176997 | 0.200471 | 0.160927 | Bacteria |
| 21 | *Parageobacillus* | 0.032893 | 0.343529 | 0.025289 | Bacteria |
| 22 | *Paenalcaligenes* | 0.045424 | 0.000941 | 0.321854 | Bacteria |
| 23 | *Pediococcus* | 0.112777 | 0.096941 | 0.086211 | Bacteria |
| 24 | *Bordetella* | 0.009398 | 0.002824 | 0.273576 | Bacteria |
| 25 | *Escherichia* | 0.057955 | 0.008471 | 0.208056 | Bacteria |
| 26 | *Listeria* | 0.108078 | 0.053647 | 0.097706 | Bacteria |
| 27 | *Wohlfahrtiimonas* | 0.172298 | 0.002824 | 0.081613 | Bacteria |
| 28 | *Sporosarcina* | 0.08145 | 0.055529 | 0.114948 | Bacteria |
| 29 | *Caldibacillus* | 0.01723 | 0.176 | 0.020691 | Bacteria |
| 30 | *Mycobacterium* | 0.023495 | 0.025412 | 0.162076 | Bacteria |
| 31 | *Gracilibacillus* | 0.057955 | 0.112 | 0.040232 | Bacteria |
| 32 | *Oligella* | 0.187961 | 0.000941 | 0.003448 | Bacteria |
| 33 | *Carnobacterium* | 0.051689 | 0.048 | 0.086211 | Bacteria |
| 34 | *Weissella* | 0.08145 | 0.056471 | 0.006897 | Bacteria |
| 35 | *Fictibacillus* | 0.01723 | 0.049882 | 0.056324 | Bacteria |
| 36 | *Enterobacter* | 0.045424 | 0.002824 | 0.070118 | Bacteria |
| 37 | *Pseudomonas* | 0.031327 | 0.011294 | 0.067819 | Bacteria |
| 38 | *Aeribacillus* | 0.03446 | 0.060235 | 0.013794 | Bacteria |
| 39 | *Pneumocystis* | 0.076751 | 0.029176 | 0 | Eukaryota |
| 40 | *Anaerobacillus* | 0.04699 | 0.043294 | 0.013794 | Bacteria |
| 41 | *Amphibacillus* | 0.043858 | 0.030118 | 0.029886 | Bacteria |
| 42 | *Klebsiella* | 0.051689 | 0.007529 | 0.028737 | Bacteria |
| 43 | *Salmonella* | 0.015663 | 0.002824 | 0.067819 | Bacteria |
| 44 | *Ornithinibacillus* | 0.037592 | 0.026353 | 0.019541 | Bacteria |
| 45 | *Shigella* | 0.020362 | 0.002824 | 0.058623 | Bacteria |
| 46 | *Novibacillus* | 0.032893 | 0.044235 | 0.003448 | Bacteria |
| 47 | *Pontibacillus* | 0.045424 | 0.027294 | 0.005747 | Bacteria |
| 48 | *Jeotgalicoccus* | 0.073618 | 0.000941 | 0.003448 | Bacteria |
| 49 | *Rhodococcus* | 0.001566 | 0.003765 | 0.070118 | Bacteria |
| 50 | *Trichococcus* | 0.029761 | 0.024471 | 0.018392 | Bacteria |
| 51 | *Flavobacterium* | 0.007832 | 0.006588 | 0.057474 | Bacteria |
| 52 | *Cyclobacterium* | 0.036026 | 0.026353 | 0.008046 | Bacteria |
| 53 | *Lachnoclostridium* | 0.039159 | 0.016941 | 0.012644 | Bacteria |
| 54 | *Domibacillus* | 0.025061 | 0.022588 | 0.020691 | Bacteria |
| 55 | *Caenibacillus* | 0.012531 | 0.040471 | 0.010345 | Bacteria |
| 56 | *Alcaligenes* | 0.004699 | 0 | 0.055175 | Bacteria |
| 57 | *Pichia* | 0.007832 | 0.003765 | 0.045979 | Eukaryota |
| 58 | *Vibrio* | 0.018796 | 0.001882 | 0.031036 | Bacteria |
| 59 | *Halobacillus* | 0.021929 | 0.017882 | 0.011495 | Bacteria |
| 60 | *Exiguobacterium* | 0.010964 | 0.026353 | 0.011495 | Bacteria |
| 61 | *Globicatella* | 0.039159 | 0 | 0.008046 | Bacteria |
| 62 | *Viridibacillus* | 0.021929 | 0.006588 | 0.018392 | Bacteria |
| 63 | *Paucisalibacillus* | 0.009398 | 0.018824 | 0.017242 | Bacteria |
| 64 | *Rummeliibacillus* | 0.007832 | 0.009412 | 0.027587 | Bacteria |
| 65 | *Desulfuribacillus* | 0.012531 | 0.029176 | 0.002299 | Bacteria |
| 66 | *Terribacillus* | 0.01723 | 0.016941 | 0.009196 | Bacteria |
| 67 | *Aquibacillus* | 0.020362 | 0.013176 | 0.009196 | Bacteria |
| 68 | *Lentibacillus* | 0.015663 | 0.009412 | 0.017242 | Bacteria |
| 69 | *Planococcus* | 0.021929 | 0.003765 | 0.016093 | Bacteria |
| 70 | *Cohnella* | 0.014097 | 0.020706 | 0.005747 | Bacteria |
| 71 | *Nitrospira* | 0.01723 | 0.016941 | 0.005747 | Bacteria |
| 72 | *Pusillimonas* | 0.003133 | 0 | 0.036783 | Bacteria |
| 73 | *Burkholderia* | 0.015663 | 0.003765 | 0.019541 | Bacteria |
| 74 | *Paraliobacillus* | 0.018796 | 0.010353 | 0.008046 | Bacteria |
| 75 | *Sporolactobacillus* | 0.014097 | 0.010353 | 0.012644 | Bacteria |
| 76 | *Sphingobacterium* | 0.007832 | 0.001882 | 0.026438 | Bacteria |
| 77 | *Erysipelothrix* | 0.007832 | 0.000941 | 0.026438 | Bacteria |
| 78 | *Streptomyces* | 0.007832 | 0.015059 | 0.011495 | Bacteria |
| 79 | *Lactococcus* | 0.014097 | 0.005647 | 0.013794 | Bacteria |
| 80 | *Alicyclobacillus* | 0.012531 | 0.009412 | 0.011495 | Bacteria |
| 81 | *Xenorhabdus* | 0.012531 | 0 | 0.020691 | Bacteria |
| 82 | *Thauera* | 0.006265 | 0.010353 | 0.016093 | Bacteria |
| 83 | *Tuberibacillus* | 0.006265 | 0.015059 | 0.010345 | Bacteria |
| 84 | *Ruminiclostridium* | 0.004699 | 0.013176 | 0.013794 | Bacteria |
| 85 | *Blautia* | 0.006265 | 0.020706 | 0.004598 | Bacteria |
| 86 | *Jeotgalibacillus* | 0.014097 | 0.006588 | 0.010345 | Bacteria |
| 87 | *Solibacillus* | 0.018796 | 0.001882 | 0.010345 | Bacteria |
| 88 | *Beggiatoa* | 0.010964 | 0.014118 | 0.005747 | Bacteria |
| 89 | *Salinicoccus* | 0.014097 | 0.007529 | 0.009196 | Bacteria |
| 90 | *Caryophanon* | 0.018796 | 0.001882 | 0.009196 | Bacteria |
| 91 | *Clostridioides* | 0.004699 | 0.000941 | 0.024139 | Bacteria |
| 92 | *Psychrobacillus* | 0.007832 | 0.010353 | 0.011495 | Bacteria |
| 93 | *Achromobacter* | 0.004699 | 0.003765 | 0.020691 | Bacteria |
| 94 | *Mucor* | 0.007832 | 0.008471 | 0.012644 | Eukaryota |
| 95 | *Heliobacterium* | 0.004699 | 0.012235 | 0.011495 | Bacteria |
| 96 | *Bhargavaea* | 0.014097 | 0.001882 | 0.011495 | Bacteria |
| 97 | *Pantoea* | 0.009398 | 0.002824 | 0.014943 | Bacteria |
| 98 | *Eisenbergiella* | 0.012531 | 0.014118 | 0 | Bacteria |
| 99 | *Atopostipes* | 0.018796 | 0.006588 | 0.001149 | Bacteria |
| 100 | *Castellaniella* | 0.001566 | 0.000941 | 0.02299 | Bacteria |
| 101 | *Morganella* | 0.009398 | 0 | 0.016093 | Bacteria |
| 102 | *Serratia* | 0.014097 | 0.000941 | 0.010345 | Bacteria |
| 103 | *Paenisporosarcina* | 0.010964 | 0.003765 | 0.010345 | Bacteria |
| 104 | *Psychrobacter* | 0.010964 | 0 | 0.012644 | Bacteria |
| 105 | *Ochrobactrum* | 0 | 0 | 0.02299 | Bacteria |
| 106 | *Bacteroides* | 0.010964 | 0.009412 | 0.002299 | Bacteria |
| 107 | *Leuconostoc* | 0.010964 | 0.006588 | 0.004598 | Bacteria |
| 108 | *Thermoflavimicrobium* | 0.003133 | 0.013176 | 0.005747 | Bacteria |
| 109 | *Marinilactibacillus* | 0.007832 | 0.009412 | 0.004598 | Bacteria |
| 110 | *Yersinia* | 0.007832 | 0.001882 | 0.011495 | Bacteria |
| 111 | *Variovorax* | 0.007832 | 0.002824 | 0.010345 | Bacteria |
| 112 | *Oblitimonas* | 0.004699 | 0 | 0.016093 | Bacteria |
| 113 | *Tetragenococcus* | 0.009398 | 0.000941 | 0.010345 | Bacteria |
| 114 | *Moellerella* | 0.012531 | 0 | 0.008046 | Bacteria |
| 115 | *Sediminibacillus* | 0.003133 | 0.014118 | 0.002299 | Bacteria |
| 116 | *Acidovorax* | 0.003133 | 0.003765 | 0.012644 | Bacteria |
| 117 | *Ruminococcus* | 0.006265 | 0.002824 | 0.010345 | Bacteria |
| 118 | *Jeotgalibaca* | 0.003133 | 0.005647 | 0.010345 | Bacteria |
| 119 | *Hydrogenophaga* | 0.007832 | 0.006588 | 0.004598 | Bacteria |
| 120 | *Advenella* | 0.001566 | 0 | 0.017242 | Bacteria |
| 121 | *Oxobacter* | 0.012531 | 0.002824 | 0.003448 | Bacteria |
| 122 | *Macrococcus* | 0.007832 | 0.008471 | 0.002299 | Bacteria |
| 123 | *Candidimonas* | 0.004699 | 0 | 0.013794 | Bacteria |
| 124 | *Paraburkholderia* | 0.006265 | 0.002824 | 0.009196 | Bacteria |
| 125 | *Chryseobacterium* | 0.004699 | 0.006588 | 0.006897 | Bacteria |
| 126 | *Halomonas* | 0.007832 | 0.001882 | 0.008046 | Bacteria |
| 127 | *Thalassobacillus* | 0.004699 | 0.003765 | 0.009196 | Bacteria |
| 128 | *Dechloromonas* | 0.009398 | 0.004706 | 0.003448 | Bacteria |
| 129 | *Neisseria* | 0.006265 | 0.000941 | 0.010345 | Bacteria |
| 130 | *Alkalibacterium* | 0.009398 | 0.000941 | 0.006897 | Bacteria |
| 131 | *Natribacillus* | 0.003133 | 0.006588 | 0.006897 | Bacteria |
| 132 | *Gemmatimonas* | 0.010964 | 0.005647 | 0 | Bacteria |
| 133 | *Pectobacterium* | 0.006265 | 0.000941 | 0.009196 | Bacteria |
| 134 | *Selenomonas* | 0.003133 | 0.008471 | 0.004598 | Bacteria |
| 135 | *Cosenzaea* | 0.007832 | 0 | 0.008046 | Bacteria |
| 136 | *Tepidibacillus* | 0.007832 | 0.005647 | 0.002299 | Bacteria |
| 137 | *Polaromonas* | 0.001566 | 0.009412 | 0.004598 | Bacteria |
| 138 | *Peptoniphilus* | 0.009398 | 0 | 0.005747 | Bacteria |
| 139 | *Parvimonas* | 0.001566 | 0.006588 | 0.006897 | Bacteria |
| 140 | *Oligella* | 0 | 0 | 0.014943 | Bacteria |
| 141 | *Planomicrobium* | 0.009398 | 0.000941 | 0.004598 | Bacteria |
| 142 | *Numidum* | 0.004699 | 0.006588 | 0.003448 | Bacteria |
| 143 | *Phascolarctobacterium* | 0 | 0.000941 | 0.013794 | Bacteria |
| 144 | *Anaeromassilibacillus* | 0.010964 | 0.003765 | 0 | Bacteria |
| 145 | *Rubrivivax* | 0.003133 | 0.005647 | 0.005747 | Bacteria |
| 146 | *Caldalkalibacillus* | 0.001566 | 0.009412 | 0.003448 | Bacteria |
| 147 | *Aeromonas* | 0.004699 | 0.002824 | 0.006897 | Bacteria |
| 148 | *Paracoccus* | 0.007832 | 0.001882 | 0.004598 | Bacteria |
| 149 | *Arthrobacter* | 0.004699 | 0.003765 | 0.005747 | Bacteria |
| 150 | *Carboxydocella* | 0.004699 | 0.003765 | 0.005747 | Bacteria |
| 151 | *Atopococcus* | 0.009398 | 0.004706 | 0 | Bacteria |
| 152 | *Rhodoferax* | 0.003133 | 0.002824 | 0.008046 | Bacteria |
| 153 | *Photobacterium* | 0.009398 | 0 | 0.004598 | Bacteria |
| 154 | *Curvibacter* | 0.003133 | 0.008471 | 0.002299 | Bacteria |
| 155 | *Legionella* | 0.006265 | 0.001882 | 0.005747 | Bacteria |
| 156 | *Pelistega* | 0.004699 | 0 | 0.009196 | Bacteria |
| 157 | *Azospirillum* | 0.004699 | 0.005647 | 0.003448 | Bacteria |
| 158 | *Prevotella* | 0.006265 | 0.002824 | 0.004598 | Bacteria |
| 159 | *Chlamydia* | 0.003133 | 0.004706 | 0.005747 | Bacteria |
| 160 | *Eubacterium* | 0.004699 | 0.001882 | 0.006897 | Bacteria |
| 161 | *Halolactibacillus* | 0.004699 | 0.001882 | 0.006897 | Bacteria |
| 162 | *Rhizobium* | 0.004699 | 0.001882 | 0.006897 | Bacteria |
| 163 | *Defluviitalea* | 0.004699 | 0.007529 | 0.001149 | Bacteria |
| 164 | *Desulfitobacterium* | 0.001566 | 0.009412 | 0.002299 | Bacteria |
| 165 | *Gilliamella* | 0.006265 | 0 | 0.006897 | Bacteria |
| 166 | *Anaerostipes* | 0.006265 | 0.005647 | 0.001149 | Bacteria |
| 167 | *Massilia* | 0.004699 | 0.003765 | 0.004598 | Bacteria |
| 168 | *Alteribacillus* | 0.004699 | 0.004706 | 0.003448 | Bacteria |
| 169 | *Sphingomonas* | 0.003133 | 0.003765 | 0.005747 | Bacteria |
| 170 | *Bradyrhizobium* | 0.001566 | 0.007529 | 0.003448 | Bacteria |
| 171 | *Enteractinococcus* | 0.012531 | 0 | 0 | Bacteria |
| 172 | *Gorillibacterium* | 0.004699 | 0.001882 | 0.005747 | Bacteria |
| 173 | *Moraxella* | 0.003133 | 0 | 0.009196 | Bacteria |
| 174 | *Taylorella* | 0.003133 | 0 | 0.009196 | Bacteria |
| 175 | *Desulfotomaculum* | 0.003133 | 0.005647 | 0.003448 | Bacteria |
| 176 | *Salimicrobium* | 0.004699 | 0.002824 | 0.004598 | Bacteria |
| 177 | *Tissierella* | 0.003133 | 0.001882 | 0.006897 | Bacteria |
| 178 | *Brackiella* | 0.001566 | 0 | 0.010345 | Bacteria |
| 179 | *Acetobacter* | 0.006265 | 0.001882 | 0.003448 | Bacteria |
| 180 | *Mesorhizobium* | 0.006265 | 0.001882 | 0.003448 | Bacteria |
| 181 | *Microbacterium* | 0.004699 | 0 | 0.006897 | Bacteria |
| 182 | *Geomicrobium* | 0.001566 | 0.001882 | 0.008046 | Bacteria |
| 183 | *Paeniclostridium* | 0.006265 | 0.002824 | 0.002299 | Bacteria |
| 184 | *Alkaliphilus* | 0.003133 | 0.004706 | 0.003448 | Bacteria |
| 185 | *Nitrosomonas* | 0.003133 | 0.004706 | 0.003448 | Bacteria |
| 186 | *Acetobacterium* | 0.001566 | 0.002824 | 0.006897 | Bacteria |
| 187 | *Chitinophaga* | 0.001566 | 0.002824 | 0.006897 | Bacteria |
| 188 | *Acidaminococcus* | 0.009398 | 0.001882 | 0 | Bacteria |
| 189 | *Bifidobacterium* | 0.006265 | 0.003765 | 0.001149 | Bacteria |
| 190 | *Dickeya* | 0.007832 | 0.000941 | 0.002299 | Bacteria |
| 191 | *Candida* | 0.010964 | 0 | 0 | Eukaryota |
| 192 | *Geobacter* | 0.004699 | 0.003765 | 0.002299 | Bacteria |
| 193 | *Comamonas* | 0.003133 | 0.001882 | 0.005747 | Bacteria |
| 194 | *Prosthecobacter* | 0.007832 | 0.002824 | 0 | Bacteria |
| 195 | *Brachybacterium* | 0.009398 | 0 | 0.001149 | Bacteria |
| 196 | *Haemophilus* | 0.004699 | 0 | 0.005747 | Bacteria |
| 197 | *Vitreoscilla* | 0.004699 | 0 | 0.005747 | Bacteria |
| 198 | *Aerococcus* | 0.004699 | 0.000941 | 0.004598 | Bacteria |
| 199 | *Rhizobacter* | 0.004699 | 0.000941 | 0.004598 | Bacteria |
| 200 | *Varibaculum* | 0.004699 | 0.000941 | 0.004598 | Bacteria |
| 201 | *Marinococcus* | 0.003133 | 0.004706 | 0.002299 | Bacteria |
| 202 | *Gottschalkia* | 0 | 0.000941 | 0.009196 | Bacteria |
| 203 | *Drancourtella* | 0.006265 | 0.003765 | 0 | Bacteria |
| 204 | *Cupriavidus* | 0.004699 | 0.001882 | 0.003448 | Bacteria |
| 205 | *Cronobacter* | 0.004699 | 0.002824 | 0.002299 | Bacteria |
| 206 | *Edaphobacillus* | 0.004699 | 0.002824 | 0.002299 | Bacteria |
| 207 | *Methylibium* | 0.001566 | 0.004706 | 0.003448 | Bacteria |
| 208 | *Marinobacter* | 0.003133 | 0.001882 | 0.004598 | Bacteria |
| 209 | *Xanthomonas* | 0.001566 | 0 | 0.008046 | Bacteria |
| 210 | *Brevibacterium* | 0.006265 | 0.000941 | 0.002299 | Bacteria |
| 211 | *Algoriphagus* | 0.003133 | 0.002824 | 0.003448 | Bacteria |
| 212 | *Methyloversatilis* | 0.003133 | 0.002824 | 0.003448 | Bacteria |
| 213 | *Marinomonas* | 0.004699 | 0 | 0.004598 | Bacteria |
| 214 | *Anaerosalibacter* | 0.004699 | 0.000941 | 0.003448 | Bacteria |
| 215 | *Lonsdalea* | 0.001566 | 0.002824 | 0.004598 | Bacteria |
| 216 | *Butyrivibrio* | 0.004699 | 0.001882 | 0.002299 | Bacteria |
| 217 | *Rheinheimera* | 0.004699 | 0.001882 | 0.002299 | Bacteria |
| 218 | *Rhodobacter* | 0.004699 | 0.001882 | 0.002299 | Bacteria |
| 219 | *Garciella* | 0.003133 | 0 | 0.005747 | Bacteria |
| 220 | *Shewanella* | 0.003133 | 0 | 0.005747 | Bacteria |
| 221 | *Dysgonomonas* | 0.004699 | 0.002824 | 0.001149 | Bacteria |
| 222 | *Oceanospirillum* | 0.003133 | 0.000941 | 0.004598 | Bacteria |
| 223 | *Aquabacterium* | 0.001566 | 0.004706 | 0.002299 | Bacteria |
| 224 | *Capnocytophaga* | 0 | 0.002824 | 0.005747 | Bacteria |
| 225 | *Pseudoalteromonas* | 0.003133 | 0.001882 | 0.003448 | Bacteria |
| 226 | *Janthinobacterium* | 0.001566 | 0 | 0.006897 | Bacteria |
| 227 | *Ilumatobacter* | 0.003133 | 0.002824 | 0.002299 | Bacteria |
| 228 | *Peptostreptococcus* | 0.003133 | 0.002824 | 0.002299 | Bacteria |
| 229 | *Kluyvera* | 0.001566 | 0.000941 | 0.005747 | Bacteria |
| 230 | *Pseudonocardia* | 0.001566 | 0.000941 | 0.005747 | Bacteria |
| 231 | *Tenuibacillus* | 0.004699 | 0 | 0.003448 | Bacteria |
| 232 | *Longilinea* | 0.003133 | 0.003765 | 0.001149 | Bacteria |
| 233 | *Alcanivorax* | 0 | 0 | 0.008046 | Bacteria |
| 234 | *Microbulbifer* | 0.004699 | 0.000941 | 0.002299 | Bacteria |
| 235 | *Paludibacter* | 0.001566 | 0.002824 | 0.003448 | Bacteria |
| 236 | *Treponema* | 0.001566 | 0.002824 | 0.003448 | Bacteria |
| 237 | *Zoogloea* | 0.001566 | 0.002824 | 0.003448 | Bacteria |
| 238 | *Leclercia* | 0 | 0.000941 | 0.006897 | Bacteria |
| 239 | *Anaerorhabdus* | 0.007832 | 0 | 0 | Bacteria |
| 240 | *Magnetospirillum* | 0.004699 | 0.001882 | 0.001149 | Bacteria |
| 241 | *Salibacterium* | 0.004699 | 0.001882 | 0.001149 | Bacteria |
| 242 | *Lambdavirus* | 0.004699 | 0.001882 | 0.001149 | Virus |
| 243 | *Facklamia* | 0.003133 | 0 | 0.004598 | Bacteria |
| 244 | *Pelomonas* | 0.004699 | 0.002824 | 0 | Bacteria |
| 245 | *Dyadobacter* | 0.003133 | 0.000941 | 0.003448 | Bacteria |
| 246 | *Erwinia* | 0.003133 | 0.000941 | 0.003448 | Bacteria |
| 247 | *Lutispora* | 0.003133 | 0.000941 | 0.003448 | Bacteria |
| 248 | *Desulfospira* | 0 | 0.002824 | 0.004598 | Bacteria |
| 249 | *Maledivibacter* | 0.006265 | 0 | 0.001149 | Bacteria |
| 250 | *Novosphingobium* | 0.003133 | 0.001882 | 0.002299 | Bacteria |
| 251 | *Rhodopirellula* | 0.003133 | 0.001882 | 0.002299 | Bacteria |
| 252 | *Edwardsiella* | 0.001566 | 0 | 0.005747 | Bacteria |
| 253 | *Sporomusa* | 0.001566 | 0.005647 | 0 | Bacteria |
| 254 | *Bartonella* | 0.001566 | 0.000941 | 0.004598 | Bacteria |
| 255 | *Pedobacter* | 0.001566 | 0.000941 | 0.004598 | Bacteria |
| 256 | *Caldicoprobacter* | 0 | 0.004706 | 0.002299 | Bacteria |
| 257 | *Idiomarina* | 0.004699 | 0 | 0.002299 | Bacteria |
| 258 | *Marinospirillum* | 0.004699 | 0 | 0.002299 | Bacteria |
| 259 | *Photorhabdus* | 0.004699 | 0 | 0.002299 | Bacteria |
| 260 | *Frankia* | 0.001566 | 0.001882 | 0.003448 | Bacteria |
| 261 | *Herbaspirillum* | 0.001566 | 0.001882 | 0.003448 | Bacteria |
| 262 | *Pseudoxanthomonas* | 0.001566 | 0.001882 | 0.003448 | Bacteria |
| 263 | *Stenotrophomonas* | 0.001566 | 0.001882 | 0.003448 | Bacteria |
| 264 | *Sinorhizobium* | 0.004699 | 0.000941 | 0.001149 | Bacteria |
| 265 | *Citrobacter* | 0.001566 | 0.002824 | 0.002299 | Bacteria |
| 266 | *Lihuaxuella* | 0.001566 | 0.002824 | 0.002299 | Bacteria |
| 267 | *Acholeplasma* | 0 | 0.000941 | 0.005747 | Bacteria |
| 268 | *Rubeoparvulum* | 0 | 0.000941 | 0.005747 | Bacteria |
| 269 | *Cellulosilyticum* | 0.004699 | 0.001882 | 0 | Bacteria |
| 270 | *Veillonella* | 0.004699 | 0.001882 | 0 | Bacteria |
| 271 | *Anaerocolumna* | 0.003133 | 0 | 0.003448 | Bacteria |
| 272 | *Azoarcus* | 0.003133 | 0 | 0.003448 | Bacteria |
| 273 | *Orrella* | 0.003133 | 0 | 0.003448 | Bacteria |
| 274 | *Marinobacterium* | 0 | 0.001882 | 0.004598 | Bacteria |
| 275 | *Amycolatopsis* | 0.003133 | 0.000941 | 0.002299 | Bacteria |
| 276 | *Deinococcus* | 0.003133 | 0.000941 | 0.002299 | Bacteria |
| 277 | *Desulfovibrio* | 0.003133 | 0.000941 | 0.002299 | Bacteria |
| 278 | *Porphyrobacter* | 0.003133 | 0.000941 | 0.002299 | Bacteria |
| 279 | *Tatumella* | 0.003133 | 0.000941 | 0.002299 | Bacteria |
| 280 | *Thermicanus* | 0.001566 | 0.004706 | 0 | Bacteria |
| 281 | *Agatevirus* | 0.001566 | 0.004706 | 0 | Virus |
| 282 | *Effusibacillus* | 0 | 0.002824 | 0.003448 | Bacteria |
| 283 | *Yaniella* | 0.006265 | 0 | 0 | Bacteria |
| 284 | *Collinsella* | 0.003133 | 0.001882 | 0.001149 | Bacteria |
| 285 | *Agrobacterium* | 0.001566 | 0 | 0.004598 | Bacteria |
| 286 | *Isobaculum* | 0.001566 | 0 | 0.004598 | Bacteria |
| 287 | *Leucobacter* | 0.001566 | 0 | 0.004598 | Bacteria |
| 288 | *Pirellula* | 0 | 0.003765 | 0.002299 | Bacteria |
| 289 | *Pelobacter* | 0.003133 | 0.002824 | 0 | Bacteria |
| 290 | *Trichosporon* | 0.003133 | 0.002824 | 0 | Eukaryota |
| 291 | *Akkermansia* | 0.001566 | 0.000941 | 0.003448 | Bacteria |
| 292 | *Sporanaerobacter* | 0.001566 | 0.000941 | 0.003448 | Bacteria |
| 293 | *Thiobacillus* | 0.001566 | 0.000941 | 0.003448 | Bacteria |
| 294 | *Hyphomicrobium* | 0.004699 | 0 | 0.001149 | Bacteria |
| 295 | *Methyloceanibacter* | 0.004699 | 0 | 0.001149 | Bacteria |
| 296 | *Azonexus* | 0.001566 | 0.001882 | 0.002299 | Bacteria |
| 297 | *Duganella* | 0.001566 | 0.001882 | 0.002299 | Bacteria |
| 298 | *Paucibacter* | 0.001566 | 0.001882 | 0.002299 | Bacteria |
| 299 | *Piscibacillus* | 0.001566 | 0.001882 | 0.002299 | Bacteria |
| 300 | *Paenisporosarcina* | 0 | 0 | 0.005747 | Bacteria |
| 301 | *Allofustis* | 0 | 0.005647 | 0 | Bacteria |
| 302 | *Arcobacter* | 0.004699 | 0.000941 | 0 | Bacteria |
| 303 | *Phenylobacterium* | 0.004699 | 0.000941 | 0 | Bacteria |
| 304 | *Pseudobacteroides* | 0.004699 | 0.000941 | 0 | Bacteria |
| 305 | *Altererythrobacter* | 0 | 0.000941 | 0.004598 | Bacteria |
| 306 | *Dehalobacter* | 0 | 0.000941 | 0.004598 | Bacteria |
| 307 | *Bdellovibrio* | 0.003133 | 0 | 0.002299 | Bacteria |
| 308 | *Campylobacter* | 0.003133 | 0 | 0.002299 | Bacteria |
| 309 | *Faecalibacterium* | 0.003133 | 0 | 0.002299 | Bacteria |
| 310 | *Lachnoanaerobaculum* | 0.003133 | 0 | 0.002299 | Bacteria |
| 311 | *Pseudoflavonifractor* | 0.001566 | 0.003765 | 0 | Bacteria |
| 312 | *Polynucleobacter* | 0 | 0.001882 | 0.003448 | Bacteria |
| 313 | *Cephaloticoccus* | 0.003133 | 0.000941 | 0.001149 | Bacteria |
| 314 | *Devosia* | 0.003133 | 0.000941 | 0.001149 | Bacteria |
| 315 | *Helicobacter* | 0.003133 | 0.000941 | 0.001149 | Bacteria |
| 316 | *Pedosphaera* | 0.003133 | 0.000941 | 0.001149 | Bacteria |
| 317 | *Shimazuella* | 0.003133 | 0.000941 | 0.001149 | Bacteria |
| 318 | *Methanosarcina* | 0.003133 | 0.000941 | 0.001149 | Archaea |
| 319 | *Chthoniobacter* | 0.003133 | 0.001882 | 0 | Bacteria |
| 320 | *Salinivibrio* | 0.001566 | 0 | 0.003448 | Bacteria |
| 321 | *Francisella* | 0 | 0.003765 | 0.001149 | Bacteria |
| 322 | *Pontibacter* | 0 | 0.003765 | 0.001149 | Bacteria |
| 323 | *Propionispora* | 0 | 0.003765 | 0.001149 | Bacteria |
| 324 | *Steroidobacter* | 0 | 0.003765 | 0.001149 | Bacteria |
| 325 | *Alkalibacillus* | 0.001566 | 0.000941 | 0.002299 | Bacteria |
| 326 | *Desulfobulbus* | 0.001566 | 0.000941 | 0.002299 | Bacteria |
| 327 | *Fusobacterium* | 0.001566 | 0.000941 | 0.002299 | Bacteria |
| 328 | *Hymenobacter* | 0.001566 | 0.000941 | 0.002299 | Bacteria |
| 329 | *Loktanella* | 0.001566 | 0.000941 | 0.002299 | Bacteria |
| 330 | *Mariniphaga* | 0.001566 | 0.000941 | 0.002299 | Bacteria |
| 331 | *Massilibacterium* | 0.001566 | 0.000941 | 0.002299 | Bacteria |
| 332 | *Nitrosococcus* | 0.001566 | 0.000941 | 0.002299 | Bacteria |
| 333 | *Rhodovulum* | 0.001566 | 0.000941 | 0.002299 | Bacteria |
| 334 | *Paraclostridium* | 0.004699 | 0 | 0 | Bacteria |
| 335 | *Geotrichum* | 0.004699 | 0 | 0 | Eukaryota |
| 336 | *P68virus* | 0.004699 | 0 | 0 | Virus |
| 337 | *Alteromonas* | 0.001566 | 0.001882 | 0.001149 | Bacteria |
| 338 | *Dendrosporobacter* | 0.001566 | 0.001882 | 0.001149 | Bacteria |
| 339 | *Fuerstia* | 0.001566 | 0.001882 | 0.001149 | Bacteria |
| 340 | *Hyphomonas* | 0.001566 | 0.001882 | 0.001149 | Bacteria |
| 341 | *Oleiphilus* | 0.001566 | 0.001882 | 0.001149 | Bacteria |
| 342 | *Halothiobacillus* | 0 | 0 | 0.004598 | Bacteria |
| 343 | *Kinetoplastibacterium* | 0 | 0 | 0.004598 | Bacteria |
| 344 | *Acaryochloris* | 0.001566 | 0.002824 | 0 | Bacteria |
| 345 | *Desulfuromonas* | 0.001566 | 0.002824 | 0 | Bacteria |
| 346 | *Geothrix* | 0.001566 | 0.002824 | 0 | Bacteria |
| 347 | *Sunxiuqinia* | 0.001566 | 0.002824 | 0 | Bacteria |
| 348 | *Verrucomicrobium* | 0.001566 | 0.002824 | 0 | Bacteria |
| 349 | *Azohydromonas* | 0 | 0.000941 | 0.003448 | Bacteria |
| 350 | *Brucella* | 0 | 0.000941 | 0.003448 | Bacteria |
| 351 | *Cloacibacterium* | 0 | 0.000941 | 0.003448 | Bacteria |
| 352 | *Empedobacter* | 0 | 0.000941 | 0.003448 | Bacteria |
| 353 | *Paraglaciecola* | 0 | 0.000941 | 0.003448 | Bacteria |
| 354 | *Roseimaritima* | 0 | 0.000941 | 0.003448 | Bacteria |
| 355 | *Sphingobium* | 0 | 0.000941 | 0.003448 | Bacteria |
| 356 | *Thermobacillus* | 0 | 0.000941 | 0.003448 | Bacteria |
| 357 | *Thioalkalivibrio* | 0 | 0.000941 | 0.003448 | Bacteria |
| 358 | *Alkanindiges* | 0.003133 | 0 | 0.001149 | Bacteria |
| 359 | *Arenimonas* | 0.003133 | 0 | 0.001149 | Bacteria |
| 360 | *Christensenella* | 0.003133 | 0 | 0.001149 | Bacteria |
| 361 | *Desemzia* | 0.003133 | 0 | 0.001149 | Bacteria |
| 362 | *Fabibacter* | 0.003133 | 0 | 0.001149 | Bacteria |
| 363 | *Gallibacterium* | 0.003133 | 0 | 0.001149 | Bacteria |
| 364 | *Lacticigenium* | 0.003133 | 0 | 0.001149 | Bacteria |
| 365 | *Lysobacter* | 0.003133 | 0 | 0.001149 | Bacteria |
| 366 | *Melissococcus* | 0.003133 | 0 | 0.001149 | Bacteria |
| 367 | *Methylobacterium* | 0.003133 | 0 | 0.001149 | Bacteria |
| 368 | *Rhodanobacter* | 0.003133 | 0 | 0.001149 | Bacteria |
| 369 | *Snodgrassella* | 0.003133 | 0 | 0.001149 | Bacteria |
| 370 | *Succinatimonas* | 0.003133 | 0 | 0.001149 | Bacteria |
| 371 | *Thiomicrospira* | 0.003133 | 0 | 0.001149 | Bacteria |
| 372 | *Thioploca* | 0 | 0.001882 | 0.002299 | Bacteria |
| 373 | *Blastopirellula* | 0.003133 | 0.000941 | 0 | Bacteria |
| 374 | *Desulfobacter* | 0.003133 | 0.000941 | 0 | Bacteria |
| 375 | *Dokdonella* | 0.003133 | 0.000941 | 0 | Bacteria |
| 376 | *Ktedonobacter* | 0.003133 | 0.000941 | 0 | Bacteria |
| 377 | *Methylomicrobium* | 0.003133 | 0.000941 | 0 | Bacteria |
| 378 | *Anaerococcus* | 0 | 0.002824 | 0.001149 | Bacteria |
| 379 | *Haloferula* | 0 | 0.002824 | 0.001149 | Bacteria |
| 380 | *Syntrophomonas* | 0 | 0.002824 | 0.001149 | Bacteria |
| 381 | *Acidiphilium* | 0.001566 | 0 | 0.002299 | Bacteria |
| 382 | *Aquamicrobium* | 0.001566 | 0 | 0.002299 | Bacteria |
| 383 | *Aquaspirillum* | 0.001566 | 0 | 0.002299 | Bacteria |
| 384 | *Balneatrix* | 0.001566 | 0 | 0.002299 | Bacteria |
| 385 | *Gordonia* | 0.001566 | 0 | 0.002299 | Bacteria |
| 386 | *Granulicatella* | 0.001566 | 0 | 0.002299 | Bacteria |
| 387 | *Holophaga* | 0.001566 | 0 | 0.002299 | Bacteria |
| 388 | *Hungatella* | 0.001566 | 0 | 0.002299 | Bacteria |
| 389 | *Inquilinus* | 0.001566 | 0 | 0.002299 | Bacteria |
| 390 | *Lacunisphaera* | 0.001566 | 0 | 0.002299 | Bacteria |
| 391 | *Leptospira* | 0.001566 | 0 | 0.002299 | Bacteria |
| 392 | *Limnohabitans* | 0.001566 | 0 | 0.002299 | Bacteria |
| 393 | *Mycoplasma* | 0.001566 | 0 | 0.002299 | Bacteria |
| 394 | *Parapedobacter* | 0.001566 | 0 | 0.002299 | Bacteria |
| 395 | *Pasteurella* | 0.001566 | 0 | 0.002299 | Bacteria |
| 396 | *Skermanella* | 0.001566 | 0 | 0.002299 | Bacteria |
| 397 | *Thiomonas* | 0.001566 | 0 | 0.002299 | Bacteria |
| 398 | *Tolumonas* | 0.001566 | 0 | 0.002299 | Bacteria |
| 399 | *Epulopiscium* | 0 | 0.003765 | 0 | Bacteria |
| 400 | *Kocuria* | 0 | 0.003765 | 0 | Bacteria |
| 401 | *Aliicoccus* | 0.001566 | 0.000941 | 0.001149 | Bacteria |
| 402 | *Cetobacterium* | 0.001566 | 0.000941 | 0.001149 | Bacteria |
| 403 | *Chryseolinea* | 0.001566 | 0.000941 | 0.001149 | Bacteria |
| 404 | *Coprobacillus* | 0.001566 | 0.000941 | 0.001149 | Bacteria |
| 405 | *Niastella* | 0.001566 | 0.000941 | 0.001149 | Bacteria |
| 406 | *Opitutus* | 0.001566 | 0.000941 | 0.001149 | Bacteria |
| 407 | *Rugosibacter* | 0.001566 | 0.000941 | 0.001149 | Bacteria |
| 408 | *Seinonella* | 0.001566 | 0.000941 | 0.001149 | Bacteria |
| 409 | *Sulfuricella* | 0.001566 | 0.000941 | 0.001149 | Bacteria |
| 410 | *Acidibacillus* | 0.001566 | 0.001882 | 0 | Bacteria |
| 411 | *Brevundimonas* | 0.001566 | 0.001882 | 0 | Bacteria |
| 412 | *Dietzia* | 0.001566 | 0.001882 | 0 | Bacteria |
| 413 | *Dolosigranulum* | 0.001566 | 0.001882 | 0 | Bacteria |
| 414 | *Erythrobacter* | 0.001566 | 0.001882 | 0 | Bacteria |
| 415 | *Kaistia* | 0.001566 | 0.001882 | 0 | Bacteria |
| 416 | *Kyrpidia* | 0.001566 | 0.001882 | 0 | Bacteria |
| 417 | *Nocardia* | 0.001566 | 0.001882 | 0 | Bacteria |
| 418 | *Pandoraea* | 0.001566 | 0.001882 | 0 | Bacteria |
| 419 | *Paraprevotella* | 0.001566 | 0.001882 | 0 | Bacteria |
| 420 | *Perlucidibaca* | 0.001566 | 0.001882 | 0 | Bacteria |
| 421 | *Sulfuritalea* | 0.001566 | 0.001882 | 0 | Bacteria |
| 422 | *Thiorhodovibrio* | 0.001566 | 0.001882 | 0 | Bacteria |
| 423 | *Batrachochytrium* | 0.001566 | 0.001882 | 0 | Eukaryota |
| 424 | *Lichtheimia* | 0.001566 | 0.001882 | 0 | Eukaryota |
| 425 | *Adhaeribacter* | 0 | 0 | 0.003448 | Bacteria |
| 426 | *Arsenophonus* | 0 | 0 | 0.003448 | Bacteria |
| 427 | *Beijerinckia* | 0 | 0 | 0.003448 | Bacteria |
| 428 | *Chlorobium* | 0 | 0 | 0.003448 | Bacteria |
| 429 | *Curtobacterium* | 0 | 0 | 0.003448 | Bacteria |
| 430 | *Desulfonatronum* | 0 | 0 | 0.003448 | Bacteria |
| 431 | *Roseomonas* | 0 | 0 | 0.003448 | Bacteria |
| 432 | *Rubellimicrobium* | 0 | 0 | 0.003448 | Bacteria |
| 433 | *Sodalis* | 0 | 0 | 0.003448 | Bacteria |
| 434 | *Tessaracoccus* | 0 | 0 | 0.003448 | Bacteria |
| 435 | *Aquitalea* | 0 | 0.000941 | 0.002299 | Bacteria |
| 436 | *Arachidicoccus* | 0 | 0.000941 | 0.002299 | Bacteria |
| 437 | *Aurantimonas* | 0 | 0.000941 | 0.002299 | Bacteria |
| 438 | *Azotobacter* | 0 | 0.000941 | 0.002299 | Bacteria |
| 439 | *Collimonas* | 0 | 0.000941 | 0.002299 | Bacteria |
| 440 | *Hathewaya* | 0 | 0.000941 | 0.002299 | Bacteria |
| 441 | *Mucilaginibacter* | 0 | 0.000941 | 0.002299 | Bacteria |
| 442 | *Pelagirhabdus* | 0 | 0.000941 | 0.002299 | Bacteria |
| 443 | *Propionibacterium* | 0 | 0.000941 | 0.002299 | Bacteria |
| 444 | *Salegentibacter* | 0 | 0.000941 | 0.002299 | Bacteria |
| 445 | *Zavarzinella* | 0 | 0.000941 | 0.002299 | Bacteria |
| 446 | *Thalassiosira* | 0 | 0.000941 | 0.002299 | Eukaryota |
| 447 | *Anaeromyxobacter* | 0.003133 | 0 | 0 | Bacteria |
| 448 | *Chishuiella* | 0.003133 | 0 | 0 | Bacteria |
| 449 | *Desulfurella* | 0.003133 | 0 | 0 | Bacteria |
| 450 | *Endozoicomonas* | 0.003133 | 0 | 0 | Bacteria |
| 451 | *Gulbenkiania* | 0.003133 | 0 | 0 | Bacteria |
| 452 | *Kushneria* | 0.003133 | 0 | 0 | Bacteria |
| 453 | *Marinilabilia* | 0.003133 | 0 | 0 | Bacteria |
| 454 | *Methylocystis* | 0.003133 | 0 | 0 | Bacteria |
| 455 | *Nafulsella* | 0.003133 | 0 | 0 | Bacteria |
| 456 | *Nosocomiicoccus* | 0.003133 | 0 | 0 | Bacteria |
| 457 | *Pseudorhodobacter* | 0.003133 | 0 | 0 | Bacteria |
| 458 | *Salinibacillus* | 0.003133 | 0 | 0 | Bacteria |
| 459 | *Sutterella* | 0.003133 | 0 | 0 | Bacteria |
| 460 | *Tepidimonas* | 0.003133 | 0 | 0 | Bacteria |
| 461 | *Thermoflexibacter* | 0.003133 | 0 | 0 | Bacteria |
| 462 | *Meyerozyma* | 0.003133 | 0 | 0 | Eukaryota |
| 463 | *Stylonychia* | 0.003133 | 0 | 0 | Eukaryota |
| 464 | *Sextaecvirus* | 0.003133 | 0 | 0 | Virus |
| 465 | *Cellvibrio* | 0 | 0.001882 | 0.001149 | Bacteria |
| 466 | *Nostoc* | 0 | 0.001882 | 0.001149 | Bacteria |
| 467 | *Planctomyces* | 0 | 0.001882 | 0.001149 | Bacteria |
| 468 | *Runella* | 0 | 0.001882 | 0.001149 | Bacteria |
| 469 | *Thermincola* | 0 | 0.001882 | 0.001149 | Bacteria |
| 470 | *Aquincola* | 0 | 0.002824 | 0 | Bacteria |
| 471 | *Caulobacter* | 0 | 0.002824 | 0 | Bacteria |
| 472 | *Hydrocarboniphaga* | 0 | 0.002824 | 0 | Bacteria |
| 473 | *Kroppenstedtia* | 0 | 0.002824 | 0 | Bacteria |
| 474 | *Leptolyngbya* | 0 | 0.002824 | 0 | Bacteria |
| 475 | *Microcystis* | 0 | 0.002824 | 0 | Bacteria |
| 476 | *Methanocaldococcus* | 0 | 0.002824 | 0 | Archaea |
| 477 | *Achromatium* | 0.001566 | 0 | 0.001149 | Bacteria |
| 478 | *Actinomyces* | 0.001566 | 0 | 0.001149 | Bacteria |
| 479 | *Alistipes* | 0.001566 | 0 | 0.001149 | Bacteria |
| 480 | *Anaerolinea* | 0.001566 | 0 | 0.001149 | Bacteria |
| 481 | *Aureispira* | 0.001566 | 0 | 0.001149 | Bacteria |
| 482 | *Azovibrio* | 0.001566 | 0 | 0.001149 | Bacteria |
| 483 | *Bibersteinia* | 0.001566 | 0 | 0.001149 | Bacteria |
| 484 | *Cardiobacterium* | 0.001566 | 0 | 0.001149 | Bacteria |
| 485 | *Coxiella* | 0.001566 | 0 | 0.001149 | Bacteria |
| 486 | *Dorea* | 0.001566 | 0 | 0.001149 | Bacteria |
| 487 | *Enterovibrio* | 0.001566 | 0 | 0.001149 | Bacteria |
| 488 | *Ferrimonas* | 0.001566 | 0 | 0.001149 | Bacteria |
| 489 | *Ferrovum* | 0.001566 | 0 | 0.001149 | Bacteria |
| 490 | *Gemella* | 0.001566 | 0 | 0.001149 | Bacteria |
| 491 | *Hafnia* | 0.001566 | 0 | 0.001149 | Bacteria |
| 492 | *Hahella* | 0.001566 | 0 | 0.001149 | Bacteria |
| 493 | *Hoeflea* | 0.001566 | 0 | 0.001149 | Bacteria |
| 494 | *Marinagarivorans* | 0.001566 | 0 | 0.001149 | Bacteria |
| 495 | *Microvirgula* | 0.001566 | 0 | 0.001149 | Bacteria |
| 496 | *Nocardioides* | 0.001566 | 0 | 0.001149 | Bacteria |
| 497 | *Oceanimonas* | 0.001566 | 0 | 0.001149 | Bacteria |
| 498 | *Olsenella* | 0.001566 | 0 | 0.001149 | Bacteria |
| 499 | *Oribacterium* | 0.001566 | 0 | 0.001149 | Bacteria |
| 500 | *Ottowia* | 0.001566 | 0 | 0.001149 | Bacteria |
| 501 | *Oxalobacter* | 0.001566 | 0 | 0.001149 | Bacteria |
| 502 | *Paeniglutamicibacter* | 0.001566 | 0 | 0.001149 | Bacteria |
| 503 | *Parabacteroides* | 0.001566 | 0 | 0.001149 | Bacteria |
| 504 | *Phocea* | 0.001566 | 0 | 0.001149 | Bacteria |
| 505 | *Plesiomonas* | 0.001566 | 0 | 0.001149 | Bacteria |
| 506 | *Polaribacter* | 0.001566 | 0 | 0.001149 | Bacteria |
| 507 | *Proteiniborus* | 0.001566 | 0 | 0.001149 | Bacteria |
| 508 | *Pseudobacteriovorax* | 0.001566 | 0 | 0.001149 | Bacteria |
| 509 | *Renibacterium* | 0.001566 | 0 | 0.001149 | Bacteria |
| 510 | *Roseobacter* | 0.001566 | 0 | 0.001149 | Bacteria |
| 511 | *Roseovarius* | 0.001566 | 0 | 0.001149 | Bacteria |
| 512 | *Saccharibacillus* | 0.001566 | 0 | 0.001149 | Bacteria |
| 513 | *Teredinibacter* | 0.001566 | 0 | 0.001149 | Bacteria |
| 514 | *Thalassospira* | 0.001566 | 0 | 0.001149 | Bacteria |
| 515 | *Thioalkalimicrobium* | 0.001566 | 0 | 0.001149 | Bacteria |
| 516 | *Thiomargarita* | 0.001566 | 0 | 0.001149 | Bacteria |
| 517 | *Thorsellia* | 0.001566 | 0 | 0.001149 | Bacteria |
| 518 | *Xylophilus* | 0.001566 | 0 | 0.001149 | Bacteria |
| 519 | *Acanthamoeba* | 0.001566 | 0 | 0.001149 | Eukaryota |
| 520 | *Aspergillus* | 0.001566 | 0 | 0.001149 | Eukaryota |
| 521 | *Kuraishia* | 0.001566 | 0 | 0.001149 | Eukaryota |
| 522 | *Oxytricha* | 0.001566 | 0 | 0.001149 | Eukaryota |
| 523 | *Acidimicrobium* | 0.001566 | 0.000941 | 0 | Bacteria |
| 524 | *Beduini* | 0.001566 | 0.000941 | 0 | Bacteria |
| 525 | *Caedibacter* | 0.001566 | 0.000941 | 0 | Bacteria |
| 526 | *Chloroflexus* | 0.001566 | 0.000941 | 0 | Bacteria |
| 527 | *Cloacibacillus* | 0.001566 | 0.000941 | 0 | Bacteria |
| 528 | *Flavihumibacter* | 0.001566 | 0.000941 | 0 | Bacteria |
| 529 | *Fusicatenibacter* | 0.001566 | 0.000941 | 0 | Bacteria |
| 530 | *Glaciecola* | 0.001566 | 0.000941 | 0 | Bacteria |
| 531 | *Halobacteroides* | 0.001566 | 0.000941 | 0 | Bacteria |
| 532 | *Merdibacter* | 0.001566 | 0.000941 | 0 | Bacteria |
| 533 | *Methylocaldum* | 0.001566 | 0.000941 | 0 | Bacteria |
| 534 | *Methylotenera* | 0.001566 | 0.000941 | 0 | Bacteria |
| 535 | *Nocardiopsis* | 0.001566 | 0.000941 | 0 | Bacteria |
| 536 | *Oceanibaculum* | 0.001566 | 0.000941 | 0 | Bacteria |
| 537 | *Proteiniclasticum* | 0.001566 | 0.000941 | 0 | Bacteria |
| 538 | *Raoultella* | 0.001566 | 0.000941 | 0 | Bacteria |
| 539 | *Roseateles* | 0.001566 | 0.000941 | 0 | Bacteria |
| 540 | *Rubinisphaera* | 0.001566 | 0.000941 | 0 | Bacteria |
| 541 | *Sphaerotilus* | 0.001566 | 0.000941 | 0 | Bacteria |
| 542 | *Spirosoma* | 0.001566 | 0.000941 | 0 | Bacteria |
| 543 | *Methanocella* | 0.001566 | 0.000941 | 0 | Archaea |
| 544 | *Nitrososphaera* | 0.001566 | 0.000941 | 0 | Archaea |
| 545 | *Ichthyophthirius* | 0.001566 | 0.000941 | 0 | Eukaryota |
| 546 | *Actinobacillus* | 0 | 0 | 0.002299 | Bacteria |
| 547 | *Aggregatibacter* | 0 | 0 | 0.002299 | Bacteria |
| 548 | *Agromyces* | 0 | 0 | 0.002299 | Bacteria |
| 549 | *Atopococcus* | 0 | 0 | 0.002299 | Bacteria |
| 550 | *Basilea* | 0 | 0 | 0.002299 | Bacteria |
| 551 | *Brochothrix* | 0 | 0 | 0.002299 | Bacteria |
| 552 | *Buttiauxella* | 0 | 0 | 0.002299 | Bacteria |
| 553 | *Calditerricola* | 0 | 0 | 0.002299 | Bacteria |
| 554 | *Chromobacterium* | 0 | 0 | 0.002299 | Bacteria |
| 555 | *Commensalibacter* | 0 | 0 | 0.002299 | Bacteria |
| 556 | *Dethiobacter* | 0 | 0 | 0.002299 | Bacteria |
| 557 | *Ectothiorhodospira* | 0 | 0 | 0.002299 | Bacteria |
| 558 | *Elizabethkingia* | 0 | 0 | 0.002299 | Bacteria |
| 559 | *Emticicia* | 0 | 0 | 0.002299 | Bacteria |
| 560 | *Halonatronum* | 0 | 0 | 0.002299 | Bacteria |
| 561 | *Helcococcus* | 0 | 0 | 0.002299 | Bacteria |
| 562 | *Herminiimonas* | 0 | 0 | 0.002299 | Bacteria |
| 563 | *Immundisolibacter* | 0 | 0 | 0.002299 | Bacteria |
| 564 | *Intestinibacter* | 0 | 0 | 0.002299 | Bacteria |
| 565 | *Lacinutrix* | 0 | 0 | 0.002299 | Bacteria |
| 566 | *Laribacter* | 0 | 0 | 0.002299 | Bacteria |
| 567 | *Leucothrix* | 0 | 0 | 0.002299 | Bacteria |
| 568 | *Mageeibacillus* | 0 | 0 | 0.002299 | Bacteria |
| 569 | *Megasphaera* | 0 | 0 | 0.002299 | Bacteria |
| 570 | *Microvirga* | 0 | 0 | 0.002299 | Bacteria |
| 571 | *Neglecta* | 0 | 0 | 0.002299 | Bacteria |
| 572 | *Nitrincola* | 0 | 0 | 0.002299 | Bacteria |
| 573 | *Nitrosospira* | 0 | 0 | 0.002299 | Bacteria |
| 574 | *Oceaniovalibus* | 0 | 0 | 0.002299 | Bacteria |
| 575 | *Pelosinus* | 0 | 0 | 0.002299 | Bacteria |
| 576 | *Phaseolibacter* | 0 | 0 | 0.002299 | Bacteria |
| 577 | *Pilibacter* | 0 | 0 | 0.002299 | Bacteria |
| 578 | *Pluralibacter* | 0 | 0 | 0.002299 | Bacteria |
| 579 | *Pseudospirillum* | 0 | 0 | 0.002299 | Bacteria |
| 580 | *Salinicola* | 0 | 0 | 0.002299 | Bacteria |
| 581 | *Salinimicrobium* | 0 | 0 | 0.002299 | Bacteria |
| 582 | *Salsuginibacillus* | 0 | 0 | 0.002299 | Bacteria |
| 583 | *Sediminispirochaeta* | 0 | 0 | 0.002299 | Bacteria |
| 584 | *Sphingopyxis* | 0 | 0 | 0.002299 | Bacteria |
| 585 | *Spirochaeta* | 0 | 0 | 0.002299 | Bacteria |
| 586 | *Sulfurifustis* | 0 | 0 | 0.002299 | Bacteria |
| 587 | *Tenacibaculum* | 0 | 0 | 0.002299 | Bacteria |
| 588 | *Tepidibacter* | 0 | 0 | 0.002299 | Bacteria |
| 589 | *Thermoanaerobacterium* | 0 | 0 | 0.002299 | Bacteria |
| 590 | *Thermotalea* | 0 | 0 | 0.002299 | Bacteria |
| 591 | *Neospora* | 0 | 0 | 0.002299 | Eukaryota |
| 592 | *Bellilinea* | 0 | 0.000941 | 0.001149 | Bacteria |
| 593 | *Belnapia* | 0 | 0.000941 | 0.001149 | Bacteria |
| 594 | *Bergeriella* | 0 | 0.000941 | 0.001149 | Bacteria |
| 595 | *Bosea* | 0 | 0.000941 | 0.001149 | Bacteria |
| 596 | *Caballeronia* | 0 | 0.000941 | 0.001149 | Bacteria |
| 597 | *Caldithrix* | 0 | 0.000941 | 0.001149 | Bacteria |
| 598 | *Catalinimonas* | 0 | 0.000941 | 0.001149 | Bacteria |
| 599 | *Corallococcus* | 0 | 0.000941 | 0.001149 | Bacteria |
| 600 | *Crocinitomix* | 0 | 0.000941 | 0.001149 | Bacteria |
| 601 | *Desulfitibacter* | 0 | 0.000941 | 0.001149 | Bacteria |
| 602 | *Eremococcus* | 0 | 0.000941 | 0.001149 | Bacteria |
| 603 | *Flammeovirga* | 0 | 0.000941 | 0.001149 | Bacteria |
| 604 | *Flavisolibacter* | 0 | 0.000941 | 0.001149 | Bacteria |
| 605 | *Fluviicola* | 0 | 0.000941 | 0.001149 | Bacteria |
| 606 | *Fodinicurvata* | 0 | 0.000941 | 0.001149 | Bacteria |
| 607 | *Giesbergeria* | 0 | 0.000941 | 0.001149 | Bacteria |
| 608 | *Hippea* | 0 | 0.000941 | 0.001149 | Bacteria |
| 609 | *Hyalangium* | 0 | 0.000941 | 0.001149 | Bacteria |
| 610 | *Hylemonella* | 0 | 0.000941 | 0.001149 | Bacteria |
| 611 | *Intrasporangium* | 0 | 0.000941 | 0.001149 | Bacteria |
| 612 | *Leeuwenhoekiella* | 0 | 0.000941 | 0.001149 | Bacteria |
| 613 | *Methylomonas* | 0 | 0.000941 | 0.001149 | Bacteria |
| 614 | *Methylovorus* | 0 | 0.000941 | 0.001149 | Bacteria |
| 615 | *Micromonospora* | 0 | 0.000941 | 0.001149 | Bacteria |
| 616 | *Mogibacterium* | 0 | 0.000941 | 0.001149 | Bacteria |
| 617 | *Moorella* | 0 | 0.000941 | 0.001149 | Bacteria |
| 618 | *Neptunomonas* | 0 | 0.000941 | 0.001149 | Bacteria |
| 619 | *Nevskia* | 0 | 0.000941 | 0.001149 | Bacteria |
| 620 | *Novispirillum* | 0 | 0.000941 | 0.001149 | Bacteria |
| 621 | *Phormidesmis* | 0 | 0.000941 | 0.001149 | Bacteria |
| 622 | *Ralstonia* | 0 | 0.000941 | 0.001149 | Bacteria |
| 623 | *Rhodospirillum* | 0 | 0.000941 | 0.001149 | Bacteria |
| 624 | *Rhodovibrio* | 0 | 0.000941 | 0.001149 | Bacteria |
| 625 | *Salipaludibacillus* | 0 | 0.000941 | 0.001149 | Bacteria |
| 626 | *Saprospira* | 0 | 0.000941 | 0.001149 | Bacteria |
| 627 | *Sedimentibacter* | 0 | 0.000941 | 0.001149 | Bacteria |
| 628 | *Solimonas* | 0 | 0.000941 | 0.001149 | Bacteria |
| 629 | *Tepidanaerobacter* | 0 | 0.000941 | 0.001149 | Bacteria |
| 630 | *Turicibacter* | 0 | 0.000941 | 0.001149 | Bacteria |
| 631 | *Vaginella* | 0 | 0.000941 | 0.001149 | Bacteria |
| 632 | *Arenibacter* | 0 | 0.001882 | 0 | Bacteria |
| 633 | *Borreliella* | 0 | 0.001882 | 0 | Bacteria |
| 634 | *Bryobacter* | 0 | 0.001882 | 0 | Bacteria |
| 635 | *Caldanaerobacter* | 0 | 0.001882 | 0 | Bacteria |
| 636 | *Chthonomonas* | 0 | 0.001882 | 0 | Bacteria |
| 637 | *Geitlerinema* | 0 | 0.001882 | 0 | Bacteria |
| 638 | *Haliea* | 0 | 0.001882 | 0 | Bacteria |
| 639 | *Ideonella* | 0 | 0.001882 | 0 | Bacteria |
| 640 | *Lewinella* | 0 | 0.001882 | 0 | Bacteria |
| 641 | *Micrococcus* | 0 | 0.001882 | 0 | Bacteria |
| 642 | *Oscillibacter* | 0 | 0.001882 | 0 | Bacteria |
| 643 | *Pelagibacterium* | 0 | 0.001882 | 0 | Bacteria |
| 644 | *Phycicoccus* | 0 | 0.001882 | 0 | Bacteria |
| 645 | *Planctomicrobium* | 0 | 0.001882 | 0 | Bacteria |
| 646 | *Roseivirga* | 0 | 0.001882 | 0 | Bacteria |
| 647 | *Rubrobacter* | 0 | 0.001882 | 0 | Bacteria |
| 648 | *Sandaracinus* | 0 | 0.001882 | 0 | Bacteria |
| 649 | *Tepidimicrobium* | 0 | 0.001882 | 0 | Bacteria |
| 650 | *Verminephrobacter* | 0 | 0.001882 | 0 | Bacteria |
| 651 | *Penicillium* | 0 | 0.001882 | 0 | Eukaryota |
| 652 | *Methanolinea* | 0.001566 | 0 | 0 | Archaea |
| 653 | *Acetabularia* | 0.001566 | 0 | 0 | Eukaryota |
| 654 | *Acidomyces* | 0.001566 | 0 | 0 | Eukaryota |
| 655 | *Albugo* | 0.001566 | 0 | 0 | Eukaryota |
| 656 | *Chlorella* | 0.001566 | 0 | 0 | Eukaryota |
| 657 | *Cyberlindnera* | 0.001566 | 0 | 0 | Eukaryota |
| 658 | *Debaryomyces* | 0.001566 | 0 | 0 | Eukaryota |
| 659 | *Exophiala* | 0.001566 | 0 | 0 | Eukaryota |
| 660 | *Fragilariopsis* | 0.001566 | 0 | 0 | Eukaryota |
| 661 | *Galdieria* | 0.001566 | 0 | 0 | Eukaryota |
| 662 | *Hypsizygus* | 0.001566 | 0 | 0 | Eukaryota |
| 663 | *Leucosporidium* | 0.001566 | 0 | 0 | Eukaryota |
| 664 | *Metschnikowia* | 0.001566 | 0 | 0 | Eukaryota |
| 665 | *Nannochloropsis* | 0.001566 | 0 | 0 | Eukaryota |
| 666 | *Plasmodiophora* | 0.001566 | 0 | 0 | Eukaryota |
| 667 | *Pyronema* | 0.001566 | 0 | 0 | Eukaryota |
| 668 | *Saprolegnia* | 0.001566 | 0 | 0 | Eukaryota |
| 669 | *Spathaspora* | 0.001566 | 0 | 0 | Eukaryota |
| 670 | *Stentor* | 0.001566 | 0 | 0 | Eukaryota |
| 671 | *Tetrahymena* | 0.001566 | 0 | 0 | Eukaryota |
| 672 | *Bc431virus* | 0.001566 | 0 | 0 | Virus |
| 673 | *Cba181virus* | 0.001566 | 0 | 0 | Virus |
| 674 | *Kayvirus* | 0.001566 | 0 | 0 | Virus |
| 675 | *Sep1virus* | 0.001566 | 0 | 0 | Virus |
| 676 | *Silviavirus* | 0.001566 | 0 | 0 | Virus |
| 677 | *Haloprofundus* | 0 | 0 | 0.001149 | Archaea |
| 678 | *Methanoregula* | 0 | 0 | 0.001149 | Archaea |
| 679 | *Thermoproteus* | 0 | 0 | 0.001149 | Archaea |
| 680 | *Asterionella* | 0 | 0 | 0.001149 | Eukaryota |
| 681 | *Calocera* | 0 | 0 | 0.001149 | Eukaryota |
| 682 | *Ectocarpus* | 0 | 0 | 0.001149 | Eukaryota |
| 683 | *Eimeria* | 0 | 0 | 0.001149 | Eukaryota |
| 684 | *Emiliania* | 0 | 0 | 0.001149 | Eukaryota |
| 685 | *Ostreobium* | 0 | 0 | 0.001149 | Eukaryota |
| 686 | *Parasitella* | 0 | 0 | 0.001149 | Eukaryota |
| 687 | *Pseudocohnilembus* | 0 | 0 | 0.001149 | Eukaryota |
| 688 | *Trypanosoma* | 0 | 0 | 0.001149 | Eukaryota |
| 689 | *Verticillium* | 0 | 0 | 0.001149 | Eukaryota |
| 690 | *Yarrowia* | 0 | 0 | 0.001149 | Eukaryota |
| 691 | *Andromedavirus* | 0 | 0 | 0.001149 | Virus |
| 692 | *Slashvirus* | 0 | 0 | 0.001149 | Virus |
| 693 | *Haloterrigena* | 0 | 0.000941 | 0 | Archaea |
| 694 | *Methanosaeta* | 0 | 0.000941 | 0 | Archaea |
| 695 | *Methanosalsum* | 0 | 0.000941 | 0 | Archaea |
| 696 | *Pyrococcus* | 0 | 0.000941 | 0 | Archaea |
| 697 | *Conidiobolus* | 0 | 0.000941 | 0 | Eukaryota |
| 698 | *Hirsutella* | 0 | 0.000941 | 0 | Eukaryota |
| 699 | *Puccinia* | 0 | 0.000941 | 0 | Eukaryota |
| 700 | *Rhodotorula* | 0 | 0.000941 | 0 | Eukaryota |
| 701 | *Sclerotinia* | 0 | 0.000941 | 0 | Eukaryota |
| 702 | *Trypanozoon* | 0 | 0.000941 | 0 | Eukaryota |
| 703 | *Muscavirus* | 0 | 0.000941 | 0 | Virus |
| 704 | unclassified bacterial genera | 0.25688 | 0.197647 | 0.212653 | Bacteria |
| 705 | unclassified archaeal genera | 0 | 0 | 0.002299 | Archaea |
| 706 | unclassified eukaryotic genera | 0.007832 | 0.005647 | 0.012644 | Eukaryota |
| 707 | unclassified viral genera | 0.722084 | 0.112941 | 0.011495 | Virus |
