# Supplementary material for: Metagenomic-Metabolomic Mining of Kinema, a Naturally Fermented Soybean Food of the Eastern Himalayas
Source: Front Microbiol. 2022 Apr 29;13:868383. doi: 10.3389/fmicb.2022.868383 (PMC9106393; doi:10.3389/fmicb.2022.868383)
Supplement: Supplementary file 3 [file Table_3.DOCX]

| **Supplementary Table 5\| Minor species with a relative abundance of less than 1%.** | | | | | |
| --- | --- | --- | --- | --- | --- |
| **Sl. No.** | **Species** | **Relative Abundance (%)** | | | **Domain** |
|  |  | ***Kinema***  **(India)** | ***Kinema***  **(Nepal)** | ***Kinema***  **(Bhutan)** |  |
| 1 | *Bacillus andreraoultii* | 1.00877 | 1.331558 | 0.285517 | Bacteria |
| 2 | *Ureibacillus thermosphaericus* | 0.074114 | 2.346871 | 0.040448 | Bacteria |
| 3 | *Bacillus* phage Grass | 0.156462 | 2.045939 | 0 | Viruses |
| 4 | *Providencia stuartii* | 1.708733 | 0.016644 | 0.285517 | Bacteria |
| 5 | *Proteus mirabilis* | 0.535266 | 0.013316 | 1.215827 | Bacteria |
| 6 | *Kerstersia gyiorum* | 0.024705 | 0.019973 | 1.575103 | Bacteria |
| 7 | *Bacillus* phage PM1 | 1.021122 | 0.199734 | 0 | Viruses |
| 8 | *Bacillus thuringiensis* | 0.069996 | 0.05992 | 1.082586 | Bacteria |
| 9 | *Oceanobacillus oncorhynchi* | 0.024705 | 0.046605 | 1.089724 | Bacteria |
| 10 | *Bacillus eiseniae* | 0.12764 | 0.319574 | 0.701896 | Bacteria |
| 11 | *Bacillus stratosphericus* | 0.275868 | 0.466045 | 0.406862 | Bacteria |
| 12 | *Ignatzschineria larvae* | 0.739881 | 0.004437 | 0.341627 | Bacteria |
| 13 | *Bacillus* sp. MSP13 | 0.345864 | 0.69241 | 0.023793 | Bacteria |
| 14 | *Streptococcus pneumoniae* | 0.078231 | 0.109854 | 0.763758 | Bacteria |
| 15 | *Enterococcus faecalis* | 0.247046 | 0.36285 | 0.306931 | Bacteria |
| 16 | *Geobacillus* sp. 8 | 0.275868 | 0.569241 | 0.03331 | Bacteria |
| 17 | *Corynebacterium stationis* | 0.424095 | 0.036618 | 0.399724 | Bacteria |
| 18 | *Bacillus sonorensis* | 0.395273 | 0.329561 | 0.099931 | Bacteria |
| 19 | *Paenalcaligenes hominis* | 0.119405 | 0.003329 | 0.666207 | Bacteria |
| 20 | *Bacillus* phage SIOphi | 0.782312 | 0.009987 | 0 | Viruses |
| 21 | *Bacillus alveayuensis* | 0.037057 | 0.699068 | 0.03331 | Bacteria |
| 22 | *Staphylococcus hominis* | 0.27175 | 0.39281 | 0.095172 | Bacteria |
| 23 | *Enterococcus faecium* | 0.317042 | 0.066578 | 0.349759 | Bacteria |
| 24 | *Pediococcus acidilactici* | 0.275868 | 0.312916 | 0.140379 | Bacteria |
| 25 | *Caldibacillus debilis* | 0.045292 | 0.622503 | 0.042828 | Bacteria |
| 26 | *Acinetobacter baumannii* | 0.242928 | 0.036618 | 0.411621 | Bacteria |
| 27 | *Bacillus pumilus* | 0.107053 | 0.139814 | 0.430655 | Bacteria |
| 28 | *Vagococcus fluvialis* | 0.275868 | 0.003329 | 0.397345 | Bacteria |
| 29 | *Staphylococcus aureus* | 0.382921 | 0.146471 | 0.135621 | Bacteria |
| 30 | *Bacillus smithii* | 0.115288 | 0.476032 | 0.064241 | Bacteria |
| 31 | *Vagococcus lutrae* | 0.123523 | 0.006658 | 0.523448 | Bacteria |
| 32 | *Parageobacillus thermoglucosidans* | 0.065879 | 0.522636 | 0.014276 | Bacteria |
| 33 | *Bacillus anthracis* | 0.024705 | 0.019973 | 0.523448 | Bacteria |
| 34 | *Anoxybacillus tepidamans* | 0.008235 | 0.532623 | 0.011897 | Bacteria |
| 35 | *Bacillus velezensis* | 0.111171 | 0.286285 | 0.149897 | Bacteria |
| 36 | *Escherichia coli* | 0.14411 | 0.026631 | 0.373552 | Bacteria |
| 37 | *Bacillus sporothermodurans* | 0.279985 | 0.216378 | 0.021414 | Bacteria |
| 38 | *Staphylococcus sciuri* | 0.485857 | 0.006658 | 0.021414 | Bacteria |
| 39 | *Lysinibacillus sphaericus* | 0.251163 | 0.103196 | 0.159414 | Bacteria |
| 40 | *Oceanobacillus sojae* | 0.045292 | 0.109854 | 0.340241 | Bacteria |
| 41 | *Bordetella trematum* | 0.004117 | 0.003329 | 0.466345 | Bacteria |
| 42 | *Listeria monocytogenes* | 0.197637 | 0.133156 | 0.130862 | Bacteria |
| 43 | *Gracilibacillus boraciitolerans* | 0.115288 | 0.316245 | 0.026172 | Bacteria |
| 44 | *Bacillus fordii* | 0.16058 | 0.229694 | 0.047586 | Bacteria |
| 45 | *Oligella ureolytica* | 0.424095 | 0.003329 | 0.007138 | Bacteria |
| 46 | *Geobacillus* sp. WCH70 | 0.082349 | 0.329561 | 0.007138 | Bacteria |
| 47 | *Mycobacterium abscessus* | 0.045292 | 0.073236 | 0.276 | Bacteria |
| 48 | *Anoxybacillus amylolyticus* | 0.004117 | 0.379494 | 0.004759 | Bacteria |
| 49 | *Bacillus nealsonii* | 0.028822 | 0.02996 | 0.321207 | Bacteria |
| 50 | *Bacillus clausii* | 0.16058 | 0.073236 | 0.145138 | Bacteria |
| 51 | *Enterococcus cecorum* | 0.053527 | 0.079893 | 0.202241 | Bacteria |
| 52 | *Aeribacillus pallidus* | 0.090583 | 0.213049 | 0.028552 | Bacteria |
| 53 | *Carnobacterium maltaromaticum* | 0.069996 | 0.139814 | 0.121345 | Bacteria |
| 54 | *Oceanobacillus caeni* | 0.107053 | 0.206391 | 0.009517 | Bacteria |
| 55 | *Bacillus* sp. BT1B_CT2 | 0.197637 | 0.096538 | 0.026172 | Bacteria |
| 56 | *Acinetobacter* sp. WCHA30 | 0.189402 | 0.006658 | 0.118965 | Bacteria |
| 57 | *Bacillus cytotoxicus* | 0.218224 | 0.056591 | 0.03569 | Bacteria |
| 58 | *Pneumocystis jirovecii* | 0.201754 | 0.103196 | 0 | Eukaryota |
| 59 | *Kurthia huakuii* | 0.205871 | 0.046605 | 0.047586 | Bacteria |
| 60 | *Bacillus nakamurai* | 0.098818 | 0.176431 | 0.023793 | Bacteria |
| 61 | *Anoxybacillus* sp*.* UARK*-*01 | 0.049409 | 0.236352 | 0.007138 | Bacteria |
| 62 | *Staphylococcus gallinarum* | 0.284103 | 0.006658 | 0 | Bacteria |
| 63 | *Lactobacillus fermentum* | 0.028822 | 0.006658 | 0.254586 | Bacteria |
| 64 | *Bacillus* sp. LM 4-2 | 0.028822 | 0.166445 | 0.090414 | Bacteria |
| 65 | *Bacillus azotoformans* | 0.123523 | 0.123169 | 0.038069 | Bacteria |
| 66 | *Bacillus mycoides* | 0.020587 | 0.009987 | 0.252207 | Bacteria |
| 67 | *Bacillus tequilensis* | 0.074114 | 0.079893 | 0.121345 | Bacteria |
| 68 | *Lysinibacillus xylanilyticus* | 0.061761 | 0.019973 | 0.192724 | Bacteria |
| 69 | *Lactobacillus salivarius* | 0.247046 | 0.016644 | 0.009517 | Bacteria |
| 70 | *Clostridium colicanis* | 0.004117 | 0.259654 | 0.004759 | Bacteria |
| 71 | *Virgibacillus halodenitrificans* | 0.094701 | 0.153129 | 0.016655 | Bacteria |
| 72 | *Wohlfahrtiimonas chitiniclastica* | 0.185284 | 0.006658 | 0.059483 | Bacteria |
| 73 | *Bacillus methanolicus* | 0.045292 | 0.136485 | 0.069 | Bacteria |
| 74 | *Novibacillus thermophilus* | 0.086466 | 0.156458 | 0.007138 | Bacteria |
| 75 | *Bacillus* sp. VT-16-64 | 0.078231 | 0.11984 | 0.049966 | Bacteria |
| 76 | *Geobacillus stearothermophilus* | 0.049409 | 0.173103 | 0.023793 | Bacteria |
| 77 | *Enterococcus asini* | 0.069996 | 0.006658 | 0.168931 | Bacteria |
| 78 | *Oceanobacillus timonensis* | 0.028822 | 0.076565 | 0.138 | Bacteria |
| 79 | *Anaerobacillus* sp. NB2006 | 0.115288 | 0.103196 | 0.014276 | Bacteria |
| 80 | *Clostridium argentinense* | 0.168815 | 0.009987 | 0.052345 | Bacteria |
| 81 | *Paenibacillus* sp. P22 | 0.045292 | 0.066578 | 0.109448 | Bacteria |
| 82 | *Oceanobacillus jeddahense* | 0.032939 | 0.026631 | 0.159414 | Bacteria |
| 83 | *Bacillus acidiproducens* | 0.024705 | 0.109854 | 0.083276 | Bacteria |
| 84 | *Bacillus* sp. NRRL B-41327 | 0.172932 | 0.039947 | 0.002379 | Bacteria |
| 85 | *Bacillus* sp. EGD-AK10 | 0.041174 | 0.083222 | 0.090414 | Bacteria |
| 86 | *Virgibacillus proomii* | 0.049409 | 0.11984 | 0.045207 | Bacteria |
| 87 | *Virgibacillus pantothenticus* | 0.049409 | 0.136485 | 0.028552 | Bacteria |
| 88 | *Weissella paramesenteroides* | 0.148227 | 0.056591 | 0.007138 | Bacteria |
| 89 | *Cyclobacterium qasimii* | 0.107053 | 0.086551 | 0.016655 | Bacteria |
| 90 | *Myroides odoratimimus* | 0.012352 | 0 | 0.192724 | Bacteria |
| 91 | *Lysinibacillus macroides* | 0.053527 | 0.079893 | 0.071379 | Bacteria |
| 92 | *Bacillus pseudofirmus* | 0.028822 | 0.159787 | 0.009517 | Bacteria |
| 93 | *Caenibacillus caldisaponilyticus* | 0.032939 | 0.143142 | 0.021414 | Bacteria |
| 94 | *Parageobacillus toebii* | 0 | 0.186418 | 0.009517 | Bacteria |
| 95 | *Brevibacillus panacihumi* | 0.012352 | 0.046605 | 0.135621 | Bacteria |
| 96 | *Salmonella enterica* | 0.041174 | 0.009987 | 0.140379 | Bacteria |
| 97 | *Streptococcus anginosus* | 0.020587 | 0.146471 | 0.014276 | Bacteria |
| 98 | *Lysinibacillus* sp. FJAT-14222 | 0.053527 | 0.009987 | 0.111828 | Bacteria |
| 99 | *Bacillus* sp. Marseille-P2366 | 0.057644 | 0.086551 | 0.030931 | Bacteria |
| 100 | *Fictibacillus phosphorivorans* | 0.024705 | 0.109854 | 0.03331 | Bacteria |
| 101 | *Bacillus massiliosenegalensis* | 0.028822 | 0.109854 | 0.028552 | Bacteria |
| 102 | *Bacillus farraginis* | 0.024705 | 0.08988 | 0.052345 | Bacteria |
| 103 | *Bacillus niacini* | 0.057644 | 0.096538 | 0.011897 | Bacteria |
| 104 | *Bacillus acidicola* | 0.037057 | 0.113182 | 0.014276 | Bacteria |
| 105 | *Acinetobacter* sp. NIPH 2171 | 0.020587 | 0.003329 | 0.140379 | Bacteria |
| 106 | *Bacillus* sp. EB01 | 0.053527 | 0.103196 | 0.007138 | Bacteria |
| 107 | *Lysinibacillus odysseyi* | 0.069996 | 0.069907 | 0.023793 | Bacteria |
| 108 | *Enterococcus casseliflavus* | 0.045292 | 0.013316 | 0.10469 | Bacteria |
| 109 | *Oceanobacillus damuensis* | 0.049409 | 0.073236 | 0.040448 | Bacteria |
| 110 | *Acinetobacter* sp. YZS-X1-1 | 0.024705 | 0.003329 | 0.133241 | Bacteria |
| 111 | *Parageobacillus* genomosp. 1 | 0 | 0.159787 | 0 | Bacteria |
| 112 | *Acinetobacter* sp. NIPH 899 | 0.024705 | 0.003329 | 0.130862 | Bacteria |
| 113 | *Kurthia* sp. Dielmo | 0.111171 | 0.019973 | 0.026172 | Bacteria |
| 114 | *Providencia rettgeri* | 0.098818 | 0 | 0.057103 | Bacteria |
| 115 | *Bacillus* sp. Marseille-P2384 | 0.020587 | 0.099867 | 0.03331 | Bacteria |
| 116 | *Bacillus solani* | 0.037057 | 0.099867 | 0.016655 | Bacteria |
| 117 | *Bacillus* sp. X1(2014) | 0.024705 | 0.116511 | 0.011897 | Bacteria |
| 118 | *Lysinibacillus sp. FJAT-14745* | 0.049409 | 0.003329 | 0.097552 | Bacteria |
| 119 | *Lysinibacillus sp. AC-3* | 0.041174 | 0.006658 | 0.099931 | Bacteria |
| 120 | *Bacillus* sp. FMQ74 | 0.053527 | 0.039947 | 0.052345 | Bacteria |
| 121 | *Rhodococcus rhodochrous* | 0 | 0.009987 | 0.135621 | Bacteria |
| 122 | *Amphibacillus sediminis* | 0.057644 | 0.079893 | 0.007138 | Bacteria |
| 123 | *Parageobacillus thermantarcticus* | 0.012352 | 0.129827 | 0.002379 | Bacteria |
| 124 | *Aneurinibacillus aneurinilyticus* | 0.074114 | 0.013316 | 0.057103 | Bacteria |
| 125 | *Klebsiella pneumoniae* | 0.086466 | 0.019973 | 0.038069 | Bacteria |
| 126 | *Ornithinibacillus contaminans* | 0.049409 | 0.073236 | 0.021414 | Bacteria |
| 127 | *Sporosarcina newyorkensis* | 0.028822 | 0.103196 | 0.011897 | Bacteria |
| 128 | *Parageobacillus caldoxylosilyticus* | 0.004117 | 0.116511 | 0.021414 | Bacteria |
| 129 | *Bacillus oceanisediminis* | 0.053527 | 0.066578 | 0.019034 | Bacteria |
| 130 | *Pontibacillus litoralis* | 0.098818 | 0.036618 | 0.002379 | Bacteria |
| 131 | *Bacillus* phage SPG24 | 0.008235 | 0.126498 | 0.002379 | Viruses |
| 132 | *Enterobacter cloacae* | 0.078231 | 0.003329 | 0.054724 | Bacteria |
| 133 | *Virgibacillus soli* | 0.032939 | 0.046605 | 0.054724 | Bacteria |
| 134 | *Corynebacterium ammoniagenes* | 0.024705 | 0 | 0.109448 | Bacteria |
| 135 | *Bacillus shackletonii* | 0.049409 | 0.063249 | 0.021414 | Bacteria |
| 136 | *Sporosarcina koreensis* | 0.037057 | 0.013316 | 0.083276 | Bacteria |
| 137 | *Wohlfahrtiimonas* sp. 34C10-3-10 | 0.090583 | 0 | 0.042828 | Bacteria |
| 138 | *Desulfuribacillus alkaliarsenatis* | 0.032939 | 0.099867 | 0 | Bacteria |
| 139 | *Bacillus* sp. KCTC 13219 | 0.032939 | 0.069907 | 0.028552 | Bacteria |
| 140 | *Anoxybacillus flavithermus* | 0.008235 | 0.11984 | 0.002379 | Bacteria |
| 141 | *Oceanobacillus picturae* | 0.028822 | 0.069907 | 0.030931 | Bacteria |
| 142 | *Brevibacillus thermoruber* | 0.012352 | 0.049933 | 0.066621 | Bacteria |
| 143 | *Bacillus* sp. CC120222-01 | 0.037057 | 0.046605 | 0.042828 | Bacteria |
| 144 | *Sporosarcina psychrophila* | 0.053527 | 0.02996 | 0.042828 | Bacteria |
| 145 | *Pichia kudriavzevii* | 0.020587 | 0.009987 | 0.095172 | Eukaryota |
| 146 | *Bacillus mojavensis* | 0.057644 | 0.036618 | 0.030931 | Bacteria |
| 147 | *Paenibacillus dendritiformis* | 0.024705 | 0.016644 | 0.083276 | Bacteria |
| 148 | *Bacillus* sp. FJAT-27225 | 0.065879 | 0.046605 | 0.011897 | Bacteria |
| 149 | *Bacillus galactosidilyticus* | 0.008235 | 0.053262 | 0.061862 | Bacteria |
| 150 | *Kurthia massiliensis* | 0.078231 | 0.023302 | 0.021414 | Bacteria |
| 151 | *Clostridium* sp. 7_2_43FAA | 0 | 0.013316 | 0.109448 | Bacteria |
| 152 | *Bacillus* sp. MB2021 | 0.041174 | 0.066578 | 0.014276 | Bacteria |
| 153 | *Clostridium pasteurianum* | 0.061761 | 0.046605 | 0.011897 | Bacteria |
| 154 | *Listeria grayi* | 0.053527 | 0.02996 | 0.03569 | Bacteria |
| 155 | *Aquibacillus* sp. Marseille-P3518 | 0.053527 | 0.046605 | 0.019034 | Bacteria |
| 156 | *Vagococcus penaei* | 0.045292 | 0 | 0.071379 | Bacteria |
| 157 | *Bacillus dielmoensis* | 0.024705 | 0.069907 | 0.019034 | Bacteria |
| 158 | *Bacillus agaradhaerens* | 0.028822 | 0.069907 | 0.014276 | Bacteria |
| 159 | *Bacillus vallismortis* | 0.037057 | 0.023302 | 0.052345 | Bacteria |
| 160 | *Bacillus safensis* | 0.020587 | 0.013316 | 0.078517 | Bacteria |
| 161 | *Aneurinibacillus thermoaerophilus* | 0.012352 | 0.099867 | 0 | Bacteria |
| 162 | *Lactobacillus agilis* | 0.107053 | 0 | 0.004759 | Bacteria |
| 163 | *Rummeliibacillus stabekisii* | 0.020587 | 0.033289 | 0.057103 | Bacteria |
| 164 | *Enterococcus saccharolyticus* | 0.094701 | 0.003329 | 0.011897 | Bacteria |
| 165 | *Bacillus* sp. MUM 116 | 0.01647 | 0.083222 | 0.009517 | Bacteria |
| 166 | *Geobacillus* sp. BCO2 | 0.01647 | 0.063249 | 0.028552 | Bacteria |
| 167 | *Bacillus* sp. B14905 | 0.049409 | 0.019973 | 0.038069 | Bacteria |
| 168 | *Bacillus fumarioli* | 0.032939 | 0.05992 | 0.014276 | Bacteria |
| 169 | *Bacillus* sp. SB47 | 0.074114 | 0.023302 | 0.009517 | Bacteria |
| 170 | *Paenibacillus alvei* | 0.008235 | 0.05992 | 0.038069 | Bacteria |
| 171 | *Bacillus* sp. FJAT-29814 | 0 | 0.053262 | 0.049966 | Bacteria |
| 172 | *Bacillus* sp. B-jedd | 0.024705 | 0.049933 | 0.028552 | Bacteria |
| 173 | *Bacillus rubiinfantis* | 0.037057 | 0.056591 | 0.009517 | Bacteria |
| 174 | *Shigella sonnei* | 0.024705 | 0.006658 | 0.071379 | Bacteria |
| 175 | *Aneurinibacillus migulanus* | 0.008235 | 0.063249 | 0.030931 | Bacteria |
| 176 | *Paenibacillus* sp. oral taxon 786 | 0.037057 | 0.056591 | 0.007138 | Bacteria |
| 177 | *Bacillus niameyensis* | 0.037057 | 0.046605 | 0.016655 | Bacteria |
| 178 | *Streptococcus agalactiae* | 0.037057 | 0.046605 | 0.016655 | Bacteria |
| 179 | *Bacillus* sp. OxB-1 | 0.020587 | 0.019973 | 0.059483 | Bacteria |
| 180 | *Bacillus* sp. TH008 | 0.069996 | 0.013316 | 0.016655 | Bacteria |
| 181 | *Alcaligenes faecalis* | 0.004117 | 0 | 0.095172 | Bacteria |
| 182 | *Amphibacillus xylanus* | 0.045292 | 0.019973 | 0.030931 | Bacteria |
| 183 | *Bacillus* sp. A053 | 0.012352 | 0.05992 | 0.023793 | Bacteria |
| 184 | *Lysinibacillus saudimassiliensis* | 0.053527 | 0.006658 | 0.03569 | Bacteria |
| 185 | *Bacillus timonensis* | 0.020587 | 0.036618 | 0.038069 | Bacteria |
| 186 | *Virgibacillus alimentarius* | 0.032939 | 0.043276 | 0.019034 | Bacteria |
| 187 | *Bacillus altitudinis* | 0 | 0 | 0.095172 | Bacteria |
| 188 | *Bacillus* sp. JS | 0.012352 | 0.046605 | 0.03569 | Bacteria |
| 189 | *Geobacillus kaustophilus* | 0.004117 | 0.079893 | 0.009517 | Bacteria |
| 190 | *Lysinibacillus* sp. BF-4 | 0.032939 | 0.036618 | 0.023793 | Bacteria |
| 191 | *Wohlfahrtiimonas larvae* | 0.065879 | 0.003329 | 0.023793 | Bacteria |
| 192 | *Bacillus marisflavi* | 0.032939 | 0.023302 | 0.03569 | Bacteria |
| 193 | *Virgibacillus senegalensis* | 0.032939 | 0.036618 | 0.019034 | Bacteria |
| 194 | *Geobacillus* sp. LEMMY01 | 0.032939 | 0.053262 | 0.002379 | Bacteria |
| 195 | *Paraliobacillus ryukyuensis* | 0.045292 | 0.033289 | 0.009517 | Bacteria |
| 196 | *Bacillus xiamenensis* | 0.028822 | 0.013316 | 0.045207 | Bacteria |
| 197 | *Brevibacillus parabrevis* | 0.008235 | 0.023302 | 0.054724 | Bacteria |
| 198 | *Thermoactinomyces* sp. Gus2-1 | 0 | 0.083222 | 0.002379 | Bacteria |
| 199 | *Brevibacillus laterosporus* | 0.020587 | 0.043276 | 0.021414 | Bacteria |
| 200 | *Lysinibacillus* sp. ZYM-1 | 0.01647 | 0.02996 | 0.038069 | Bacteria |
| 201 | *Lysinibacillus massiliensis* | 0.020587 | 0.023302 | 0.040448 | Bacteria |
| 202 | *Bacillus gobiensis* | 0.037057 | 0.02996 | 0.016655 | Bacteria |
| 203 | *Eisenbergiella tayi* | 0.032939 | 0.049933 | 0 | Bacteria |
| 204 | *Bacillus megaterium* | 0.020587 | 0.039947 | 0.021414 | Bacteria |
| 205 | *Bacillus* sp. CMAA 1185 | 0.01647 | 0.043276 | 0.021414 | Bacteria |
| 206 | *Heliobacterium modesticaldum* | 0.012352 | 0.043276 | 0.023793 | Bacteria |
| 207 | *Paenibacillus* sp. FF9 | 0.045292 | 0.033289 | 0 | Bacteria |
| 208 | *Paucisalibacillus globulus* | 0.01647 | 0.039947 | 0.021414 | Bacteria |
| 209 | *Geobacillus* virus E3 | 0 | 0.076565 | 0 | Viruses |
| 210 | *Paenibacillus* sp. IHBB 10380 | 0.012352 | 0.046605 | 0.016655 | Bacteria |
| 211 | *Cohnella* sp. 6021052837 | 0.028822 | 0.046605 | 0 | Bacteria |
| 212 | *Atopostipes suicloacalis* | 0.049409 | 0.023302 | 0.002379 | Bacteria |
| 213 | *Geobacillus vulcani* | 0.045292 | 0.026631 | 0.002379 | Bacteria |
| 214 | *Bacillus panaciterrae* | 0 | 0.05992 | 0.014276 | Bacteria |
| 215 | *Bacillus massilioanorexius* | 0.028822 | 0.033289 | 0.011897 | Bacteria |
| 216 | *Terribacillus halophilus* | 0.020587 | 0.036618 | 0.016655 | Bacteria |
| 217 | *Lactobacillus crispatus* | 0 | 0 | 0.073759 | Bacteria |
| 218 | *Anoxybacillus* sp. DT3-1 | 0 | 0.073236 | 0 | Bacteria |
| 219 | *Geobacillus* sp. A8 | 0 | 0.073236 | 0 | Bacteria |
| 220 | *Mucor ambiguus* | 0.01647 | 0.02996 | 0.026172 | Eukaryota |
| 221 | *Acinetobacter lwoffii* | 0 | 0.003329 | 0.069 | Bacteria |
| 222 | *Bacillus weihenstephanensis* | 0.008235 | 0.006658 | 0.057103 | Bacteria |
| 223 | *Bacillus ginsengihumi* | 0.020587 | 0.02996 | 0.021414 | Bacteria |
| 224 | *Bacillus wiedmannii* | 0 | 0.009987 | 0.061862 | Bacteria |
| 225 | *Paenibacillus odorifer* | 0.008235 | 0.043276 | 0.019034 | Bacteria |
| 226 | *Bacillus vietnamensis* | 0.037057 | 0.023302 | 0.009517 | Bacteria |
| 227 | *Clostridium cochlearium* | 0.01647 | 0.053262 | 0 | Bacteria |
| 228 | *Oceanobacillus massiliensis* | 0.024705 | 0.006658 | 0.038069 | Bacteria |
| 229 | *Oceanobacillus limi* | 0.032939 | 0.026631 | 0.009517 | Bacteria |
| 230 | *Brevibacillus agri* | 0.012352 | 0.023302 | 0.03331 | Bacteria |
| 231 | *Clostridium* sp. 12(A) | 0.061761 | 0 | 0.007138 | Bacteria |
| 232 | *Aneurinibacillus* sp. XH2 | 0.004117 | 0.05992 | 0.004759 | Bacteria |
| 233 | *Thermoflavimicrobium dichotomicum* | 0.008235 | 0.046605 | 0.011897 | Bacteria |
| 234 | *Brevibacillus* sp. WF146 | 0.004117 | 0.043276 | 0.019034 | Bacteria |
| 235 | *Paenibacillus rhizosphaerae* | 0 | 0.003329 | 0.061862 | Bacteria |
| 236 | *Bacillus cecembensis* | 0.020587 | 0.02996 | 0.014276 | Bacteria |
| 237 | *Lysinibacillus sinduriensis* | 0.01647 | 0.009987 | 0.038069 | Bacteria |
| 238 | *Paenibacillus macerans* | 0.041174 | 0.013316 | 0.009517 | Bacteria |
| 239 | *Streptococcus salivarius* | 0.020587 | 0.026631 | 0.016655 | Bacteria |
| 240 | *Clostridium botulinum* | 0.032939 | 0.016644 | 0.014276 | Bacteria |
| 241 | *Thermoactinomyces* sp. CDF | 0 | 0.063249 | 0 | Bacteria |
| 242 | *Bacillus tuaregi* | 0.012352 | 0.036618 | 0.014276 | Bacteria |
| 243 | *Bacillus* sp. NSP9.1 | 0.037057 | 0.016644 | 0.009517 | Bacteria |
| 244 | *Bacillus* phage SP-10 | 0.004117 | 0.056591 | 0.002379 | Viruses |
| 245 | *Clostridium sporogenes* | 0.004117 | 0.036618 | 0.021414 | Bacteria |
| 246 | *Paenibacillus polymyxa* | 0.004117 | 0.053262 | 0.004759 | Bacteria |
| 247 | *Lysinibacillus contaminans* | 0.020587 | 0 | 0.040448 | Bacteria |
| 248 | *Acinetobacter bohemicus* | 0.01647 | 0.02996 | 0.014276 | Bacteria |
| 249 | *Bacillus firmus* | 0 | 0.019973 | 0.040448 | Bacteria |
| 250 | *Bacillus cihuensis* | 0.024705 | 0.033289 | 0.002379 | Bacteria |
| 251 | *Bacillus gaemokensis* | 0.01647 | 0.019973 | 0.023793 | Bacteria |
| 252 | *Bacillus* sp. FJAT-27997 | 0.01647 | 0.036618 | 0.007138 | Bacteria |
| 253 | *Enterococcus gallinarum* | 0.024705 | 0.006658 | 0.028552 | Bacteria |
| 254 | *Brevibacillus brevis* | 0.008235 | 0.013316 | 0.038069 | Bacteria |
| 255 | *Lactobacillus rhamnosus* | 0.01647 | 0.033289 | 0.009517 | Bacteria |
| 256 | *Gracilibacillus halophilus* | 0.004117 | 0.049933 | 0.004759 | Bacteria |
| 257 | *Psychrobacillus* sp. OK032 | 0.008235 | 0.033289 | 0.016655 | Bacteria |
| 258 | *Lactobacillus delbrueckii* | 0.004117 | 0.003329 | 0.049966 | Bacteria |
| 259 | *Sediminibacillus halophilus* | 0.008235 | 0.046605 | 0.002379 | Bacteria |
| 260 | *Bacillus flexus* | 0.020587 | 0.019973 | 0.016655 | Bacteria |
| 261 | *Sporosarcina* sp. HYO08 | 0.020587 | 0.019973 | 0.016655 | Bacteria |
| 262 | *Halobacillus hunanensis* | 0.01647 | 0.033289 | 0.007138 | Bacteria |
| 263 | *Fictibacillus enclensis* | 0.004117 | 0 | 0.052345 | Bacteria |
| 264 | *Bacillus badius* | 0.028822 | 0.019973 | 0.007138 | Bacteria |
| 265 | *Bacillus humi* | 0.024705 | 0.016644 | 0.014276 | Bacteria |
| 266 | *Bacillus cohnii* | 0.020587 | 0.02996 | 0.004759 | Bacteria |
| 267 | *Bacillus* phage BSNPO1 | 0.008235 | 0.046605 | 0 | Viruses |
| 268 | *Corynebacterium provencense* | 0 | 0 | 0.054724 | Bacteria |
| 269 | *Bacillus* sp. MKU004 | 0 | 0.049933 | 0.004759 | Bacteria |
| 270 | *Geobacillus* sp. WSUCF1 | 0.01647 | 0.023302 | 0.014276 | Bacteria |
| 271 | *Lysinibacillus fusiformis* | 0.008235 | 0.009987 | 0.03569 | Bacteria |
| 272 | *Geobacillus thermoleovorans* | 0 | 0.013316 | 0.040448 | Bacteria |
| 273 | *Geobacillus* sp. 44C | 0 | 0.053262 | 0 | Bacteria |
| 274 | *Bacillus* phage Mgbh1 | 0.01647 | 0.036618 | 0 | Viruses |
| 275 | *Bacillus mesonae* | 0.01647 | 0.026631 | 0.009517 | Bacteria |
| 276 | *Fictibacillus macauensis* | 0.01647 | 0.026631 | 0.009517 | Bacteria |
| 277 | *Bacillus* sp. FJAT-44921 | 0.004117 | 0.043276 | 0.004759 | Bacteria |
| 278 | *Bacillus* sp. 1310(2010) | 0.028822 | 0.023302 | 0 | Bacteria |
| 279 | *Bacillus* sp. CPSM8 | 0.045292 | 0.006658 | 0 | Bacteria |
| 280 | *Jeotgalicoccus psychrophilus* | 0.049409 | 0 | 0.002379 | Bacteria |
| 281 | *Brevibacillus* sp. CF112 | 0 | 0.013316 | 0.038069 | Bacteria |
| 282 | *Paenibacillus larvae* | 0.01647 | 0.013316 | 0.021414 | Bacteria |
| 283 | *Virgibacillus dokdonensis* | 0.024705 | 0.016644 | 0.009517 | Bacteria |
| 284 | *Bacillus aquimaris* | 0.004117 | 0.02996 | 0.016655 | Bacteria |
| 285 | *Bacillus dakarensis* | 0.008235 | 0.023302 | 0.019034 | Bacteria |
| 286 | *Lysinibacillus manganicus* | 0.020587 | 0.013316 | 0.016655 | Bacteria |
| 287 | *Bacillus* sp. RUPDJ | 0.020587 | 0.02996 | 0 | Bacteria |
| 288 | *Lysinibacillus boronitolerans* | 0.01647 | 0.009987 | 0.023793 | Bacteria |
| 289 | *Oceanobacillus* sp. Castelsardo | 0.008235 | 0.02996 | 0.011897 | Bacteria |
| 290 | *Tuberibacillus calidus* | 0.008235 | 0.02996 | 0.011897 | Bacteria |
| 291 | *Oxobacter pfennigii* | 0.032939 | 0.009987 | 0.007138 | Bacteria |
| 292 | *Bacillus* sp. MN5 | 0 | 0 | 0.049966 | Bacteria |
| 293 | *Paenibacillus* sp. FSL H7-0331 | 0 | 0.049933 | 0 | Bacteria |
| 294 | *Moellerella wisconsensis* | 0.032939 | 0 | 0.016655 | Bacteria |
| 295 | *Marinilactibacillus piezotolerans* | 0.01647 | 0.02996 | 0.002379 | Bacteria |
| 296 | *Lactobacillus reuteri* | 0.008235 | 0.006658 | 0.03331 | Bacteria |
| 297 | *Enterococcus mundtii* | 0.020587 | 0.013316 | 0.014276 | Bacteria |
| 298 | *Proteus vulgaris* | 0.004117 | 0.003329 | 0.040448 | Bacteria |
| 299 | *Bacillus* sp. FJAT-27445 | 0.004117 | 0.019973 | 0.023793 | Bacteria |
| 300 | *Bacillus korlensis* | 0.020587 | 0.019973 | 0.007138 | Bacteria |
| 301 | *Clostridioides mangenotii* | 0.008235 | 0.003329 | 0.03569 | Bacteria |
| 302 | *Bacillus atrophaeus* | 0.012352 | 0.013316 | 0.021414 | Bacteria |
| 303 | *Pusillimonas* sp. T7-7 | 0.004117 | 0 | 0.042828 | Bacteria |
| 304 | *Caenimonas* sp. SL110 | 0.004117 | 0.033289 | 0.009517 | Bacteria |
| 305 | *Bacillus koreensis* | 0.01647 | 0.023302 | 0.007138 | Bacteria |
| 306 | *Streptococcus equinus* | 0 | 0.013316 | 0.03331 | Bacteria |
| 307 | *Myroides injenensis* | 0 | 0.003329 | 0.042828 | Bacteria |
| 308 | *Atopococcus tabaci* | 0.024705 | 0.016644 | 0.004759 | Bacteria |
| 309 | *Bacillus* sp. F56 | 0.01647 | 0.019973 | 0.009517 | Bacteria |
| 310 | *Enterococcus massiliensis* | 0.01647 | 0.019973 | 0.009517 | Bacteria |
| 311 | *Natribacillus halophilus* | 0.008235 | 0.023302 | 0.014276 | Bacteria |
| 312 | *Oblitimonas alkaliphila* | 0.012352 | 0 | 0.03331 | Bacteria |
| 313 | *Anoxybacillus* sp. 103 | 0.028822 | 0.016644 | 0 | Bacteria |
| 314 | *Bacillus coahuilensis* | 0.008235 | 0.02996 | 0.007138 | Bacteria |
| 315 | *Lentibacillus jeotgali* | 0.01647 | 0.016644 | 0.011897 | Bacteria |
| 316 | *Exiguobacterium* sp. NG55 | 0.008235 | 0.036618 | 0 | Bacteria |
| 317 | *Bacillus* phage PBC4 | 0.024705 | 0.019973 | 0 | Viruses |
| 318 | *Bacillus fastidiosus* | 0.008235 | 0.026631 | 0.009517 | Bacteria |
| 319 | *Myroides profundi* | 0.008235 | 0 | 0.03569 | Bacteria |
| 320 | *Lysinibacillus* sp. F5 | 0.008235 | 0.033289 | 0.002379 | Bacteria |
| 321 | *Bacillus simplex* | 0.004117 | 0.013316 | 0.026172 | Bacteria |
| 322 | *Paucisalibacillus* sp. EB02 | 0.008235 | 0.023302 | 0.011897 | Bacteria |
| 323 | *Bacillus horikoshii* | 0.020587 | 0.013316 | 0.009517 | Bacteria |
| 324 | *Bacillus soli* | 0.012352 | 0.016644 | 0.014276 | Bacteria |
| 325 | *Enterococcus durans* | 0.01647 | 0.009987 | 0.016655 | Bacteria |
| 326 | *Clostridium* sp. DL-VIII | 0.020587 | 0.019973 | 0.002379 | Bacteria |
| 327 | *Numidum massiliense* | 0.012352 | 0.023302 | 0.007138 | Bacteria |
| 328 | *Macrococcus caseolyticus* | 0.012352 | 0.02996 | 0 | Bacteria |
| 329 | *Paenibacillus* sp. DMB5 | 0.012352 | 0.02996 | 0 | Bacteria |
| 330 | *Caryophanon latum* | 0.024705 | 0.003329 | 0.014276 | Bacteria |
| 331 | *Bacillus* sp. GeD10 | 0.004117 | 0 | 0.038069 | Bacteria |
| 332 | *Sporosarcina ureae* | 0.01647 | 0.006658 | 0.019034 | Bacteria |
| 333 | *Anaeromassilibacillus senegalensis* | 0.028822 | 0.013316 | 0 | Bacteria |
| 334 | *Parvimonas micra* | 0.004117 | 0.023302 | 0.014276 | Bacteria |
| 335 | *Defluviitalea phaphyphila* | 0.012352 | 0.026631 | 0.002379 | Bacteria |
| 336 | *Bacillus psychrosaccharolyticus* | 0.004117 | 0.013316 | 0.023793 | Bacteria |
| 337 | *Tuberibacillus* sp. Marseille-P3662 | 0.008235 | 0.023302 | 0.009517 | Bacteria |
| 338 | *Candidimonas bauzanensis* | 0.012352 | 0 | 0.028552 | Bacteria |
| 339 | *Gracilibacillus timonensis* | 0.012352 | 0 | 0.028552 | Bacteria |
| 340 | *Kurthia senegalensis* | 0.024705 | 0.006658 | 0.009517 | Bacteria |
| 341 | *Jeotgalibaca* sp. PTS2502 | 0.004117 | 0.019973 | 0.016655 | Bacteria |
| 342 | *Jeotgalicoccus halophilus* | 0.037057 | 0.003329 | 0 | Bacteria |
| 343 | *Oceanobacillus iheyensis* | 0.004117 | 0.009987 | 0.026172 | Bacteria |
| 344 | *Thermoactinomyces* sp. AS95 | 0 | 0.039947 | 0 | Bacteria |
| 345 | *Bacillus zhangzhouensis* | 0.004117 | 0 | 0.03569 | Bacteria |
| 346 | *Enterococcus aquimarinus* | 0.020587 | 0 | 0.019034 | Bacteria |
| 347 | *Clostridium* sp. DMHC 10 | 0.037057 | 0 | 0.002379 | Bacteria |
| 348 | *Bacillus gottheilii* | 0.008235 | 0.016644 | 0.014276 | Bacteria |
| 349 | *Bacillus lentus* | 0.008235 | 0.016644 | 0.014276 | Bacteria |
| 350 | *Castellaniella defragrans* | 0 | 0.003329 | 0.03569 | Bacteria |
| 351 | *Bacillus trypoxylicola* | 0.012352 | 0.026631 | 0 | Bacteria |
| 352 | *Domibacillus tundrae* | 0.01647 | 0.019973 | 0.002379 | Bacteria |
| 353 | *Bacillus litoralis* | 0 | 0.026631 | 0.011897 | Bacteria |
| 354 | *Bacillus clarkii* | 0.012352 | 0.016644 | 0.009517 | Bacteria |
| 355 | *Bacillus* sp. LF1 | 0.008235 | 0.019973 | 0.009517 | Bacteria |
| 356 | *Pusillimonas noertemannii* | 0.004117 | 0 | 0.03331 | Bacteria |
| 357 | *Cosenzaea myxofaciens* | 0.020587 | 0 | 0.016655 | Bacteria |
| 358 | *Bacillus* sp. NSP2.1 | 0 | 0.013316 | 0.023793 | Bacteria |
| 359 | *Leuconostoc citreum* | 0.012352 | 0.019973 | 0.004759 | Bacteria |
| 360 | *Jeotgalicoccus saudimassiliensis* | 0.037057 | 0 | 0 | Bacteria |
| 361 | *Sporolactobacillus terrae* | 0.020587 | 0.006658 | 0.009517 | Bacteria |
| 362 | *Lentibacillus halodurans* | 0.012352 | 0.009987 | 0.014276 | Bacteria |
| 363 | *Staphylococcus xylosus* | 0.024705 | 0 | 0.011897 | Bacteria |
| 364 | *Pontibacillus yanchengensis* | 0.004117 | 0.02996 | 0.002379 | Bacteria |
| 365 | *Paenibacillus senegalensis* | 0.020587 | 0.013316 | 0.002379 | Bacteria |
| 366 | *Lactobacillus casei* | 0.012352 | 0 | 0.023793 | Bacteria |
| 367 | *Paenibacillus naphthalenovorans* | 0.012352 | 0.016644 | 0.007138 | Bacteria |
| 368 | *Virgibacillus chiguensis* | 0.012352 | 0.016644 | 0.007138 | Bacteria |
| 369 | *Bacillus ligniniphilus* | 0.004117 | 0.019973 | 0.011897 | Bacteria |
| 370 | *Bacillus* sp. SDLI1 | 0.008235 | 0.013316 | 0.014276 | Bacteria |
| 371 | *Bacillus* sp. N24 | 0 | 0 | 0.03569 | Bacteria |
| 372 | *Myroides odoratus* | 0 | 0 | 0.03569 | Bacteria |
| 373 | *Geobacillus thermodenitrificans* | 0 | 0.033289 | 0.002379 | Bacteria |
| 374 | *Lactococcus lactis* | 0.012352 | 0.006658 | 0.016655 | Bacteria |
| 375 | *Paenibacillus popilliae* | 0.012352 | 0.006658 | 0.016655 | Bacteria |
| 376 | *Bacillus obstructivus* | 0.004117 | 0.026631 | 0.004759 | Bacteria |
| 377 | *Bacillus* sp. MRMR6 | 0.008235 | 0.019973 | 0.007138 | Bacteria |
| 378 | *Ornithinibacillus halophilus* | 0.020587 | 0.009987 | 0.004759 | Bacteria |
| 379 | *Bacillus marmarensis* | 0.032939 | 0 | 0.002379 | Bacteria |
| 380 | *Bacillus* sp. SG-1 | 0.024705 | 0.003329 | 0.007138 | Bacteria |
| 381 | *Bacillus caseinilyticus* | 0.004117 | 0.016644 | 0.014276 | Bacteria |
| 382 | *Bacillus* sp. FJAT-14578 | 0.004117 | 0.023302 | 0.007138 | Bacteria |
| 383 | *Domibacillus indicus* | 0.01647 | 0.013316 | 0.004759 | Bacteria |
| 384 | *Acinetobacter gerneri* | 0 | 0.003329 | 0.030931 | Bacteria |
| 385 | *Bacillus krulwichiae* | 0.008235 | 0.023302 | 0.002379 | Bacteria |
| 386 | *Bacillus* sp. 2_A_57_CT2 | 0 | 0.026631 | 0.007138 | Bacteria |
| 387 | *Bacillus* sp. 5B6 | 0 | 0.026631 | 0.007138 | Bacteria |
| 388 | *Bacillus horneckiae* | 0.004117 | 0.019973 | 0.009517 | Bacteria |
| 389 | *Globicatella sulfidifaciens* | 0.028822 | 0 | 0.004759 | Bacteria |
| 390 | *Bacillus* sp. ok061 | 0 | 0 | 0.03331 | Bacteria |
| 391 | *Bacillus* sp. J37 | 0.008235 | 0.003329 | 0.021414 | Bacteria |
| 392 | *Enteractinococcus helveticum* | 0.032939 | 0 | 0 | Bacteria |
| 393 | *Bacillus bogoriensis* | 0.012352 | 0.013316 | 0.007138 | Bacteria |
| 394 | *Bacillus alkalitelluris* | 0.004117 | 0.016644 | 0.011897 | Bacteria |
| 395 | *Streptococcus suis* | 0.008235 | 0.009987 | 0.014276 | Bacteria |
| 396 | *Brevibacillus massiliensis* | 0 | 0.013316 | 0.019034 | Bacteria |
| 397 | *Acinetobacter johnsonii* | 0.008235 | 0 | 0.023793 | Bacteria |
| 398 | *Carboxydocella* sp. ULO1 | 0.012352 | 0.009987 | 0.009517 | Bacteria |
| 399 | *Viridibacillus* sp. FSL H7-0596 | 0.012352 | 0.009987 | 0.009517 | Bacteria |
| 400 | *Bacillus* sp. SA1-12 | 0.004117 | 0.013316 | 0.014276 | Bacteria |
| 401 | *Staphylococcus carnosus* | 0.008235 | 0.023302 | 0 | Bacteria |
| 402 | *Proteus hauseri* | 0.012352 | 0 | 0.019034 | Bacteria |
| 403 | *Acidaminococcus intestini* | 0.024705 | 0.006658 | 0 | Bacteria |
| 404 | *Paeniclostridium sordellii* | 0.01647 | 0.009987 | 0.004759 | Bacteria |
| 405 | *Bacillus* sp. J33 | 0.020587 | 0.003329 | 0.007138 | Bacteria |
| 406 | *Advenella kashmirensis* | 0 | 0 | 0.030931 | Bacteria |
| 407 | *Bacillus* sp. GZT | 0 | 0 | 0.030931 | Bacteria |
| 408 | *Amphibacillus jilinensis* | 0.012352 | 0.006658 | 0.011897 | Bacteria |
| 409 | *Bacillus* sp. UNC41MFS5 | 0.012352 | 0.006658 | 0.011897 | Bacteria |
| 410 | *Paenisporosarcina indica* | 0.012352 | 0.006658 | 0.011897 | Bacteria |
| 411 | *Anoxybacillus ayderensis* | 0.008235 | 0.019973 | 0.002379 | Bacteria |
| 412 | *Prosthecobacter debontii* | 0.020587 | 0.009987 | 0 | Bacteria |
| 413 | *Enterococcus italicus* | 0.020587 | 0 | 0.009517 | Bacteria |
| 414 | *Bacillus massiliogorillae* | 0 | 0.013316 | 0.016655 | Bacteria |
| 415 | *Bacillus* sp. FJAT-18017 | 0.012352 | 0.003329 | 0.014276 | Bacteria |
| 416 | *Bacillus bataviensis* | 0.004117 | 0.023302 | 0.002379 | Bacteria |
| 417 | *Clostridium saccharobutylicum* | 0.01647 | 0.013316 | 0 | Bacteria |
| 418 | *Drancourtella massiliensis* | 0.01647 | 0.013316 | 0 | Bacteria |
| 419 | *Gemmatimonas phototrophica* | 0.01647 | 0.013316 | 0 | Bacteria |
| 420 | *Vibrio parahaemolyticus* | 0.008235 | 0 | 0.021414 | Bacteria |
| 421 | *Alteribacillus bidgolensis* | 0.008235 | 0.016644 | 0.004759 | Bacteria |
| 422 | *Anoxybacillus* sp. BCO1 | 0.008235 | 0.016644 | 0.004759 | Bacteria |
| 423 | *Bacillus aidingensis* | 0.008235 | 0.016644 | 0.004759 | Bacteria |
| 424 | *Bacillus persicus* | 0.008235 | 0.016644 | 0.004759 | Bacteria |
| 425 | *Bacillus salsus* | 0.008235 | 0.016644 | 0.004759 | Bacteria |
| 426 | *Acinetobacter* sp. ANC 5600 | 0 | 0.003329 | 0.026172 | Bacteria |
| 427 | *Planomicrobium glaciei* | 0.024705 | 0 | 0.004759 | Bacteria |
| 428 | *Gracilibacillus ureilyticus* | 0.008235 | 0.006658 | 0.014276 | Bacteria |
| 429 | *Pseudomonas aeruginosa* | 0.012352 | 0 | 0.016655 | Bacteria |
| 430 | *Beggiatoa alba* | 0.012352 | 0.016644 | 0 | Bacteria |
| 431 | *Paenibacillus* sp. IHB B 3084 | 0.012352 | 0.016644 | 0 | Bacteria |
| 432 | *Anaerobacillus alkalidiazotrophicus* | 0.004117 | 0.019973 | 0.004759 | Bacteria |
| 433 | *Aneurinibacillus tyrosinisolvens* | 0.004117 | 0.019973 | 0.004759 | Bacteria |
| 434 | *Beggiatoa leptomitiformis* | 0.004117 | 0.019973 | 0.004759 | Bacteria |
| 435 | *Bacillus* sp. FJAT-22058 | 0.01647 | 0.009987 | 0.002379 | Bacteria |
| 436 | *Jeotgalibacillus alimentarius* | 0.01647 | 0.009987 | 0.002379 | Bacteria |
| 437 | *Flavobacterium marinum* | 0 | 0 | 0.028552 | Bacteria |
| 438 | *Bacillus* sp. 1NLA3E | 0.012352 | 0.006658 | 0.009517 | Bacteria |
| 439 | *Paenibacillus selenitireducens* | 0.012352 | 0.006658 | 0.009517 | Bacteria |
| 440 | *Enterococcus* sp. RIT-PI-f | 0.01647 | 0 | 0.011897 | Bacteria |
| 441 | *Bacillus pseudomycoides* | 0 | 0.023302 | 0.004759 | Bacteria |
| 442 | *Geobacillus* sp. 46C-IIa | 0 | 0.023302 | 0.004759 | Bacteria |
| 443 | *Bacillus* phage vB_BanS-Tsamsa | 0.024705 | 0.003329 | 0 | Viruses |
| 444 | *Vagococcus* sp. D7T301 | 0.004117 | 0 | 0.023793 | Bacteria |
| 445 | *Bacillus oryziterrae* | 0.004117 | 0.016644 | 0.007138 | Bacteria |
| 446 | *Bacillus intestinalis* | 0.01647 | 0.006658 | 0.004759 | Bacteria |
| 447 | *Bacillus kribbensis* | 0 | 0.013316 | 0.014276 | Bacteria |
| 448 | *Bacillus bingmayongensis* | 0.004117 | 0.006658 | 0.016655 | Bacteria |
| 449 | *Bacillus* sp. UNC125MFCrub1.1 | 0.004117 | 0.006658 | 0.016655 | Bacteria |
| 450 | *Carnobacterium gallinarum* | 0.008235 | 0 | 0.019034 | Bacteria |
| 451 | *Enterococcus phoeniculicola* | 0.008235 | 0 | 0.019034 | Bacteria |
| 452 | *Bacillus* sp. CDB3 | 0 | 0.003329 | 0.023793 | Bacteria |
| 453 | *Lactobacillus coleohominis* | 0 | 0.003329 | 0.023793 | Bacteria |
| 454 | *Bacillus* sp. SJS | 0 | 0.019973 | 0.007138 | Bacteria |
| 455 | *Fictibacillus gelatini* | 0 | 0.019973 | 0.007138 | Bacteria |
| 456 | *Bacillus lonarensis* | 0.012352 | 0.009987 | 0.004759 | Bacteria |
| 457 | *Edaphobacillus lindanitolerans* | 0.012352 | 0.009987 | 0.004759 | Bacteria |
| 458 | *Staphylococcus lentus* | 0.024705 | 0 | 0.002379 | Bacteria |
| 459 | *Staphylococcus saprophyticus* | 0.01647 | 0.003329 | 0.007138 | Bacteria |
| 460 | *Burkholderia pseudomallei* | 0.008235 | 0.006658 | 0.011897 | Bacteria |
| 461 | *Selenomonas bovis* | 0 | 0.026631 | 0 | Bacteria |
| 462 | *Gilliamella apicola* | 0.012352 | 0 | 0.014276 | Bacteria |
| 463 | *Domibacillus* sp. SAB 38 | 0.008235 | 0.013316 | 0.004759 | Bacteria |
| 464 | *Geobacillus* sp. 12AMOR1 | 0.008235 | 0.013316 | 0.004759 | Bacteria |
| 465 | *Carnobacterium viridans* | 0.020587 | 0.003329 | 0.002379 | Bacteria |
| 466 | *Bacillus* sp. Leaf49 | 0 | 0 | 0.026172 | Bacteria |
| 467 | *Lactobacillus dextrinicus* | 0 | 0 | 0.026172 | Bacteria |
| 468 | *Paenibacillus yonginensis* | 0.012352 | 0.006658 | 0.007138 | Bacteria |
| 469 | *Viridibacillus arvi* | 0.01647 | 0 | 0.009517 | Bacteria |
| 470 | *Brevibacillus reuszeri* | 0 | 0.006658 | 0.019034 | Bacteria |
| 471 | *Weissella hellenica* | 0.012352 | 0.013316 | 0 | Bacteria |
| 472 | *Brackiella oedipodis* | 0.004117 | 0 | 0.021414 | Bacteria |
| 473 | *Brevibacillus* sp. OK042 | 0.004117 | 0 | 0.021414 | Bacteria |
| 474 | *Bacillus* sp. UNC437CL72CviS29 | 0.004117 | 0.016644 | 0.004759 | Bacteria |
| 475 | *Solibacillus isronensis* | 0.020587 | 0 | 0.004759 | Bacteria |
| 476 | *Bacillus halmapalus* | 0 | 0.013316 | 0.011897 | Bacteria |
| 477 | *Enterococcus canis* | 0.012352 | 0.003329 | 0.009517 | Bacteria |
| 478 | *Lactobacillus jensenii* | 0.012352 | 0.003329 | 0.009517 | Bacteria |
| 479 | *Ornithinibacillus scapharcae* | 0.012352 | 0.003329 | 0.009517 | Bacteria |
| 480 | *Vagococcus teuberi* | 0.012352 | 0.003329 | 0.009517 | Bacteria |
| 481 | *Varibaculum timonense* | 0.012352 | 0.003329 | 0.009517 | Bacteria |
| 482 | *Bacillus* sp. OK048 | 0.008235 | 0 | 0.016655 | Bacteria |
| 483 | *Enterococcus pallens* | 0.008235 | 0 | 0.016655 | Bacteria |
| 484 | *Lactobacillus plantarum* | 0.008235 | 0 | 0.016655 | Bacteria |
| 485 | *Weissella jogaejeotgali* | 0.008235 | 0.016644 | 0 | Bacteria |
| 486 | *Acinetobacter indicus* | 0 | 0.003329 | 0.021414 | Bacteria |
| 487 | *Bacillus pseudalcaliphilus* | 0 | 0.019973 | 0.004759 | Bacteria |
| 488 | *Bacillus* sp. CBA7126 | 0.024705 | 0 | 0 | Bacteria |
| 489 | *Trichococcus palustris* | 0.004117 | 0.013316 | 0.007138 | Bacteria |
| 490 | *Paenibacillus* sp. P1XP2 | 0.01647 | 0.003329 | 0.004759 | Bacteria |
| 491 | *Clostridium beijerinckii* | 0.008235 | 0.006658 | 0.009517 | Bacteria |
| 492 | *Virgibacillus* sp. SK37 | 0.008235 | 0.006658 | 0.009517 | Bacteria |
| 493 | *Clostridium magnum* | 0.004117 | 0.003329 | 0.016655 | Bacteria |
| 494 | *Corynebacterium glutamicum* | 0.004117 | 0.003329 | 0.016655 | Bacteria |
| 495 | *Lactobacillus mucosae* | 0.004117 | 0.003329 | 0.016655 | Bacteria |
| 496 | *Lactobacillus suebicus* | 0.004117 | 0.019973 | 0 | Bacteria |
| 497 | *Longilinea arvoryzae* | 0.008235 | 0.013316 | 0.002379 | Bacteria |
| 498 | *Bacillus* sp. 5mfcol3.1 | 0 | 0 | 0.023793 | Bacteria |
| 499 | *Bacillus* sp. TD41 | 0 | 0 | 0.023793 | Bacteria |
| 500 | *Jeotgalibacillus malaysiensis* | 0.008235 | 0.003329 | 0.011897 | Bacteria |
| 501 | *Blautia producta* | 0 | 0.023302 | 0 | Bacteria |
| 502 | *Aeribacillus* phage AP45 | 0 | 0.023302 | 0 | Viruses |
| 503 | *Acinetobacter pittii* | 0.004117 | 0 | 0.019034 | Bacteria |
| 504 | *Bacillus aryabhattai* | 0.004117 | 0.016644 | 0.002379 | Bacteria |
| 505 | *Bacillus* sp. UNC438CL73TsuS30 | 0.004117 | 0.016644 | 0.002379 | Bacteria |
| 506 | *Halobacillus dabanensis* | 0.004117 | 0.016644 | 0.002379 | Bacteria |
| 507 | *Pontibacillus halophilus* | 0.004117 | 0.016644 | 0.002379 | Bacteria |
| 508 | *Thermoactinomyces daqus* | 0.004117 | 0.016644 | 0.002379 | Bacteria |
| 509 | *Bhargavaea beijingensis* | 0.020587 | 0 | 0.002379 | Bacteria |
| 510 | *Halobacillus salinus* | 0.020587 | 0 | 0.002379 | Bacteria |
| 511 | *Anaerosalibacter* sp. Marseille-P3206 | 0.012352 | 0.003329 | 0.007138 | Bacteria |
| 512 | *Bacillus* sp. FJAT-25496 | 0.012352 | 0.003329 | 0.007138 | Bacteria |
| 513 | *Corynebacterium casei* | 0.012352 | 0.003329 | 0.007138 | Bacteria |
| 514 | *Salinicoccus albus* | 0.012352 | 0.003329 | 0.007138 | Bacteria |
| 515 | *Bacillus manliponensis* | 0.004117 | 0.006658 | 0.011897 | Bacteria |
| 516 | *Morganella morganii* | 0.008235 | 0 | 0.014276 | Bacteria |
| 517 | *Bacillus* sp. 522_BSPC | 0 | 0.003329 | 0.019034 | Bacteria |
| 518 | *Anoxybacillus pushchinoensis* | 0 | 0.019973 | 0.002379 | Bacteria |
| 519 | *Paenibacillus glucanolyticus* | 0 | 0.019973 | 0.002379 | Bacteria |
| 520 | *Paenibacillus durus* | 0.01647 | 0.003329 | 0.002379 | Bacteria |
| 521 | *Bacillus ndiopicus* | 0.012352 | 0 | 0.009517 | Bacteria |
| 522 | *Geobacillus* genomosp. 3 | 0.008235 | 0.013316 | 0 | Bacteria |
| 523 | *Acinetobacter radioresistens* | 0.012352 | 0 | 0.021414 | Bacteria |
| 524 | *Acinetobacter schindleri* | 0 | 0 | 0.021414 | Bacteria |
| 525 | *Acinetobacter* sp. NCu2D-2 | 0 | 0 | 0.021414 | Bacteria |
| 526 | *Bacillus australimaris* | 0 | 0 | 0.021414 | Bacteria |
| 527 | *Virgibacillus massiliensis* | 0 | 0 | 0.021414 | Bacteria |
| 528 | *Caldicoprobacter oshimai* | 0 | 0.016644 | 0.004759 | Bacteria |
| 529 | *Bacillus sinesaloumensis* | 0.012352 | 0.006658 | 0.002379 | Bacteria |
| 530 | *Bacillus weihaiensis* | 0.012352 | 0.006658 | 0.002379 | Bacteria |
| 531 | *Pediococcus pentosaceus* | 0.012352 | 0.006658 | 0.002379 | Bacteria |
| 532 | *Lactobacillus ruminis* | 0.004117 | 0.009987 | 0.007138 | Bacteria |
| 533 | *Zoogloea* sp. LCSB751 | 0.004117 | 0.009987 | 0.007138 | Bacteria |
| 534 | *Caryophanon tenue* | 0.01647 | 0 | 0.004759 | Bacteria |
| 535 | *Providencia heimbachae* | 0.01647 | 0 | 0.004759 | Bacteria |
| 536 | *Acinetobacter junii* | 0.008235 | 0.003329 | 0.009517 | Bacteria |
| 537 | *Bacillus vireti* | 0.008235 | 0.003329 | 0.009517 | Bacteria |
| 538 | *Bhargavaea cecembensis* | 0.008235 | 0.003329 | 0.009517 | Bacteria |
| 539 | *Bacillus* sp. FJAT-25547 | 0 | 0.006658 | 0.014276 | Bacteria |
| 540 | *Bacillus* sp. RRD69 | 0.004117 | 0 | 0.016655 | Bacteria |
| 541 | *Corynebacterium variabile* | 0.004117 | 0 | 0.016655 | Bacteria |
| 542 | *Lysinibacillus* sp. AR18-8 | 0.004117 | 0 | 0.016655 | Bacteria |
| 543 | *Morganella psychrotolerans* | 0.004117 | 0 | 0.016655 | Bacteria |
| 544 | *Cohnella thermotolerans* | 0.004117 | 0.016644 | 0 | Bacteria |
| 545 | *Thermicanus aegyptius* | 0.004117 | 0.016644 | 0 | Bacteria |
| 546 | *Bacillus* phage Basilisk | 0.004117 | 0.016644 | 0 | Viruses |
| 547 | *Bacillus* phage Bobb | 0.004117 | 0.016644 | 0 | Viruses |
| 548 | *Azospirillum lipoferum* | 0.008235 | 0.009987 | 0.002379 | Bacteria |
| 549 | *Domibacillus antri* | 0.008235 | 0.009987 | 0.002379 | Bacteria |
| 550 | *Tepidibacillus decaturensis* | 0.008235 | 0.009987 | 0.002379 | Bacteria |
| 551 | *Virgibacillus* sp. 6R | 0.008235 | 0.009987 | 0.002379 | Bacteria |
| 552 | *Bacillus* phage SPP1 | 0.008235 | 0.009987 | 0.002379 | Viruses |
| 553 | *Anaerorhabdus furcosa* | 0.020587 | 0 | 0 | Bacteria |
| 554 | *Enterococcus avium* | 0 | 0.013316 | 0.007138 | Bacteria |
| 555 | *Bacillus* sp. TS-2 | 0.012352 | 0.003329 | 0.004759 | Bacteria |
| 556 | *Bacillus* sp. FJAT-27986 | 0.004117 | 0.006658 | 0.009517 | Bacteria |
| 557 | *Chlamydia trachomatis* | 0.004117 | 0.006658 | 0.009517 | Bacteria |
| 558 | *Garciella nitratireducens* | 0.008235 | 0 | 0.011897 | Bacteria |
| 559 | *Bacillus alcalophilus* | 0 | 0.003329 | 0.016655 | Bacteria |
| 560 | *Allofustis seminis* | 0 | 0.019973 | 0 | Bacteria |
| 561 | *Geobacillus zalihae* | 0 | 0.019973 | 0 | Bacteria |
| 562 | *Geobacillus* sp. PA-3 | 0.004117 | 0.013316 | 0.002379 | Bacteria |
| 563 | *Staphylococcus vitulinus* | 0.01647 | 0.003329 | 0 | Bacteria |
| 564 | *Bacillus axarquiensis* | 0.008235 | 0.006658 | 0.004759 | Bacteria |
| 565 | *Paenibacillus terrigena* | 0.008235 | 0.006658 | 0.004759 | Bacteria |
| 566 | *Erysipelothrix larvae* | 0.012352 | 0 | 0.007138 | Bacteria |
| 567 | *Sporolactobacillus laevolacticus* | 0.012352 | 0 | 0.007138 | Bacteria |
| 568 | *Tenuibacillus multivorans* | 0.012352 | 0 | 0.007138 | Bacteria |
| 569 | *Vitreoscilla* sp. SN6 | 0.012352 | 0 | 0.007138 | Bacteria |
| 570 | *Bacillus endophyticus* | 0.004117 | 0.003329 | 0.011897 | Bacteria |
| 571 | *Shigella dysenteriae* | 0.004117 | 0.003329 | 0.011897 | Bacteria |
| 572 | *Acinetobacter gandensis* | 0 | 0 | 0.019034 | Bacteria |
| 573 | *Bacillus* sp. 0711P9-1 | 0 | 0 | 0.019034 | Bacteria |
| 574 | *Corynebacterium* sp. CNJ-954 | 0 | 0 | 0.019034 | Bacteria |
| 575 | *Bacillus* sp. FJAT-27245 | 0 | 0.016644 | 0.002379 | Bacteria |
| 576 | *Bifidobacterium adolescentis* | 0.012352 | 0.006658 | 0 | Bacteria |
| 577 | *Clostridium tetani* | 0.012352 | 0.006658 | 0 | Bacteria |
| 578 | *Listeria innocua* | 0.012352 | 0.006658 | 0 | Bacteria |
| 579 | *Methylibium* sp. NZG | 0.012352 | 0.006658 | 0 | Bacteria |
| 580 | *Bacillus* sp. NC2-31 | 0.004117 | 0.009987 | 0.004759 | Bacteria |
| 581 | *Lihuaxuella thermophila* | 0.004117 | 0.009987 | 0.004759 | Bacteria |
| 582 | *Paludibacter propionicigenes* | 0.004117 | 0.009987 | 0.004759 | Bacteria |
| 583 | *Rubrivivax benzoatilyticus* | 0.004117 | 0.009987 | 0.004759 | Bacteria |
| 584 | *Bacillus* sp. FJAT-26652 | 0.01647 | 0 | 0.002379 | Bacteria |
| 585 | *Jeotgalicoccus marinus* | 0.01647 | 0 | 0.002379 | Bacteria |
| 586 | *Maledivibacter halophilus* | 0.01647 | 0 | 0.002379 | Bacteria |
| 587 | *Providencia alcalifaciens* | 0.01647 | 0 | 0.002379 | Bacteria |
| 588 | *Lutispora thermophila* | 0.008235 | 0.003329 | 0.007138 | Bacteria |
| 589 | *Pectobacterium carotovorum* | 0.008235 | 0.003329 | 0.007138 | Bacteria |
| 590 | *Acetobacterium bakii* | 0 | 0.006658 | 0.011897 | Bacteria |
| 591 | *Bacillus shacheensis* | 0 | 0.006658 | 0.011897 | Bacteria |
| 592 | *Bacillus* sp. HMSC76G11 | 0 | 0.006658 | 0.011897 | Bacteria |
| 593 | *Acinetobacter* sp. HR7 | 0.004117 | 0 | 0.014276 | Bacteria |
| 594 | *Clostridioides difficile* | 0.004117 | 0 | 0.014276 | Bacteria |
| 595 | *Paenibacillus harenae* | 0.004117 | 0 | 0.014276 | Bacteria |
| 596 | *Alkaliphilus transvaalensis* | 0.008235 | 0.009987 | 0 | Bacteria |
| 597 | *Streptomyces alboniger* | 0.008235 | 0.009987 | 0 | Bacteria |
| 598 | *Virgibacillus subterraneus* | 0.008235 | 0.009987 | 0 | Bacteria |
| 599 | *Trichosporon asahii* | 0.008235 | 0.009987 | 0 | Eukaryota |
| 600 | *Polaromonas naphthalenivorans* | 0 | 0.013316 | 0.004759 | Bacteria |
| 601 | *Streptococcus parauberis* | 0 | 0.013316 | 0.004759 | Bacteria |
| 602 | *Bacillus* sp. BSC154 | 0.012352 | 0.003329 | 0.002379 | Bacteria |
| 603 | *Bacillus aurantiacus* | 0.004117 | 0.006658 | 0.007138 | Bacteria |
| 604 | *Bacillus drentensis* | 0.004117 | 0.006658 | 0.007138 | Bacteria |
| 605 | *Bacillus* sp. ES3 | 0.004117 | 0.006658 | 0.007138 | Bacteria |
| 606 | *Sporosarcina globispora* | 0.004117 | 0.006658 | 0.007138 | Bacteria |
| 607 | *Enterococcus sulfureus* | 0.008235 | 0 | 0.009517 | Bacteria |
| 608 | *Escherichia fergusonii* | 0.008235 | 0 | 0.009517 | Bacteria |
| 609 | *Bacillus malacitensis* | 0 | 0.003329 | 0.014276 | Bacteria |
| 610 | *Bacillus wakoensis* | 0 | 0.003329 | 0.014276 | Bacteria |
| 611 | *Leclercia adecarboxylata* | 0 | 0.003329 | 0.014276 | Bacteria |
| 612 | *Bacillus* sp. BS-02 | 0.004117 | 0.013316 | 0 | Bacteria |
| 613 | *Pseudoflavonifractor capillosus* | 0.004117 | 0.013316 | 0 | Bacteria |
| 614 | *Sporomusa malonica* | 0.004117 | 0.013316 | 0 | Bacteria |
| 615 | *Alicyclobacillus contaminans* | 0.008235 | 0.006658 | 0.002379 | Bacteria |
| 616 | *Dechloromonas aromatica* | 0.008235 | 0.006658 | 0.002379 | Bacteria |
| 617 | *Bacillus* sp. 105MF | 0 | 0.009987 | 0.007138 | Bacteria |
| 618 | *Clostridium perfringens* | 0 | 0.009987 | 0.007138 | Bacteria |
| 619 | *Effusibacillus pohliae* | 0 | 0.009987 | 0.007138 | Bacteria |
| 620 | *Ornithinibacillus californiensis* | 0.012352 | 0 | 0.004759 | Bacteria |
| 621 | *Providencia sneebia* | 0.012352 | 0 | 0.004759 | Bacteria |
| 622 | *Sporosarcina* sp. D27 | 0.012352 | 0 | 0.004759 | Bacteria |
| 623 | *Gorillibacterium massiliense* | 0.004117 | 0.003329 | 0.009517 | Bacteria |
| 624 | *Halobacillus massiliensis* | 0.004117 | 0.003329 | 0.009517 | Bacteria |
| 625 | *Oceanobacillus manasiensis* | 0.004117 | 0.003329 | 0.009517 | Bacteria |
| 626 | *Acinetobacter idrijaensis* | 0 | 0 | 0.016655 | Bacteria |
| 627 | *Bacillus* sp. H1a | 0 | 0 | 0.016655 | Bacteria |
| 628 | *Bacillus* sp. N35-10-4 | 0 | 0 | 0.016655 | Bacteria |
| 629 | *Myroides xuanwuensis* | 0 | 0 | 0.016655 | Bacteria |
| 630 | *Ochrobactrum intermedium* | 0 | 0 | 0.016655 | Bacteria |
| 631 | *Anaerobacillus alkalilacustris* | 0 | 0.016644 | 0 | Bacteria |
| 632 | *Anoxybacillus kamchatkensis* | 0 | 0.016644 | 0 | Bacteria |
| 633 | *Bacillus* sp. ECU0013 | 0 | 0.016644 | 0 | Bacteria |
| 634 | *Desulfitobacterium metallireducens* | 0 | 0.016644 | 0 | Bacteria |
| 635 | *Geobacillus* sp. B4113_201601 | 0 | 0.016644 | 0 | Bacteria |
| 636 | *Paenibacillus* sp. FSL R7-277 | 0 | 0.016644 | 0 | Bacteria |
| 637 | *Bacillus okhensis* | 0.004117 | 0.009987 | 0.002379 | Bacteria |
| 638 | *Chlamydia abortus* | 0.004117 | 0.009987 | 0.002379 | Bacteria |
| 639 | *Ilumatobacter coccineus* | 0.004117 | 0.009987 | 0.002379 | Bacteria |
| 640 | *Salinicoccus halodurans* | 0.004117 | 0.009987 | 0.002379 | Bacteria |
| 641 | *Yaniella halotolerans* | 0.01647 | 0 | 0 | Bacteria |
| 642 | *Bacillus* sp. FJAT-29937 | 0.008235 | 0.003329 | 0.004759 | Bacteria |
| 643 | *Clostridium akagii* | 0.008235 | 0.003329 | 0.004759 | Bacteria |
| 644 | *Dechloromonas denitrificans* | 0.008235 | 0.003329 | 0.004759 | Bacteria |
| 645 | *Oceanospirillum multiglobuliferum* | 0.008235 | 0.003329 | 0.004759 | Bacteria |
| 646 | *Tissierella praeacuta* | 0.008235 | 0.003329 | 0.004759 | Bacteria |
| 647 | *Bacillus luciferensis* | 0 | 0.006658 | 0.009517 | Bacteria |
| 648 | *Bacillus* sp. LL01 | 0 | 0.006658 | 0.009517 | Bacteria |
| 649 | *Paenibacillus macquariensis* | 0 | 0.006658 | 0.009517 | Bacteria |
| 650 | *Planococcus maritimus* | 0 | 0.006658 | 0.009517 | Bacteria |
| 651 | *Trichococcus ilyis* | 0 | 0.006658 | 0.009517 | Bacteria |
| 652 | *Acinetobacter* sp. WCHA34 | 0.004117 | 0 | 0.011897 | Bacteria |
| 653 | *Erysipelothrix rhusiopathiae* | 0.004117 | 0 | 0.011897 | Bacteria |
| 654 | *Erysipelothrix tonsillarum* | 0.004117 | 0 | 0.011897 | Bacteria |
| 655 | *Listeria fleischmannii* | 0.004117 | 0 | 0.011897 | Bacteria |
| 656 | *Francisella tularensis* | 0 | 0.013316 | 0.002379 | Bacteria |
| 657 | *Paenibacillus* sp. DMB20 | 0 | 0.013316 | 0.002379 | Bacteria |
| 658 | *Steroidobacter denitrificans* | 0 | 0.013316 | 0.002379 | Bacteria |
| 659 | *Weissella oryzae* | 0 | 0.013316 | 0.002379 | Bacteria |
| 660 | *Gemmatimonas* sp. SG8_23 | 0.012352 | 0.003329 | 0 | Bacteria |
| 661 | *Pseudobacteroides cellulosolvens* | 0.012352 | 0.003329 | 0 | Bacteria |
| 662 | *Staphylococcus cohnii* | 0.012352 | 0.003329 | 0 | Bacteria |
| 663 | *Staphylococcus fleurettii* | 0.012352 | 0.003329 | 0 | Bacteria |
| 664 | *Tepidibacillus* sp. HK-1 | 0.012352 | 0.003329 | 0 | Bacteria |
| 665 | *Azonexus hydrophilus* | 0.004117 | 0.006658 | 0.004759 | Bacteria |
| 666 | *Lysinibacillus varians* | 0.004117 | 0.006658 | 0.004759 | Bacteria |
| 667 | *Paucibacter* sp. KCTC 42545 | 0.004117 | 0.006658 | 0.004759 | Bacteria |
| 668 | *Piscibacillus halophilus* | 0.004117 | 0.006658 | 0.004759 | Bacteria |
| 669 | *Streptococcus equi* | 0.004117 | 0.006658 | 0.004759 | Bacteria |
| 670 | *Klebsiella aerogenes* | 0.008235 | 0 | 0.007138 | Bacteria |
| 671 | *Orrella dioscoreae* | 0.008235 | 0 | 0.007138 | Bacteria |
| 672 | *Pantoea ananatis* | 0.008235 | 0 | 0.007138 | Bacteria |
| 673 | *Sporosarcina* sp. ZBG7A | 0.008235 | 0 | 0.007138 | Bacteria |
| 674 | *Tetragenococcus muriaticus* | 0.008235 | 0 | 0.007138 | Bacteria |
| 675 | *Alicyclobacillus acidoterrestris* | 0 | 0.003329 | 0.011897 | Bacteria |
| 676 | *Rubeoparvulum massiliense* | 0 | 0.003329 | 0.011897 | Bacteria |
| 677 | *Chthoniobacter flavus* | 0.008235 | 0.006658 | 0 | Bacteria |
| 678 | *Paracoccus chinensis* | 0.008235 | 0.006658 | 0 | Bacteria |
| 679 | *Bacillus siamensis* | 0 | 0.009987 | 0.004759 | Bacteria |
| 680 | *Alkalibacterium subtropicum* | 0.012352 | 0 | 0.002379 | Bacteria |
| 681 | *Clostridium neonatale* | 0.012352 | 0 | 0.002379 | Bacteria |
| 682 | *Enterobacter hormaechei* | 0.012352 | 0 | 0.002379 | Bacteria |
| 683 | *Enterococcus hirae* | 0.012352 | 0 | 0.002379 | Bacteria |
| 684 | *Leptothrix cholodnii* | 0.012352 | 0 | 0.002379 | Bacteria |
| 685 | *Serratia marcescens* | 0.012352 | 0 | 0.002379 | Bacteria |
| 686 | *Paenisporosarcina* sp. TG20 | 0.004117 | 0.003329 | 0.007138 | Bacteria |
| 687 | *Paraliobacillus* sp. PM-2 | 0.004117 | 0.003329 | 0.007138 | Bacteria |
| 688 | *Virgibacillus siamensis* | 0.004117 | 0.003329 | 0.007138 | Bacteria |
| 689 | *Acinetobacter* sp. ANC 4945 | 0 | 0 | 0.014276 | Bacteria |
| 690 | *Bacillus* sp. 123MFChir2 | 0 | 0 | 0.014276 | Bacteria |
| 691 | *Bacillus* sp. ABP14 | 0 | 0 | 0.014276 | Bacteria |
| 692 | *Bacillus* sp. M 2-6 | 0 | 0 | 0.014276 | Bacteria |
| 693 | *Bordetella pertussis* | 0 | 0 | 0.014276 | Bacteria |
| 694 | *Aquabacterium parvum* | 0.004117 | 0.009987 | 0 | Bacteria |
| 695 | *Clostridium* sp. KNHs205 | 0.004117 | 0.009987 | 0 | Bacteria |
| 696 | *Geothrix fermentans* | 0.004117 | 0.009987 | 0 | Bacteria |
| 697 | *Lonsdalea quercina* | 0.004117 | 0.009987 | 0.007138 | Bacteria |
| 698 | *Nitrospira* sp. SCN 59-13 | 0.004117 | 0.009987 | 0 | Bacteria |
| 699 | *Nitrospira* sp. ST-bin5 | 0.004117 | 0.009987 | 0 | Bacteria |
| 700 | *Paenibacillus algorifonticola* | 0.004117 | 0.009987 | 0 | Bacteria |
| 701 | *Paenibacillus* sp. 1_12 | 0.004117 | 0.009987 | 0 | Bacteria |
| 702 | *Pontibacillus chungwhensis* | 0.004117 | 0.009987 | 0 | Bacteria |
| 703 | *Bacillus akibai* | 0.008235 | 0.003329 | 0.002379 | Bacteria |
| 704 | *Bacillus jeotgali* | 0.008235 | 0.003329 | 0.002379 | Bacteria |
| 705 | *Bacillus muralis* | 0.008235 | 0.003329 | 0.002379 | Bacteria |
| 706 | *Beggiatoa* sp. 4572_84 | 0.008235 | 0.003329 | 0.002379 | Bacteria |
| 707 | *Clostridium ihumii* | 0.008235 | 0.003329 | 0.002379 | Bacteria |
| 708 | *Gorillibacterium* sp. SN4 | 0.008235 | 0.003329 | 0.002379 | Bacteria |
| 709 | *Paenibacillus ginsengihumi* | 0.008235 | 0.003329 | 0.002379 | Bacteria |
| 710 | *Paenibacillus riograndensis* | 0.008235 | 0.003329 | 0.002379 | Bacteria |
| 711 | *Pedosphaera parvula* | 0.008235 | 0.003329 | 0.002379 | Bacteria |
| 712 | *Peptostreptococcus russellii* | 0.008235 | 0.003329 | 0.002379 | Bacteria |
| 713 | *Pseudomonas* sp. EGD-AK9 | 0.008235 | 0.003329 | 0.002379 | Bacteria |
| 714 | *Shimazuella kribbensis* | 0.008235 | 0.003329 | 0.002379 | Bacteria |
| 715 | *Tetragenococcus halophilus* | 0.008235 | 0.003329 | 0.002379 | Bacteria |
| 716 | *Carnobacterium* sp. CP1 | 0 | 0.006658 | 0.007138 | Bacteria |
| 717 | *Paenibacillus beijingensis* | 0 | 0.006658 | 0.007138 | Bacteria |
| 718 | *Acinetobacter equi* | 0.004117 | 0 | 0.009517 | Bacteria |
| 719 | *Acinetobacter towneri* | 0.004117 | 0 | 0.009517 | Bacteria |
| 720 | *Bacillus* sp. JKS001846 | 0.004117 | 0 | 0.009517 | Bacteria |
| 721 | *Bacillus* sp. NRRL B-14911 | 0.004117 | 0 | 0.009517 | Bacteria |
| 722 | *Enterococcus dispar* | 0.004117 | 0 | 0.009517 | Bacteria |
| 723 | *Isobaculum melis* | 0.004117 | 0 | 0.009517 | Bacteria |
| 724 | *Paenibacillus* sp. 11 | 0.004117 | 0 | 0.009517 | Bacteria |
| 725 | *Proteus* sp. H24 | 0.004117 | 0 | 0.009517 | Bacteria |
| 726 | *Timonella senegalensis* | 0.004117 | 0 | 0.009517 | Bacteria |
| 727 | *Vibrio cholerae* | 0.004117 | 0 | 0.009517 | Bacteria |
| 728 | *Anoxybacillus* sp. SK3-4 | 0.008235 | 0.013316 | 0 | Bacteria |
| 729 | *Epulopiscium* sp. SCG-C07WGA-EpuloA2 | 0 | 0.013316 | 0 | Bacteria |
| 730 | *Geobacillus* sp. G11MC16 | 0 | 0.013316 | 0 | Bacteria |
| 731 | *Marinococcus halophilus* | 0 | 0.013316 | 0 | Bacteria |
| 732 | *Paenibacillus* sp. BIHB4019 | 0 | 0.013316 | 0 | Bacteria |
| 733 | *Propionispora* sp. 2/2-37 | 0 | 0.013316 | 0 | Bacteria |
| 734 | *Bacillus indicus* | 0.004117 | 0.006658 | 0.002379 | Bacteria |
| 735 | *Dendrosporobacter quercicolus* | 0.004117 | 0.006658 | 0.002379 | Bacteria |
| 736 | *Enterococcus* sp. kppr-6 | 0.004117 | 0.006658 | 0.002379 | Bacteria |
| 737 | *Fuerstia marisgermanicae* | 0.004117 | 0.006658 | 0.002379 | Bacteria |
| 738 | *Jeotgalibacillus soli* Cunha et al. 2012 | 0.004117 | 0.006658 | 0.002379 | Bacteria |
| 739 | *Paenibacillus massiliensis* | 0.004117 | 0.006658 | 0.002379 | Bacteria |
| 740 | *Planococcus* sp. L10.15 | 0.004117 | 0.006658 | 0.002379 | Bacteria |
| 741 | *Bacillus chagannorensis* | 0.008235 | 0 | 0.004759 | Bacteria |
| 742 | *Bacillus* sp. FJAT-27231 | 0.008235 | 0 | 0.004759 | Bacteria |
| 743 | *Bacillus* sp. FJAT-27251 | 0.008235 | 0 | 0.004759 | Bacteria |
| 744 | *Burkholderia cepacia* | 0.008235 | 0 | 0.004759 | Bacteria |
| 745 | *Enterococcus canintestini* | 0.008235 | 0 | 0.004759 | Bacteria |
| 746 | *Enterococcus termitis* | 0.008235 | 0 | 0.004759 | Bacteria |
| 747 | *Lachnoanaerobaculum saburreum* | 0.008235 | 0 | 0.004759 | Bacteria |
| 748 | *Lactobacillus oris* | 0.008235 | 0 | 0.004759 | Bacteria |
| 749 | *Xenorhabdus bovienii* | 0.008235 | 0 | 0.004759 | Bacteria |
| 750 | *Acinetobacter kyonggiensis* | 0 | 0.003329 | 0.009517 | Bacteria |
| 751 | *Brevibacillus* sp. BC25 | 0 | 0.003329 | 0.009517 | Bacteria |
| 752 | *Paenibacillus ehimensis* | 0 | 0.003329 | 0.009517 | Bacteria |
| 753 | *Paenibacillus pini* | 0 | 0.003329 | 0.009517 | Bacteria |
| 754 | *Bacillus* sp. URHB0009 | 0 | 0.009987 | 0.002379 | Bacteria |
| 755 | *Clostridium oryzae* | 0 | 0.009987 | 0.002379 | Bacteria |
| 756 | *Clostridium* sp. Bc-iso-3 | 0 | 0.009987 | 0.002379 | Bacteria |
| 757 | *Clostridium* sp. HMP27 | 0 | 0.009987 | 0.002379 | Bacteria |
| 758 | *Haloferula* sp. BvORR071 | 0 | 0.009987 | 0.002379 | Bacteria |
| 759 | *Paenibacillus* sp. FSL H8-0548 | 0 | 0.009987 | 0.002379 | Bacteria |
| 760 | *Pirellula* sp. SH-Sr6A | 0 | 0.009987 | 0.002379 | Bacteria |
| 761 | *Thauera phenylacetica* | 0 | 0.009987 | 0.002379 | Bacteria |
| 762 | *Alicyclobacillus vulcanalis* | 0.012352 | 0 | 0 | Bacteria |
| 763 | *Clostridium acidisoli* | 0.012352 | 0 | 0 | Bacteria |
| 764 | *Lactobacillus paralimentarius* | 0.012352 | 0 | 0 | Bacteria |
| 765 | *Paraclostridium bifermentans* | 0.012352 | 0 | 0 | Bacteria |
| 766 | *Peptoniphilus* sp. KHD4 | 0.012352 | 0 | 0 | Bacteria |
| 767 | *Staphylococcus equorum* | 0.012352 | 0 | 0 | Bacteria |
| 768 | *Staphylococcus haemolyticus* | 0.012352 | 0 | 0 | Bacteria |
| 769 | *Staphylococcus warneri* | 0.012352 | 0 | 0 | Bacteria |
| 770 | *Geotrichum candidum* | 0.012352 | 0 | 0 | Eukaryota |
| 771 | *Alkalibacillus haloalkaliphilus* | 0.004117 | 0.003329 | 0.004759 | Bacteria |
| 772 | *Bacillus daliensis* | 0.004117 | 0.003329 | 0.004759 | Bacteria |
| 773 | *Bacillus subterraneus* | 0.004117 | 0.003329 | 0.004759 | Bacteria |
| 774 | *Carnobacterium inhibens* | 0.004117 | 0.003329 | 0.004759 | Bacteria |
| 775 | *Halolactibacillus halophilus* | 0.004117 | 0.003329 | 0.004759 | Bacteria |
| 776 | *Massilibacterium senegalense* | 0.004117 | 0.003329 | 0.004759 | Bacteria |
| 777 | *Paenibacillus wynnii* | 0.004117 | 0.003329 | 0.004759 | Bacteria |
| 778 | *Ruminococcus flavefaciens* | 0.004117 | 0.003329 | 0.004759 | Bacteria |
| 779 | *Yersinia enterocolitica* | 0.004117 | 0.003329 | 0.004759 | Bacteria |
| 780 | *Acinetobacter bouvetii* | 0 | 0 | 0.011897 | Bacteria |
| 781 | *Acinetobacter calcoaceticus* | 0 | 0 | 0.011897 | Bacteria |
| 782 | *Acinetobacter rudis* | 0 | 0 | 0.011897 | Bacteria |
| 783 | *Acinetobacter* sp. ANC 4149 | 0 | 0 | 0.011897 | Bacteria |
| 784 | *Bacillus patagoniensis* | 0 | 0 | 0.011897 | Bacteria |
| 785 | *Bacillus* sp. bc15 | 0 | 0 | 0.011897 | Bacteria |
| 786 | *Chitinophaga pinensis* | 0 | 0 | 0.011897 | Bacteria |
| 787 | *Corynebacterium diphtheriae* | 0 | 0 | 0.011897 | Bacteria |
| 788 | *Flavobacterium ummariense* | 0 | 0 | 0.011897 | Bacteria |
| 789 | *Myroides marinus* | 0 | 0 | 0.011897 | Bacteria |
| 790 | *Myroides* sp. ZB35 | 0 | 0 | 0.011897 | Bacteria |
| 791 | *Paenibacillus ihumii* | 0 | 0 | 0.011897 | Bacteria |
| 792 | *Proteus penneri* | 0 | 0 | 0.011897 | Bacteria |
| 793 | *Proteus* sp. 3M | 0 | 0 | 0.011897 | Bacteria |
| 794 | *Shewanella* sp. POL2 | 0 | 0 | 0.011897 | Bacteria |
| 795 | *Sphingobacterium* sp. T2 | 0 | 0 | 0.011897 | Bacteria |
| 796 | *Tetragenococcus solitarius* | 0 | 0 | 0.011897 | Bacteria |
| 797 | *Anoxybacillus suryakundensis* | 0.008235 | 0.003329 | 0 | Bacteria |
| 798 | *Blastopirellula marina* | 0.008235 | 0.003329 | 0 | Bacteria |
| 799 | *Carnobacterium mobile* | 0.008235 | 0.003329 | 0 | Bacteria |
| 800 | *Clostridium carboxidivorans* | 0.008235 | 0.003329 | 0 | Bacteria |
| 801 | *Dysgonomonas mossii* | 0.008235 | 0.003329 | 0 | Bacteria |
| 802 | *Ktedonobacter racemifer* | 0.008235 | 0.003329 | 0 | Bacteria |
| 803 | *Lactobacillus sunkii* | 0.008235 | 0.003329 | 0 | Bacteria |
| 804 | *Streptococcus infantarius* | 0.008235 | 0.003329 | 0 | Bacteria |
| 805 | *Trichococcus pasteurii* | 0.008235 | 0.003329 | 0 | Bacteria |
| 806 | *Aquabacterium* sp. NJ1 | 0 | 0.006658 | 0.004759 | Bacteria |
| 807 | *Bacillus abyssalis* | 0 | 0.006658 | 0.004759 | Bacteria |
| 808 | *Bacillus* sp. JCM 19034 | 0 | 0.006658 | 0.004759 | Bacteria |
| 809 | *Bacillus* sp. JCM 19041 | 0 | 0.006658 | 0.004759 | Bacteria |
| 810 | *Bacillus* sp. V-88 | 0 | 0.006658 | 0.004759 | Bacteria |
| 811 | *Clostridium acetireducens* | 0 | 0.006658 | 0.004759 | Bacteria |
| 812 | *Domibacillus enclensis* | 0 | 0.006658 | 0.004759 | Bacteria |
| 813 | *Fictibacillus solisalsi* | 0 | 0.006658 | 0.004759 | Bacteria |
| 814 | *Thioploca ingrica* | 0 | 0.006658 | 0.004759 | Bacteria |
| 815 | *Acinetobacter* sp. SFC | 0.004117 | 0 | 0.007138 | Bacteria |
| 816 | *Bacillus* sp. FJAT-27916 | 0.004117 | 0 | 0.007138 | Bacteria |
| 817 | *Bacillus* sp. NH11B | 0.004117 | 0 | 0.007138 | Bacteria |
| 818 | *Carnobacterium* sp. AT7 | 0.004117 | 0 | 0.007138 | Bacteria |
| 819 | *Castellaniella caeni* | 0.004117 | 0 | 0.007138 | Bacteria |
| 820 | *Edwardsiella tarda* | 0.004117 | 0 | 0.007138 | Bacteria |
| 821 | *Enterococcus columbae* | 0.004117 | 0 | 0.007138 | Bacteria |
| 822 | *Lachnoclostridium phytofermentans* | 0.004117 | 0 | 0.007138 | Bacteria |
| 823 | *Lactococcus garvieae* | 0.004117 | 0 | 0.007138 | Bacteria |
| 824 | *Lentibacillus persicus* | 0.004117 | 0 | 0.007138 | Bacteria |
| 825 | *Paenibacillus* sp. Marseille-P2472 | 0.004117 | 0 | 0.007138 | Bacteria |
| 826 | *Planococcus donghaensis* | 0.004117 | 0 | 0.007138 | Bacteria |
| 827 | *Sporolactobacillus vineae* | 0.004117 | 0 | 0.007138 | Bacteria |
| 828 | *Acidibacillus ferrooxidans* | 0.004117 | 0.006658 | 0 | Bacteria |
| 829 | *Cellulosilyticum* sp. I15G10I2 | 0.004117 | 0.006658 | 0 | Bacteria |
| 830 | *Cohnella laeviribosi* | 0.004117 | 0.006658 | 0 | Bacteria |
| 831 | *Dechloromonas agitata* | 0.004117 | 0.006658 | 0 | Bacteria |
| 832 | *Gracilibacillus kekensis* | 0.004117 | 0.006658 | 0 | Bacteria |
| 833 | *Kyrpidia tusciae* | 0.004117 | 0.006658 | 0 | Bacteria |
| 834 | *Lactococcus piscium* | 0.004117 | 0.006658 | 0 | Bacteria |
| 835 | *Lentibacillus amyloliquefaciens* | 0.004117 | 0.006658 | 0 | Bacteria |
| 836 | *Paenibacillus* sp. VT-16-81 | 0.004117 | 0.006658 | 0 | Bacteria |
| 837 | *Sulfuritalea hydrogenivorans* | 0.004117 | 0.006658 | 0 | Bacteria |
| 838 | *Batrachochytrium dendrobatidis* | 0.004117 | 0.006658 | 0 | Eukaryota |
| 839 | *Acetobacter syzygii* | 0.008235 | 0 | 0.002379 | Bacteria |
| 840 | *Anaerocolumna xylanovorans* | 0.008235 | 0 | 0.002379 | Bacteria |
| 841 | *Bacillus hemicellulosilyticus* | 0.008235 | 0 | 0.002379 | Bacteria |
| 842 | *Bacillus* sp. L_1B0_12 | 0.008235 | 0 | 0.002379 | Bacteria |
| 843 | *Bacteroides fragilis* | 0.008235 | 0 | 0.002379 | Bacteria |
| 844 | *Desemzia incerta* | 0.008235 | 0 | 0.002379 | Bacteria |
| 845 | *Globicatella* sp. HMSC072A10 | 0.008235 | 0 | 0.002379 | Bacteria |
| 846 | *Klebsiella oxytoca* | 0.008235 | 0 | 0.002379 | Bacteria |
| 847 | *Lacticigenium naphtae* | 0.008235 | 0 | 0.002379 | Bacteria |
| 848 | *Lactobacillus farciminis* | 0.008235 | 0 | 0.002379 | Bacteria |
| 849 | *Lactobacillus ghanensis* | 0.008235 | 0 | 0.002379 | Bacteria |
| 850 | *Marinomonas spartinae* | 0.008235 | 0 | 0.002379 | Bacteria |
| 851 | *Melissococcus plutonius* | 0.008235 | 0 | 0.002379 | Bacteria |
| 852 | *Paenibacillus* sp. OSY-SE | 0.008235 | 0 | 0.002379 | Bacteria |
| 853 | *Planococcus rifietoensis* | 0.008235 | 0 | 0.002379 | Bacteria |
| 854 | *Porphyrobacter* sp. LM 6 | 0.008235 | 0 | 0.002379 | Bacteria |
| 855 | *Providencia burhodogranariea* | 0.008235 | 0 | 0.002379 | Bacteria |
| 856 | *Staphylococcus epidermidis* | 0.008235 | 0 | 0.002379 | Bacteria |
| 857 | *Virgibacillus salinus* | 0.008235 | 0 | 0.002379 | Bacteria |
| 858 | *Virgibacillus* sp. CM-4 | 0.008235 | 0 | 0.002379 | Bacteria |
| 859 | *Stylonychia lemnae* | 0.008235 | 0 | 0.002379 | Eukaryota |
| 860 | *Bacillus* sp. FJAT-27215 | 0 | 0.003329 | 0.007138 | Bacteria |
| 861 | *Brevibacillus choshinensis* | 0 | 0.003329 | 0.007138 | Bacteria |
| 862 | *Brevibacillus* sp. Leaf182 | 0 | 0.003329 | 0.007138 | Bacteria |
| 863 | *Cloacibacterium normanense* | 0 | 0.003329 | 0.007138 | Bacteria |
| 864 | *Domibacillus iocasae* | 0 | 0.003329 | 0.007138 | Bacteria |
| 865 | *Domibacillus robiginosus* | 0 | 0.003329 | 0.007138 | Bacteria |
| 866 | *Geobacillus icigianus* | 0 | 0.003329 | 0.007138 | Bacteria |
| 867 | *Pseudoalteromonas luteoviolacea* | 0 | 0.003329 | 0.007138 | Bacteria |
| 868 | *Roseimaritima ulvae* | 0 | 0.003329 | 0.007138 | Bacteria |
| 869 | *Thermobacillus composti* | 0 | 0.003329 | 0.007138 | Bacteria |
| 870 | *Yersinia pestis* | 0 | 0.003329 | 0.007138 | Bacteria |
| 871 | *Aquincola tertiaricarbonis* | 0 | 0.009987 | 0 | Bacteria |
| 872 | *Bacillus halodurans* | 0 | 0.009987 | 0 | Bacteria |
| 873 | *Bacillus* sp. Pc3 | 0 | 0.009987 | 0 | Bacteria |
| 874 | *Bacillus* sp. UNCCL81 | 0 | 0.009987 | 0 | Bacteria |
| 875 | *Clostridium aceticum* | 0 | 0.009987 | 0 | Bacteria |
| 876 | *Clostridium* sp. N3C | 0 | 0.009987 | 0 | Bacteria |
| 877 | *Geobacillus jurassicus* | 0 | 0.009987 | 0 | Bacteria |
| 878 | *Geobacillus* sp. 15 | 0 | 0.009987 | 0 | Bacteria |
| 879 | *Gracilibacillus lacisalsi* | 0 | 0.009987 | 0 | Bacteria |
| 880 | *Kroppenstedtia eburnea* | 0 | 0.009987 | 0 | Bacteria |
| 881 | *Pseudomonas japonica* | 0 | 0.009987 | 0 | Bacteria |
| 882 | *Syntrophomonas zehnderi* | 0 | 0.009987 | 0 | Bacteria |
| 883 | *Weissella thailandensis* | 0 | 0.009987 | 0 | Bacteria |
| 884 | *Methanocaldococcus jannaschii* | 0 | 0.009987 | 0 | Archaea |
| 885 | *Aliicoccus persicus* | 0.004117 | 0.003329 | 0.002379 | Bacteria |
| 886 | *Alkalibacterium* sp. AK22 | 0.004117 | 0.003329 | 0.002379 | Bacteria |
| 887 | *Aneurinibacillus terranovensis* | 0.004117 | 0.003329 | 0.002379 | Bacteria |
| 888 | *Bacillus* sp. FJAT-20673 | 0.004117 | 0.003329 | 0.002379 | Bacteria |
| 889 | *Bhargavaea ginsengi* | 0.004117 | 0.003329 | 0.002379 | Bacteria |
| 890 | *Coprobacillus* sp. 8_1_38FAA | 0.004117 | 0.003329 | 0.002379 | Bacteria |
| 891 | *Cronobacter sakazakii* | 0.004117 | 0.003329 | 0.002379 | Bacteria |
| 892 | *Fusobacterium nucleatum* | 0.004117 | 0.003329 | 0.002379 | Bacteria |
| 893 | *Hydrogenophaga* sp. Root209 | 0.004117 | 0.003329 | 0.002379 | Bacteria |
| 894 | *Jeotgalibacillus campisalis* | 0.004117 | 0.003329 | 0.002379 | Bacteria |
| 895 | *Lactococcus raffinolactis* | 0.004117 | 0.003329 | 0.002379 | Bacteria |
| 896 | *Massilia* sp. CF038 | 0.004117 | 0.003329 | 0.002379 | Bacteria |
| 897 | *Opitutus* sp. GAS368 | 0.004117 | 0.003329 | 0.002379 | Bacteria |
| 898 | *Paenibacillus* sp. 1ZS3-15 | 0.004117 | 0.003329 | 0.002379 | Bacteria |
| 899 | *Rheinheimera* sp. SA_1 | 0.004117 | 0.003329 | 0.002379 | Bacteria |
| 900 | *Rugosibacter aromaticivorans* | 0.004117 | 0.003329 | 0.002379 | Bacteria |
| 901 | *Salibacterium halotolerans* | 0.004117 | 0.003329 | 0.002379 | Bacteria |
| 902 | *Salimicrobium flavidum* | 0.004117 | 0.003329 | 0.002379 | Bacteria |
| 903 | *Seinonella peptonophila* | 0.004117 | 0.003329 | 0.002379 | Bacteria |
| 904 | *Selenomonas ruminantium* | 0.004117 | 0.003329 | 0.002379 | Bacteria |
| 905 | *Sporosarcina* sp. EUR3 2.2.2 | 0.004117 | 0.003329 | 0.002379 | Bacteria |
| 906 | *Viridibacillus arenosi* | 0.004117 | 0.003329 | 0.002379 | Bacteria |
| 907 | *Bacillus* phage Shbh1 | 0.004117 | 0.003329 | 0.002379 | Viruses |
| 908 | *Acinetobacter nosocomialis* | 0 | 0 | 0.009517 | Bacteria |
| 909 | *Acinetobacter* sp. WCHA60 | 0 | 0 | 0.009517 | Bacteria |
| 910 | *Albidiferax* sp. OV413 | 0 | 0 | 0.009517 | Bacteria |
| 911 | *Bacillus* sp. 166amftsu | 0 | 0 | 0.009517 | Bacteria |
| 912 | *Bacillus* sp. ru9509.4 | 0 | 0 | 0.009517 | Bacteria |
| 913 | *Bacillus* sp. TD42 | 0 | 0 | 0.009517 | Bacteria |
| 914 | *Bacillus* sp. TH007 | 0 | 0 | 0.009517 | Bacteria |
| 915 | *Clostridium baratii* | 0 | 0 | 0.009517 | Bacteria |
| 916 | *Enterococcus rivorum* | 0 | 0 | 0.009517 | Bacteria |
| 917 | *Lactobacillus gasseri* | 0 | 0 | 0.009517 | Bacteria |
| 918 | *Lysinibacillus* sp. LK3 | 0 | 0 | 0.009517 | Bacteria |
| 919 | *Myroides* sp. A21 | 0 | 0 | 0.009517 | Bacteria |
| 920 | *Paenibacillus* sp. RU5A | 0 | 0 | 0.009517 | Bacteria |
| 921 | *Paenibacillus* sp. Soil522 | 0 | 0 | 0.009517 | Bacteria |
| 922 | *Pseudomonas* sp. TTU2014-080ASC | 0 | 0 | 0.009517 | Bacteria |
| 923 | *Shigella flexneri* | 0 | 0 | 0.009517 | Bacteria |
| 924 | *Thermoactinomyces* sp. DSM 45892 | 0 | 0 | 0.009517 | Bacteria |
| 925 | *Alkaliphilus metalliredigens* | 0 | 0.006658 | 0.002379 | Bacteria |
| 926 | *Bacillus boroniphilus* | 0 | 0.006658 | 0.002379 | Bacteria |
| 927 | *Bacillus* sp. 72 | 0 | 0.006658 | 0.002379 | Bacteria |
| 928 | *Bacillus* sp. YP1 | 0 | 0.006658 | 0.002379 | Bacteria |
| 929 | *Clostridium scatologenes* | 0 | 0.006658 | 0.002379 | Bacteria |
| 930 | *Clostridium* sp. DSM 8431 | 0 | 0.006658 | 0.002379 | Bacteria |
| 931 | *Exiguobacterium aurantiacum* | 0 | 0.006658 | 0.002379 | Bacteria |
| 932 | *Lactobacillus brevis* | 0 | 0.006658 | 0.002379 | Bacteria |
| 933 | *Nitrospira* sp. HN-bin3 | 0 | 0.006658 | 0.002379 | Bacteria |
| 934 | *Oceanobacillus* sp. E9 | 0 | 0.006658 | 0.002379 | Bacteria |
| 935 | *Paenibacillus phocaensis* | 0 | 0.006658 | 0.002379 | Bacteria |
| 936 | *Paenibacillus* sp. NAIST15-1 | 0 | 0.006658 | 0.002379 | Bacteria |
| 937 | *Polynucleobacter* sp. VK13 | 0 | 0.006658 | 0.002379 | Bacteria |
| 938 | *Prevotella paludivivens* | 0 | 0.006658 | 0.002379 | Bacteria |
| 939 | *Rhodobacter* sp. CACIA14H1 | 0 | 0.006658 | 0.002379 | Bacteria |
| 940 | *Stenotrophomonas maltophilia* | 0 | 0.006658 | 0.002379 | Bacteria |
| 941 | *Treponema socranskii* | 0 | 0.006658 | 0.002379 | Bacteria |
| 942 | *Acidiphilium multivorum* | 0.004117 | 0 | 0.004759 | Bacteria |
| 943 | *Acinetobacter bereziniae* | 0.004117 | 0 | 0.004759 | Bacteria |
| 944 | *Acinetobacter* sp. SFB | 0.004117 | 0 | 0.004759 | Bacteria |
| 945 | *Acinetobacter tandoii* | 0.004117 | 0 | 0.004759 | Bacteria |
| 946 | *Advenella mimigardefordensis* | 0.004117 | 0 | 0.004759 | Bacteria |
| 947 | *Alkalibacterium putridalgicola* | 0.004117 | 0 | 0.004759 | Bacteria |
| 948 | *Aquamicrobium defluvii* | 0.004117 | 0 | 0.004759 | Bacteria |
| 949 | *Aquaspirillum* sp. LM1 | 0.004117 | 0 | 0.004759 | Bacteria |
| 950 | *Bacillus* sp. JCM 19047 | 0.004117 | 0 | 0.004759 | Bacteria |
| 951 | *Bacillus* sp. NRRL B-41282 | 0.004117 | 0 | 0.004759 | Bacteria |
| 952 | *Bacillus testis* | 0.004117 | 0 | 0.004759 | Bacteria |
| 953 | *Balneatrix alpaca* | 0.004117 | 0 | 0.004759 | Bacteria |
| 954 | *Clostridium acetobutylicum* | 0.004117 | 0 | 0.004759 | Bacteria |
| 955 | *Clostridium* sp. Marseille-P299 | 0.004117 | 0 | 0.004759 | Bacteria |
| 956 | *Enterococcus* sp. HSIEG1 | 0.004117 | 0 | 0.004759 | Bacteria |
| 957 | *Flavobacterium psychrophilum* | 0.004117 | 0 | 0.004759 | Bacteria |
| 958 | *Holophaga foetida* | 0.004117 | 0 | 0.004759 | Bacteria |
| 959 | *Hungatella hathewayi* | 0.004117 | 0 | 0.004759 | Bacteria |
| 960 | *Inquilinus limosus* | 0.004117 | 0 | 0.004759 | Bacteria |
| 961 | *Jeotgalibaca dankookensis* | 0.004117 | 0 | 0.004759 | Bacteria |
| 962 | *Lacunisphaera limnophila* | 0.004117 | 0 | 0.004759 | Bacteria |
| 963 | *Limnohabitans* sp. 103DPR2 | 0.004117 | 0 | 0.004759 | Bacteria |
| 964 | *Marinospirillum minutulum* | 0.004117 | 0 | 0.004759 | Bacteria |
| 965 | *Nitrosococcus watsonii* | 0.004117 | 0 | 0.004759 | Bacteria |
| 966 | *Paenibacillus ferrarius* | 0.004117 | 0 | 0.004759 | Bacteria |
| 967 | *Paenibacillus kribbensis* | 0.004117 | 0 | 0.004759 | Bacteria |
| 968 | *Paenibacillus swuensis* | 0.004117 | 0 | 0.004759 | Bacteria |
| 969 | *Paenisporosarcina quisquiliarum* | 0.004117 | 0 | 0.004759 | Bacteria |
| 970 | *Paenisporosarcina* sp. HGH0030 | 0.004117 | 0 | 0.004759 | Bacteria |
| 971 | *Pelistega* sp. MC2 | 0.004117 | 0 | 0.004759 | Bacteria |
| 972 | *Providencia rustigianii* | 0.004117 | 0 | 0.004759 | Bacteria |
| 973 | *Sphingobacterium* sp. JB170 | 0.004117 | 0 | 0.004759 | Bacteria |
| 974 | *Sporosarcina* sp. P37 | 0.004117 | 0 | 0.004759 | Bacteria |
| 975 | *Taylorella equigenitalis* | 0.004117 | 0 | 0.004759 | Bacteria |
| 976 | *Tolumonas auensis* | 0.004117 | 0 | 0.004759 | Bacteria |
| 977 | *Acinetobacter* sp. NIPH 2100 | 0.008235 | 0 | 0 | Bacteria |
| 978 | *Bacillus infantis* | 0.008235 | 0 | 0 | Bacteria |
| 979 | *Bacillus* sp. H15-1 | 0.008235 | 0 | 0 | Bacteria |
| 980 | *Brachybacterium faecium* | 0.008235 | 0 | 0 | Bacteria |
| 981 | *Cellulosilyticum ruminicola* | 0.008235 | 0 | 0 | Bacteria |
| 982 | *Cephaloticoccus primus* | 0.008235 | 0 | 0 | Bacteria |
| 983 | *Chishuiella changwenlii* | 0.008235 | 0 | 0 | Bacteria |
| 984 | *Clostridium frigidicarnis* | 0.008235 | 0 | 0 | Bacteria |
| 985 | *Desulfurella acetivorans* | 0.008235 | 0 | 0 | Bacteria |
| 986 | *Dokdonella koreensis* | 0.008235 | 0 | 0 | Bacteria |
| 987 | *Enterococcus ureasiticus* | 0.008235 | 0 | 0 | Bacteria |
| 988 | *Facklamia ignava* | 0.008235 | 0 | 0 | Bacteria |
| 989 | *Faecalibacterium prausnitzii* | 0.008235 | 0 | 0 | Bacteria |
| 990 | *Geobacter* sp. OR-1 | 0.008235 | 0 | 0 | Bacteria |
| 991 | *Gulbenkiania mobilis* | 0.008235 | 0 | 0 | Bacteria |
| 992 | *Halomonas utahensis* | 0.008235 | 0 | 0 | Bacteria |
| 993 | *Ideonella* sp. B508-1 | 0.008235 | 0 | 0 | Bacteria |
| 994 | *Lactobacillus helveticus* | 0.008235 | 0 | 0 | Bacteria |
| 995 | *Lactococcus chungangensis* | 0.008235 | 0 | 0 | Bacteria |
| 996 | *Leuconostoc mesenteroides* | 0.008235 | 0 | 0 | Bacteria |
| 997 | *Marinilabilia salmonicolor* | 0.008235 | 0 | 0 | Bacteria |
| 998 | *Marinospirillum insulare* | 0.008235 | 0 | 0 | Bacteria |
| 999 | *Methyloceanibacter marginalis* | 0.008235 | 0 | 0 | Bacteria |
| 1000 | *Methyloversatilis universalis* | 0.008235 | 0 | 0 | Bacteria |
| 1001 | *Nafulsella turpanensis* | 0.008235 | 0 | 0 | Bacteria |
| 1002 | *Oligella* sp. HMSC09E12 | 0.008235 | 0 | 0 | Bacteria |
| 1003 | *Paenibacillus* sp. NFR01 | 0.008235 | 0 | 0 | Bacteria |
| 1004 | *Paracoccus tibetensis* | 0.008235 | 0 | 0 | Bacteria |
| 1005 | *Photobacterium aphoticum* | 0.008235 | 0 | 0 | Bacteria |
| 1006 | *Pseudorhodobacter psychrotolerans* | 0.008235 | 0 | 0 | Bacteria |
| 1007 | *Psychrobacillus psychrotolerans* | 0.008235 | 0 | 0 | Bacteria |
| 1008 | *Rheinheimera texasensis* | 0.008235 | 0 | 0 | Bacteria |
| 1009 | *Salinibacillus kushneri* | 0.008235 | 0 | 0 | Bacteria |
| 1010 | *Salinicoccus qingdaonensis* | 0.008235 | 0 | 0 | Bacteria |
| 1011 | *Serratia nematodiphila* | 0.008235 | 0 | 0 | Bacteria |
| 1012 | *Solibacillus silvestris* | 0.008235 | 0 | 0 | Bacteria |
| 1013 | *Staphylococcus agnetis* | 0.008235 | 0 | 0 | Bacteria |
| 1014 | *Staphylococcus pasteuri* | 0.008235 | 0 | 0 | Bacteria |
| 1015 | *Staphylococcus* sp. HMSC13A10 | 0.008235 | 0 | 0 | Bacteria |
| 1016 | *Staphylococcus* sp. MB371 | 0.008235 | 0 | 0 | Bacteria |
| 1017 | *Thermoflexibacter ruber* | 0.008235 | 0 | 0 | Bacteria |
| 1018 | *Veillonella magna* | 0.008235 | 0 | 0 | Bacteria |
| 1019 | *Yersinia aldovae* | 0.008235 | 0 | 0 | Bacteria |
| 1020 | *Candida tanzawaensis* | 0.008235 | 0 | 0 | Eukaryota |
| 1021 | *Meyerozyma guilliermondii* | 0.008235 | 0 | 0 | Eukaryota |
| 1022 | *Staphylococcus* phage BP39 | 0.008235 | 0 | 0 | Viruses |
| 1023 | *Staphylococcus* phage Andhra | 0.008235 | 0 | 0 | Viruses |
| 1024 | *Bacillus* phage 1102phi1-3 | 0.008235 | 0 | 0 | Viruses |
| 1025 | *Acholeplasma oculi* | 0 | 0.003329 | 0.004759 | Bacteria |
| 1026 | *Acinetobacter seifertii* | 0 | 0.003329 | 0.004759 | Bacteria |
| 1027 | *Acinetobacter seohaensis* | 0 | 0.003329 | 0.004759 | Bacteria |
| 1028 | *Alicyclobacillus shizuokensis* | 0 | 0.003329 | 0.004759 | Bacteria |
| 1029 | *Aquitalea magnusonii* | 0 | 0.003329 | 0.004759 | Bacteria |
| 1030 | *Bacillus novalis* | 0 | 0.003329 | 0.004759 | Bacteria |
| 1031 | *Bacillus oleronius* | 0 | 0.003329 | 0.004759 | Bacteria |
| 1032 | *Bacillus solimangrovi* | 0 | 0.003329 | 0.004759 | Bacteria |
| 1033 | *Bacillus* sp. B25(2016b) | 0 | 0.003329 | 0.004759 | Bacteria |
| 1034 | *Bacillus* sp. Soil745 | 0 | 0.003329 | 0.004759 | Bacteria |
| 1035 | *Clostridium amylolyticum* | 0 | 0.003329 | 0.004759 | Bacteria |
| 1036 | *Hathewaya proteolytica* | 0 | 0.003329 | 0.004759 | Bacteria |
| 1037 | *Paenibacillus pabuli* | 0 | 0.003329 | 0.004759 | Bacteria |
| 1038 | *Paenibacillus* sp. GP183 | 0 | 0.003329 | 0.004759 | Bacteria |
| 1039 | *Paenisporosarcina* sp. TG-14 | 0 | 0.003329 | 0.004759 | Bacteria |
| 1040 | *Paraglaciecola psychrophila* | 0 | 0.003329 | 0.004759 | Bacteria |
| 1041 | *Pelagirhabdus alkalitolerans* | 0 | 0.003329 | 0.004759 | Bacteria |
| 1042 | *Planomicrobium okeanokoites* | 0 | 0.003329 | 0.004759 | Bacteria |
| 1043 | *Pontibacillus marinus* | 0 | 0.003329 | 0.004759 | Bacteria |
| 1044 | *Rhizobacter* sp. Root404 | 0 | 0.003329 | 0.004759 | Bacteria |
| 1045 | *Sediminispirochaeta bajacaliforniensis* | 0 | 0.003329 | 0.004759 | Bacteria |
| 1046 | *Thalassobacillus devorans* | 0 | 0.003329 | 0.004759 | Bacteria |
| 1047 | *Acetobacterium dehalogenans* | 0.004117 | 0.003329 | 0 | Bacteria |
| 1048 | *Alicyclobacillus ferrooxydans* | 0.004117 | 0.003329 | 0 | Bacteria |
| 1049 | *Arcobacter butzleri* | 0.004117 | 0.003329 | 0 | Bacteria |
| 1050 | *Bacillus* sp. 17376 | 0.004117 | 0.003329 | 0 | Bacteria |
| 1051 | *Bacillus* sp. MSP5.4 | 0.004117 | 0.003329 | 0 | Bacteria |
| 1052 | *Bacillus* sp. NRRL B-41294 | 0.004117 | 0.003329 | 0 | Bacteria |
| 1053 | *Beduini massiliensis* | 0.004117 | 0.003329 | 0 | Bacteria |
| 1054 | *Brevibacterium senegalense* | 0.004117 | 0.003329 | 0 | Bacteria |
| 1055 | *Carnobacterium iners* | 0.004117 | 0.003329 | 0 | Bacteria |
| 1056 | *Chitinophaga niabensis* | 0.004117 | 0.003329 | 0 | Bacteria |
| 1057 | *Chloroflexus aggregans* | 0.004117 | 0.003329 | 0 | Bacteria |
| 1058 | *Chryseolinea serpens* | 0.004117 | 0.003329 | 0 | Bacteria |
| 1059 | *Citrobacter amalonaticus* | 0.004117 | 0.003329 | 0 | Bacteria |
| 1060 | *Clostridium lundense* | 0.004117 | 0.003329 | 0 | Bacteria |
| 1061 | *Desulfotomaculum geothermicum* | 0.004117 | 0.003329 | 0 | Bacteria |
| 1062 | *Desulfuromonas thiophila* | 0.004117 | 0.003329 | 0 | Bacteria |
| 1063 | *Dysgonomonas capnocytophagoides* | 0.004117 | 0.003329 | 0 | Bacteria |
| 1064 | *Fusicatenibacter saccharivorans* | 0.004117 | 0.003329 | 0 | Bacteria |
| 1065 | *Halobacteroides halobius* | 0.004117 | 0.003329 | 0 | Bacteria |
| 1066 | *Hydrogenophaga flava* | 0.004117 | 0.003329 | 0 | Bacteria |
| 1067 | *Lactobacillus nodensis* | 0.004117 | 0.003329 | 0 | Bacteria |
| 1068 | *Legionella pneumophila* | 0.004117 | 0.003329 | 0 | Bacteria |
| 1069 | *Mariniphaga anaerophila* | 0.004117 | 0.003329 | 0 | Bacteria |
| 1070 | *Massilia niastensis* | 0.004117 | 0.003329 | 0 | Bacteria |
| 1071 | *Merdibacter massiliensis* | 0.004117 | 0.003329 | 0 | Bacteria |
| 1072 | *Nitrospira defluvii* | 0.004117 | 0.003329 | 0 | Bacteria |
| 1073 | *Nitrospira* sp. ND1 | 0.004117 | 0.003329 | 0 | Bacteria |
| 1074 | *Paenibacillus alginolyticus* | 0.004117 | 0.003329 | 0 | Bacteria |
| 1075 | *Paenibacillus amylolyticus* | 0.004117 | 0.003329 | 0 | Bacteria |
| 1076 | *Paenibacillus pinihumi* | 0.004117 | 0.003329 | 0 | Bacteria |
| 1077 | *Pelobacter carbinolicus* | 0.004117 | 0.003329 | 0 | Bacteria |
| 1078 | *Porphyrobacter dokdonensis* | 0.004117 | 0.003329 | 0 | Bacteria |
| 1079 | *Proteiniclasticum ruminis* | 0.004117 | 0.003329 | 0 | Bacteria |
| 1080 | *Pseudomonas psychrotolerans* | 0.004117 | 0.003329 | 0 | Bacteria |
| 1081 | *Rhodoferax fermentans* | 0.004117 | 0.003329 | 0 | Bacteria |
| 1082 | *Rhodovulum* sp. PH10 | 0.004117 | 0.003329 | 0 | Bacteria |
| 1083 | *Rubinisphaera brasiliensis* | 0.004117 | 0.003329 | 0 | Bacteria |
| 1084 | *Salibacterium qingdaonense* | 0.004117 | 0.003329 | 0 | Bacteria |
| 1085 | *Sphaerotilus natans* | 0.004117 | 0.003329 | 0 | Bacteria |
| 1086 | *Sphingobacterium wenxiniae* | 0.004117 | 0.003329 | 0 | Bacteria |
| 1087 | *Streptococcus marmotae* | 0.004117 | 0.003329 | 0 | Bacteria |
| 1088 | *Sunxiuqinia dokdonensis* | 0.004117 | 0.003329 | 0 | Bacteria |
| 1089 | *Thauera terpenica* | 0.004117 | 0.003329 | 0 | Bacteria |
| 1090 | *Trichococcus flocculiformis* | 0.004117 | 0.003329 | 0 | Bacteria |
| 1091 | *Ichthyophthirius multifiliis* | 0.004117 | 0.003329 | 0 | Eukaryota |
| 1092 | *Acinetobacter brisouii* | 0 | 0 | 0.007138 | Bacteria |
| 1093 | *Acinetobacter* sp. 51m | 0 | 0 | 0.007138 | Bacteria |
| 1094 | *Acinetobacter* sp. MF4642 | 0 | 0 | 0.007138 | Bacteria |
| 1095 | *Acinetobacter* sp. MN12 | 0 | 0 | 0.007138 | Bacteria |
| 1096 | *Acinetobacter tjernbergiae* | 0 | 0 | 0.007138 | Bacteria |
| 1097 | *Acinetobacter ursingii* | 0 | 0 | 0.007138 | Bacteria |
| 1098 | *Adhaeribacter aquaticus* | 0 | 0 | 0.007138 | Bacteria |
| 1099 | *Alcanivorax hongdengensis* | 0 | 0 | 0.007138 | Bacteria |
| 1100 | *Altererythrobacter marensis* | 0 | 0 | 0.007138 | Bacteria |
| 1101 | *Bacillus selenatarsenatis* | 0 | 0 | 0.007138 | Bacteria |
| 1102 | *Bacillus* sp. FJAT-21955 | 0 | 0 | 0.007138 | Bacteria |
| 1103 | *Bacillus* sp. FJAT-27238 | 0 | 0 | 0.007138 | Bacteria |
| 1104 | *Bacillus* sp. G3(2015) | 0 | 0 | 0.007138 | Bacteria |
| 1105 | *Bacillus* sp. LK2 | 0 | 0 | 0.007138 | Bacteria |
| 1106 | *Bacillus* sp. N35-10-2 | 0 | 0 | 0.007138 | Bacteria |
| 1107 | *Bacillus* sp. UMTAT18 | 0 | 0 | 0.007138 | Bacteria |
| 1108 | *Beijerinckia mobilis* | 0 | 0 | 0.007138 | Bacteria |
| 1109 | *Blattabacterium punctulatus* | 0 | 0 | 0.007138 | Bacteria |
| 1110 | *Bordetella ansorpii* | 0 | 0 | 0.007138 | Bacteria |
| 1111 | *Bordetella* sp. N | 0 | 0 | 0.007138 | Bacteria |
| 1112 | *Carnobacterium divergens* | 0 | 0 | 0.007138 | Bacteria |
| 1113 | *Clostridium haemolyticum* | 0 | 0 | 0.007138 | Bacteria |
| 1114 | *Clostridium tunisiense* | 0 | 0 | 0.007138 | Bacteria |
| 1115 | *Cohnella kolymensis* | 0 | 0 | 0.007138 | Bacteria |
| 1116 | *Comamonas kerstersii* | 0 | 0 | 0.007138 | Bacteria |
| 1117 | *Dehalobacter* sp. E1 | 0 | 0 | 0.007138 | Bacteria |
| 1118 | *Enterococcus malodoratus* | 0 | 0 | 0.007138 | Bacteria |
| 1119 | *Enterococcus silesiacus* | 0 | 0 | 0.007138 | Bacteria |
| 1120 | *Enterococcus villorum* | 0 | 0 | 0.007138 | Bacteria |
| 1121 | *Escherichia albertii* | 0 | 0 | 0.007138 | Bacteria |
| 1122 | *Facklamia miroungae* | 0 | 0 | 0.007138 | Bacteria |
| 1123 | *Flavobacterium rivuli* | 0 | 0 | 0.007138 | Bacteria |
| 1124 | *Haemophilus influenzae* | 0 | 0 | 0.007138 | Bacteria |
| 1125 | *Halothiobacillus* sp. LS2 | 0 | 0 | 0.007138 | Bacteria |
| 1126 | *Lactobacillus hokkaidonensis* | 0 | 0 | 0.007138 | Bacteria |
| 1127 | *Lactobacillus paracasei* | 0 | 0 | 0.007138 | Bacteria |
| 1128 | *Myroides guanonis* | 0 | 0 | 0.007138 | Bacteria |
| 1129 | *Paenibacillus assamensis* | 0 | 0 | 0.007138 | Bacteria |
| 1130 | *Paenibacillus* sp. GM2 | 0 | 0 | 0.007138 | Bacteria |
| 1131 | *Paenibacillus* sp. TCA20 | 0 | 0 | 0.007138 | Bacteria |
| 1132 | *Ruminococcus bromii* | 0 | 0 | 0.007138 | Bacteria |
| 1133 | *Sphingobacterium paucimobilis* | 0 | 0 | 0.007138 | Bacteria |
| 1134 | *Sporanaerobacter* sp. PP17-6a | 0 | 0 | 0.007138 | Bacteria |
| 1135 | *Streptococcus pyogenes* | 0 | 0 | 0.007138 | Bacteria |
| 1136 | *Taylorella asinigenitalis* | 0 | 0 | 0.007138 | Bacteria |
| 1137 | *Thiobacillus denitrificans* | 0 | 0 | 0.007138 | Bacteria |
| 1138 | *Xanthomonas arboricola* | 0 | 0 | 0.007138 | Bacteria |
| 1139 | *Acaryochloris marina* | 0 | 0.006658 | 0 | Bacteria |
| 1140 | *Acinetobacter* sp. CIP-A165 | 0 | 0.006658 | 0 | Bacteria |
| 1141 | *Alicyclobacillus* sp. RIFOXYA1_FULL_53_8 | 0 | 0.006658 | 0 | Bacteria |
| 1142 | *Aneurinibacillus soli* | 0 | 0.006658 | 0 | Bacteria |
| 1143 | *Anoxybacillus* sp. KU2-6(11) | 0 | 0.006658 | 0 | Bacteria |
| 1144 | *Anoxybacillus* sp. P3H1B | 0 | 0.006658 | 0 | Bacteria |
| 1145 | *Bacillus* sp. FJAT-26390 | 0 | 0.006658 | 0 | Bacteria |
| 1146 | *Bacteroides helcogenes* | 0 | 0.006658 | 0 | Bacteria |
| 1147 | *Beggiatoa* sp. PS | 0 | 0.006658 | 0 | Bacteria |
| 1148 | *Bifidobacterium kashiwanohense* | 0 | 0.006658 | 0 | Bacteria |
| 1149 | *Bryobacter aggregatus* | 0 | 0.006658 | 0 | Bacteria |
| 1150 | *Caldanaerobacter subterraneus* | 0 | 0.006658 | 0 | Bacteria |
| 1151 | *Caulobacter vibrioides* | 0 | 0.006658 | 0 | Bacteria |
| 1152 | *Cellvibrio mixtus* | 0 | 0.006658 | 0 | Bacteria |
| 1153 | *Chryseobacterium gleum* | 0 | 0.006658 | 0 | Bacteria |
| 1154 | *Chthonomonas calidirosea* | 0 | 0.006658 | 0 | Bacteria |
| 1155 | *Clostridium* sp. BL8 | 0 | 0.006658 | 0 | Bacteria |
| 1156 | *Clostridium tyrobutyricum* | 0 | 0.006658 | 0 | Bacteria |
| 1157 | *Desulfitobacterium dichloroeliminans* | 0 | 0.006658 | 0 | Bacteria |
| 1158 | *Desulfuromonas* sp. TF | 0 | 0.006658 | 0 | Bacteria |
| 1159 | *Dietzia cinnamea* | 0 | 0.006658 | 0 | Bacteria |
| 1160 | *Dolosigranulum pigrum* | 0 | 0.006658 | 0 | Bacteria |
| 1161 | *Exiguobacterium* sp. Leaf196 | 0 | 0.006658 | 0 | Bacteria |
| 1162 | *Geobacillus* sp. 44B | 0 | 0.006658 | 0 | Bacteria |
| 1163 | *Geobacillus* sp. Sah69 | 0 | 0.006658 | 0 | Bacteria |
| 1164 | *Geobacillus* sp. Y4.1MC1 | 0 | 0.006658 | 0 | Bacteria |
| 1165 | *Geobacillus subterraneus* | 0 | 0.006658 | 0 | Bacteria |
| 1166 | *Geobacillus thermocatenulatus* | 0 | 0.006658 | 0 | Bacteria |
| 1167 | *Geobacter lovleyi* | 0 | 0.006658 | 0 | Bacteria |
| 1168 | *Haliea salexigens* | 0 | 0.006658 | 0 | Bacteria |
| 1169 | *Halobacillus halophilus* | 0 | 0.006658 | 0 | Bacteria |
| 1170 | *Hydrocarboniphaga daqingensis* | 0 | 0.006658 | 0 | Bacteria |
| 1171 | *Ideonella sakaiensis* | 0 | 0.006658 | 0 | Bacteria |
| 1172 | *Kocuria* sp. CNJ-770 | 0 | 0.006658 | 0 | Bacteria |
| 1173 | *Lactobacillus curvatus* | 0 | 0.006658 | 0 | Bacteria |
| 1174 | *Leptolyngbya boryana* | 0 | 0.006658 | 0 | Bacteria |
| 1175 | *Lewinella cohaerens* | 0 | 0.006658 | 0 | Bacteria |
| 1176 | *Microcystis aeruginosa* | 0 | 0.006658 | 0 | Bacteria |
| 1177 | *Nitrosomonas* sp. Nm58 | 0 | 0.006658 | 0 | Bacteria |
| 1178 | *Nostoc punctiforme* | 0 | 0.006658 | 0 | Bacteria |
| 1179 | *Paenibacillus borealis* | 0 | 0.006658 | 0 | Bacteria |
| 1180 | *Paenibacillus* sp. 32352 | 0 | 0.006658 | 0 | Bacteria |
| 1181 | *Paenibacillus* sp. FSL R7-0273 | 0 | 0.006658 | 0 | Bacteria |
| 1182 | *Paenibacillus* sp. LC231 | 0 | 0.006658 | 0 | Bacteria |
| 1183 | *Paenibacillus* sp. UNC499MF | 0 | 0.006658 | 0 | Bacteria |
| 1184 | *Planctomicrobium piriforme* | 0 | 0.006658 | 0 | Bacteria |
| 1185 | *Planctomyces* sp. SH-PL14 | 0 | 0.006658 | 0 | Bacteria |
| 1186 | *Polaromonas* sp. CG9_12 | 0 | 0.006658 | 0 | Bacteria |
| 1187 | *Pontibacter indicus* | 0 | 0.006658 | 0 | Bacteria |
| 1188 | *Pontibacter roseus* | 0 | 0.006658 | 0 | Bacteria |
| 1189 | *Roseivirga spongicola* | 0 | 0.006658 | 0 | Bacteria |
| 1190 | *Rubrobacter xylanophilus* | 0 | 0.006658 | 0 | Bacteria |
| 1191 | *Salimicrobium halophilum* | 0 | 0.006658 | 0 | Bacteria |
| 1192 | *Sandaracinus amylolyticus* | 0 | 0.006658 | 0 | Bacteria |
| 1193 | *Streptomyces laurentii* | 0 | 0.006658 | 0 | Bacteria |
| 1194 | *Streptomyces rubidus* | 0 | 0.006658 | 0 | Bacteria |
| 1195 | *Sunxiuqinia elliptica* | 0 | 0.006658 | 0 | Bacteria |
| 1196 | *Tepidimicrobium xylanilyticum* | 0 | 0.006658 | 0 | Bacteria |
| 1197 | *Terribacillus aidingensis* | 0 | 0.006658 | 0 | Bacteria |
| 1198 | *Thiorhodovibrio* sp. 970 | 0 | 0.006658 | 0 | Bacteria |
| 1199 | *Penicillium steckii* | 0 | 0.006658 | 0 | Eukaryota |
| 1200 | *Geobacillus* phage GBSV1 | 0 | 0.006658 | 0 | Viruses |
| 1201 | *Acetobacter pasteurianus* | 0.004117 | 0 | 0.002379 | Bacteria |
| 1202 | *Agrobacterium tumefaciens* | 0.004117 | 0 | 0.002379 | Bacteria |
| 1203 | *Bacillus* sp. FJAT-22090 | 0.004117 | 0 | 0.002379 | Bacteria |
| 1204 | *Beggiatoa* sp. IS2 | 0.004117 | 0 | 0.002379 | Bacteria |
| 1205 | *Bibersteinia trehalosi* | 0.004117 | 0 | 0.002379 | Bacteria |
| 1206 | *Burkholderia ubonensis* | 0.004117 | 0 | 0.002379 | Bacteria |
| 1207 | *Cardiobacterium valvarum* | 0.004117 | 0 | 0.002379 | Bacteria |
| 1208 | *Christensenella minuta* | 0.004117 | 0 | 0.002379 | Bacteria |
| 1209 | *Clostridium intestinale* | 0.004117 | 0 | 0.002379 | Bacteria |
| 1210 | *Clostridium puniceum* | 0.004117 | 0 | 0.002379 | Bacteria |
| 1211 | *Clostridium saccharoperbutylacetonicum* | 0.004117 | 0 | 0.002379 | Bacteria |
| 1212 | *Clostridium sulfidigenes* | 0.004117 | 0 | 0.002379 | Bacteria |
| 1213 | *Clostridium tetanomorphum* | 0.004117 | 0 | 0.002379 | Bacteria |
| 1214 | *Corynebacterium flavescens* | 0.004117 | 0 | 0.002379 | Bacteria |
| 1215 | *Corynebacterium jeikeium* | 0.004117 | 0 | 0.002379 | Bacteria |
| 1216 | *Coxiella* sp. RIFCSPHIGHO2_12_FULL_42_15 | 0.004117 | 0 | 0.002379 | Bacteria |
| 1217 | *Domibacillus* sp. SAOS 44 | 0.004117 | 0 | 0.002379 | Bacteria |
| 1218 | *Enterobacter mori* | 0.004117 | 0 | 0.002379 | Bacteria |
| 1219 | *Enterococcus moraviensis* | 0.004117 | 0 | 0.002379 | Bacteria |
| 1220 | *Erwinia tracheiphila* | 0.004117 | 0 | 0.002379 | Bacteria |
| 1221 | *Eubacterium* sp. AB3007 | 0.004117 | 0 | 0.002379 | Bacteria |
| 1222 | *Fabibacter misakiensis* | 0.004117 | 0 | 0.002379 | Bacteria |
| 1223 | *Flavobacterium indicum* | 0.004117 | 0 | 0.002379 | Bacteria |
| 1224 | *Gemella asaccharolytica* | 0.004117 | 0 | 0.002379 | Bacteria |
| 1225 | *Globicatella sanguinis* | 0.004117 | 0 | 0.002379 | Bacteria |
| 1226 | *Hafnia alvei* | 0.004117 | 0 | 0.002379 | Bacteria |
| 1227 | *Helicobacter pylori* | 0.004117 | 0 | 0.002379 | Bacteria |
| 1228 | *Lactobacillus murinus* | 0.004117 | 0 | 0.002379 | Bacteria |
| 1229 | *Lactobacillus vaccinostercus* | 0.004117 | 0 | 0.002379 | Bacteria |
| 1230 | *Lactobacillus vaginalis* | 0.004117 | 0 | 0.002379 | Bacteria |
| 1231 | *Lactobacillus wasatchensis* | 0.004117 | 0 | 0.002379 | Bacteria |
| 1232 | *Legionella massiliensis* | 0.004117 | 0 | 0.002379 | Bacteria |
| 1233 | *Legionella waltersii* | 0.004117 | 0 | 0.002379 | Bacteria |
| 1234 | *Marinagarivorans algicola* | 0.004117 | 0 | 0.002379 | Bacteria |
| 1235 | *Marinilactibacillus psychrotolerans* | 0.004117 | 0 | 0.002379 | Bacteria |
| 1236 | *Methylibium* sp. CF059 | 0.004117 | 0 | 0.002379 | Bacteria |
| 1237 | *Microbulbifer* sp. HZ11 | 0.004117 | 0 | 0.002379 | Bacteria |
| 1238 | *Microvirgula aerodenitrificans* | 0.004117 | 0 | 0.002379 | Bacteria |
| 1239 | *Nitrospira* sp. SG-bin1 | 0.004117 | 0 | 0.002379 | Bacteria |
| 1240 | *Oceanimonas* sp. GK1 | 0.004117 | 0 | 0.002379 | Bacteria |
| 1241 | *Oribacterium* sp. oral taxon 078 | 0.004117 | 0 | 0.002379 | Bacteria |
| 1242 | *Ottowia thiooxydans* | 0.004117 | 0 | 0.002379 | Bacteria |
| 1243 | *Oxalobacter formigenes* | 0.004117 | 0 | 0.002379 | Bacteria |
| 1244 | *Paenibacillus fonticola* | 0.004117 | 0 | 0.002379 | Bacteria |
| 1245 | *Paenibacillus glacialis* | 0.004117 | 0 | 0.002379 | Bacteria |
| 1246 | *Paenibacillus lactis* | 0.004117 | 0 | 0.002379 | Bacteria |
| 1247 | *Paenibacillus pasadenensis* | 0.004117 | 0 | 0.002379 | Bacteria |
| 1248 | *Paenibacillus* sp. yr247 | 0.004117 | 0 | 0.002379 | Bacteria |
| 1249 | *Parabacteroides* sp. Marseille-P3160 | 0.004117 | 0 | 0.002379 | Bacteria |
| 1250 | *Paraburkholderia aspalathi* | 0.004117 | 0 | 0.002379 | Bacteria |
| 1251 | *Paraburkholderia megapolitana* | 0.004117 | 0 | 0.002379 | Bacteria |
| 1252 | *Pasteurella multocida* | 0.004117 | 0 | 0.002379 | Bacteria |
| 1253 | *Pedobacter ruber* | 0.004117 | 0 | 0.002379 | Bacteria |
| 1254 | *Phocea massiliensis* | 0.004117 | 0 | 0.002379 | Bacteria |
| 1255 | *Planococcus massiliensis* | 0.004117 | 0 | 0.002379 | Bacteria |
| 1256 | *Planococcus plakortidis* | 0.004117 | 0 | 0.002379 | Bacteria |
| 1257 | *Proteiniborus ethanoligenes* | 0.004117 | 0 | 0.002379 | Bacteria |
| 1258 | *Pseudobacteriovorax antillogorgiicola* | 0.004117 | 0 | 0.002379 | Bacteria |
| 1259 | *Pseudonocardia acaciae* | 0.004117 | 0 | 0.002379 | Bacteria |
| 1260 | *Renibacterium salmoninarum* | 0.004117 | 0 | 0.002379 | Bacteria |
| 1261 | *Rhizobacter gummiphilus* | 0.004117 | 0 | 0.002379 | Bacteria |
| 1262 | *Saccharibacillus sacchari* | 0.004117 | 0 | 0.002379 | Bacteria |
| 1263 | *Salinicoccus roseus* | 0.004117 | 0 | 0.002379 | Bacteria |
| 1264 | *Salinicoccus* sp. YB14-2 | 0.004117 | 0 | 0.002379 | Bacteria |
| 1265 | *Serratia odorifera* | 0.004117 | 0 | 0.002379 | Bacteria |
| 1266 | *Serratia symbiotica* | 0.004117 | 0 | 0.002379 | Bacteria |
| 1267 | *Sphingobacterium lactis* | 0.004117 | 0 | 0.002379 | Bacteria |
| 1268 | *Sphingobacterium mizutaii* | 0.004117 | 0 | 0.002379 | Bacteria |
| 1269 | *Staphylococcus auricularis* | 0.004117 | 0 | 0.002379 | Bacteria |
| 1270 | *Stenotrophomonas* sp. MB339 | 0.004117 | 0 | 0.002379 | Bacteria |
| 1271 | *Streptococcus sobrinus* | 0.004117 | 0 | 0.002379 | Bacteria |
| 1272 | *Streptococcus uberis* | 0.004117 | 0 | 0.002379 | Bacteria |
| 1273 | *Succinatimonas hippei* | 0.004117 | 0 | 0.002379 | Bacteria |
| 1274 | *Sulfuricella denitrificans* | 0.004117 | 0 | 0.002379 | Bacteria |
| 1275 | *Thalassobacillus cyri* | 0.004117 | 0 | 0.002379 | Bacteria |
| 1276 | *Thiomonas* sp. CB2 | 0.004117 | 0 | 0.002379 | Bacteria |
| 1277 | *Thorsellia anophelis* | 0.004117 | 0 | 0.002379 | Bacteria |
| 1278 | *Variovorax paradoxus* | 0.004117 | 0 | 0.002379 | Bacteria |
| 1279 | *Xylophilus ampelinus* | 0.004117 | 0 | 0.002379 | Bacteria |
| 1280 | *Acanthamoeba castellanii* | 0.004117 | 0 | 0.002379 | Eukaryota |
| 1281 | *Kuraishia capsulata* | 0.004117 | 0 | 0.002379 | Eukaryota |
| 1282 | *Oxytricha trifallax* | 0.004117 | 0 | 0.002379 | Eukaryota |
| 1283 | *Acinetobacter guillouiae* | 0 | 0.003329 | 0.002379 | Bacteria |
| 1284 | *Acinetobacter kookii* | 0 | 0.003329 | 0.002379 | Bacteria |
| 1285 | *Acinetobacter* sp. 983759 | 0 | 0.003329 | 0.002379 | Bacteria |
| 1286 | *Acinetobacter* sp. P8-3-8 | 0 | 0.003329 | 0.002379 | Bacteria |
| 1287 | *Arachidicoccus* sp. BS20 | 0 | 0.003329 | 0.002379 | Bacteria |
| 1288 | *Arthrobacter* sp. W1 | 0 | 0.003329 | 0.002379 | Bacteria |
| 1289 | *Bacillus aerophilus* | 0 | 0.003329 | 0.002379 | Bacteria |
| 1290 | *Bacillus mannanilyticus* | 0 | 0.003329 | 0.002379 | Bacteria |
| 1291 | *Bacillus* sp. 491mf | 0 | 0.003329 | 0.002379 | Bacteria |
| 1292 | *Bacillus* sp. FJAT-25509 | 0 | 0.003329 | 0.002379 | Bacteria |
| 1293 | *Bacillus* sp. FJAT-27264 | 0 | 0.003329 | 0.002379 | Bacteria |
| 1294 | *Bartonella tamiae* | 0 | 0.003329 | 0.002379 | Bacteria |
| 1295 | *Bellilinea caldifistulae* | 0 | 0.003329 | 0.002379 | Bacteria |
| 1296 | *Bordetella holmesii* | 0 | 0.003329 | 0.002379 | Bacteria |
| 1297 | *Carnobacterium alterfunditum* | 0 | 0.003329 | 0.002379 | Bacteria |
| 1298 | *Catalinimonas alkaloidigena* | 0 | 0.003329 | 0.002379 | Bacteria |
| 1299 | *Chitinophaga filiformis* | 0 | 0.003329 | 0.002379 | Bacteria |
| 1300 | *Clostridium butyricum* | 0 | 0.003329 | 0.002379 | Bacteria |
| 1301 | *Clostridium cellulovorans* | 0 | 0.003329 | 0.002379 | Bacteria |
| 1302 | *Clostridium saudiense* | 0 | 0.003329 | 0.002379 | Bacteria |
| 1303 | *Clostridium* sp. ASBs410 | 0 | 0.003329 | 0.002379 | Bacteria |
| 1304 | *Collinsella aerofaciens* | 0 | 0.003329 | 0.002379 | Bacteria |
| 1305 | *Comamonas terrae* | 0 | 0.003329 | 0.002379 | Bacteria |
| 1306 | *Desulfitobacterium hafniense* | 0 | 0.003329 | 0.002379 | Bacteria |
| 1307 | *Desulfosporosinus lacus* | 0 | 0.003329 | 0.002379 | Bacteria |
| 1308 | *Desulfotomaculum gibsoniae* | 0 | 0.003329 | 0.002379 | Bacteria |
| 1309 | *Desulfovibrio inopinatus* | 0 | 0.003329 | 0.002379 | Bacteria |
| 1310 | *Desulfuribacillus stibiiarsenatis* | 0 | 0.003329 | 0.002379 | Bacteria |
| 1311 | *Empedobacter brevis* | 0 | 0.003329 | 0.002379 | Bacteria |
| 1312 | *Enterobacter asburiae* | 0 | 0.003329 | 0.002379 | Bacteria |
| 1313 | *Enterococcus pseudoavium* | 0 | 0.003329 | 0.002379 | Bacteria |
| 1314 | *Eremococcus coleocola* | 0 | 0.003329 | 0.002379 | Bacteria |
| 1315 | *Erwinia typographi* | 0 | 0.003329 | 0.002379 | Bacteria |
| 1316 | *Fictibacillus arsenicus* | 0 | 0.003329 | 0.002379 | Bacteria |
| 1317 | *Fictibacillus* sp. FJAT-27399 | 0 | 0.003329 | 0.002379 | Bacteria |
| 1318 | *Flavisolibacter ginsengisoli* | 0 | 0.003329 | 0.002379 | Bacteria |
| 1319 | *Flavobacterium succinicans* | 0 | 0.003329 | 0.002379 | Bacteria |
| 1320 | *Fluviicola taffensis* | 0 | 0.003329 | 0.002379 | Bacteria |
| 1321 | *Fodinicurvata fenggangensis* | 0 | 0.003329 | 0.002379 | Bacteria |
| 1322 | *Giesbergeria anulus* | 0 | 0.003329 | 0.002379 | Bacteria |
| 1323 | *Gottschalkia acidurici* | 0 | 0.003329 | 0.002379 | Bacteria |
| 1324 | *Halobacillus kuroshimensis* | 0 | 0.003329 | 0.002379 | Bacteria |
| 1325 | *Hyalangium minutum* | 0 | 0.003329 | 0.002379 | Bacteria |
| 1326 | *Hydrogenophaga palleronii* | 0 | 0.003329 | 0.002379 | Bacteria |
| 1327 | *Hylemonella gracilis* | 0 | 0.003329 | 0.002379 | Bacteria |
| 1328 | *Intrasporangium oryzae* | 0 | 0.003329 | 0.002379 | Bacteria |
| 1329 | *Lautropia* sp. SCN 69-89 | 0 | 0.003329 | 0.002379 | Bacteria |
| 1330 | *Listeria rocourtiae* | 0 | 0.003329 | 0.002379 | Bacteria |
| 1331 | *Methyloversatilis discipulorum* | 0 | 0.003329 | 0.002379 | Bacteria |
| 1332 | *Mucilaginibacter paludis* | 0 | 0.003329 | 0.002379 | Bacteria |
| 1333 | *Novispirillum itersonii* | 0 | 0.003329 | 0.002379 | Bacteria |
| 1334 | *Paenibacillus antibioticophila* | 0 | 0.003329 | 0.002379 | Bacteria |
| 1335 | *Paenibacillus sanguinis* | 0 | 0.003329 | 0.002379 | Bacteria |
| 1336 | *Paenibacillus* sp. 32O-W | 0 | 0.003329 | 0.002379 | Bacteria |
| 1337 | *Paenibacillus* sp. FJAT-26967 | 0 | 0.003329 | 0.002379 | Bacteria |
| 1338 | *Paenibacillus* sp. RIFOXYA1_FULL_44_5 | 0 | 0.003329 | 0.002379 | Bacteria |
| 1339 | *Paenibacillus* sp. SIT18 | 0 | 0.003329 | 0.002379 | Bacteria |
| 1340 | *Paenibacillus stellifer* | 0 | 0.003329 | 0.002379 | Bacteria |
| 1341 | *Paenibacillus taiwanensis* | 0 | 0.003329 | 0.002379 | Bacteria |
| 1342 | *Paenibacillus wulumuqiensis* | 0 | 0.003329 | 0.002379 | Bacteria |
| 1343 | *Phormidesmis priestleyi* | 0 | 0.003329 | 0.002379 | Bacteria |
| 1344 | *Polaromonas* sp. JS666 | 0 | 0.003329 | 0.002379 | Bacteria |
| 1345 | *Pseudomonas indica* | 0 | 0.003329 | 0.002379 | Bacteria |
| 1346 | *Pseudomonas xanthomarina* | 0 | 0.003329 | 0.002379 | Bacteria |
| 1347 | *Rhodoferax antarcticus* | 0 | 0.003329 | 0.002379 | Bacteria |
| 1348 | *Rhodovibrio salinarum* | 0 | 0.003329 | 0.002379 | Bacteria |
| 1349 | *Rubrivivax gelatinosus* | 0 | 0.003329 | 0.002379 | Bacteria |
| 1350 | *Rubrivivax* sp. SCN 70-15 | 0 | 0.003329 | 0.002379 | Bacteria |
| 1351 | *Salipaludibacillus aurantiacus* | 0 | 0.003329 | 0.002379 | Bacteria |
| 1352 | *Saprospira grandis* | 0 | 0.003329 | 0.002379 | Bacteria |
| 1353 | *Sediminibacillus albus* | 0 | 0.003329 | 0.002379 | Bacteria |
| 1354 | *Tepidanaerobacter syntrophicus* | 0 | 0.003329 | 0.002379 | Bacteria |
| 1355 | *Thauera* sp. ZV-1-C | 0 | 0.003329 | 0.002379 | Bacteria |
| 1356 | *Vaginella massiliensis* | 0 | 0.003329 | 0.002379 | Bacteria |
| 1357 | *Variovorax* sp. YR216 | 0 | 0.003329 | 0.002379 | Bacteria |
| 1358 | *Thalassiosira oceanica* | 0 | 0.003329 | 0.002379 | Eukaryota |
| 1359 | *Achromobacter* sp. DMS1 | 0 | 0 | 0.004759 | Bacteria |
| 1360 | *Acidovorax* sp. 12322-1 | 0 | 0 | 0.004759 | Bacteria |
| 1361 | *Acinetobacter boissieri* | 0 | 0 | 0.004759 | Bacteria |
| 1362 | *Acinetobacter celticus* | 0 | 0 | 0.004759 | Bacteria |
| 1363 | *Acinetobacter haemolyticus* | 0 | 0 | 0.004759 | Bacteria |
| 1364 | *Acinetobacter* sp. 1294596 | 0 | 0 | 0.004759 | Bacteria |
| 1365 | *Acinetobacter* sp. CIP 101966 | 0 | 0 | 0.004759 | Bacteria |
| 1366 | *Acinetobacter* sp. CIP 102136 | 0 | 0 | 0.004759 | Bacteria |
| 1367 | *Acinetobacter* sp. CIP 51.11 | 0 | 0 | 0.004759 | Bacteria |
| 1368 | *Acinetobacter* sp. MDS7A | 0 | 0 | 0.004759 | Bacteria |
| 1369 | *Acinetobacter* sp. SFA | 0 | 0 | 0.004759 | Bacteria |
| 1370 | *Acinetobacter* sp. TTH0-4 | 0 | 0 | 0.004759 | Bacteria |
| 1371 | *Aggregatibacter actinomycetemcomitans* | 0 | 0 | 0.004759 | Bacteria |
| 1372 | *Algiphilus aromaticivorans* | 0 | 0 | 0.004759 | Bacteria |
| 1373 | *Alicyclobacillus herbarius* | 0 | 0 | 0.004759 | Bacteria |
| 1374 | *Amphibacillus marinus* | 0 | 0 | 0.004759 | Bacteria |
| 1375 | *Anaerobacillus macyae* | 0 | 0 | 0.004759 | Bacteria |
| 1376 | *Arthrobacter halophytocola* | 0 | 0 | 0.004759 | Bacteria |
| 1377 | *Aurantimonas* sp. 22II-16-19i | 0 | 0 | 0.004759 | Bacteria |
| 1378 | *Azohydromonas lata* | 0 | 0 | 0.004759 | Bacteria |
| 1379 | *Bacillus rhizosphaerae* | 0 | 0 | 0.004759 | Bacteria |
| 1380 | *Bacillus* sp. 7_6_55CFAA_CT2 | 0 | 0 | 0.004759 | Bacteria |
| 1381 | *Bacillus* sp. G1(2015b) | 0 | 0 | 0.004759 | Bacteria |
| 1382 | *Bacillus* sp. Leaf75 | 0 | 0 | 0.004759 | Bacteria |
| 1383 | *Bacillus* sp. NH24A2 | 0 | 0 | 0.004759 | Bacteria |
| 1384 | *Bacillus* sp. RUTrin4 | 0 | 0 | 0.004759 | Bacteria |
| 1385 | *Bacillus* sp. WP8 | 0 | 0 | 0.004759 | Bacteria |
| 1386 | *Basilea psittacipulmonis* | 0 | 0 | 0.004759 | Bacteria |
| 1387 | *Bordetella avium* | 0 | 0 | 0.004759 | Bacteria |
| 1388 | *Bordetella* genomosp. 13 | 0 | 0 | 0.004759 | Bacteria |
| 1389 | *Bordetella petrii* | 0 | 0 | 0.004759 | Bacteria |
| 1390 | *Brochothrix campestris* | 0 | 0 | 0.004759 | Bacteria |
| 1391 | *Brucella suis* | 0 | 0 | 0.004759 | Bacteria |
| 1392 | *Calditerricola satsumensis* | 0 | 0 | 0.004759 | Bacteria |
| 1393 | *Capnocytophaga cynodegmi* | 0 | 0 | 0.004759 | Bacteria |
| 1394 | *Citrobacter freundii* | 0 | 0 | 0.004759 | Bacteria |
| 1395 | *Clostridium* sp. CL-2 | 0 | 0 | 0.004759 | Bacteria |
| 1396 | *Clostridium* sp. Maddingley MBC34-26 | 0 | 0 | 0.004759 | Bacteria |
| 1397 | *Corynebacterium camporealensis* | 0 | 0 | 0.004759 | Bacteria |
| 1398 | *Corynebacterium capitovis* | 0 | 0 | 0.004759 | Bacteria |
| 1399 | *Corynebacterium kroppenstedtii* | 0 | 0 | 0.004759 | Bacteria |
| 1400 | *Corynebacterium lubricantis* | 0 | 0 | 0.004759 | Bacteria |
| 1401 | *Corynebacterium phocae* | 0 | 0 | 0.004759 | Bacteria |
| 1402 | *Desulfobulbus elongatus* | 0 | 0 | 0.004759 | Bacteria |
| 1403 | *Desulfonatronum thiodismutans* | 0 | 0 | 0.004759 | Bacteria |
| 1404 | *Dethiobacter alkaliphilus* | 0 | 0 | 0.004759 | Bacteria |
| 1405 | *Duganella phyllosphaerae* | 0 | 0 | 0.004759 | Bacteria |
| 1406 | *Emticicia oligotrophica* | 0 | 0 | 0.004759 | Bacteria |
| 1407 | *Enterobacter cancerogenus* | 0 | 0 | 0.004759 | Bacteria |
| 1408 | *Enterococcus haemoperoxidus* | 0 | 0 | 0.004759 | Bacteria |
| 1409 | *Enterococcus* sp. HMSC076E04 | 0 | 0 | 0.004759 | Bacteria |
| 1410 | *Eubacterium* sp. ER2 | 0 | 0 | 0.004759 | Bacteria |
| 1411 | *Flavobacterium terrigena* | 0 | 0 | 0.004759 | Bacteria |
| 1412 | *Geomicrobium* sp. JCM 19038 | 0 | 0 | 0.004759 | Bacteria |
| 1413 | *Geomicrobium* sp. JCM 19055 | 0 | 0 | 0.004759 | Bacteria |
| 1414 | *Granulicatella balaenopterae* | 0 | 0 | 0.004759 | Bacteria |
| 1415 | *Halonatronum saccharophilum* | 0 | 0 | 0.004759 | Bacteria |
| 1416 | *Herminiimonas arsenicoxydans* | 0 | 0 | 0.004759 | Bacteria |
| 1417 | *Immundisolibacter cernigliae* | 0 | 0 | 0.004759 | Bacteria |
| 1418 | *Intestinibacter bartlettii* | 0 | 0 | 0.004759 | Bacteria |
| 1419 | *Janthinobacterium lividum* | 0 | 0 | 0.004759 | Bacteria |
| 1420 | *Klebsiella* sp. RIT-PI-d | 0 | 0 | 0.004759 | Bacteria |
| 1421 | *Kluyvera cryocrescens* | 0 | 0 | 0.004759 | Bacteria |
| 1422 | *Lacinutrix* sp. Hel_I_90 | 0 | 0 | 0.004759 | Bacteria |
| 1423 | *Lactobacillus acetotolerans* | 0 | 0 | 0.004759 | Bacteria |
| 1424 | *Lactobacillus fuchuensis* | 0 | 0 | 0.004759 | Bacteria |
| 1425 | *Lactobacillus harbinensis* | 0 | 0 | 0.004759 | Bacteria |
| 1426 | *Lactobacillus ingluviei* | 0 | 0 | 0.004759 | Bacteria |
| 1427 | *Lactobacillus johnsonii* | 0 | 0 | 0.004759 | Bacteria |
| 1428 | *Lactobacillus* sp. HMSC08B12 | 0 | 0 | 0.004759 | Bacteria |
| 1429 | *Lactobacillus* sp. HMSC24D01 | 0 | 0 | 0.004759 | Bacteria |
| 1430 | *Laribacter hongkongensis* | 0 | 0 | 0.004759 | Bacteria |
| 1431 | *Leucothrix mucor* | 0 | 0 | 0.004759 | Bacteria |
| 1432 | *Listeria floridensis* | 0 | 0 | 0.004759 | Bacteria |
| 1433 | *Mageeibacillus indolicus* | 0 | 0 | 0.004759 | Bacteria |
| 1434 | *Mycobacterium kansasii* | 0 | 0 | 0.004759 | Bacteria |
| 1435 | *Neglecta timonensis* | 0 | 0 | 0.004759 | Bacteria |
| 1436 | *Neisseria arctica* | 0 | 0 | 0.004759 | Bacteria |
| 1437 | *Oceaniovalibus guishaninsula* | 0 | 0 | 0.004759 | Bacteria |
| 1438 | *Oceanospirillum beijerinckii* | 0 | 0 | 0.004759 | Bacteria |
| 1439 | *Paenibacillus catalpae* | 0 | 0 | 0.004759 | Bacteria |
| 1440 | *Paenibacillus* sp. A9 | 0 | 0 | 0.004759 | Bacteria |
| 1441 | *Paenibacillus* sp. CF384 | 0 | 0 | 0.004759 | Bacteria |
| 1442 | *Paenibacillus* sp. HW567 | 0 | 0 | 0.004759 | Bacteria |
| 1443 | *Paenibacillus* sp. Soil750 | 0 | 0 | 0.004759 | Bacteria |
| 1444 | *Parapedobacter luteus* | 0 | 0 | 0.004759 | Bacteria |
| 1445 | *Peptoniphilus indolicus* | 0 | 0 | 0.004759 | Bacteria |
| 1446 | *Peptoniphilus* sp. oral taxon 375 | 0 | 0 | 0.004759 | Bacteria |
| 1447 | *Phaseolibacter flectens* | 0 | 0 | 0.004759 | Bacteria |
| 1448 | *Pilibacter termitis* | 0 | 0 | 0.004759 | Bacteria |
| 1449 | *Pluralibacter gergoviae* | 0 | 0 | 0.004759 | Bacteria |
| 1450 | *Proteus* sp. HMSC10D02 | 0 | 0 | 0.004759 | Bacteria |
| 1451 | *Pseudomonas resinovorans* | 0 | 0 | 0.004759 | Bacteria |
| 1452 | *Pseudonocardia spinosispora* | 0 | 0 | 0.004759 | Bacteria |
| 1453 | *Pseudospirillum japonicum* | 0 | 0 | 0.004759 | Bacteria |
| 1454 | *Psychrobacillus psychrodurans* | 0 | 0 | 0.004759 | Bacteria |
| 1455 | *Psychrobacter* sp. AntiMn-1 | 0 | 0 | 0.004759 | Bacteria |
| 1456 | *Rhodovulum* sp. ES.010 | 0 | 0 | 0.004759 | Bacteria |
| 1457 | *Rubellimicrobium thermophilum* | 0 | 0 | 0.004759 | Bacteria |
| 1458 | *Ruminiclostridium thermocellum* | 0 | 0 | 0.004759 | Bacteria |
| 1459 | *Salegentibacter mishustinae* | 0 | 0 | 0.004759 | Bacteria |
| 1460 | *Salimicrobium album* | 0 | 0 | 0.004759 | Bacteria |
| 1461 | *Salinimicrobium xinjiangense* | 0 | 0 | 0.004759 | Bacteria |
| 1462 | *Salsuginibacillus kocurii* | 0 | 0 | 0.004759 | Bacteria |
| 1463 | *Skermanella stibiiresistens* | 0 | 0 | 0.004759 | Bacteria |
| 1464 | *Sphingobacterium thalpophilum* | 0 | 0 | 0.004759 | Bacteria |
| 1465 | *Sphingopyxis flava* | 0 | 0 | 0.004759 | Bacteria |
| 1466 | *Staphylococcus capitis* | 0 | 0 | 0.004759 | Bacteria |
| 1467 | *Streptococcus mitis* | 0 | 0 | 0.004759 | Bacteria |
| 1468 | *Sulfurifustis variabilis* | 0 | 0 | 0.004759 | Bacteria |
| 1469 | *Thauera butanivorans* | 0 | 0 | 0.004759 | Bacteria |
| 1470 | *Thermoanaerobacterium thermosaccharolyticum* | 0 | 0 | 0.004759 | Bacteria |
| 1471 | *Thermotalea metallivorans* | 0 | 0 | 0.004759 | Bacteria |
| 1472 | *Variovorax* sp. CF313 | 0 | 0 | 0.004759 | Bacteria |
| 1473 | *Vitreoscilla stercoraria* | 0 | 0 | 0.004759 | Bacteria |
| 1474 | *Xanthomonas citri* | 0 | 0 | 0.004759 | Bacteria |
| 1475 | *Zavarzinella formosa* | 0 | 0 | 0.004759 | Bacteria |
| 1476 | *Neospora caninum* | 0 | 0 | 0.004759 | Eukaryota |
| 1477 | *Methanocella paludicola* | 0.004117 | 0 | 0 | Archaea |
| 1478 | *Methanolinea tarda* | 0.004117 | 0 | 0 | Archaea |
| 1479 | *Acetabularia acetabulum* | 0.004117 | 0 | 0 | Eukaryota |
| 1480 | *Acidomyces richmondensis* | 0.004117 | 0 | 0 | Eukaryota |
| 1481 | *Candida albicans* | 0.004117 | 0 | 0 | Eukaryota |
| 1482 | *Candida maltosa* | 0.004117 | 0 | 0 | Eukaryota |
| 1483 | *Candida orthopsilosis* | 0.004117 | 0 | 0 | Eukaryota |
| 1484 | *Candida sake* | 0.004117 | 0 | 0 | Eukaryota |
| 1485 | *Candida subhashii* | 0.004117 | 0 | 0 | Eukaryota |
| 1486 | *Chlorella variabilis* | 0.004117 | 0 | 0 | Eukaryota |
| 1487 | *Cyberlindnera jadinii* | 0.004117 | 0 | 0 | Eukaryota |
| 1488 | *Debaryomyces fabryi* | 0.004117 | 0 | 0 | Eukaryota |
| 1489 | *Exophiala aquamarina* | 0.004117 | 0 | 0 | Eukaryota |
| 1490 | *Fragilariopsis cylindrus* | 0.004117 | 0 | 0 | Eukaryota |
| 1491 | *Galdieria sulphuraria* | 0.004117 | 0 | 0 | Eukaryota |
| 1492 | *Hypsizygus marmoreus* | 0.004117 | 0 | 0 | Eukaryota |
| 1493 | *Leucosporidium creatinivorum* | 0.004117 | 0 | 0 | Eukaryota |
| 1494 | *Lichtheimia ramosa* | 0.004117 | 0 | 0 | Eukaryota |
| 1495 | *Metschnikowia bicuspidata* | 0.004117 | 0 | 0 | Eukaryota |
| 1496 | *Mucor circinelloides* | 0.004117 | 0 | 0 | Eukaryota |
| 1497 | *Nannochloropsis gaditana* | 0.004117 | 0 | 0 | Eukaryota |
| 1498 | *Plasmodiophora brassicae* | 0.004117 | 0 | 0 | Eukaryota |
| 1499 | *Pyronema omphalodes* | 0.004117 | 0 | 0 | Eukaryota |
| 1500 | *Spathaspora passalidarum* | 0.004117 | 0 | 0 | Eukaryota |
| 1501 | *Stentor coeruleus* | 0.004117 | 0 | 0 | Eukaryota |
| 1502 | *Tetrahymena thermophila* | 0.004117 | 0 | 0 | Eukaryota |
| 1503 | *Cellulophaga* phage phi18:1 | 0.004117 | 0 | 0 | Viruses |
| 1504 | *Enterococcus* phage IME-EFm5 | 0.004117 | 0 | 0 | Viruses |
| 1505 | *Mycobacterium* phage Idleandcovert | 0.004117 | 0 | 0 | Viruses |
| 1506 | *Pseudoalteromonas* phage H105/1 | 0.004117 | 0 | 0 | Viruses |
| 1507 | *Streptococcus* phage C1 | 0.004117 | 0 | 0 | Viruses |
| 1508 | *Staphylococcus* phage Stau2 | 0.004117 | 0 | 0 | Viruses |
| 1509 | *Bacillus* phage BCJA1c | 0.004117 | 0 | 0 | Viruses |
| 1510 | *Bacillus* phage Eldridge | 0.004117 | 0 | 0 | Viruses |
| 1511 | *Bacillus* phage BCP8-2 | 0.004117 | 0 | 0 | Viruses |
| 1512 | *Geobacillus* virus E2 | 0.004117 | 0 | 0 | Viruses |
| 1513 | *Haloterrigena thermotolerans* | 0 | 0.003329 | 0 | Archaea |
| 1514 | *Methanocella arvoryzae* | 0 | 0.003329 | 0 | Archaea |
| 1515 | *Methanosaeta harundinacea* | 0 | 0.003329 | 0 | Archaea |
| 1516 | *Methanosalsum zhilinae* | 0 | 0.003329 | 0 | Archaea |
| 1517 | *Pyrococcus furiosus* | 0 | 0.003329 | 0 | Archaea |
| 1518 | *Conidiobolus coronatus* | 0 | 0.003329 | 0 | Eukaryota |
| 1519 | *Hirsutella minnesotensis* | 0 | 0.003329 | 0 | Eukaryota |
| 1520 | *Lichtheimia corymbifera* | 0 | 0.003329 | 0 | Eukaryota |
| 1521 | *Puccinia striiformis* | 0 | 0.003329 | 0 | Eukaryota |
| 1522 | *Rhodotorula graminis* | 0 | 0.003329 | 0 | Eukaryota |
| 1523 | *Sclerotinia sclerotiorum* | 0 | 0.003329 | 0 | Eukaryota |
| 1524 | *Trypanosoma brucei* | 0 | 0.003329 | 0 | Eukaryota |
| 1525 | *Bacillus* phage QCM8 | 0 | 0.003329 | 0 | Viruses |
| 1526 | *Enterococcus* phage EFDG1 | 0 | 0.003329 | 0 | Viruses |
| 1527 | *Haloprofundus marisrubri* | 0 | 0 | 0.002379 | Archaea |
| 1528 | *Methanoregula formicica* | 0 | 0 | 0.002379 | Archaea |
| 1529 | *Thermoproteus* sp. AZ2 | 0 | 0 | 0.002379 | Archaea |
| 1530 | *Aspergillus carbonarius* | 0 | 0 | 0.002379 | Eukaryota |
| 1531 | *Asterionella formosa* | 0 | 0 | 0.002379 | Eukaryota |
| 1532 | *Ectocarpus siliculosus* | 0 | 0 | 0.002379 | Eukaryota |
| 1533 | *Emiliania huxleyi* | 0 | 0 | 0.002379 | Eukaryota |
| 1534 | *Parasitella parasitica* | 0 | 0 | 0.002379 | Eukaryota |
| 1535 | *Pseudocohnilembus persalinus* | 0 | 0 | 0.002379 | Eukaryota |
| 1536 | *Thalassiosira pseudonana* | 0 | 0 | 0.002379 | Eukaryota |
| 1537 | *Trypanosoma theileri* | 0 | 0 | 0.002379 | Eukaryota |
| 1538 | *Yarrowia lipolytica* | 0 | 0 | 0.002379 | Eukaryota |
| 1539 | *Bacillus* phage 0305phi8-36 | 0 | 0 | 0.002379 | Viruses |
| 1540 | *Bacillus* phage BCD7 | 0 | 0 | 0.002379 | Viruses |
| 1541 | *Bacillus* phage PBC1 | 0 | 0 | 0.002379 | Viruses |
| 1542 | *Bacillus* virus 1 | 0 | 0 | 0.002379 | Viruses |
| 1543 | *Bacillus* virus Andromeda | 0 | 0 | 0.002379 | Viruses |
| 1544 | *Bacillus* virus G | 0 | 0 | 0.002379 | Viruses |
| 1545 | *Bacillus* phage Stahl | 0 | 0 | 0.002379 | Viruses |
| 1546 | *Lactobacillus* phage phiPYB5 | 0 | 0 | 0.002379 | Viruses |
| 1547 | *Pseudoalteromonas* phage vB_PspS-H40/1 | 0 | 0 | 0.002379 | Viruses |
| 1548 | *Vibrio* phage ICP1 | 0 | 0 | 0.002379 | Viruses |
| 1549 | unclassified bacterial species | 1.965269 | 2.85495 | 7.098131 | Bacteria |
| 1550 | unclassified eukaryotic species | 0.032939 | 0.026631 | 0.03569 | Eukaryota |
| 1551 | unclassified archaeal species | 0.004117 | 0.003329 | 0 | Archaea |
| 1552 | unclassified viral species | 0.004117 | 0.009987 | 0 | Viruses |

| **Supplementary Table 6: *Bacillus* species detected in *kinema* metagenome.** | | | | |
| --- | --- | --- | --- | --- |
| **Sl. No.** | **Species** | **Relative Abundance (%)** | | |
|  |  | ***Kinema* (India)** | ***Kinema* (Nepal)** | ***Kinema* (Bhutan)** |
| 1 | *Bacillus subtilis* | 29.57138 | 29.04461 | 14.26634 |
| 2 | *Bacillus glycinifermentans* | 13.52987 | 18.69174 | 6.604963 |
| 3 | *Bacillus cereus* | 0.378803 | 0.599201 | 18.83937 |
| 4 | *Bacillus licheniformis* | 7.724297 | 4.324234 | 0.354517 |
| 5 | *Bacillus thermoamylovorans* | 3.722156 | 2.480027 | 2.643413 |
| 6 | *Bacillus coagulans* | 0.757607 | 2.083888 | 2.664827 |
| 7 | *Bacillus circulans* | 0.14411 | 0.109854 | 4.948964 |
| 8 | *Bacillus paralicheniformis* | 4.331535 | 0.316245 | 0.059483 |
| 9 | *Bacillus amyloliquefaciens* | 2.095771 | 0.559254 | 1.301482 |
| 10 | *Bacillus andreraoultii* | 1.00877 | 1.331558 | 0.285517 |
| 11 | *Bacillus thuringiensis* | 0.069996 | 0.05992 | 1.082586 |
| 12 | *Bacillus eiseniae* | 0.12764 | 0.319574 | 0.701896 |
| 13 | *Bacillus stratosphericus* | 0.275868 | 0.466045 | 0.406862 |
| 14 | *Bacillus* sp. MSP13 | 0.345864 | 0.69241 | 0.023793 |
| 15 | *Bacillus sonorensis* | 0.395273 | 0.329561 | 0.099931 |
| 16 | *Bacillus alveayuensis* | 0.037057 | 0.699068 | 0.03331 |
| 17 | *Bacillus pumilus* | 0.107053 | 0.139814 | 0.430655 |
| 18 | *Bacillus smithii* | 0.115288 | 0.476032 | 0.064241 |
| 19 | *Bacillus anthracis* | 0.024705 | 0.019973 | 0.523448 |
| 20 | *Bacillus velezensis* | 0.111171 | 0.286285 | 0.149897 |
| 21 | *Bacillus sporothermodurans* | 0.279985 | 0.216378 | 0.021414 |
| 22 | *Bacillus fordii* | 0.16058 | 0.229694 | 0.047586 |
| 23 | *Bacillus nealsonii* | 0.028822 | 0.02996 | 0.321207 |
| 24 | *Bacillus clausii* | 0.16058 | 0.073236 | 0.145138 |
| 25 | *Bacillus* sp. BT1B_CT2 | 0.197637 | 0.096538 | 0.026172 |
| 26 | *Bacillus cytotoxicus* | 0.218224 | 0.056591 | 0.03569 |
| 27 | *Bacillus nakamurai* | 0.098818 | 0.176431 | 0.023793 |
| 28 | *Bacillus* sp. LM 4-2 | 0.028822 | 0.166445 | 0.090414 |
| 29 | *Bacillus azotoformans* | 0.123523 | 0.123169 | 0.038069 |
| 30 | *Bacillus mycoides* | 0.020587 | 0.009987 | 0.252207 |
| 31 | *Bacillus tequilensis* | 0.074114 | 0.079893 | 0.121345 |
| 32 | *Bacillus methanolicus* | 0.045292 | 0.136485 | 0.069 |
| 33 | *Bacillus* sp. VT-16-64 | 0.078231 | 0.11984 | 0.049966 |
| 34 | *Bacillus acidiproducens* | 0.024705 | 0.109854 | 0.083276 |
| 35 | *Bacillus* sp. NRRL B-41327 | 0.172932 | 0.039947 | 0.002379 |
| 36 | *Bacillus* sp. EGD-AK10 | 0.041174 | 0.083222 | 0.090414 |
| 37 | *Bacillus pseudofirmus* | 0.028822 | 0.159787 | 0.009517 |
| 38 | *Bacillus* sp. Marseille-P2366 | 0.057644 | 0.086551 | 0.030931 |
| 39 | *Bacillus massiliosenegalensis* | 0.028822 | 0.109854 | 0.028552 |
| 40 | *Bacillus farraginis* | 0.024705 | 0.08988 | 0.052345 |
| 41 | *Bacillus niacini* | 0.057644 | 0.096538 | 0.011897 |
| 42 | *Bacillus acidicola* | 0.037057 | 0.113182 | 0.014276 |
| 43 | *Bacillus* sp. EB01 | 0.053527 | 0.103196 | 0.007138 |
| 44 | *Bacillus* sp. Marseille-P2384 | 0.020587 | 0.099867 | 0.03331 |
| 45 | *Bacillus solani* | 0.037057 | 0.099867 | 0.016655 |
| 46 | *Bacillus* sp. X1(2014) | 0.024705 | 0.116511 | 0.011897 |
| 47 | *Bacillus* sp. FMQ74 | 0.053527 | 0.039947 | 0.052345 |
| 48 | *Bacillus oceanisediminis* | 0.053527 | 0.066578 | 0.019034 |
| 49 | *Bacillus shackletonii* | 0.049409 | 0.063249 | 0.021414 |
| 50 | *Bacillus* sp. KCTC 13219 | 0.032939 | 0.069907 | 0.028552 |
| 51 | *Bacillus* sp. CC120222-01 | 0.037057 | 0.046605 | 0.042828 |
| 52 | *Bacillus mojavensis* | 0.057644 | 0.036618 | 0.030931 |
| 53 | *Bacillus* sp. FJAT-27225 | 0.065879 | 0.046605 | 0.011897 |
| 54 | *Bacillus galactosidilyticus* | 0.008235 | 0.053262 | 0.061862 |
| 55 | *Bacillus* sp. MB2021 | 0.041174 | 0.066578 | 0.014276 |
| 56 | *Bacillus dielmoensis* | 0.024705 | 0.069907 | 0.019034 |
| 57 | *Bacillus agaradhaerens* | 0.028822 | 0.069907 | 0.014276 |
| 58 | *Bacillus vallismortis* | 0.037057 | 0.023302 | 0.052345 |
| 59 | *Bacillus safensis* | 0.020587 | 0.013316 | 0.078517 |
| 60 | *Bacillus* sp. MUM 116 | 0.01647 | 0.083222 | 0.009517 |
| 61 | *Bacillus* sp. B14905 | 0.049409 | 0.019973 | 0.038069 |
| 62 | *Bacillus fumarioli* | 0.032939 | 0.05992 | 0.014276 |
| 63 | *Bacillus* sp. SB47 | 0.074114 | 0.023302 | 0.009517 |
| 64 | *Bacillus* sp. FJAT-29814 | 0 | 0.053262 | 0.049966 |
| 65 | *Bacillus* sp. B-jedd | 0.024705 | 0.049933 | 0.028552 |
| 66 | *Bacillus rubiinfantis* | 0.037057 | 0.056591 | 0.009517 |
| 67 | *Bacillus niameyensis* | 0.037057 | 0.046605 | 0.016655 |
| 68 | *Bacillus* sp. OxB-1 | 0.020587 | 0.019973 | 0.059483 |
| 69 | *Bacillus* sp. TH008 | 0.069996 | 0.013316 | 0.016655 |
| 70 | *Bacillus* sp. A053 | 0.012352 | 0.05992 | 0.023793 |
| 71 | *Bacillus timonensis* | 0.020587 | 0.036618 | 0.038069 |
| 72 | *Bacillus altitudinis* | 0 | 0 | 0.095172 |
| 73 | *Bacillus* sp. JS | 0.012352 | 0.046605 | 0.03569 |
| 74 | *Bacillus marisflavi* | 0.032939 | 0.023302 | 0.03569 |
| 75 | *Bacillus xiamenensis* | 0.028822 | 0.013316 | 0.045207 |
| 76 | *Bacillus gobiensis* | 0.037057 | 0.02996 | 0.016655 |
| 77 | *Bacillus megaterium* | 0.020587 | 0.039947 | 0.021414 |
| 78 | *Bacillus* sp. CMAA 1185 | 0.01647 | 0.043276 | 0.021414 |
| 79 | *Bacillus panaciterrae* | 0 | 0.05992 | 0.014276 |
| 80 | *Bacillus massilioanorexius* | 0.028822 | 0.033289 | 0.011897 |
| 81 | *Bacillus weihenstephanensis* | 0.008235 | 0.006658 | 0.057103 |
| 82 | *Bacillus ginsengihumi* | 0.020587 | 0.02996 | 0.021414 |
| 83 | *Bacillus wiedmannii* | 0 | 0.009987 | 0.061862 |
| 84 | *Bacillus vietnamensis* | 0.037057 | 0.023302 | 0.009517 |
| 85 | *Bacillus cecembensis* | 0.020587 | 0.02996 | 0.014276 |
| 86 | *Bacillus tuaregi* | 0.012352 | 0.036618 | 0.014276 |
| 87 | *Bacillus* sp. NSP9.1 | 0.037057 | 0.016644 | 0.009517 |
| 88 | *Bacillus firmus* | 0 | 0.019973 | 0.040448 |
| 89 | *Bacillus cihuensis* | 0.024705 | 0.033289 | 0.002379 |
| 90 | *Bacillus gaemokensis* | 0.01647 | 0.019973 | 0.023793 |
| 91 | *Bacillus* sp. FJAT-27997 | 0.01647 | 0.036618 | 0.007138 |
| 92 | *Bacillus flexus* | 0.020587 | 0.019973 | 0.016655 |
| 93 | *Bacillus badius* | 0.028822 | 0.019973 | 0.007138 |
| 94 | *Bacillus humi* | 0.024705 | 0.016644 | 0.014276 |
| 95 | *Bacillus cohnii* | 0.020587 | 0.02996 | 0.004759 |
| 96 | *Bacillus* sp. MKU004 | 0 | 0.049933 | 0.004759 |
| 97 | *Bacillus mesonae* | 0.01647 | 0.026631 | 0.009517 |
| 98 | *Bacillus* sp. FJAT-44921 | 0.004117 | 0.043276 | 0.004759 |
| 99 | *Bacillus* sp. 1310(2010) | 0.028822 | 0.023302 | 0 |
| 100 | *Bacillus* sp. CPSM8 | 0.045292 | 0.006658 | 0 |
| 101 | *Bacillus aquimaris* | 0.004117 | 0.02996 | 0.016655 |
| 102 | *Bacillus dakarensis* | 0.008235 | 0.023302 | 0.019034 |
| 103 | *Bacillus* sp. RUPDJ | 0.020587 | 0.02996 | 0 |
| 104 | *Bacillus* sp. MN5 | 0 | 0 | 0.049966 |
| 105 | *Bacillus* sp. FJAT-27445 | 0.004117 | 0.019973 | 0.023793 |
| 106 | *Bacillus korlensis* | 0.020587 | 0.019973 | 0.007138 |
| 107 | *Bacillus atrophaeus* | 0.012352 | 0.013316 | 0.021414 |
| 108 | *Bacillus koreensis* | 0.01647 | 0.023302 | 0.007138 |
| 109 | *Bacillus* sp. F56 | 0.01647 | 0.019973 | 0.009517 |
| 110 | *Bacillus coahuilensis* | 0.008235 | 0.02996 | 0.007138 |
| 111 | *Bacillus fastidiosus* | 0.008235 | 0.026631 | 0.009517 |
| 112 | *Bacillus simplex* | 0.004117 | 0.013316 | 0.026172 |
| 113 | *Bacillus horikoshii* | 0.020587 | 0.013316 | 0.009517 |
| 114 | *Bacillus soli* | 0.012352 | 0.016644 | 0.014276 |
| 115 | *Bacillus* sp. GeD10 | 0.004117 | 0 | 0.038069 |
| 116 | *Bacillus psychrosaccharolyticus* | 0.004117 | 0.013316 | 0.023793 |
| 117 | *Bacillus zhangzhouensis* | 0.004117 | 0 | 0.03569 |
| 118 | *Bacillus gottheilii* | 0.008235 | 0.016644 | 0.014276 |
| 119 | *Bacillus lentus* | 0.008235 | 0.016644 | 0.014276 |
| 120 | *Bacillus trypoxylicola* | 0.012352 | 0.026631 | 0 |
| 121 | *Bacillus litoralis* | 0 | 0.026631 | 0.011897 |
| 122 | *Bacillus clarkii* | 0.012352 | 0.016644 | 0.009517 |
| 123 | *Bacillus* sp. LF1 | 0.008235 | 0.019973 | 0.009517 |
| 124 | *Bacillus* sp. NSP2.1 | 0 | 0.013316 | 0.023793 |
| 125 | *Bacillus ligniniphilus* | 0.004117 | 0.019973 | 0.011897 |
| 126 | *Bacillus* sp. SDLI1 | 0.008235 | 0.013316 | 0.014276 |
| 127 | *Bacillus* sp. N24 | 0 | 0 | 0.03569 |
| 128 | *Bacillus obstructivus* | 0.004117 | 0.026631 | 0.004759 |
| 129 | *Bacillus* sp. MRMR6 | 0.008235 | 0.019973 | 0.007138 |
| 130 | *Bacillus marmarensis* | 0.032939 | 0 | 0.002379 |
| 131 | *Bacillus* sp. SG-1 | 0.024705 | 0.003329 | 0.007138 |
| 132 | *Bacillus caseinilyticus* | 0.004117 | 0.016644 | 0.014276 |
| 133 | *Bacillus* sp. FJAT-14578 | 0.004117 | 0.023302 | 0.007138 |
| 134 | *Bacillus krulwichiae* | 0.008235 | 0.023302 | 0.002379 |
| 135 | *Bacillus* sp. 2_A_57_CT2 | 0 | 0.026631 | 0.007138 |
| 136 | *Bacillus* sp. 5B6 | 0 | 0.026631 | 0.007138 |
| 137 | *Bacillus horneckiae* | 0.004117 | 0.019973 | 0.009517 |
| 138 | *Bacillus* sp. ok061 | 0 | 0 | 0.03331 |
| 139 | *Bacillus* sp. J37 | 0.008235 | 0.003329 | 0.021414 |
| 140 | *Bacillus bogoriensis* | 0.012352 | 0.013316 | 0.007138 |
| 141 | *Bacillus alkalitelluris* | 0.004117 | 0.016644 | 0.011897 |
| 142 | *Bacillus* sp. SA1-12 | 0.004117 | 0.013316 | 0.014276 |
| 143 | *Bacillus* sp. J33 | 0.020587 | 0.003329 | 0.007138 |
| 144 | *Bacillus* sp. GZT | 0 | 0 | 0.030931 |
| 145 | *Bacillus* sp. UNC41MFS5 | 0.012352 | 0.006658 | 0.011897 |
| 146 | *Bacillus massiliogorillae* | 0 | 0.013316 | 0.016655 |
| 147 | *Bacillus* sp. FJAT-18017 | 0.012352 | 0.003329 | 0.014276 |
| 148 | *Bacillus bataviensis* | 0.004117 | 0.023302 | 0.002379 |
| 149 | *Bacillus aidingensis* | 0.008235 | 0.016644 | 0.004759 |
| 150 | *Bacillus persicus* | 0.008235 | 0.016644 | 0.004759 |
| 151 | *Bacillus salsus* | 0.008235 | 0.016644 | 0.004759 |
| 152 | *Bacillus* sp. FJAT-22058 | 0.01647 | 0.009987 | 0.002379 |
| 153 | *Bacillus* sp. 1NLA3E | 0.012352 | 0.006658 | 0.009517 |
| 154 | *Bacillus pseudomycoides* | 0 | 0.023302 | 0.004759 |
| 155 | *Bacillus oryziterrae* | 0.004117 | 0.016644 | 0.007138 |
| 156 | *Bacillus intestinalis* | 0.01647 | 0.006658 | 0.004759 |
| 157 | *Bacillus kribbensis* | 0 | 0.013316 | 0.014276 |
| 158 | *Bacillus bingmayongensis* | 0.004117 | 0.006658 | 0.016655 |
| 159 | *Bacillus* sp. UNC125MFCrub1.1 | 0.004117 | 0.006658 | 0.016655 |
| 160 | *Bacillus* sp. CDB3 | 0 | 0.003329 | 0.023793 |
| 161 | *Bacillus* sp. SJS | 0 | 0.019973 | 0.007138 |
| 162 | *Bacillus lonarensis* | 0.012352 | 0.009987 | 0.004759 |
| 163 | *Bacillus* sp. Leaf49 | 0 | 0 | 0.026172 |
| 164 | *Bacillus* sp. UNC437CL72CviS29 | 0.004117 | 0.016644 | 0.004759 |
| 165 | *Bacillus halmapalus* | 0 | 0.013316 | 0.011897 |
| 166 | *Bacillus* sp. OK048 | 0.008235 | 0 | 0.016655 |
| 167 | *Bacillus pseudalcaliphilus* | 0 | 0.019973 | 0.004759 |
| 168 | *Bacillus* sp. CBA7126 | 0.024705 | 0 | 0 |
| 169 | *Bacillus* sp. 5mfcol3.1 | 0 | 0 | 0.023793 |
| 170 | *Bacillus* sp. TD41 | 0 | 0 | 0.023793 |
| 171 | *Bacillus aryabhattai* | 0.004117 | 0.016644 | 0.002379 |
| 172 | *Bacillus* sp. UNC438CL73TsuS30 | 0.004117 | 0.016644 | 0.002379 |
| 173 | *Bacillus* sp. FJAT-25496 | 0.012352 | 0.003329 | 0.007138 |
| 174 | *Bacillus manliponensis* | 0.004117 | 0.006658 | 0.011897 |
| 175 | *Bacillus* sp. 522_BSPC | 0 | 0.003329 | 0.019034 |
| 176 | *Bacillus ndiopicus* | 0.012352 | 0 | 0.009517 |
| 177 | *Bacillus australimaris* | 0 | 0 | 0.021414 |
| 178 | *Bacillus sinesaloumensis* | 0.012352 | 0.006658 | 0.002379 |
| 179 | *Bacillus weihaiensis* | 0.012352 | 0.006658 | 0.002379 |
| 180 | *Bacillus vireti* | 0.008235 | 0.003329 | 0.009517 |
| 181 | *Bacillus* sp. FJAT-25547 | 0 | 0.006658 | 0.014276 |
| 182 | *Bacillus* sp. RRD69 | 0.004117 | 0 | 0.016655 |
| 183 | *Bacillus* sp. TS-2 | 0.012352 | 0.003329 | 0.004759 |
| 184 | *Bacillus* sp. FJAT-27986 | 0.004117 | 0.006658 | 0.009517 |
| 185 | *Bacillus alcalophilus* | 0 | 0.003329 | 0.016655 |
| 186 | *Bacillus axarquiensis* | 0.008235 | 0.006658 | 0.004759 |
| 187 | *Bacillus endophyticus* | 0.004117 | 0.003329 | 0.011897 |
| 188 | *Bacillus* sp. 0711P9-1 | 0 | 0 | 0.019034 |
| 189 | *Bacillus* sp. FJAT-27245 | 0 | 0.016644 | 0.002379 |
| 190 | *Bacillus* sp. NC2-31 | 0.004117 | 0.009987 | 0.004759 |
| 191 | *Bacillus* sp. FJAT-26652 | 0.01647 | 0 | 0.002379 |
| 192 | *Bacillus shacheensis* | 0 | 0.006658 | 0.011897 |
| 193 | *Bacillus* sp. HMSC76G11 | 0 | 0.006658 | 0.011897 |
| 194 | *Bacillus* sp. BSC154 | 0.012352 | 0.003329 | 0.002379 |
| 195 | *Bacillus aurantiacus* | 0.004117 | 0.006658 | 0.007138 |
| 196 | *Bacillus drentensis* | 0.004117 | 0.006658 | 0.007138 |
| 197 | *Bacillus* sp. ES3 | 0.004117 | 0.006658 | 0.007138 |
| 198 | *Bacillus malacitensis* | 0 | 0.003329 | 0.014276 |
| 199 | *Bacillus wakoensis* | 0 | 0.003329 | 0.014276 |
| 200 | *Bacillus* sp. BS-02 | 0.004117 | 0.013316 | 0 |
| 201 | *Bacillus* sp. 105MF | 0 | 0.009987 | 0.007138 |
| 202 | *Bacillus* sp. H1a | 0 | 0 | 0.016655 |
| 203 | *Bacillus* sp. N35-10-4 | 0 | 0 | 0.016655 |
| 204 | *Bacillus* sp. ECU0013 | 0 | 0.016644 | 0 |
| 205 | *Bacillus okhensis* | 0.004117 | 0.009987 | 0.002379 |
| 206 | *Bacillus* sp. FJAT-29937 | 0.008235 | 0.003329 | 0.004759 |
| 207 | *Bacillus luciferensis* | 0 | 0.006658 | 0.009517 |
| 208 | *Bacillus* sp. LL01 | 0 | 0.006658 | 0.009517 |
| 209 | *Bacillus siamensis* | 0 | 0.009987 | 0.004759 |
| 210 | *Bacillus* sp. 123MFChir2 | 0 | 0 | 0.014276 |
| 211 | *Bacillus* sp. ABP14 | 0 | 0 | 0.014276 |
| 212 | *Bacillus* sp. M 2-6 | 0 | 0 | 0.014276 |
| 213 | *Bacillus akibai* | 0.008235 | 0.003329 | 0.002379 |
| 214 | *Bacillus jeotgali* | 0.008235 | 0.003329 | 0.002379 |
| 215 | *Bacillus muralis* | 0.008235 | 0.003329 | 0.002379 |
| 216 | *Bacillus* sp. JKS001846 | 0.004117 | 0 | 0.009517 |
| 217 | *Bacillus* sp. NRRL B-14911 | 0.004117 | 0 | 0.009517 |
| 218 | *Bacillus indicus* | 0.004117 | 0.006658 | 0.002379 |
| 219 | *Bacillus chagannorensis* | 0.008235 | 0 | 0.004759 |
| 220 | *Bacillus* sp. FJAT-27231 | 0.008235 | 0 | 0.004759 |
| 221 | *Bacillus* sp. FJAT-27251 | 0.008235 | 0 | 0.004759 |
| 222 | *Bacillus* sp. URHB0009 | 0 | 0.009987 | 0.002379 |
| 223 | *Bacillus daliensis* | 0.004117 | 0.003329 | 0.004759 |
| 224 | *Bacillus subterraneus* | 0.004117 | 0.003329 | 0.004759 |
| 225 | *Bacillus patagoniensis* | 0 | 0 | 0.011897 |
| 226 | *Bacillus* sp. bc15 | 0 | 0 | 0.011897 |
| 227 | *Bacillus abyssalis* | 0 | 0.006658 | 0.004759 |
| 228 | *Bacillus* sp. JCM 19034 | 0 | 0.006658 | 0.004759 |
| 229 | *Bacillus* sp. JCM 19041 | 0 | 0.006658 | 0.004759 |
| 230 | *Bacillus* sp. V-88 | 0 | 0.006658 | 0.004759 |
| 231 | *Bacillus* sp. FJAT-27916 | 0.004117 | 0 | 0.007138 |
| 232 | *Bacillus* sp. NH11B | 0.004117 | 0 | 0.007138 |
| 233 | *Bacillus hemicellulosilyticus* | 0.008235 | 0 | 0.002379 |
| 234 | *Bacillus* sp. L_1B0_12 | 0.008235 | 0 | 0.002379 |
| 235 | *Bacillus* sp. FJAT-27215 | 0 | 0.003329 | 0.007138 |
| 236 | *Bacillus halodurans* | 0 | 0.009987 | 0 |
| 237 | *Bacillus* sp. Pc3 | 0 | 0.009987 | 0 |
| 238 | *Bacillus* sp. UNCCL81 | 0 | 0.009987 | 0 |
| 239 | *Bacillus* sp. FJAT-20673 | 0.004117 | 0.003329 | 0.002379 |
| 240 | *Bacillus* sp. 166amftsu | 0 | 0 | 0.009517 |
| 241 | *Bacillus* sp. ru9509.4 | 0 | 0 | 0.009517 |
| 242 | *Bacillus* sp. TD42 | 0 | 0 | 0.009517 |
| 243 | *Bacillus* sp. TH007 | 0 | 0 | 0.009517 |
| 244 | *Bacillus boroniphilus* | 0 | 0.006658 | 0.002379 |
| 245 | *Bacillus* sp. 72 | 0 | 0.006658 | 0.002379 |
| 246 | *Bacillus* sp. YP1 | 0 | 0.006658 | 0.002379 |
| 247 | *Bacillus* sp. JCM 19047 | 0.004117 | 0 | 0.004759 |
| 248 | *Bacillus* sp. NRRL B-41282 | 0.004117 | 0 | 0.004759 |
| 249 | *Bacillus testis* | 0.004117 | 0 | 0.004759 |
| 250 | *Bacillus infantis* | 0.008235 | 0 | 0 |
| 251 | *Bacillus* sp. H15-1 | 0.008235 | 0 | 0 |
| 252 | *Bacillus novalis* | 0 | 0.003329 | 0.004759 |
| 253 | *Bacillus oleronius* | 0 | 0.003329 | 0.004759 |
| 254 | *Bacillus solimangrovi* | 0 | 0.003329 | 0.004759 |
| 255 | *Bacillus* sp. B25(2016b) | 0 | 0.003329 | 0.004759 |
| 256 | *Bacillus* sp. Soil745 | 0 | 0.003329 | 0.004759 |
| 257 | *Bacillus* sp. 17376 | 0.004117 | 0.003329 | 0 |
| 258 | *Bacillus* sp. MSP5.4 | 0.004117 | 0.003329 | 0 |
| 259 | *Bacillus* sp. NRRL B-41294 | 0.004117 | 0.003329 | 0 |
| 260 | *Bacillus selenatarsenatis* | 0 | 0 | 0.007138 |
| 261 | *Bacillus* sp. FJAT-21955 | 0 | 0 | 0.007138 |
| 262 | *Bacillus* sp. FJAT-27238 | 0 | 0 | 0.007138 |
| 263 | *Bacillus* sp. G3(2015) | 0 | 0 | 0.007138 |
| 264 | *Bacillus* sp. LK2 | 0 | 0 | 0.007138 |
| 265 | *Bacillus* sp. N35-10-2 | 0 | 0 | 0.007138 |
| 266 | *Bacillus* sp. UMTAT18 | 0 | 0 | 0.007138 |
| 267 | *Bacillus* sp. FJAT-26390 | 0 | 0.006658 | 0 |
| 268 | *Bacillus* sp. FJAT-22090 | 0.004117 | 0 | 0.002379 |
| 269 | *Bacillus aerophilus* | 0 | 0.003329 | 0.002379 |
| 270 | *Bacillus mannanilyticus* | 0 | 0.003329 | 0.002379 |
| 271 | *Bacillus* sp. 491mf | 0 | 0.003329 | 0.002379 |
| 272 | *Bacillus* sp. FJAT-25509 | 0 | 0.003329 | 0.002379 |
| 273 | *Bacillus* sp. FJAT-27264 | 0 | 0.003329 | 0.002379 |
| 274 | *Bacillus rhizosphaerae* | 0 | 0 | 0.004759 |
| 275 | *Bacillus* sp. 7_6_55CFAA_CT2 | 0 | 0 | 0.004759 |
| 276 | *Bacillus* sp. G1(2015b) | 0 | 0 | 0.004759 |
| 277 | *Bacillus* sp. Leaf75 | 0 | 0 | 0.004759 |
| 278 | *Bacillus* sp. NH24A2 | 0 | 0 | 0.004759 |
| 279 | *Bacillus* sp. RUTrin4 | 0 | 0 | 0.004759 |
| 280 | *Bacillus* sp. WP8 | 0 | 0 | 0.004759 |
