# Supplementary material for: Metagenomic-Metabolomic Mining of Kinema, a Naturally Fermented Soybean Food of the Eastern Himalayas
Source: Front Microbiol. 2022 Apr 29;13:868383. doi: 10.3389/fmicb.2022.868383 (PMC9106393; doi:10.3389/fmicb.2022.868383)
Supplement: Supplementary file 4 [file Table_4.DOCX]

| **Supplementary Table 7\| Overall lactic acid bacteria detected in *kinema* metagenome.** | | | | |
| --- | --- | --- | --- | --- |
| Sl. No. | Species | Relative Abundance (%) | | |
|  |  | *Kinema*  (India) | *Kinema*  (Nepal) | *Kinema*  (Bhutan) |
| 1 | *Streptococcus pneumoniae* | 0.078231 | 0.109854 | 0.763758 |
| 2 | *Enterococcus faecalis* | 0.247046 | 0.36285 | 0.306931 |
| 3 | *Enterococcus faecium* | 0.317042 | 0.066578 | 0.349759 |
| 4 | *Pediococcus acidilactici* | 0.275868 | 0.312916 | 0.140379 |
| 5 | *Vagococcus fluvialis* | 0.275868 | 0.003329 | 0.397345 |
| 6 | *Vagococcus lutrae* | 0.123523 | 0.006658 | 0.523448 |
| 7 | *Enterococcus cecorum* | 0.053527 | 0.079893 | 0.202241 |
| 8 | *Carnobacterium maltaromaticum* | 0.069996 | 0.139814 | 0.121345 |
| 9 | *Lactobacillus fermentum* | 0.028822 | 0.006658 | 0.254586 |
| 10 | *Lactobacillus salivarius* | 0.247046 | 0.016644 | 0.009517 |
| 11 | *Enterococcus asini* | 0.069996 | 0.006658 | 0.168931 |
| 12 | *Weissella paramesenteroides* | 0.148227 | 0.056591 | 0.007138 |
| 13 | *Streptococcus anginosus* | 0.020587 | 0.146471 | 0.014276 |
| 14 | *Enterococcus casseliflavus* | 0.045292 | 0.013316 | 0.10469 |
| 15 | *Vagococcus penaei* | 0.045292 | 0 | 0.071379 |
| 16 | *Lactobacillus agilis* | 0.107053 | 0 | 0.004759 |
| 17 | *Enterococcus saccharolyticus* | 0.094701 | 0.003329 | 0.011897 |
| 18 | *Streptococcus agalactiae* | 0.037057 | 0.046605 | 0.016655 |
| 19 | *Lactobacillus crispatus* | 0 | 0 | 0.073759 |
| 20 | *Streptococcus salivarius* | 0.020587 | 0.026631 | 0.016655 |
| 21 | *Enterococcus gallinarum* | 0.024705 | 0.006658 | 0.028552 |
| 22 | *Lactobacillus rhamnosus* | 0.01647 | 0.033289 | 0.009517 |
| 23 | *Lactobacillus delbrueckii* | 0.004117 | 0.003329 | 0.049966 |
| 24 | *Lactobacillus reuteri* | 0.008235 | 0.006658 | 0.03331 |
| 25 | *Enterococcus mundtii* | 0.020587 | 0.013316 | 0.014276 |
| 26 | *Streptococcus equinus* | 0 | 0.013316 | 0.03331 |
| 27 | *Enterococcus massiliensis* | 0.01647 | 0.019973 | 0.009517 |
| 28 | *Enterococcus durans* | 0.01647 | 0.009987 | 0.016655 |
| 29 | *Enterococcus aquimarinus* | 0.020587 | 0 | 0.019034 |
| 30 | *Leuconostoc citreum* | 0.012352 | 0.019973 | 0.004759 |
| 31 | *Sporolactobacillus terrae* | 0.020587 | 0.006658 | 0.009517 |
| 32 | *Lactobacillus casei* | 0.012352 | 0 | 0.023793 |
| 33 | *Lactococcus lactis* | 0.012352 | 0.006658 | 0.016655 |
| 34 | *Streptococcus suis* | 0.008235 | 0.009987 | 0.014276 |
| 35 | *Enterococcus italicus* | 0.020587 | 0 | 0.009517 |
| 36 | *Enterococcus* sp. RIT-PI-f | 0.01647 | 0 | 0.011897 |
| 37 | *Vagococcus* sp. D7T301 | 0.004117 | 0 | 0.023793 |
| 38 | *Carnobacterium gallinarum* | 0.008235 | 0 | 0.019034 |
| 39 | *Enterococcus phoeniculicola* | 0.008235 | 0 | 0.019034 |
| 40 | *Lactobacillus coleohominis* | 0 | 0.003329 | 0.023793 |
| 41 | *Carnobacterium viridans* | 0.020587 | 0.003329 | 0.002379 |
| 42 | *Lactobacillus dextrinicus* | 0 | 0 | 0.026172 |
| 43 | *Weissella hellenica* | 0.012352 | 0.013316 | 0 |
| 44 | *Enterococcus canis* | 0.012352 | 0.003329 | 0.009517 |
| 45 | *Lactobacillus jensenii* | 0.012352 | 0.003329 | 0.009517 |
| 46 | *Vagococcus teuberi* | 0.012352 | 0.003329 | 0.009517 |
| 47 | *Enterococcus pallens* | 0.008235 | 0 | 0.016655 |
| 48 | *Lactobacillus plantarum* | 0.008235 | 0 | 0.016655 |
| 49 | *Weissella jogaejeotgali* | 0.008235 | 0.016644 | 0 |
| 50 | *Lactobacillus mucosae* | 0.004117 | 0.003329 | 0.016655 |
| 51 | *Lactobacillus suebicus* | 0.004117 | 0.019973 | 0 |
| 52 | *Pediococcus pentosaceus* | 0.012352 | 0.006658 | 0.002379 |
| 53 | *Lactobacillus ruminis* | 0.004117 | 0.009987 | 0.007138 |
| 54 | *Enterococcus avium* | 0 | 0.013316 | 0.007138 |
| 55 | *Sporolactobacillus laevolacticus* | 0.012352 | 0 | 0.007138 |
| 56 | *Streptococcus parauberis* | 0 | 0.013316 | 0.004759 |
| 57 | *Enterococcus sulfureus* | 0.008235 | 0 | 0.009517 |
| 58 | *Weissella oryzae* | 0 | 0.013316 | 0.002379 |
| 59 | *Streptococcus equi* | 0.004117 | 0.006658 | 0.004759 |
| 60 | *Tetragenococcus muriaticus* | 0.008235 | 0 | 0.007138 |
| 61 | *Enterococcus hirae* | 0.012352 | 0 | 0.002379 |
| 62 | *Tetragenococcus halophilus* | 0.008235 | 0.003329 | 0.002379 |
| 63 | *Carnobacterium* sp. CP1 | 0 | 0.006658 | 0.007138 |
| 64 | *Enterococcus dispar* | 0.004117 | 0 | 0.009517 |
| 65 | *Enterococcus* sp. kppr-6 | 0.004117 | 0.006658 | 0.002379 |
| 66 | *Enterococcus canintestini* | 0.008235 | 0 | 0.004759 |
| 67 | *Enterococcus termitis* | 0.008235 | 0 | 0.004759 |
| 68 | *Lactobacillus oris* | 0.008235 | 0 | 0.004759 |
| 69 | *Lactobacillus paralimentarius* | 0.012352 | 0 | 0 |
| 70 | *Carnobacterium inhibens* | 0.004117 | 0.003329 | 0.004759 |
| 71 | *Tetragenococcus solitarius* | 0 | 0 | 0.011897 |
| 72 | *Carnobacterium mobile* | 0.008235 | 0.003329 | 0 |
| 73 | *Lactobacillus sunkii* | 0.008235 | 0.003329 | 0 |
| 74 | *Streptococcus infantarius* | 0.008235 | 0.003329 | 0 |
| 75 | *Carnobacterium* sp. AT7 | 0.004117 | 0 | 0.007138 |
| 76 | *Enterococcus columbae* | 0.004117 | 0 | 0.007138 |
| 77 | *Lactococcus garvieae* | 0.004117 | 0 | 0.007138 |
| 78 | *Sporolactobacillus vineae* | 0.004117 | 0 | 0.007138 |
| 79 | *Lactococcus piscium* | 0.004117 | 0.006658 | 0 |
| 80 | *Lactobacillus farciminis* | 0.008235 | 0 | 0.002379 |
| 81 | *Lactobacillus ghanensis* | 0.008235 | 0 | 0.002379 |
| 82 | *Weissella thailandensis* | 0 | 0.009987 | 0 |
| 83 | *Lactococcus raffinolactis* | 0.004117 | 0.003329 | 0.002379 |
| 84 | *Enterococcus rivorum* | 0 | 0 | 0.009517 |
| 85 | *Lactobacillus gasseri* | 0 | 0 | 0.009517 |
| 86 | *Lactobacillus brevis* | 0 | 0.006658 | 0.002379 |
| 87 | *Enterococcus* sp. HSIEG1 | 0.004117 | 0 | 0.004759 |
| 88 | *Enterococcus ureasiticus* | 0.008235 | 0 | 0 |
| 89 | *Lactobacillus helveticus* | 0.008235 | 0 | 0 |
| 90 | *Lactococcus chungangensis* | 0.008235 | 0 | 0 |
| 91 | *Leuconostoc mesenteroides* | 0.008235 | 0 | 0 |
| 92 | *Carnobacterium iners* | 0.004117 | 0.003329 | 0 |
| 93 | *Lactobacillus nodensis* | 0.004117 | 0.003329 | 0 |
| 94 | *Streptococcus marmotae* | 0.004117 | 0.003329 | 0 |
| 95 | *Carnobacterium divergens* | 0 | 0 | 0.007138 |
| 96 | *Enterococcus malodoratus* | 0 | 0 | 0.007138 |
| 97 | *Enterococcus silesiacus* | 0 | 0 | 0.007138 |
| 98 | *Enterococcus villorum* | 0 | 0 | 0.007138 |
| 99 | *Lactobacillus hokkaidonensis* | 0 | 0 | 0.007138 |
| 100 | *Lactobacillus paracasei* | 0 | 0 | 0.007138 |
| 101 | *Streptococcus pyogenes* | 0 | 0 | 0.007138 |
| 102 | *Lactobacillus curvatus* | 0 | 0.006658 | 0 |
| 103 | *Enterococcus moraviensis* | 0.004117 | 0 | 0.002379 |
| 104 | *Lactobacillus murinus* | 0.004117 | 0 | 0.002379 |
| 105 | *Lactobacillus vaccinostercus* | 0.004117 | 0 | 0.002379 |
| 106 | *Lactobacillus vaginalis* | 0.004117 | 0 | 0.002379 |
| 107 | *Lactobacillus wasatchensis* | 0.004117 | 0 | 0.002379 |
| 108 | *Streptococcus sobrinus* | 0.004117 | 0 | 0.002379 |
| 109 | *Streptococcus uberis* | 0.004117 | 0 | 0.002379 |
| 110 | *Carnobacterium alterfunditum* | 0 | 0.003329 | 0.002379 |
| 111 | *Enterococcus pseudoavium* | 0 | 0.003329 | 0.002379 |
| 112 | *Enterococcus haemoperoxidus* | 0 | 0 | 0.004759 |
| 113 | *Enterococcus* sp. HMSC076E04 | 0 | 0 | 0.004759 |
| 114 | *Lactobacillus acetotolerans* | 0 | 0 | 0.004759 |
| 115 | *Lactobacillus fuchuensis* | 0 | 0 | 0.004759 |
| 116 | *Lactobacillus harbinensis* | 0 | 0 | 0.004759 |
| 117 | *Lactobacillus ingluviei* | 0 | 0 | 0.004759 |
| 118 | *Lactobacillus johnsonii* | 0 | 0 | 0.004759 |
| 119 | *Lactobacillus* sp. HMSC08B12 | 0 | 0 | 0.004759 |
| 120 | *Lactobacillus* sp. HMSC24D01 | 0 | 0 | 0.004759 |
| 121 | *Streptococcus mitis* | 0 | 0 | 0.004759 |

| **Supplementary Table 8: Overall viral species detected in *kinema* metagenome.** | | | | |
| --- | --- | --- | --- | --- |
| Sl. No. | Species | Relative Abundance (%) | | |
|  |  | *Kinema*  (India) | *Kinema*  (Nepal) | *Kinema*  (Bhutan) |
| 1 | *Aeribacillus* phage AP45 | 0 | 0.023302 | 0 |
| 2 | *Bacillus* phage 0305phi8-36 | 0 | 0 | 0.002379 |
| 3 | *Bacillus* phage 1102phi1-3 | 0.008235 | 0 | 0 |
| 4 | *Bacillus* phage Basilisk | 0.004117 | 0.016644 | 0 |
| 5 | *Bacillus* phage BCD7 | 0 | 0 | 0.002379 |
| 6 | *Bacillus* phage BCJA1c | 0.004117 | 0 | 0 |
| 7 | *Bacillus* phage BCP8-2 | 0.004117 | 0 | 0 |
| 8 | *Bacillus* phage Bobb | 0.004117 | 0.016644 | 0 |
| 9 | *Bacillus* phage BSNPO1 | 0.008235 | 0.046605 | 0 |
| 10 | *Bacillus* phage Eldridge | 0.004117 | 0 | 0 |
| 11 | *Bacillus* phage Grass | 0.156462 | 2.045939 | 0 |
| 12 | *Bacillus* phage Mgbh1 | 0.01647 | 0.036618 | 0 |
| 13 | *Bacillus* phage PBC1 | 0 | 0 | 0.002379 |
| 14 | *Bacillus* phage PBC4 | 0.024705 | 0.019973 | 0 |
| 15 | *Bacillus* phage phiNIT1 | 0.172932 | 4.327563 | 0 |
| 16 | *Bacillus* phage PM1 | 1.021122 | 0.199734 | 0 |
| 17 | *Bacillus* phage QCM8 | 0 | 0.003329 | 0 |
| 18 | *Bacillus* phage Shbh1 | 0.004117 | 0.003329 | 0.002379 |
| 19 | *Bacillus* phage SIOphi | 0.782312 | 0.009987 | 0 |
| 20 | *Bacillus* phage SP-10 | 0.004117 | 0.056591 | 0.002379 |
| 21 | *Bacillus* phage SPG24 | 0.008235 | 0.126498 | 0.002379 |
| 22 | *Bacillus* phage SPP1 | 0.008235 | 0.009987 | 0.002379 |
| 23 | *Bacillus* phage Stahl | 0 | 0 | 0.002379 |
| 24 | *Bacillus* phage vB_BanS-Tsamsa | 0.024705 | 0.003329 | 0 |
| 25 | *Bacillus* virus 1 | 0 | 0 | 0.002379 |
| 26 | *Bacillus* virus Andromeda | 0 | 0 | 0.002379 |
| 27 | *Bacillus* virus G | 0 | 0 | 0.002379 |
| 28 | *Cellulophaga* phage phi18:1 | 0.004117 | 0 | 0 |
| 29 | *Enterococcus* phage EFDG1 | 0 | 0.003329 | 0 |
| 30 | *Enterococcus* phage IME-EFm5 | 0.004117 | 0 | 0 |
| 31 | *Geobacillus* phage GBSV1 | 0 | 0.006658 | 0 |
| 32 | *Geobacillus* virus E2 | 0.004117 | 0 | 0 |
| 33 | *Geobacillus* virus E3 | 0 | 0.076565 | 0 |
| 34 | *Lactobacillus* phage phiPYB5 | 0 | 0 | 0.002379 |
| 35 | *Mycobacterium* phage Idleandcovert | 0.004117 | 0 | 0 |
| 36 | *Pseudoalteromonas* phage H105/1 | 0.004117 | 0 | 0 |
| 37 | *Pseudoalteromonas* phage vB_PspS-H40/1 | 0 | 0 | 0.002379 |
| 38 | *Staphylococcus* phage Andhra | 0.008235 | 0 | 0 |
| 39 | *Staphylococcus* phage BP39 | 0.008235 | 0 | 0 |
| 40 | *Staphylococcus* phage Stau2 | 0.004117 | 0 | 0 |
| 41 | *Streptococcus* phage C1 | 0.004117 | 0 | 0 |
| 42 | *Vibrio* phage ICP1 | 0 | 0 | 0.002379 |
| 43 | unclassified viral species | 0.004117 | 0.009987 | 0 |
