# Supplementary material for: Metagenomic-Metabolomic Mining of Kinema, a Naturally Fermented Soybean Food of the Eastern Himalayas
Source: Front Microbiol. 2022 Apr 29;13:868383. doi: 10.3389/fmicb.2022.868383 (PMC9106393; doi:10.3389/fmicb.2022.868383)
Supplement: Supplementary file 5 [file Table_5.DOCX]

| **Supplementary Table 9: Overall eukaryotic species (molds, yeasts and other microbial eukaryotes) detected in *kinema* metagenome.** | | | | |
| --- | --- | --- | --- | --- |
| Sl. No. | Species | Relative Abundance (%) | | |
|  |  | *Kinema*  (India) | *Kinema*  (Nepal) | *Kinema*  (Bhutan) |
| Molds | |  |  |  |
| 1 | *Acidomyces richmondensis* | 0.004117 | 0 | 0 |
| 2 | *Aspergillus carbonarius* | 0 | 0 | 0.002379 |
| 3 | *Batrachochytrium dendrobatidis* | 0.004117 | 0.006658 | 0 |
| 4 | *Conidiobolus coronatus* | 0 | 0.003329 | 0 |
| 5 | *Exophiala aquamarina* | 0.004117 | 0 | 0 |
| 6 | *Geotrichum candidum* | 0.012352 | 0 | 0 |
| 7 | *Hirsutella minnesotensis* | 0 | 0.003329 | 0 |
| 8 | *Hypsizygus marmoreus* | 0.004117 | 0 | 0 |
| 9 | *Lichtheimia corymbifera* | 0 | 0.003329 | 0 |
| 10 | *Lichtheimia ramosa* | 0.004117 | 0 | 0 |
| 11 | *Mucor ambiguus* | 0.01647 | 0.02996 | 0.026172 |
| 12 | *Mucor circinelloides* | 0.004117 | 0 | 0 |
| 13 | *Penicillium steckii* | 0 | 0.006658 | 0 |
| 14 | *Puccinia striiformis* | 0 | 0.003329 | 0 |
| 15 | *Pyronema omphalodes* | 0.004117 | 0 | 0 |
| 16 | *Sclerotinia sclerotiorum* | 0 | 0.003329 | 0 |
| 17 | *Yarrowia lipolytica* | 0 | 0 | 0.002379 |
| Yeasts | | | | |
| 1 | *Candida albicans* | 0.004117 | 0 | 0 |
| 2 | *Candida maltosa* | 0.004117 | 0 | 0 |
| 3 | *Candida orthopsilosis* | 0.004117 | 0 | 0 |
| 4 | *Candida sake* | 0.004117 | 0 | 0 |
| 5 | *Candida subhashii* | 0.004117 | 0 | 0 |
| 6 | *Candida tanzawaensis* | 0.008235 | 0 | 0 |
| 7 | *Cyberlindnera jadinii* | 0.004117 | 0 | 0 |
| 8 | *Debaryomyces fabryi* | 0.004117 | 0 | 0 |
| 9 | *Kuraishia capsulata* | 0.004117 | 0 | 0.002379 |
| 10 | *Leucosporidium creatinivorum* | 0.004117 | 0 | 0 |
| 11 | *Metschnikowia bicuspidata* | 0.004117 | 0 | 0 |
| 12 | *Meyerozyma guilliermondii* | 0.008235 | 0 | 0 |
| 13 | *Pichia kudriavzevii* | 0.020587 | 0.009987 | 0.095172 |
| 14 | *Pneumocystis jirovecii* | 0.201754 | 0.103196 | 0 |
| 15 | *Rhodotorula graminis* | 0 | 0.003329 | 0 |
| 16 | *Spathaspora passalidarum* | 0.004117 | 0 | 0 |
| 17 | *Trichosporon asahii* | 0.008235 | 0.009987 | 0 |
| Other microbial eukaryotes | | | | |
| 1 | *Acetabularia acetabulum* | 0.004117 | 0 | 0 |
| 2 | *Asterionella formosa* | 0 | 0 | 0.002379 |
| 3 | *Chlorella variabilis* | 0.004117 | 0 | 0 |
| 4 | *Ectocarpus siliculosus* | 0 | 0 | 0.002379 |
| 5 | *Emiliania huxleyi* | 0 | 0 | 0.002379 |
| 6 | *Fragilariopsis cylindrus* | 0.004117 | 0 | 0 |
| 7 | *Galdieria sulphuraria* | 0.004117 | 0 | 0 |
| 8 | *Ichthyophthirius multifiliis* | 0.004117 | 0.003329 | 0 |
| 9 | *Nannochloropsis gaditana* | 0.004117 | 0 | 0 |
| 10 | *Neospora caninum* | 0 | 0 | 0.004759 |
| 11 | *Oxytricha trifallax* | 0.004117 | 0 | 0.002379 |
| 12 | *Stentor coeruleus* | 0.004117 | 0 | 0 |
| 13 | *Stylonychia lemnae* | 0.008235 | 0 | 0.002379 |
| 14 | *Tetrahymena thermophila* | 0.004117 | 0 | 0 |
| 15 | *Thalassiosira oceanica* | 0 | 0.003329 | 0.002379 |
| 16 | *Thalassiosira pseudonana* | 0 | 0 | 0.002379 |
| 17 | *Parasitella parasitica* | 0 | 0 | 0.002379 |
| 18 | *Plasmodiophora brassicae* | 0.004117 | 0 | 0 |
| 19 | *Acanthamoeba castellanii* | 0.004117 | 0 | 0.002379 |
| 20 | *Pseudocohnilembus persalinus* | 0 | 0 | 0.002379 |
| 21 | *Trypanosoma theileri* | 0 | 0 | 0.002379 |
| 22 | *Trypanosoma brucei* | 0 | 0.003329 | 0 |
| 23 | unclassified eukaryotic species | 0.032939 | 0.026631 | 0.03569 |

| **Supplementary Tables 10: Overall archaeal species detected in *kinema* metagenome.** | | | | |
| --- | --- | --- | --- | --- |
| Sl. No. | Species | Relative Abundance (%) | | |
|  |  | Kinema  (India) | Kinema  (Nepal) | Kinema  (Bhutan) |
| 1 | *Haloprofundus marisrubri* | 0 | 0 | 0.002379 |
| 2 | *Haloterrigena thermotolerans* | 0 | 0.003329 | 0 |
| 3 | *Methanocaldococcus jannaschii* | 0 | 0.009987 | 0 |
| 4 | *Methanocella arvoryzae* | 0 | 0.003329 | 0 |
| 5 | *Methanocella paludicola* | 0.004117 | 0 | 0 |
| 6 | *Methanolinea tarda* | 0.004117 | 0 | 0 |
| 7 | *Methanoregula formicica* | 0 | 0 | 0.002379 |
| 8 | *Methanosaeta harundinacea* | 0 | 0.003329 | 0 |
| 9 | *Methanosalsum zhilinae* | 0 | 0.003329 | 0 |
| 10 | *Pyrococcus furiosus* | 0 | 0.003329 | 0 |
| 11 | *Thermoproteus* sp. AZ2 | 0 | 0 | 0.002379 |
| 12 | unclassified archaeal species | 0.004117 | 0.003329 | 0 |
