# Supplementary material for: Metagenomic-Metabolomic Mining of Kinema, a Naturally Fermented Soybean Food of the Eastern Himalayas
Source: Front Microbiol. 2022 Apr 29;13:868383. doi: 10.3389/fmicb.2022.868383 (PMC9106393; doi:10.3389/fmicb.2022.868383)
Supplement: Supplementary file 6 [file Table_6.DOCX]

| **Supplementary Table 11: Unique and shared bacterial species in *kinema*** | | |
| --- | --- | --- |
| Samples site | No. of species | Bacterial species |
| Bhutan, India, Nepal | 479 | *Paludibacter propionicigenes* |
|  |  | *Acinetobacter* sp. WCHA30 |
|  |  | *Bacillus clausii* |
|  |  | *Parageobacillus thermoglucosidans* |
|  |  | *Bacillus subterraneus* |
|  |  | *Geobacillus* sp. LEMMY01 |
|  |  | *Domibacillus indicus* |
|  |  | *Salibacterium halotolerans* |
|  |  | *Enterococcus faecium* |
|  |  | *Chlamydia trachomatis* |
|  |  | *Seinonella peptonophila* |
|  |  | *Paenibacillus* sp. oral taxon 786 |
|  |  | *Paenalcaligenes hominis* |
|  |  | *Bacillus bogoriensis* |
|  |  | *Selenomonas ruminantium* |
|  |  | *Clostridium beijerinckii* |
|  |  | *Lutispora thermophila* |
|  |  | *Bacillus safensis* |
|  |  | *Paenibacillus larvae* |
|  |  | *Paenisporosarcina* sp. TG20 |
|  |  | *Carnobacterium viridans* |
|  |  | *Bacillus gottheilii* |
|  |  | *Brevibacillus parabrevis* |
|  |  | *Bacillus korlensis* |
|  |  | *Bordetella trematum* |
|  |  | *Paenibacillus selenitireducens* |
|  |  | *Geobacillus* sp. BCO2 |
|  |  | *Anaerosalibacter* sp. Marseille-P3206 |
|  |  | *Virgibacillus pantothenticus* |
|  |  | *Bacillus caseinilyticus* |
|  |  | *Bacillus psychrosaccharolyticus* |
|  |  | *Bacillus* sp. SB47 |
|  |  | *Beggiatoa* sp. 4572_84 |
|  |  | *Bacillus ligniniphilus* |
|  |  | *Bacillus atrophaeus* |
|  |  | *Lysinibacillus varians* |
|  |  | *Jeotgalibaca* sp. PTS2502 |
|  |  | *Lysinibacillus massiliensis* |
|  |  | *Dechloromonas aromatica* |
|  |  | *Bacillus glycinifermentans* |
|  |  | *Pontibacillus litoralis* |
|  |  | *Rubrivivax benzoatilyticus* |
|  |  | *Bacillus salsus* |
|  |  | *Bacillus gaemokensis* |
|  |  | *Vagococcus fluvialis* |
|  |  | *Bacillus krulwichiae* |
|  |  | *Sediminibacillus halophilus* |
|  |  | *Lentibacillus halodurans* |
|  |  | *Geobacillus vulcani* |
|  |  | *Gracilibacillus halophilus* |
|  |  | *Bacillus* sp. J37 |
|  |  | *Brevibacillus brevis* |
|  |  | *Peptostreptococcus russellii* |
|  |  | *Bacillus nakamurai* |
|  |  | *Streptococcus pneumoniae* |
|  |  | *Hydrogenophaga* sp. Root209 |
|  |  | *Bacillus* sp. JS |
|  |  | *Ignatzschineria larvae* |
|  |  | *Weissella paramesenteroides* |
|  |  | *Longilinea arvoryzae* |
|  |  | *Bacillus akibai* |
|  |  | *Amphibacillus jilinensis* |
|  |  | *Tetragenococcus halophilus* |
|  |  | *Bhargavaea ginsengi* |
|  |  | *Vagococcus lutrae* |
|  |  | *Bacillus muralis* |
|  |  | *Aeribacillus pallidus* |
|  |  | *Clostridium ihumii* |
|  |  | *Cyclobacterium qasimii* |
|  |  | *Bacillus* sp. UNC41MFS5 |
|  |  | *Bacillus paralicheniformis* |
|  |  | *Pontibacillus halophilus* |
|  |  | *Fictibacillus phosphorivorans* |
|  |  | *Varibaculum timonense* |
|  |  | *Jeotgalibacillus campisalis* |
|  |  | *Carnobacterium maltaromaticum* |
|  |  | *Bacillus* sp. NSP9.1 |
|  |  | *Geobacillus* sp. WCH70 |
|  |  | *Bacillus* sp. EGD-AK10 |
|  |  | *Alteribacillus bidgolensis* |
|  |  | *Azonexus hydrophilus* |
|  |  | *Bacillus acidiproducens* |
|  |  | *Fusobacterium nucleatum* |
|  |  | *Bacillus coahuilensis* |
|  |  | *Aneurinibacillus terranovensis* |
|  |  | *Psychrobacillus* sp. OK032 |
|  |  | *Oceanobacillus sojae* |
|  |  | *Anoxybacillus flavithermus* |
|  |  | *Chlamydia abortus* |
|  |  | *Bacillus niacini* |
|  |  | *Shimazuella kribbensis* |
|  |  | *Bacillus galactosidilyticus* |
|  |  | *Edaphobacillus lindanitolerans* |
|  |  | *Bacillus koreensis* |
|  |  | *Bacillus subtilis* |
|  |  | *Bacillus jeotgali* |
|  |  | *Alkalibacterium* sp. AK22 |
|  |  | *Bacillus badius* |
|  |  | *Paenibacillus polymyxa* |
|  |  | *Lonsdalea quercina* |
|  |  | *Lysinibacillus odysseyi* |
|  |  | *Bacillus shackletonii* |
|  |  | *Sporosarcina globispora* |
|  |  | *Bacillus andreraoultii* |
|  |  | *Bacillus pumilus* |
|  |  | *Ornithinibacillus contaminans* |
|  |  | *Enterococcus* sp. kppr-6 |
|  |  | *Paraliobacillus* sp. PM-2 |
|  |  | *Bacillus* sp. BSC154 |
|  |  | *Dechloromonas denitrificans* |
|  |  | *Bacillus* sp. SG-1 |
|  |  | *Jeotgalibacillus malaysiensis* |
|  |  | *Enterococcus cecorum* |
|  |  | *Bacillus licheniformis* |
|  |  | *Oceanobacillus timonensis* |
|  |  | *Oceanobacillus damuensis* |
|  |  | *Bacillus* sp. UNC437CL72CviS29 |
|  |  | *Gracilibacillus boraciitolerans* |
|  |  | *Anoxybacillus ayderensis* |
|  |  | *Bacillus massilioanorexius* |
|  |  | *Bacillus fumarioli* |
|  |  | *Beggiatoa leptomitiformis* |
|  |  | *Fictibacillus macauensis* |
|  |  | *Virgibacillus siamensis* |
|  |  | *Virgibacillus soli* |
|  |  | *Tuberibacillus* sp. Marseille-P3662 |
|  |  | *Paenibacillus naphthalenovorans* |
|  |  | *Parvimonas micra* |
|  |  | *Bacillus* sp. VT-16-64 |
|  |  | *Ruminococcus flavefaciens* |
|  |  | *Bacillus* sp. EB01 |
|  |  | *Anoxybacillus* sp. BCO1 |
|  |  | *Virgibacillus* sp. SK37 |
|  |  | *Enterococcus faecalis* |
|  |  | *Bacillus* sp. FJAT-27997 |
|  |  | *Caryophanon latum* |
|  |  | *Bacillus* sp. MSP13 |
|  |  | *Atopococcus tabaci* |
|  |  | *Acinetobacter* sp. YZS-X1-1 |
|  |  | *Shigella dysenteriae* |
|  |  | *Bacillus* sp. MB2021 |
|  |  | *Streptococcus anginosus* |
|  |  | *Enterococcus gallinarum* |
|  |  | *Halobacillus massiliensis* |
|  |  | *Kurthia* sp. 11kri321 |
|  |  | *Bacillus* sp. CC120222-01 |
|  |  | *Corynebacterium glutamicum* |
|  |  | *Bacillus amyloliquefaciens* |
|  |  | *Marinilactibacillus piezotolerans* |
|  |  | *Sporosarcina psychrophila* |
|  |  | *Paenibacillus macerans* |
|  |  | *Alicyclobacillus contaminans* |
|  |  | *Wohlfahrtiimonas larvae* |
|  |  | *Aliicoccus persicus* |
|  |  | *Parageobacillus thermantarcticus* |
|  |  | *Bacillus* sp. UNC125MFCrub1.1 |
|  |  | *Paucisalibacillus globulus* |
|  |  | *Bacillus okhensis* |
|  |  | *Bacillus weihenstephanensis* |
|  |  | *Bacillus marisflavi* |
|  |  | *Bacillus aidingensis* |
|  |  | *Bacillus agaradhaerens* |
|  |  | *Proteus mirabilis* |
|  |  | *Ureibacillus thermosphaericus* |
|  |  | *Lactobacillus salivarius* |
|  |  | *Bacillus* sp. Marseille-P2384 |
|  |  | *Oceanobacillus massiliensis* |
|  |  | *Clostridium sporogenes* |
|  |  | *Oceanospirillum multiglobuliferum* |
|  |  | *Staphylococcus aureus* |
|  |  | *Salinicoccus albus* |
|  |  | *Geobacillus* sp. 8 |
|  |  | *Bacillus smithii* |
|  |  | *Leuconostoc citreum* |
|  |  | *Paenibacillus wynnii* |
|  |  | *Bacillus mycoides* |
|  |  | *Bacillus* sp. B-jedd |
|  |  | *Enterococcus saccharolyticus* |
|  |  | *Natribacillus halophilus* |
|  |  | *Streptococcus suis* |
|  |  | *Listeria grayi* |
|  |  | *Geobacillus* sp. 12AMOR1 |
|  |  | *Lactobacillus fermentum* |
|  |  | *Bacillus* sp. FJAT-27445 |
|  |  | *Bacillus indicus* |
|  |  | *Bacillus aryabhattai* |
|  |  | *Bacillus cihuensis* |
|  |  | *Enterococcus massiliensis* |
|  |  | *Bacillus obstructivus* |
|  |  | *Amphibacillus xylanus* |
|  |  | *Staphylococcus saprophyticus* |
|  |  | *Bacillus drentensis* |
|  |  | *Aquibacillus* sp. Marseille-P3518 |
|  |  | *Bacillus* sp. FJAT-22058 |
|  |  | *Bacillus massiliosenegalensis* |
|  |  | *Bacillus fordii* |
|  |  | *Domibacillus* sp. SAB 38 |
|  |  | *Opitutus* sp. GAS368 |
|  |  | *Staphylococcus sciuri* |
|  |  | *Acinetobacter* sp. NIPH 899 |
|  |  | *Burkholderia pseudomallei* |
|  |  | *Kurthia massiliensis* |
|  |  | *Paenibacillus yonginensis* |
|  |  | *Enterococcus casseliflavus* |
|  |  | *Lactobacillus mucosae* |
|  |  | *Bacillus gobiensis* |
|  |  | *Halobacillus hunanensis* |
|  |  | *Bacillus* sp. FMQ74 |
|  |  | *Bacillus clarkii* |
|  |  | *Bacillus* sp. CMAA 1185 |
|  |  | *Bacillus tuaregi* |
|  |  | *Lysinibacillus fusiformis* |
|  |  | *Sporosarcina* sp. HYO08 |
|  |  | *Kurthia senegalensis* |
|  |  | *Bacillus* sp. J33 |
|  |  | *Tuberibacillus calidus* |
|  |  | *Bacillus ginsengihumi* |
|  |  | *Bacillus* sp. ES3 |
|  |  | *Lentibacillus jeotgali* |
|  |  | *Lactobacillus rhamnosus* |
|  |  | *Pontibacillus yanchengensis* |
|  |  | *Jeotgalibacillus alimentarius* |
|  |  | *Atopostipes suicloacalis* |
|  |  | *Bacillus coagulans* |
|  |  | *Bacillus circulans* |
|  |  | *Cronobacter sakazakii* |
|  |  | *Defluviitalea phaphyphila* |
|  |  | *Sporosarcina ureae* |
|  |  | *Bacillus* sp. BT1B_CT2 |
|  |  | *Bacillus sporothermodurans* |
|  |  | *Bacillus manliponensis* |
|  |  | *Sporosarcina* sp. EUR3 2.2.2 |
|  |  | *Bacillus anthracis* |
|  |  | *Bacillus cecembensis* |
|  |  | *Paenibacillus alvei* |
|  |  | *Trichococcus palustris* |
|  |  | *Pseudomonas* sp. EGD-AK9 |
|  |  | *Bacillus velezensis* |
|  |  | *Paenibacillus terrigena* |
|  |  | *Bhargavaea cecembensis* |
|  |  | *Geobacillus* sp. WSUCF1 |
|  |  | *Bacillus lentus* |
|  |  | *Parageobacillus caldoxylosilyticus* |
|  |  | *Bacillus daliensis* |
|  |  | *Acinetobacter* sp. NIPH 2171 |
|  |  | *Geobacillus* sp. PA-3 |
|  |  | *Bacillus* sp. FJAT-27986 |
|  |  | *Kurthia huakuii* |
|  |  | *Tissierella praeacuta* |
|  |  | *Lactobacillus delbrueckii* |
|  |  | *Azospirillum lipoferum* |
|  |  | *Bacillus alveayuensis* |
|  |  | *Paenibacillus* sp. P22 |
|  |  | *Lysinibacillus* sp. FJAT-14222 |
|  |  | *Ilumatobacter coccineus* |
|  |  | *Bacillus thermoamylovorans* |
|  |  | *Lysinibacillus saudimassiliensis* |
|  |  | *Wohlfahrtiimonas chitiniclastica* |
|  |  | *Bacillus bataviensis* |
|  |  | *Bacillus* sp. X1(2014) |
|  |  | *Lactococcus raffinolactis* |
|  |  | *Heliobacterium modesticaldum* |
|  |  | *Bacillus* sp. FJAT-29937 |
|  |  | *Gorillibacterium massiliense* |
|  |  | *Thermoactinomyces daqus* |
|  |  | *Alkalibacillus haloalkaliphilus* |
|  |  | *Aneurinibacillus aneurinilyticus* |
|  |  | *Paeniclostridium sordellii* |
|  |  | *Streptococcus agalactiae* |
|  |  | *Bacillus megaterium* |
|  |  | *Bacillus* sp. UNC438CL73TsuS30 |
|  |  | *Viridibacillus arenosi* |
|  |  | *Virgibacillus senegalensis* |
|  |  | *Bacillus thuringiensis* |
|  |  | *Enterococcus asini* |
|  |  | *Paenibacillus senegalensis* |
|  |  | *Lysinibacillus xylanilyticus* |
|  |  | *Bacillus aurantiacus* |
|  |  | *Bacillus* sp. FJAT-27225 |
|  |  | *Corynebacterium casei* |
|  |  | *Bacillus weihaiensis* |
|  |  | *Providencia stuartii* |
|  |  | *Bacillus alkalitelluris* |
|  |  | *Klebsiella pneumoniae* |
|  |  | *Lactococcus lactis* |
|  |  | *Anoxybacillus amylolyticus* |
|  |  | *Bacillus acidicola* |
|  |  | *Brevibacillus* sp. WF146 |
|  |  | *Bacillus* sp. NRRL B-41327 |
|  |  | *Bacillus persicus* |
|  |  | *Anaerobacillus* sp. NB2006 |
|  |  | *Bacillus* sp. LF1 |
|  |  | *Acinetobacter junii* |
|  |  | *Oceanobacillus* sp. Castelsardo |
|  |  | *Lysinibacillus* sp. ZYM-1 |
|  |  | *Bacillus niameyensis* |
|  |  | *Lysinibacillus* sp. FJAT-14745 |
|  |  | *Listeria monocytogenes* |
|  |  | *Bacillus vireti* |
|  |  | *Bacillus farraginis* |
|  |  | *Anaerobacillus alkalidiazotrophicus* |
|  |  | *Bacillus* sp. NC2-31 |
|  |  | *Clostridioides mangenotii* |
|  |  | *Virgibacillus alimentarius* |
|  |  | *Lysinibacillus macroides* |
|  |  | *Lysinibacillus sphaericus* |
|  |  | *Dendrosporobacter quercicolus* |
|  |  | *Acinetobacter bohemicus* |
|  |  | *Caenibacillus caldisaponilyticus* |
|  |  | *Ornithinibacillus scapharcae* |
|  |  | *Gracilibacillus ureilyticus* |
|  |  | *Bacillus oryziterrae* |
|  |  | *Oceanobacillus picturae* |
|  |  | *Bacillus timonensis* |
|  |  | *Paenibacillus* sp. P1XP2 |
|  |  | *Carboxydocella* sp. ULO1 |
|  |  | *Bacillus* sp. FJAT-20673 |
|  |  | *Virgibacillus* sp. 6R |
|  |  | *Bacillus* sp. F56 |
|  |  | *Carnobacterium inhibens* |
|  |  | *Fuerstia marisgermanicae* |
|  |  | *Staphylococcus hominis* |
|  |  | *Paenisporosarcina indica* |
|  |  | *Lysinibacillus* sp. BF-4 |
|  |  | *Clostridium colicanis* |
|  |  | *Bacillus cereus* |
|  |  | *Kerstersia gyiorum* |
|  |  | *Enterococcus durans* |
|  |  | *Streptococcus equi* |
|  |  | *Lysinibacillus manganicus* |
|  |  | *Lysinibacillus* sp. AC-3 |
|  |  | *Bacillus methanolicus* |
|  |  | *Sporosarcina koreensis* |
|  |  | *Lihuaxuella thermophila* |
|  |  | *Bacillus endophyticus* |
|  |  | *Paenibacillus popilliae* |
|  |  | *Bacillus dakarensis* |
|  |  | *Clostridium botulinum* |
|  |  | *Lysinibacillus sinduriensis* |
|  |  | *Rheinheimera* sp. SA_1 |
|  |  | *Massilia* sp. CF038 |
|  |  | *Paenibacillus massiliensis* |
|  |  | *Paucisalibacillus* sp. EB02 |
|  |  | *Halobacillus dabanensis* |
|  |  | *Bacillus* sp. SA1-12 |
|  |  | *Aneurinibacillus tyrosinisolvens* |
|  |  | *Bacillus aquimaris* |
|  |  | *Aneurinibacillus migulanus* |
|  |  | *Proteus vulgaris* |
|  |  | *Pediococcus pentosaceus* |
|  |  | *Ornithinibacillus halophilus* |
|  |  | *Massilibacterium senegalense* |
|  |  | *Salimicrobium flavidum* |
|  |  | *Pectobacterium carotovorum* |
|  |  | *Bacillus horneckiae* |
|  |  | *Paenibacillus odorifer* |
|  |  | *Coprobacillus* sp. 8_1_38FAA |
|  |  | *Bacillus flexus* |
|  |  | *Rugosibacter aromaticivorans* |
|  |  | *Bacillus dielmoensis* |
|  |  | *Bacillus horikoshii* |
|  |  | *Bacillus cohnii* |
|  |  | *Lactobacillus jensenii* |
|  |  | *Bacillus* sp. SDLI1 |
|  |  | *Paenibacillus durus* |
|  |  | *Paraliobacillus ryukyuensis* |
|  |  | *Oxobacter pfennigii* |
|  |  | *Salinicoccus halodurans* |
|  |  | *Bacillus sinesaloumensis* |
|  |  | *Bacillus stratosphericus* |
|  |  | *Virgibacillus proomii* |
|  |  | *Streptococcus salivarius* |
|  |  | *Lactobacillus ruminis* |
|  |  | *Bacillus bingmayongensis* |
|  |  | *Bacillus axarquiensis* |
|  |  | *Bacillus* sp. OxB-1 |
|  |  | *Terribacillus halophilus* |
|  |  | *Escherichia coli* |
|  |  | *Enterococcus canis* |
|  |  | *Bacillus* sp. FJAT-44921 |
|  |  | *Oceanobacillus caeni* |
|  |  | *Bacillus lonarensis* |
|  |  | *Clostridium akagii* |
|  |  | *Brevibacillus panacihumi* |
|  |  | *Yersinia enterocolitica* |
|  |  | *Paucibacter* sp. KCTC 42545 |
|  |  | *Virgibacillus dokdonensis* |
|  |  | *Kurthia* sp. Dielmo |
|  |  | *Paenibacillus* sp. IHBB 10380 |
|  |  | *Halolactibacillus halophilus* |
|  |  | *Anoxybacillus tepidamans* |
|  |  | *Virgibacillus halodenitrificans* |
|  |  | *Bacillus solani* |
|  |  | *Lysinibacillus* sp. F5 |
|  |  | *Caldibacillus debilis* |
|  |  | *Bacillus* sp. FJAT-18017 |
|  |  | *Oceanobacillus oncorhynchi* |
|  |  | *Clostridium magnum* |
|  |  | *Paenibacillus ginsengihumi* |
|  |  | *Lactobacillus reuteri* |
|  |  | *Bacillus mojavensis* |
|  |  | *Bacillus xiamenensis* |
|  |  | *Bacillus vallismortis* |
|  |  | *Bacillus* sp. KCTC 13219 |
|  |  | *Tepidibacillus decaturensis* |
|  |  | *Brevibacillus laterosporus* |
|  |  | *Mycobacterium abscessus* |
|  |  | *Bacillus sonorensis* |
|  |  | *Bacillus* sp. TS-2 |
|  |  | *Geobacillus kaustophilus* |
|  |  | *Rummeliibacillus stabekisii* |
|  |  | *Domibacillus antri* |
|  |  | *Pedosphaera parvula* |
|  |  | *Bacillus tequilensis* |
|  |  | *Amphibacillus sediminis* |
|  |  | *Oceanobacillus iheyensis* |
|  |  | *Jeotgalibacillus* soli Cunha et al. 2012 |
|  |  | *Sporolactobacillus terrae* |
|  |  | *Bacillus* sp. TH008 |
|  |  | *Paenibacillus* sp. 1ZS3-15 |
|  |  | *Enterobacter cloacae* |
|  |  | *Brevibacillus agri* |
|  |  | *Bacillus cytotoxicus* |
|  |  | *Paenibacillus dendritiformis* |
|  |  | *Oceanobacillus jeddahense* |
|  |  | *Gorillibacterium* sp. SN4 |
|  |  | *Novibacillus thermophilus* |
|  |  | *Paenibacillus riograndensis* |
|  |  | *Bacillus nealsonii* |
|  |  | *Oceanobacillus manasiensis* |
|  |  | *Viridibacillus* sp. FSL H7-0596 |
|  |  | *Bacillus vietnamensis* |
|  |  | *Shigella sonnei* |
|  |  | *Bacillus mesonae* |
|  |  | *Brevibacillus thermoruber* |
|  |  | *Lysinibacillus boronitolerans* |
|  |  | *Bacillus* sp. A053 |
|  |  | *Bacillus* sp. MUM 116 |
|  |  | *Bacillus* sp. B14905 |
|  |  | *Bacillus* sp. FJAT-14578 |
|  |  | *Clostridium* sp. DL-VIII |
|  |  | *Anoxybacillus* sp. UARK-01 |
|  |  | *Piscibacillus halophilus* |
|  |  | *Planococcus* sp. L10.15 |
|  |  | *Domibacillus tundrae* |
|  |  | *Bacillus* sp. Marseille-P2366 |
|  |  | *Bacillus* sp. LM 4-2 |
|  |  | *Bacillus azotoformans* |
|  |  | *Brevibacillus borstelensis* |
|  |  | *Bacillus fastidiosus* |
|  |  | *Thermoflavimicrobium dichotomicum* |
|  |  | *Pediococcus acidilactici* |
|  |  | *Bacillus oceanisediminis* |
|  |  | *Bacillus rubiinfantis* |
|  |  | *Oceanobacillus limi* |
|  |  | *Virgibacillus chiguensis* |
|  |  | *Bacillus eiseniae* |
|  |  | *Aneurinibacillus* sp. XH2 |
|  |  | *Zoogloea* sp. LCSB751 |
|  |  | *Corynebacterium stationis* |
|  |  | *Bacillus* sp. MRMR6 |
|  |  | *Bacillus soli* |
|  |  | *Bacillus simplex* |
|  |  | *Bacillus intestinalis* |
|  |  | *Sporosarcina newyorkensis* |
|  |  | *Bacillus* sp. 1NLA3E |
|  |  | *Bacillus pseudofirmus* |
|  |  | *Caenimonas* sp. SL110 |
|  |  | *Clostridium argentinense* |
|  |  | *Enterococcus mundtii* |
|  |  | *Vagococcus teuberi* |
|  |  | *Bacillus* sp. FJAT-25496 |
|  |  | *Oligella ureolytica* |
|  |  | *Salmonella enterica* |
|  |  | *Acinetobacter baumannii* |
|  |  | *Geobacillus stearothermophilus* |
|  |  | *Bacillus humi* |
|  |  | *Clostridium pasteurianum* |
|  |  | *Numidum massiliense* |
|  | | |
| India Nepal | 121 | *Acidibacillus ferrooxidans* |
|  |  | *Acidaminococcus intestini* |
|  |  | *Pseudoflavonifractor capillosus* |
|  |  | *Pseudobacteroides cellulosolvens* |
|  |  | *Rhodoferax fermentans* |
|  |  | *Drancourtella massiliensis* |
|  |  | *Paenibacillus pinihumi* |
|  |  | *Listeria innocua* |
|  |  | *Nitrospira defluvii* |
|  |  | *Gemmatimonas phototrophica* |
|  |  | *Clostridium cochlearium* |
|  |  | *Methylibium* sp. NZG |
|  |  | *Clostridium saccharobutylicum* |
|  |  | *Proteiniclasticum ruminis* |
|  |  | *Jeotgalicoccus halophilus* |
|  |  | *Pseudomonas psychrotolerans* |
|  |  | *Desulfuribacillus alkaliarsenatis* |
|  |  | *Nitrospira* sp. SCN 59-13 |
|  |  | *Lactococcus piscium* |
|  |  | *Lactobacillus suebicus* |
|  |  | *Trichococcus pasteurii* |
|  |  | *Paenibacillus amylolyticus* |
|  |  | *Halobacteroides halobius* |
|  |  | *Cohnella* sp. 6021052837 |
|  |  | *Chryseolinea serpens* |
|  |  | *Streptococcus infantarius* |
|  |  | *Exiguobacterium* sp. NG55 |
|  |  | *Sphaerotilus natans* |
|  |  | *Aneurinibacillus thermoaerophilus* |
|  |  | *Lentibacillus amyloliquefaciens* |
|  |  | *Hydrogenophaga flava* |
|  |  | *Bacillus* sp. NRRL B-41294 |
|  |  | *Staphylococcus cohnii* |
|  |  | *Anoxybacillus* sp. SK3-4 |
|  |  | *Carnobacterium iners* |
|  |  | *Bacillus* sp. 1310(2010) |
|  |  | *Legionella pneumophila* |
|  |  | *Paenibacillus* sp. DMB5 |
|  |  | *Lactobacillus sunkii* |
|  |  | *Rhodovulum* sp. PH10 |
|  |  | *Acetobacterium dehalogenans* |
|  |  | *Dysgonomonas mossii* |
|  |  | *Gracilibacillus kekensis* |
|  |  | *Streptomyces alboniger* |
|  |  | *Anoxybacillus suryakundensis* |
|  |  | *Chloroflexus aggregans* |
|  |  | *Merdibacter massiliensis* |
|  |  | *Sporomusa malonica* |
|  |  | *Sphingobacterium wenxiniae* |
|  |  | *Dechloromonas agitata* |
|  |  | *Thermicanus aegyptius* |
|  |  | *Rubinisphaera brasiliensis* |
|  |  | *Weissella hellenica* |
|  |  | *Trichococcus flocculiformis* |
|  |  | *Lactobacillus nodensis* |
|  |  | *Staphylococcus fleurettii* |
|  |  | *Bacillus trypoxylicola* |
|  |  | *Salibacterium qingdaonense* |
|  |  | *Staphylococcus carnosus* |
|  |  | *Bacillus* sp. MSP5.4 |
|  |  | *Nitrospira* sp. ND1 |
|  |  | *Cellulosilyticum* sp. I15G10I2 |
|  |  | *Bacillus* sp. CPSM8 |
|  |  | *Blastopirellula marina* |
|  |  | *Massilia niastensis* |
|  |  | *Alicyclobacillus ferrooxydans* |
|  |  | *Pontibacillus chungwhensis* |
|  |  | *Streptococcus marmotae* |
|  |  | *Porphyrobacter dokdonensis* |
|  |  | *Chthoniobacter flavus* |
|  |  | *Carnobacterium mobile* |
|  |  | *Prosthecobacter debontii* |
|  |  | *Paenibacillus alginolyticus* |
|  |  | *Bacillus* sp. 17376 |
|  |  | *Beduini massiliensis* |
|  |  | *Paenibacillus* sp. 1_12 |
|  |  | *Macrococcus caseolyticus* |
|  |  | *Paenibacillus* sp. VT-16-81 |
|  |  | *Staphylococcus gallinarum* |
|  |  | *Clostridium lundense* |
|  |  | *Cohnella thermotolerans* |
|  |  | *Kyrpidia tusciae* |
|  |  | *Clostridium* sp. KNHs205 |
|  |  | *Aquabacterium parvum* |
|  |  | *Bifidobacterium adolescentis* |
|  |  | *Paenibacillus algorifonticola* |
|  |  | *Paenibacillus* sp. IHB B 3084 |
|  |  | *Anoxybacillus* sp. 103 |
|  |  | *Ktedonobacter racemifer* |
|  |  | *Fusicatenibacter saccharivorans* |
|  |  | *Gemmatimonas* sp. SG8_23 |
|  |  | *Desulfotomaculum geothermicum* |
|  |  | *Dysgonomonas capnocytophagoides* |
|  |  | *Paracoccus chinensis* |
|  |  | *Desulfuromonas thiophila* |
|  |  | *Bacillus* sp. BS-02 |
|  |  | *Sulfuritalea hydrogenivorans* |
|  |  | *Staphylococcus vitulinus* |
|  |  | *Tepidibacillus* sp. HK-1 |
|  |  | *Clostridium carboxidivorans* |
|  |  | *Alkaliphilus transvaalensis* |
|  |  | *Nitrospira* sp. ST-bin5 |
|  |  | *Paenibacillus* sp. FF9 |
|  |  | *Thauera terpenica* |
|  |  | *Beggiatoa alba* |
|  |  | *Cohnella laeviribosi* |
|  |  | *Sunxiuqinia dokdonensis* |
|  |  | *Geothrix fermentans* |
|  |  | *Arcobacter butzleri* |
|  |  | *Citrobacter amalonaticus* |
|  |  | *Chitinophaga niabensis* |
|  |  | *Weissella jogaejeotgali* |
|  |  | *Eisenbergiella tayi* |
|  |  | *Mariniphaga anaerophila* |
|  |  | *Geobacillus* genomosp. 3 |
|  |  | *Anaeromassilibacillus senegalensis* |
|  |  | *Pelobacter carbinolicus* |
|  |  | *Bacillus* sp. RUPDJ |
|  |  | *Virgibacillus subterraneus* |
|  |  | *Clostridium tetani* |
|  |  | *Brevibacterium senegalense* |
|  | | |
| Bhutan India | 256 | *Vibrio parahaemolyticus* |
|  |  | *Microvirgula aerodenitrificans* |
|  |  | *Lactobacillus casei* |
|  |  | *Candidimonas bauzanensis* |
|  |  | *Erysipelothrix rhusiopathiae* |
|  |  | *Anaerocolumna xylanovorans* |
|  |  | *Carnobacterium* sp. AT7 |
|  |  | *Acinetobacter* sp. HR7 |
|  |  | *Acinetobacter towneri* |
|  |  | *Legionella massiliensis* |
|  |  | *Succinatimonas hippei* |
|  |  | *Thiomonas* sp. CB2 |
|  |  | *Acinetobacter* sp. SFC |
|  |  | *Planococcus rifietoensis* |
|  |  | *Morganella morganii* |
|  |  | *Myroides profundi* |
|  |  | *Gracilibacillus timonensis* |
|  |  | *Edwardsiella tarda* |
|  |  | *Paraburkholderia aspalathi* |
|  |  | *Corynebacterium jeikeium* |
|  |  | *Bacillus* sp. JKS001846 |
|  |  | *Lactobacillus ghanensis* |
|  |  | *Clostridium saccharoperbutylacetonicum* |
|  |  | *Erwinia tracheiphila* |
|  |  | *Marinospirillum minutulum* |
|  |  | *Enterococcus dispar* |
|  |  | *Bacillus* sp. GeD10 |
|  |  | *Pseudonocardia acaciae* |
|  |  | *Solibacillus isronensis* |
|  |  | *Moellerella wisconsensis* |
|  |  | *Sporosarcina* sp. P37 |
|  |  | *Oblitimonas alkaliphila* |
|  |  | *Pantoea ananatis* |
|  |  | *Providencia alcalifaciens* |
|  |  | *Bacteroides fragilis* |
|  |  | *Coxiella* sp. RIFCSPHIGHO2_12_FULL_42_15 |
|  |  | *Xylophilus ampelinus* |
|  |  | *Serratia odorifera* |
|  |  | *Oribacterium* sp. oral taxon 078 |
|  |  | *Pusillimonas noertemannii* |
|  |  | *Sphingobacterium mizutaii* |
|  |  | *Wohlfahrtiimonas* sp. 34C10-3-10 |
|  |  | *Stenotrophomonas* sp. MB339 |
|  |  | *Enterococcus italicus* |
|  |  | *Brackiella oedipodis* |
|  |  | *Sporosarcina* sp. ZBG7A |
|  |  | *Staphylococcus auricularis* |
|  |  | *Garciella nitratireducens* |
|  |  | *Paenisporosarcina quisquiliarum* |
|  |  | *Clostridium* sp. DMHC 10 |
|  |  | *Enterococcus moraviensis* |
|  |  | *Streptococcus uberis* |
|  |  | *Alkalibacterium putridalgicola* |
|  |  | *Ottowia thiooxydans* |
|  |  | *Lactobacillus agilis* |
|  |  | *Paenibacillus swuensis* |
|  |  | *Globicatella sulfidifaciens* |
|  |  | *Vagococcus* sp. D7T301 |
|  |  | *Eubacterium* sp. AB3007 |
|  |  | *Serratia marcescens* |
|  |  | *Planococcus massiliensis* |
|  |  | *Providencia rettgeri* |
|  |  | *Paenibacillus fonticola* |
|  |  | *Enterococcus aquimarinus* |
|  |  | *Lactobacillus murinus* |
|  |  | *Nitrosococcus watsonii* |
|  |  | *Streptococcus sobrinus* |
|  |  | *Clostridium tetanomorphum* |
|  |  | *Staphylococcus lentus* |
|  |  | *Castellaniella caeni* |
|  |  | *Providencia heimbachae* |
|  |  | *Jeotgalibaca dankookensis* |
|  |  | *Legionella waltersii* |
|  |  | *Lactobacillus plantarum* |
|  |  | *Aquamicrobium defluvii* |
|  |  | *Paenibacillus harenae* |
|  |  | *Lacticigenium naphtae* |
|  |  | *Caryophanon tenue* |
|  |  | *Planococcus plakortidis* |
|  |  | *Providencia sneebia* |
|  |  | *Flavobacterium indicum* |
|  |  | *Vagococcus penaei* |
|  |  | *Lachnoclostridium phytofermentans* |
|  |  | *Paenibacillus pasadenensis* |
|  |  | *Christensenella minuta* |
|  |  | *Morganella psychrotolerans* |
|  |  | *Enterococcus phoeniculicola* |
|  |  | *Paenibacillus* sp. yr247 |
|  |  | *Viridibacillus arvi* |
|  |  | *Sulfuricella denitrificans* |
|  |  | *Bacillus* sp. L_1B0_12 |
|  |  | *Hafnia alvei* |
|  |  | *Clostridium sulfidigenes* |
|  |  | *Thorsellia anophelis* |
|  |  | *Xenorhabdus bovienii* |
|  |  | *Paenibacillus* sp. OSY-SE |
|  |  | *Globicatella* sp. HMSC072A10 |
|  |  | *Helicobacter* sp. |
|  |  | *Providencia burhodogranariea* |
|  |  | *Ornithinibacillus californiensis* |
|  |  | *Leptothrix cholodnii* |
|  |  | *Marinomonas spartinae* |
|  |  | *Enterobacter hormaechei* |
|  |  | *Paenibacillus ferrarius* |
|  |  | *Bacillus* sp. FJAT-27916 |
|  |  | *Enterococcus canintestini* |
|  |  | *Methylibium* sp. CF059 |
|  |  | *Jeotgalicoccus marinus* |
|  |  | *Proteiniborus ethanoligenes* |
|  |  | *Domibacillus* sp. SAOS 44 |
|  |  | *Lactococcus garvieae* |
|  |  | *Corynebacterium variabile* |
|  |  | *Isobaculum melis* |
|  |  | *Nitrospira* sp. SG-bin1 |
|  |  | *Clostridium acetobutylicum* |
|  |  | *Pusillimonas* sp. T7-7 |
|  |  | *Bacillus* sp. FJAT-26652 |
|  |  | *Proteus* sp. H24 |
|  |  | *Enterococcus sulfureus* |
|  |  | *Salinicoccus roseus* |
|  |  | *Providencia rustigianii* |
|  |  | *Paenibacillus kribbensis* |
|  |  | *Serratia symbiotica* |
|  |  | *Thalassobacillus cyri* |
|  |  | *Klebsiella aerogenes* |
|  |  | *Lactobacillus wasatchensis* |
|  |  | *Corynebacterium ammoniagenes* |
|  |  | *Acetobacter syzygii* |
|  |  | *Myroides odoratimimus* |
|  |  | *Bacillus* sp. OK048 |
|  |  | *Pedobacter ruber* |
|  |  | *Sporolactobacillus laevolacticus* |
|  |  | *Globicatella sanguinis* |
|  |  | *Lentibacillus persicus* |
|  |  | *Clostridium neonatale* |
|  |  | *Halobacillus salinus* |
|  |  | *Gilliamella apicola* |
|  |  | *Enterococcus* sp. RIT-PI-f |
|  |  | *Bacillus zhangzhouensis* |
|  |  | *Lacunisphaera limnophila* |
|  |  | *Lactobacillus vaccinostercus* |
|  |  | *Bacillus* sp. RRD69 |
|  |  | *Rhizobacter gummiphilus* |
|  |  | *Clostridium puniceum* |
|  |  | *Oceanimonas* sp. GK1 |
|  |  | *Variovorax paradoxus* |
|  |  | *Acetobacter pasteurianus* |
|  |  | *Marinagarivorans algicola* |
|  |  | *Paenibacillus* sp. 11 |
|  |  | *Bacillus testis* |
|  |  | *Parabacteroides* sp. Marseille-P3160 |
|  |  | *Bacillus ndiopicus* |
|  |  | *Fictibacillus enclensis* |
|  |  | *Tolumonas auensis* |
|  |  | *Clostridium* sp. 12(A) |
|  |  | *Sporolactobacillus vineae* |
|  |  | *Bacillus hemicellulosilyticus* |
|  |  | *Klebsiella oxytoca* |
|  |  | *Bhargavaea beijingensis* |
|  |  | *Enterococcus termitis* |
|  |  | *Inquilinus limosus* |
|  |  | *Bacillus chagannorensis* |
|  |  | *Limnohabitans* sp. 103DPR2 |
|  |  | *Bacillus* sp. FJAT-22090 |
|  |  | *Planococcus donghaensis* |
|  |  | *Pelistega* sp. MC2 |
|  |  | *Alcaligenes faecalis* |
|  |  | *Beggiatoa* sp. IS2 |
|  |  | *Vitreoscilla* sp. SN6 |
|  |  | *Pasteurella multocida* |
|  |  | *Pseudomonas aeruginosa* |
|  |  | *Acinetobacter bereziniae* |
|  |  | *Enterococcus columbae* |
|  |  | *Acinetobacter johnsonii* |
|  |  | *Bacillus* sp. NRRL B-41282 |
|  |  | *Corynebacterium flavescens* |
|  |  | *Burkholderia ubonensis* |
|  |  | *Melissococcus plutonius* |
|  |  | *Clostridioides difficile* |
|  |  | *Orrella dioscoreae* |
|  |  | *Agrobacterium tumefaciens* |
|  |  | *Phocea massiliensis* |
|  |  | *Acidiphilium multivorum* |
|  |  | *Advenella mimigardefordensis* |
|  |  | *Alkalibacterium subtropicum* |
|  |  | *Acinetobacter pittii* |
|  |  | *Carnobacterium gallinarum* |
|  |  | *Brevibacillus* sp. OK042 |
|  |  | *Virgibacillus* *salinus* |
|  |  | *Virgibacillus* sp. CM-4 |
|  |  | *Taylorella equigenitalis* |
|  |  | *Aquaspirillum* sp. LM1 |
|  |  | *Enterococcus hirae* |
|  |  | *Proteus hauseri* |
|  |  | *Saccharibacillus sacchari* |
|  |  | *Tenuibacillus multivorans* |
|  |  | *Oxalobacter formigenes* |
|  |  | *Bacillus* sp. NRRL B-14911 |
|  |  | *Enterococcus* sp. HSIEG1 |
|  |  | *Acinetobacter* sp. WCHA34 |
|  |  | *Erysipelothrix tonsillarum* |
|  |  | *Bacillus* sp. FJAT-27251 |
|  |  | *Porphyrobacter* sp. LM 6 |
|  |  | *Staphylococcus xylosus* |
|  |  | *Microbulbifer* sp. HZ11 |
|  |  | *Jeotgalicoccus psychrophilus* |
|  |  | *Lachnoanaerobaculum saburreum* |
|  |  | *Sphingobacterium lactis* |
|  |  | *Holophaga foetida* |
|  |  | *Listeria fleischmannii* |
|  |  | *Cardiobacterium valvarum* |
|  |  | *Acinetobacter tandoii* |
|  |  | *Lysinibacillus* sp. AR18-8 |
|  |  | *Cosenzaea myxofaciens* |
|  |  | *Burkholderia cepacia* |
|  |  | *Bacillus* sp. FJAT-27231 |
|  |  | *Pseudobacteriovorax antillogorgiicola* |
|  |  | *Balneatrix alpica* |
|  |  | *Vibrio cholerae* |
|  |  | *Bibersteinia trehalosi* |
|  |  | *Lactobacillus vaginalis* |
|  |  | *Sporosarcina* sp. D27 |
|  |  | *Paraburkholderia megapolitana* |
|  |  | *Salinicoccus* sp. YB14-2 |
|  |  | *Bacillus* sp. JCM 19047 |
|  |  | *Planomicrobium glaciei* |
|  |  | *Paenibacillus* sp. Marseille-P2472 |
|  |  | *Flavobacterium psychrophilum* |
|  |  | *Maledivibacter halophilus* |
|  |  | *Lactobacillus oris* |
|  |  | *Acinetobacter radioresistens* |
|  |  | *Paenisporosarcina* sp. HGH0030 |
|  |  | *Erysipelothrix larvae* |
|  |  | *Desemzia incerta* |
|  |  | *Escherichia fergusonii* |
|  |  | *Bacillus* sp. NH11B |
|  |  | *Clostridium intestinale* |
|  |  | *Timonella senegalensis* |
|  |  | *Renibacterium salmoninarum* |
|  |  | *Bacillus marmarensis* |
|  |  | *Clostridium* sp. Marseille-P299 |
|  |  | *Hungatella hathewayi* |
|  |  | *Paenibacillus lactis* |
|  |  | *Staphylococcus epidermidis* |
|  |  | *Lysinibacillus contaminans* |
|  |  | *Paenibacillus glacialis* |
|  |  | *Sphingobacterium* sp. JB170 |
|  |  | *Lactobacillus farciminis* |
|  |  | *Gemella asaccharolytica* |
|  |  | *Acinetobacter* sp. SFB |
|  |  | *Enterococcus pallens* |
|  |  | *Marinilactibacillus psychrotolerans* |
|  |  | *Acinetobacter equi* |
|  |  | *Tetragenococcus muriaticus* |
|  |  | *Enterobacter mori* |
|  |  | *Fabibacter misakiensis* |
|  | | |
| Bhutan Nepal | 215 | *Bacillus* sp. 72 |
|  |  | *Planomicrobium okeanokoites* |
|  |  | *Polaromonas naphthalenivorans* |
|  |  | *Rubrivivax gelatinosus* |
|  |  | *Thalassobacillus devorans* |
|  |  | *Acinetobacter indicus* |
|  |  | *Bacillus luciferensis* |
|  |  | *Hathewaya proteolytica* |
|  |  | *Parageobacillus toebii* |
|  |  | *Paenibacillus ehimensis* |
|  |  | *Bacillus* sp. SJS |
|  |  | *Bacillus wiedmannii* |
|  |  | *Flavisolibacter ginsengisoli* |
|  |  | *Variovorax* sp. YR216 |
|  |  | *Bacillus kribbensis* |
|  |  | *Cloacibacterium normanense* |
|  |  | *Halobacillus kuroshimensis* |
|  |  | *Pelagirhabdus alkalitolerans* |
|  |  | *Methyloversatilis discipulorum* |
|  |  | *Paraglaciecola psychrophila* |
|  |  | *Lactobacillus brevis* |
|  |  | *Geobacillus icigianus* |
|  |  | *Clostridium amylolyticum* |
|  |  | *Haloferula* sp. BvORR071 |
|  |  | *Vaginella massiliensis* |
|  |  | *Paenibacillus pabuli* |
|  |  | *Myroides injenensis* |
|  |  | *Bacillus oleronius* |
|  |  | *Acinetobacter gerneri* |
|  |  | *Paenibacillus* sp. 32O-W |
|  |  | *Bacillus boroniphilus* |
|  |  | *Streptococcus equinus* |
|  |  | *Bacillus* sp. MKU004 |
|  |  | *Phormidesmis priestleyi* |
|  |  | *Fluviicola taffensis* |
|  |  | *Bacillus* sp. B25(2016b) |
|  |  | *Bacillus* sp. 491mf |
|  |  | *Empedobacter brevis* |
|  |  | *Saprospira grandis* |
|  |  | *Thermobacillus composti* |
|  |  | *Domibacillus robiginosus* |
|  |  | *Bacillus novalis* |
|  |  | *Rubrivivax* sp. SCN 70-15 |
|  |  | *Novispirillum itersonii* |
|  |  | *Bacillus* sp. JCM 19041 |
|  |  | *Rhodovibrio salinarum* |
|  |  | *Rhizobacter* sp. Root404 |
|  |  | *Bacillus solimangrovi* |
|  |  | *Paenisporosarcina* sp. TG-14 |
|  |  | *Acinetobacter kyonggiensis* |
|  |  | *Rhodococcus rhodochrous* |
|  |  | *Paenibacillus* sp. FSL H8-0548 |
|  |  | *Clostridium acetireducens* |
|  |  | *Fodinicurvata fenggangensis* |
|  |  | *Brevibacillus reuszeri* |
|  |  | *Clostridium* sp. 7_2_43FAA |
|  |  | *Bacillus* sp. JCM 19034 |
|  |  | *Bacillus litoralis* |
|  |  | *Bacillus* sp. HMSC76G11 |
|  |  | *Bacillus panaciterrae* |
|  |  | *Thermoactinomyces* sp. Gus2-1 |
|  |  | *Bacillus* sp. FJAT-25547 |
|  |  | *Streptococcus parauberis* |
|  |  | *Fictibacillus gelatini* |
|  |  | *Lautropia* sp. SCN 69-89 |
|  |  | *Hydrogenophaga palleronii* |
|  |  | *Paenibacillus* sp. SIT18 |
|  |  | *Brevibacillus choshinensis* |
|  |  | *Paenibacillus* sp. RIFOXYA1_FULL_44_5 |
|  |  | *Bellilinea caldifistulae* |
|  |  | *Bacillus abyssalis* |
|  |  | *Caldicoprobacter oshimai* |
|  |  | *Collinsella aerofaciens* |
|  |  | *Paenibacillus* sp. GP183 |
|  |  | *Pirellula* sp. SH-Sr6A |
|  |  | *Bacillus* sp. URHB0009 |
|  |  | *Bacillus* sp. 105MF |
|  |  | *Bacillus siamensis* |
|  |  | *Acinetobacter guillouiae* |
|  |  | *Fictibacillus arsenicus* |
|  |  | *Bacillus* sp. FJAT-25509 |
|  |  | *Carnobacterium* sp. CP1 |
|  |  | *Bacillus alcalophilus* |
|  |  | *Acinetobacter kookii* |
|  |  | *Stenotrophomonas maltophilia* |
|  |  | *Castellaniella defragrans* |
|  |  | *Bacillus* sp. Soil745 |
|  |  | *Desulfovibrio inopinatus* |
|  |  | *Lactobacillus coleohominis* |
|  |  | *Brevibacillus* sp. BC25 |
|  |  | *Bacillus* sp. CDB3 |
|  |  | *Geobacillus thermoleovorans* |
|  |  | *Bartonella tamiae* |
|  |  | *Francisella tularensis* |
|  |  | *Enterococcus avium* |
|  |  | *Thioploca ingrica* |
|  |  | *Exiguobacterium aurantiacum* |
|  |  | *Fictibacillus solisalsi* |
|  |  | *Bacillus firmus* |
|  |  | *Paenibacillus wulumuqiensis* |
|  |  | *Tepidanaerobacter syntrophicus* |
|  |  | *Pseudomonas indica* |
|  |  | *Gottschalkia acidurici* |
|  |  | *Desulfosporosinus lacus* |
|  |  | *Clostridium* sp. HMP27 |
|  |  | *Pseudoalteromonas luteoviolacea* |
|  |  | *Bordetella holmesii* |
|  |  | *Hylemonella gracilis* |
|  |  | *Bacillus* sp. V-88 |
|  |  | *Desulfotomaculum gibsoniae* |
|  |  | *Catalinimonas alkaloidigena* |
|  |  | *Sediminispirochaeta bajacaliforniensis* |
|  |  | *Bacillus* sp. FJAT-27215 |
|  |  | *Thauera phenylacetica* |
|  |  | *Paenibacillus* sp. NAIST15-1 |
|  |  | *Paenibacillus glucanolyticus* |
|  |  | *Bacillus aerophilus* |
|  |  | *Treponema socranskii* |
|  |  | *Rubeoparvulum massiliense* |
|  |  | *Enterococcus pseudoavium* |
|  |  | *Flavobacterium succinicans* |
|  |  | *Bacillus wakoensis* |
|  |  | *Bacillus malacitensis* |
|  |  | *Geobacillus thermodenitrificans* |
|  |  | *Fictibacillus* sp. FJAT-27399 |
|  |  | *Arachidicoccus* sp. BS20 |
|  |  | *Bacillus* sp. 5B6 |
|  |  | *Clostridium saudiense* |
|  |  | *Leclercia adecarboxylata* |
|  |  | *Paenibacillus taiwanensis* |
|  |  | *Paenibacillus sanguinis* |
|  |  | *Effusibacillus pohliae* |
|  |  | *Giesbergeria anulus* |
|  |  | *Clostridium butyricum* |
|  |  | *Acinetobacter* sp. P8-3-8 |
|  |  | *Clostridium* sp. Bc-iso-3 |
|  |  | *Polaromonas* sp. JS666 |
|  |  | *Listeria rocourtiae* |
|  |  | *Rhodoferax antarcticus* |
|  |  | *Paenibacillus stellifer* |
|  |  | *Clostridium scatologenes* |
|  |  | *Paenibacillus antibioticophila* |
|  |  | *Oceanobacillus* sp. E9 |
|  |  | *Intrasporangium oryzae* |
|  |  | *Hyalangium minutum* |
|  |  | *Bacillus* sp. LL01 |
|  |  | *Clostridium* sp. ASBs410 |
|  |  | *Alicyclobacillus acidoterrestris* |
|  |  | *Paenibacillus phocaensis* |
|  |  | *Planococcus maritimus* |
|  |  | *Bacillus* sp. FJAT-27264 |
|  |  | *Acetobacterium bakii* |
|  |  | *Bacillus* sp. NSP2.1 |
|  |  | *Weissella oryzae* |
|  |  | *Alicyclobacillus shizuokensis* |
|  |  | *Clostridium cellulovorans* |
|  |  | *Acholeplasma oculi* |
|  |  | *Clostridium perfringens* |
|  |  | *Paenibacillus* sp. DMB20 |
|  |  | *Arthrobacter* sp. W1 |
|  |  | *Paenibacillus macquariensis* |
|  |  | *Bacillus pseudomycoides* |
|  |  | *Acinetobacter* sp. 983759 |
|  |  | *Yersinia pestis* |
|  |  | *Alkaliphilus metalliredigens* |
|  |  | *Acinetobacter seifertii* |
|  |  | *Bacillus* sp. YP1 |
|  |  | *Aquitalea magnusonii* |
|  |  | *Clostridium oryzae* |
|  |  | *Sediminibacillus albus* |
|  |  | *Acinetobacter lwoffii* |
|  |  | *Acinetobacter* sp. ANC 5600 |
|  |  | *Aquabacterium* sp. NJ1 |
|  |  | *Bacillus* sp. FJAT-27245 |
|  |  | *Pontibacillus marinus* |
|  |  | *Chitinophaga filiformis* |
|  |  | *Geobacillus* sp. 46C-IIa |
|  |  | *Roseimaritima ulvae* |
|  |  | *Desulfitobacterium hafniense* |
|  |  | *Thauera* sp. ZV-1-C |
|  |  | *Bacillus* sp. 2_A_57_CT2 |
|  |  | *Steroidobacter denitrificans* |
|  |  | *Trichococcus ilyis* |
|  |  | *Pseudomonas xanthomarina* |
|  |  | *Bacillus mannanilyticus* |
|  |  | *Nitrospira* sp. HN-bin3 |
|  |  | *Bacillus* sp. FJAT-29814 |
|  |  | *Anoxybacillus pushchinoensis* |
|  |  | *Brevibacillus* sp. Leaf182 |
|  |  | *Bacillus shacheensis* |
|  |  | *Rhodobacter* sp. CACIA14H1 |
|  |  | *Bacillus halmapalus* |
|  |  | *Bacillus pseudalcaliphilus* |
|  |  | *Bacillus massiliogorillae* |
|  |  | *Brevibacillus* sp. CF112 |
|  |  | *Mucilaginibacter paludis* |
|  |  | *Desulfuribacillus stibiiarsenatis* |
|  |  | *Enterobacter asburiae* |
|  |  | *Polynucleobacter* sp. VK13 |
|  |  | *Bacillus* sp. 522_BSPC |
|  |  | *Paenibacillus pini* |
|  |  | *Salipaludibacillus aurantiacus* |
|  |  | *Comamonas terrae* |
|  |  | *Domibacillus enclensis* |
|  |  | *Paenibacillus rhizosphaerae* |
|  |  | *Domibacillus iocasae* |
|  |  | *Carnobacterium alterfunditum* |
|  |  | *Paenibacillus* sp. FJAT-26967 |
|  |  | *Eremococcus coleocola* |
|  |  | *Paenibacillus beijingensis* |
|  |  | *Brevibacillus massiliensis* |
|  |  | *Clostridium* sp. DSM 8431 |
|  |  | *Acinetobacter seohaensis* |
|  |  | *Erwinia typographi* |
|  |  | *Prevotella paludivivens* |
|  | | |
| India | 56 | *Bacillus* sp. H15-1 |
|  |  | *Solibacillus silvestris* |
|  |  | *Staphylococcus agnetis* |
|  |  | *Acinetobacter* sp. NIPH 2100 |
|  |  | *Cellulosilyticum ruminicola* |
|  |  | *Brachybacterium faecium* |
|  |  | *Staphylococcus equorum* |
|  |  | *Clostridium frigidicarnis* |
|  |  | *Jeotgalicoccus saudimassiliensis* |
|  |  | *Lactococcus chungangensis* |
|  |  | *Yaniella halotolerans* |
|  |  | *Staphylococcus* sp. MB371 |
|  |  | *Staphylococcus pasteuri* |
|  |  | *Enteractinococcus helveticum* |
|  |  | *Enterococcus ureasiticus* |
|  |  | *Staphylococcus* sp. HMSC13A10 |
|  |  | *Faecalibacterium prausnitzii* |
|  |  | *Leuconostoc mesenteroides* |
|  |  | *Facklamia ignava* |
|  |  | *Oligella* sp. HMSC09E12 |
|  |  | *Paracoccus tibetensis* |
|  |  | *Chishuiella changwenlii* |
|  |  | *Staphylococcus haemolyticus* |
|  |  | *Nafulsella turpanensis* |
|  |  | *Veillonella magna* |
|  |  | *Desulfurella acetivorans* |
|  |  | *Psychrobacillus psychrotolerans* |
|  |  | *Bacillus infantis* |
|  |  | *Gulbenkiania mobilis* |
|  |  | *Paenibacillus* sp. NFR01 |
|  |  | *Peptoniphilus* sp. KHD4 |
|  |  | *Clostridium acidisoli* |
|  |  | *Lactobacillus paralimentarius* |
|  |  | *Methyloceanibacter marginalis* |
|  |  | *Lactobacillus helveticus* |
|  |  | *Ideonella* sp. B508-1 |
|  |  | *Salinibacillus kushneri* |
|  |  | *Paraclostridium bifermentans* |
|  |  | *Salinicoccus qingdaonensis* |
|  |  | *Photobacterium aphoticum* |
|  |  | *Pseudorhodobacter psychrotolerans* |
|  |  | *Thermoflexibacter ruber* |
|  |  | *Yersinia aldovae* |
|  |  | *Serratia nematodiphila* |
|  |  | *Alicyclobacillus vulcanalis* |
|  |  | *Bacillus* sp. CBA7126 |
|  |  | *Methyloversatilis universalis* |
|  |  | *Rheinheimera texasensis* |
|  |  | *Marinilabilia salmonicolor* |
|  |  | *Cephaloticoccus primus* |
|  |  | *Halomonas utahensis* |
|  |  | *Geobacter* sp. OR-1 |
|  |  | *Staphylococcus warneri* |
|  |  | *Dokdonella koreensis* |
|  |  | *Anaerorhabdus furcosa* |
|  |  | *Marinospirillum insulare* |
|  | | |
| Nepal | 95 | *Allofustis seminis* |
|  |  | *Halobacillus halophilus* |
|  |  | *Acinetobacter* sp. CIP-A165 |
|  |  | *Terribacillus aidingensis* |
|  |  | *Marinococcus halophilus* |
|  |  | *Kocuria* sp. CNJ-770 |
|  |  | *Desulfitobacterium metallireducens* |
|  |  | *Clostridium aceticum* |
|  |  | *Pontibacter indicus* |
|  |  | *Clostridium* sp. N3C |
|  |  | *Leptolyngbya boryana* |
|  |  | *Geobacillus zalihae* |
|  |  | *Bacillus* sp. UNCCL81 |
|  |  | *Polaromonas* sp. CG9_12 |
|  |  | *Epulopiscium* sp. SCG-C07WGA-EpuloA2 |
|  |  | *Roseivirga spongicola* |
|  |  | *Geobacillus* sp. Sah69 |
|  |  | *Pseudomonas japonica* |
|  |  | *Paenibacillus* sp. FSL R7-0273 |
|  |  | *Geobacillus* sp. B4113_201601 |
|  |  | *Paenibacillus* sp. BIHB4019 |
|  |  | *Caulobacter vibrioides* |
|  |  | *Paenibacillus borealis* |
|  |  | *Bacteroides helcogenes* |
|  |  | *Streptomyces laurentii* |
|  |  | *Geobacillus* sp. 44C |
|  |  | *Geobacillus thermocatenulatus* |
|  |  | *Clostridium tyrobutyricum* |
|  |  | *Paenibacillus* sp. FSL R7-277 |
|  |  | *Lactobacillus curvatus* |
|  |  | *Anoxybacillus kamchatkensis* |
|  |  | *Sandaracinus amylolyticus* |
|  |  | *Desulfitobacterium dichloroeliminans* |
|  |  | *Beggiatoa* sp. PS |
|  |  | *Gracilibacillus lacisalsi* |
|  |  | *Bacillus* sp. Pc3 |
|  |  | *Dolosigranulum pigrum* |
|  |  | *Bacillus* sp. ECU0013 |
|  |  | *Parageobacillus* genomosp. 1 |
|  |  | *Geobacillus* sp. A8 |
|  |  | *Selenomonas bovis* |
|  |  | *Anoxybacillus* sp. KU2-6(11) |
|  |  | *Cellvibrio mixtus* |
|  |  | *Microcystis aeruginosa* |
|  |  | *Paenibacillus* sp. UNC499MF |
|  |  | *Anaerobacillus alkalilacustris* |
|  |  | *Alicyclobacillus* sp. RIFOXYA1_FULL_53_8 |
|  |  | *Geobacillus* sp. 15 |
|  |  | *Blautia producta* |
|  |  | *Bacillus* sp. FJAT-26390 |
|  |  | *Chthonomonas calidirosea* |
|  |  | *Paenibacillus* sp. 32352 |
|  |  | *Geobacillus jurassicus* |
|  |  | *Nostoc punctiforme* |
|  |  | *Exiguobacterium* sp. Leaf196 |
|  |  | *Ideonella sakaiensis* |
|  |  | *Hydrocarboniphaga daqingensis* |
|  |  | *Clostridium* sp. BL8 |
|  |  | *Syntrophomonas zehnderi* |
|  |  | *Lewinella cohaerens* |
|  |  | *Haliea salexigens* |
|  |  | *Caldanaerobacter subterraneus* |
|  |  | *Paenibacillus* sp. FSL H7-0331 |
|  |  | *Acaryochloris marina* |
|  |  | *Paenibacillus* sp. LC231 |
|  |  | *Desulfuromonas* sp. TF |
|  |  | *Rubrobacter xylanophilus* |
|  |  | *Anoxybacillus* sp. DT3-1 |
|  |  | *Bacillus halodurans* |
|  |  | *Geobacillus* sp. Y4.1MC1 |
|  |  | *Tepidimicrobium xylanilyticum* |
|  |  | *Pontibacter roseus* |
|  |  | *Geobacter lovleyi* |
|  |  | *Sunxiuqinia elliptica* |
|  |  | *Thermoactinomyces* sp. CDF |
|  |  | *Kroppenstedtia eburnea* |
|  |  | *Propionispora* sp. 2/2-37 |
|  |  | *Thiorhodovibrio* sp. 970 |
|  |  | *Chryseobacterium gleum* |
|  |  | *Anoxybacillus* sp. P3H1B |
|  |  | *Salimicrobium halophilum* |
|  |  | *Planctomyces* sp. SH-PL14 |
|  |  | *Planctomicrobium piriforme* |
|  |  | *Nitrosomonas* sp. Nm58 |
|  |  | *Bryobacter aggregatus* |
|  |  | *Dietzia cinnamea* |
|  |  | *Bifidobacterium kashiwanohense* |
|  |  | *Weissella thailandensis* |
|  |  | *Streptomyces rubidus* |
|  |  | *Geobacillus* sp. G11MC16 |
|  |  | *Thermoactinomyces* sp. AS95 |
|  |  | *Aneurinibacillus soli* |
|  |  | *Aquincola tertiaricarbonis* |
|  |  | *Geobacillus subterraneus* |
|  |  | *Geobacillus* sp. 44B |
|  | | |
| Bhutan | 229 | *Sulfurifustis variabilis* |
|  |  | *Klebsiella* sp. RIT-PI-d |
|  |  | *Facklamia miroungae* |
|  |  | *Acinetobacter calcoaceticus* |
|  |  | *Myroides* sp. ZB35 |
|  |  | *Bacillus* sp. N35-10-4 |
|  |  | *Thiobacillus denitrificans* |
|  |  | *Oceanospirillum beijerinckii* |
|  |  | *Myroides odoratus* |
|  |  | *Bacillus* sp. 166amftsu |
|  |  | *Acinetobacter brisouii* |
|  |  | *Neglecta timonensis* |
|  |  | *Halonatronum saccharophilum* |
|  |  | *Oceaniovalibus guishaninsula* |
|  |  | *Alicyclobacillus herbarius* |
|  |  | *Pilibacter termitis* |
|  |  | *Bordetella avium* |
|  |  | *Xanthomonas citri* |
|  |  | *Salimicrobium album* |
|  |  | *Janthinobacterium lividum* |
|  |  | *Thermoanaerobacterium thermosaccharolyticum* |
|  |  | *Acinetobacter* sp. MN12 |
|  |  | *Bacillus* sp. 123MFChir2 |
|  |  | *Comamonas kerstersii* |
|  |  | *Cohnella kolymensis* |
|  |  | *Bacillus* sp. 5mfcol3.1 |
|  |  | *Staphylococcus capitis* |
|  |  | *Myroides xuanwuensis* |
|  |  | *Clostridium* sp. Maddingley MBC34-26 |
|  |  | *Paenibacillus* sp. Soil750 |
|  |  | *Thermoactinomyces* sp. DSM 45892 |
|  |  | *Tetragenococcus solitarius* |
|  |  | *Bacillus* sp. ru9509.4 |
|  |  | *Bacillus* sp. 7_6_55CFAA_CT2 |
|  |  | *Sphingobacterium paucimobilis* |
|  |  | *Acinetobacter rudis* |
|  |  | *Citrobacter freundii* |
|  |  | *Geomicrobium* sp. JCM 19038 |
|  |  | *Bacillus* sp. G1(2015b) |
|  |  | *Bacillus* sp. ok061 |
|  |  | *Enterococcus* sp. HMSC076E04 |
|  |  | *Acinetobacter* sp. ANC 4945 |
|  |  | *Bacillus australimaris* |
|  |  | *Lactobacillus gasseri* |
|  |  | *Mageeibacillus indolicus* |
|  |  | *Aggregatibacter actinomycetemcomitans* |
|  |  | *Corynebacterium capitovis* |
|  |  | *Acinetobacter gandensis* |
|  |  | *Acinetobacter tjernbergiae* |
|  |  | *Taylorella asinigenitalis* |
|  |  | *Salsuginibacillus kocurii* |
|  |  | *Listeria floridensis* |
|  |  | *Altererythrobacter marensis* |
|  |  | *Arthrobacter halophytocola* |
|  |  | *Flavobacterium marinum* |
|  |  | *Proteus penneri* |
|  |  | *Immundisolibacter cernigliae* |
|  |  | *Bordetella petrii* |
|  |  | *Acinetobacter* sp. WCHA60 |
|  |  | *Sphingobacterium* sp. T2 |
|  |  | *Halothiobacillus* sp. LS2 |
|  |  | *Adhaeribacter aquaticus* |
|  |  | *Desulfonatronum thiodismutans* |
|  |  | *Acinetobacter* sp. MDS7A |
|  |  | *Enterobacter cancerogenus* |
|  |  | *Bacillus* sp. NH24A2 |
|  |  | *Lactobacillus paracasei* |
|  |  | *Acinetobacter* sp. CIP 51.11 |
|  |  | *Phaseolibacter flectens* |
|  |  | *Corynebacterium lubricantis* |
|  |  | *Sphingopyxis flava* |
|  |  | *Bacillus* sp. Leaf75 |
|  |  | *Bacillus* sp. TH007 |
|  |  | *Acinetobacter* sp. SFA |
|  |  | *Bacillus rhizosphaerae* |
|  |  | *Laribacter* *hongkongensis* |
|  |  | *Bacillus* sp. N35-10-2 |
|  |  | *Neisseria arctica* |
|  |  | *Brochothrix campestris* |
|  |  | *Acinetobacter* sp. ANC 4149 |
|  |  | *Advenella kashmirensis* |
|  |  | *Acinetobacter boissieri* |
|  |  | *Chitinophaga pinensis* |
|  |  | *Paenibacillus* sp. CF384 |
|  |  | *Bacillus* sp. ABP14 |
|  |  | *Lactobacillus acetotolerans* |
|  |  | *Lactobacillus* sp. HMSC24D01 |
|  |  | *Bordetella* sp. N |
|  |  | *Bacillus* sp. FJAT-27238 |
|  |  | *Corynebacterium diphtheriae* |
|  |  | *Bordetella ansorpii* |
|  |  | *Bacillus* sp. GZT |
|  |  | *Alcanivorax hongdengensis* |
|  |  | *Paenibacillus* sp. HW567 |
|  |  | *Sphingobacterium thalpophilum* |
|  |  | *Paenibacillus ihumii* |
|  |  | *Acinetobacter bouvetii* |
|  |  | *Acinetobacter ursingii* |
|  |  | *Paenibacillus* sp. TCA20 |
|  |  | *Bacillus* sp. RUTrin4 |
|  |  | *Beijerinckia mobilis* |
|  |  | *Lactobacillus hokkaidonensis* |
|  |  | *Acinetobacter celticus* |
|  |  | *Paenibacillus assamensis* |
|  |  | *Brucella suis* |
|  |  | *Rubellimicrobium thermophilum* |
|  |  | *Shigella flexneri* |
|  |  | *Pseudomonas resinovorans* |
|  |  | *Herminiimonas arsenicoxydans* |
|  |  | *Bacillus* sp. bc15 |
|  |  | *Carnobacterium divergens* |
|  |  | *Anaerobacillus macyae* |
|  |  | *Corynebacterium phocae* |
|  |  | *Ruminococcus bromii* |
|  |  | *Proteus* sp. HMSC10D02 |
|  |  | *Corynebacterium kroppenstedtii* |
|  |  | *Paenibacillus* sp. GM2 |
|  |  | *Azohydromonas lata* |
|  |  | *Acinetobacter* sp. 1294596 |
|  |  | *Intestinibacter bartlettii* |
|  |  | *Acinetobacter* sp. CIP 102136 |
|  |  | *Algiphilus aromaticivorans* |
|  |  | *Peptoniphilus indolicus* |
|  |  | *Acinetobacter* sp. TTH0-4 |
|  |  | *Xanthomonas arboricola* |
|  |  | *Lacinutrix* sp. Hel_I_90 |
|  |  | *Flavobacterium ummariense* |
|  |  | *Duganella phyllosphaerae* |
|  |  | *Geomicrobium* sp. JCM 19055 |
|  |  | *Vitreoscilla stercoraria* |
|  |  | *Eubacterium* sp. ER2 |
|  |  | *Escherichia albertii* |
|  |  | *Paenibacillus* sp. A9 |
|  |  | *Calditerricola satsumensis* |
|  |  | *Pluralibacter gergoviae* |
|  |  | *Corynebacterium camporealensis* |
|  |  | *Variovorax* sp. CF313 |
|  |  | *Acinetobacter* sp. CIP 101966 |
|  |  | *Bordetella* genomosp. 13 |
|  |  | *Clostridium baratii* |
|  |  | *Virgibacillus massiliensis* |
|  |  | *Streptococcus pyogenes* |
|  |  | *Amphibacillus marinus* |
|  |  | *Bacillus altitudinis* |
|  |  | *Psychrobacillus psychrodurans* |
|  |  | *Acinetobacter* sp. MF4642 |
|  |  | *Haemophilus influenzae* |
|  |  | *Pseudospirillum japonicum* |
|  |  | *Bacillus* sp. MN5 |
|  |  | *Streptococcus mitis* |
|  |  | *Ruminiclostridium thermocellum* |
|  |  | *Basilea psittacipulmonis* |
|  |  | *Enterococcus rivorum* |
|  |  | *Bacillus patagoniensis* |
|  |  | *Skermanella stibiiresistens* |
|  |  | *Psychrobacter* sp. AntiMn-1 |
|  |  | *Bacillus* sp. TD42 |
|  |  | *Lactobacillus harbinensis* |
|  |  | *Dehalobacter* sp. E1 |
|  |  | *Paenibacillus* sp. RU5A |
|  |  | *Bacillus* sp. M 2-6 |
|  |  | *Sporanaerobacter* sp. PP17-6a |
|  |  | *Flavobacterium terrigena* |
|  |  | *Acidovorax* sp. 12322-1 |
|  |  | *Myroides guanonis* |
|  |  | *Pseudonocardia spinosispora* |
|  |  | *Lactobacillus* sp. HMSC08B12 |
|  |  | *Clostridium haemolyticum* |
|  |  | *Aurantimonas* sp. 22II-16-19i |
|  |  | *Lysinibacillus* sp. LK3 |
|  |  | *Corynebacterium* sp. CNJ-954 |
|  |  | *Acinetobacter haemolyticus* |
|  |  | *Bacillus* sp. N24 |
|  |  | *Granulicatella balaenopterae* |
|  |  | *Bacillus* sp. WP8 |
|  |  | *Paenibacillus* sp. Soil522 |
|  |  | *Thauera butanivorans* |
|  |  | *Ochrobactrum intermedium* |
|  |  | *Enterococcus silesiacus* |
|  |  | *Leucothrix mucor* |
|  |  | *Blattabacterium punctulatus* |
|  |  | *Salinimicrobium xinjiangense* |
|  |  | *Bacillus* sp. G3(2015) |
|  |  | *Bordetella pertussis* |
|  |  | *Enterococcus haemoperoxidus* |
|  |  | *Thermotalea metallivorans* |
|  |  | *Rhodovulum* sp. ES.010 |
|  |  | *Peptoniphilus* sp. oral taxon 375 |
|  |  | *Myroides* sp. A21 |
|  |  | *Bacillus selenatarsenatis* |
|  |  | *Lactobacillus crispatus* |
|  |  | *Dethiobacter alkaliphilus* |
|  |  | *Lactobacillus ingluviei* |
|  |  | *Achromobacter* sp. DMS1 |
|  |  | *Shewanella* sp. POL2 |
|  |  | *Corynebacterium provencense* |
|  |  | *Parapedobacter luteus* |
|  |  | *Salegentibacter mishustinae* |
|  |  | *Proteus* sp. 3M |
|  |  | *Emticicia oligotrophica* |
|  |  | *Acinetobacter* sp. 51m |
|  |  | *Mycobacterium kansasii* |
|  |  | *Enterococcus villorum* |
|  |  | *Acinetobacter idrijaensis* |
|  |  | *Bacillus* sp. TD41 |
|  |  | *Kluyvera cryocrescens* |
|  |  | *Lactobacillus fuchuensis* |
|  |  | *Pseudomonas* sp. TTU2014-080ASC |
|  |  | *Albidiferax* sp. OV413 |
|  |  | *Acinetobacter nosocomialis* |
|  |  | *Acinetobacter schindleri* |
|  |  | *Bacillus* sp. LK2 |
|  |  | *Bacillus* sp. FJAT-21955 |
|  |  | *Paenibacillus catalpae* |
|  |  | *Lactobacillus dextrinicus* |
|  |  | *Myroides marinus* |
|  |  | *Capnocytophaga cynodegmi* |
|  |  | *Acinetobacter* sp. NCu2D-2 |
|  |  | *Desulfobulbus elongatus* |
|  |  | *Flavobacterium rivuli* |
|  |  | *Bacillus* sp. 0711P9-1 |
|  |  | *Lactobacillus johnsonii* |
|  |  | *Enterococcus malodoratus* |
|  |  | *Clostridium* sp. CL-2 |
|  |  | *Bacillus* sp. H1a |
|  |  | *Bacillus* sp. UMTAT18 |
|  |  | *Zavarzinella formosa* |
|  |  | *Bacillus* sp. Leaf49 |
|  |  | *Clostridium tunisiense* |

| **Supplementary Table 12: Shared and unique viruses in *kinema*.** | | |
| --- | --- | --- |
| Sample site | No. of species | Viral species |
| Bhutan, India, Nepal | 4 | *Bacillus* phage Shbh1 |
|  |  | *Bacillus* phage SP-10 |
|  |  | *Bacillus* phage SPG24 |
|  |  | *Bacillus* phage SPP1 |
|  | | |
| India Nepal | 10 | *Bacillus* phage Basilisk |
|  |  | *Bacillus* phage BSNPO1 |
|  |  | *Bacillus* phage Mgbh1 |
|  |  | *Bacillus* phage SIOphi |
|  |  | *Bacillus* phage vB_BanS-Tsamsa |
|  |  | *Bacillus* phage Bobb |
|  |  | *Bacillus* phage Grass |
|  |  | *Bacillus* phage phiNIT1 |
|  |  | *Bacillus* phage PBC4 |
|  |  | *Bacillus* phage PM1 |
|  | | |
| India | 13 | *Mycobacterium* phage Idleandcovert |
|  |  | *Enterococcus* phage IME-EFm5 |
|  |  | *Bacillus* phage 1102phi1-3 |
|  |  | *Bacillus* phage BCJA1c |
|  |  | *Bacillus* phage BCP8-2 |
|  |  | *Staphylococcus* phage Stau2 |
|  |  | *Staphylococcus* phage BP39 |
|  |  | *Staphylococcus* phage Andhra |
|  |  | *Streptococcus* phage C1 |
|  |  | *Pseudoalteromonas* phage H105/1 |
|  |  | *Cellulophaga* phage phi18:1 |
|  |  | *Geobacillus* virus E2 |
|  |  | *Bacillus* phage Eldridge |
|  | | |
| Nepal | 5 | *Bacillus* phage QCM8 |
|  |  | *Aeribacillus* phage AP45 |
|  |  | *Geobacillus* phage GBSV1 |
|  |  | *Enterococcus* phage EFDG1 |
|  |  | *Geobacillus* virus E3 |
|  | | |
| Bhutan | 10 | *Bacillus* phage 0305phi8-36 |
|  |  | *Lactobacillus* phage phiPYB5 |
|  |  | *Bacillus* virus G |
|  |  | *Bacillus* virus 1 |
|  |  | *Bacillus* virus Andromeda |
|  |  | *Pseudoalteromonas* phage vB_PspS-H40/1 |
|  |  | *Bacillus* phage Stahl |
|  |  | *Vibrio* phage ICP1 |
|  |  | *Bacillus* phage PBC1 |
|  |  | *Bacillus* phage BCD7 |
