# Supplementary material for: Metagenomic-Metabolomic Mining of Kinema, a Naturally Fermented Soybean Food of the Eastern Himalayas
Source: Front Microbiol. 2022 Apr 29;13:868383. doi: 10.3389/fmicb.2022.868383 (PMC9106393; doi:10.3389/fmicb.2022.868383)
Supplement: Supplementary file 7 [file Table_7.DOCX]

| **Supplementary Table 13\| Shared and unique eukaryotic species in *kinema*.** | | |
| --- | --- | --- |
| Sample site | No. of species | Eukaryotic species |
| Bhutan, India, Nepal | 2 | *Pichia kudriavzevii* |
|  |  | *Mucor ambiguus* |
|  | | |
| India Nepal | 4 | *Ichthyophthirius multifiliis* |
|  |  | *Trichosporon asahii* |
|  |  | *Batrachochytrium dendrobatidis* |
|  |  | *Pneumocystis jirovecii* |
|  | | |
| Bhutan India | 4 | *Stylonychia lemnae* |
|  |  | *Acanthamoeba castellanii* |
|  |  | *Kuraishia capsulata* |
|  |  | *Oxytricha trifallax* |
|  | | |
| Bhutan Nepal | 1 | *Thalassiosira oceanica* |
|  | | |
| India | 27 | *Pyronema omphalodes* |
|  |  | *Acetabularia acetabulum* |
|  |  | *Geotrichum candidum* |
|  |  | *Plasmodiophora brassicae* |
|  |  | *Lichtheimia ramosa* |
|  |  | *Candida subhashii* |
|  |  | *Candida orthopsilosis* |
|  |  | *Fragilariopsis cylindrus* |
|  |  | *Mucor circinelloides* |
|  |  | *Candida tanzawaensis* |
|  |  | *Nannochloropsis gaditana* |
|  |  | *Cyberlindnera jadinii* |
|  |  | *Stentor coeruleus* |
|  |  | *Candida albicans* |
|  |  | *Chlorella variabilis* |
|  |  | *Acidomyces richmondensis* |
|  |  | *Hypsizygus marmoreus* |
|  |  | *Galdieria sulphuraria* |
|  |  | *Metschnikowia bicuspidata* |
|  |  | *Debaryomyces fabryi* |
|  |  | *Spathaspora passalidarum* |
|  |  | *Tetrahymena thermophila* |
|  |  | *Exophiala aquamarina* |
|  |  | *Leucosporidium creatinivorum* |
|  |  | *Candida sake* |
|  |  | *Meyerozyma guilliermondii* |
|  |  | *Candida maltosa* |
|  | | |
| Nepal | 8 | *Trypanosoma brucei* |
|  |  | *Puccinia striiformis* |
|  |  | *Sclerotinia sclerotiorum* |
|  |  | *Rhodotorula graminis* |
|  |  | *Lichtheimia corymbifera* |
|  |  | *Penicillium steckii* |
|  |  | *Hirsutella minnesotensis* |
|  |  | *Conidiobolus coronatus* |
|  | | |
| Bhutan | 10 | *Trypanosoma theileri* |
|  |  | *Asterionella formosa* |
|  |  | *Neospora caninum* |
|  |  | *Aspergillus carbonarius* |
|  |  | *Emiliania huxleyi* |
|  |  | *Yarrowia lipolytica* |
|  |  | *Parasitella parasitica* |
|  |  | *Pseudocohnilembus persalinus* |
|  |  | *Ectocarpus siliculosus* |
|  |  | *Thalassiosira pseudonana* |

| **Supplementary Table 14: Unique archaeal species in *kinema*** | | |
| --- | --- | --- |
| Sample site | Number of species | Archaeal species |
| India | 2 | *Methanolinea tarda* |
|  |  | *Methanocella paludicola* |
|  | | |
| Nepal | 6 | *Methanosalsum zhilinae* |
|  |  | *Methanosaeta harundinacea* |
|  |  | *Haloterrigena thermotolerans* |
|  |  | *Methanocaldococcus jannaschii* |
|  |  | *Methanocella arvoryzae* |
|  |  | *Pyrococcus furiosus* |
|  | | |
| Bhutan | 3 | *Thermoproteus* sp. AZ2 |
|  |  | *Methanoregula formicica* |
|  |  | *Haloprofundus marisrubri* |
