# Supplementary material for: Metagenomic-Metabolomic Mining of Kinema, a Naturally Fermented Soybean Food of the Eastern Himalayas
Source: Front Microbiol. 2022 Apr 29;13:868383. doi: 10.3389/fmicb.2022.868383 (PMC9106393; doi:10.3389/fmicb.2022.868383)
Supplement: Supplementary file 8 [file Table_8.DOCX]

| **Supplementary Table 15: Minor sub-pathways (Level-3) with a relative abundance of less than 1% identified using KEGG database.** | | | | |
| --- | --- | --- | --- | --- |
| Sl. No. | Level-3 (Sub-pathways) | Relative Abundance (%) | | |
|  |  | Kinema  (India) | Kinema  (Nepal) | Kinema  (Bhutan) |
| 1 | Nucleotide excision repair | 0.918706 | 0.960717 | 1.015452 |
| 2 | Phenylalanine, tyrosine and tryptophan biosynthesis | 0.941733 | 0.949028 | 0.890473 |
| 3 | Cell cycle - Caulobacter | 0.918706 | 0.911715 | 0.859229 |
| 4 | Terpenoid backbone biosynthesis | 0.870269 | 0.911715 | 0.813782 |
| 5 | Glycerolipid metabolism | 0.868681 | 0.89598 | 0.813782 |
| 6 | Fructose and mannose metabolism | 0.852801 | 0.880695 | 0.833665 |
| 7 | Ubiquinone and other terpenoid-quinone biosynthesis | 0.843272 | 0.826747 | 0.846447 |
| 8 | Homologous recombination | 0.783719 | 0.812361 | 0.761234 |
| 9 | Nicotinate and nicotinamide metabolism | 0.741635 | 0.727843 | 0.729989 |
| 10 | Nonribosomal peptide structures | 0.713049 | 0.686933 | 0.791058 |
| 11 | Riboflavin metabolism | 0.716225 | 0.766955 | 0.680282 |
| 12 | Histidine metabolism | 0.693992 | 0.670749 | 0.762654 |
| 13 | Base excision repair | 0.635233 | 0.654115 | 0.649037 |
| 14 | Pantothenate and CoA biosynthesis | 0.613 | 0.639729 | 0.60359 |
| 15 | Arginine biosynthesis | 0.565358 | 0.541274 | 0.521218 |
| 16 | Mismatch repair | 0.53836 | 0.533632 | 0.538261 |
| 17 | RNA polymerase | 0.53042 | 0.509355 | 0.549622 |
| 18 | Inositol phosphate metabolism | 0.511363 | 0.522393 | 0.481452 |
| 19 | Cationic antimicrobial peptide (CAMP) resistance | 0.49707 | 0.448664 | 0.53258 |
| 20 | Selenocompound metabolism | 0.46372 | 0.450912 | 0.521218 |
| 21 | Bacterial chemotaxis | 0.380346 | 0.415397 | 0.33375 |
| 22 | Methane metabolism | 0.373994 | 0.390671 | 0.340851 |
| 23 | Biotin metabolism | 0.34382 | 0.332227 | 0.377777 |
| 24 | Phenylalanine metabolism | 0.332703 | 0.295813 | 0.396239 |
| 25 | Sulfur relay system | 0.339056 | 0.327732 | 0.353633 |
| 26 | D-Alanine metabolism | 0.33985 | 0.31964 | 0.346532 |
| 27 | D-Glutamine and D-glutamate metabolism | 0.300942 | 0.318291 | 0.309606 |
| 28 | Benzoate degradation | 0.284267 | 0.281876 | 0.309606 |
| 29 | Protein processing in endoplasmic reticulum | 0.272356 | 0.274234 | 0.26984 |
| 30 | Bacterial secretion system | 0.241389 | 0.155549 | 0.356473 |
| 31 | Lysine degradation | 0.238212 | 0.228828 | 0.248537 |
| 32 | Polyketide sugar unit biosynthesis | 0.229478 | 0.262545 | 0.214452 |
| 33 | Biosynthesis of siderophore group nonribosomal peptides | 0.22789 | 0.239617 | 0.237175 |
| 34 | C5-Branched dibasic acid metabolism | 0.231066 | 0.230626 | 0.221553 |
| 35 | RNA transport | 0.232654 | 0.251756 | 0.181787 |
| 36 | Fatty acid degradation | 0.218361 | 0.194211 | 0.247117 |
| 37 | Glutathione metabolism | 0.208039 | 0.18567 | 0.257058 |
| 38 | Vitamin B6 metabolism | 0.212803 | 0.215341 | 0.220133 |
| 39 | Lipoic acid metabolism | 0.223126 | 0.231525 | 0.193149 |
| 40 | Valine, leucine and isoleucine degradation | 0.214391 | 0.212194 | 0.210191 |
| 41 | Biosynthesis of secondary metabolites - unclassified | 0.196128 | 0.206799 | 0.230074 |
| 42 | Ascorbate and aldarate metabolism | 0.224714 | 0.217589 | 0.187468 |
| 43 | Biofilm formation - Pseudomonas aeruginosa | 0.196922 | 0.183871 | 0.245697 |
| 44 | Tyrosine metabolism | 0.177071 | 0.14386 | 0.241436 |
| 45 | Valine, leucine and isoleucine biosynthesis | 0.18263 | 0.182972 | 0.188888 |
| 46 | Biofilm formation - Escherichia coli | 0.169925 | 0.149255 | 0.166165 |
| 47 | Aminobenzoate degradation | 0.168337 | 0.171733 | 0.140601 |
| 48 | Carbon fixation pathways in prokaryotes | 0.156426 | 0.16499 | 0.149122 |
| 49 | Epithelial cell signaling in Helicobacter pylori infection | 0.148486 | 0.151952 | 0.160484 |
| 50 | Non-homologous end-joining | 0.152456 | 0.155999 | 0.149122 |
| 51 | MAPK signaling pathway - plant | 0.138957 | 0.143411 | 0.160484 |
| 52 | Taurine and hypotaurine metabolism | 0.143722 | 0.155549 | 0.140601 |
| 53 | Lipopolysaccharide biosynthesis | 0.151662 | 0.09306 | 0.190308 |
| 54 | Phosphotransferase system (PTS) | 0.128635 | 0.121382 | 0.147702 |
| 55 | MAPK signaling pathway - fly | 0.114342 | 0.114639 | 0.1335 |
| 56 | alpha-Linolenic acid metabolism | 0.120694 | 0.134869 | 0.106516 |
| 57 | Arabinogalactan biosynthesis - Mycobacterium | 0.116724 | 0.141163 | 0.090894 |
| 58 | Phosphonate and phosphinate metabolism | 0.117518 | 0.105647 | 0.117878 |
| 59 | Other glycan degradation | 0.103225 | 0.118685 | 0.110777 |
| 60 | One carbon pool by folate | 0.109578 | 0.111492 | 0.107936 |
| 61 | beta-Lactam resistance | 0.108784 | 0.08272 | 0.129239 |
| 62 | beta-Alanine metabolism | 0.109578 | 0.106996 | 0.103676 |
| 63 | Bacterial invasion of epithelial cells | 0.103225 | 0.060691 | 0.156223 |
| 64 | Legionellosis | 0.102431 | 0.093509 | 0.117878 |
| 65 | Biofilm formation - Vibrio cholerae | 0.089727 | 0.117336 | 0.097995 |
| 66 | Penicillin and cephalosporin biosynthesis | 0.093697 | 0.086766 | 0.106516 |
| 67 | Apoptosis - fly | 0.092109 | 0.097106 | 0.092314 |
| 68 | Pertussis | 0.098461 | 0.085417 | 0.093734 |
| 69 | Secondary bile acid biosynthesis | 0.088139 | 0.090362 | 0.086633 |
| 70 | Tryptophan metabolism | 0.086551 | 0.062939 | 0.106516 |
| 71 | Autophagy - yeast | 0.087345 | 0.099803 | 0.06675 |
| 72 | Isoquinoline alkaloid biosynthesis | 0.086551 | 0.084518 | 0.082372 |
| 73 | Salmonella infection | 0.088933 | 0.07193 | 0.092314 |
| 74 | Longevity regulating pathway - multiple species | 0.076228 | 0.07193 | 0.088053 |
| 75 | Alzheimer disease | 0.072258 | 0.074178 | 0.071011 |
| 76 | Ribosome biogenesis in eukaryotes | 0.064317 | 0.071031 | 0.061069 |
| 77 | Peroxisome | 0.069876 | 0.067884 | 0.056808 |
| 78 | Caprolactam degradation | 0.062729 | 0.081371 | 0.045447 |
| 79 | NOD-like receptor signaling pathway | 0.065905 | 0.074628 | 0.045447 |
| 80 | Chloroalkane and chloroalkene degradation | 0.063523 | 0.049902 | 0.06675 |
| 81 | Zeatin biosynthesis | 0.064317 | 0.065187 | 0.048287 |
| 82 | Insulin signaling pathway | 0.051613 | 0.056645 | 0.06675 |
| 83 | Carotenoid biosynthesis | 0.047642 | 0.053948 | 0.049707 |
| 84 | Primary bile acid biosynthesis | 0.051613 | 0.055746 | 0.041186 |
| 85 | Arachidonic acid metabolism | 0.04129 | 0.046305 | 0.056808 |
| 86 | Nitrotoluene degradation | 0.046054 | 0.032369 | 0.06533 |
| 87 | Steroid hormone biosynthesis | 0.03732 | 0.047204 | 0.032665 |
| 88 | Renin-angiotensin system | 0.032556 | 0.034167 | 0.035505 |
| 89 | Mineral absorption | 0.024615 | 0.04091 | 0.019883 |
| 90 | Meiosis - yeast | 0.023821 | 0.031469 | 0.025564 |
| 91 | Neuroactive ligand-receptor interaction | 0.025409 | 0.026974 | 0.028404 |
| 92 | Tropane, piperidine and pyridine alkaloid biosynthesis | 0.030968 | 0.038213 | 0.011362 |
| 93 | Phenazine biosynthesis | 0.030174 | 0.02068 | 0.025564 |
| 94 | D-Arginine and D-ornithine metabolism | 0.026997 | 0.036864 | 0.011362 |
| 95 | Biosynthesis of unsaturated fatty acids | 0.021439 | 0.012588 | 0.032665 |
| 96 | Type I polyketide structures | 0.017469 | 0.031919 | 0.014202 |
| 97 | Biosynthesis of enediyne antibiotics | 0.019057 | 0.027873 | 0.014202 |
| 98 | cAMP signaling pathway | 0.023027 | 0.03057 | 0.005681 |
| 99 | Spliceosome | 0.014293 | 0.016634 | 0.024144 |
| 100 | Betalain biosynthesis | 0.017469 | 0.01079 | 0.022723 |
| 101 | Photosynthesis | 0.014293 | 0.018432 | 0.014202 |
| 102 | Steroid degradation | 0.016675 | 0.024726 | 0.00284 |
| 103 | Biosynthesis of type II polyketide products | 0.017469 | 0.012138 | 0.011362 |
| 104 | Limonene and pinene degradation | 0.016675 | 0.017083 | 0.007101 |
| 105 | Retrograde endocannabinoid signaling | 0.013499 | 0.007643 | 0.015622 |
| 106 | Chagas disease | 0.011117 | 0.006743 | 0.017043 |
| 107 | Sphingolipid metabolism | 0.009528 | 0.008092 | 0.015622 |
| 108 | Ethylbenzene degradation | 0.009528 | 0.005395 | 0.015622 |
| 109 | Monobactam biosynthesis | 0.010323 | 0.013487 | 0.005681 |
| 110 | Basal transcription factors | 0.008734 | 0.006294 | 0.012782 |
| 111 | Indole alkaloid biosynthesis | 0.006352 | 0.00989 | 0.009941 |
| 112 | Systemic lupus erythematosus | 0.00794 | 0.004945 | 0.012782 |
| 113 | Isoflavonoid biosynthesis | 0.007146 | 0.004496 | 0.012782 |
| 114 | Necroptosis | 0.00794 | 0.005395 | 0.009941 |
| 115 | Glycosaminoglycan degradation | 0.006352 | 0.003597 | 0.011362 |
| 116 | Retinol metabolism | 0.005558 | 0.004945 | 0.007101 |
| 117 | Geraniol degradation | 0.006352 | 0.003597 | 0.005681 |
| 118 | Cholinergic synapse | 0.00794 | 0.005844 | 0 |
| 119 | Carbapenem biosynthesis | 0.00397 | 0.002248 | 0.007101 |
| 120 | Linoleic acid metabolism | 0.00397 | 0.002248 | 0.007101 |
| 121 | N-Glycan biosynthesis | 0.00397 | 0.003597 | 0.005681 |
| 122 | Staphylococcus aureus infection | 0.007146 | 0.004046 | 0.00142 |
| 123 | Dioxin degradation | 0.00397 | 0.002697 | 0.005681 |
| 124 | Amoebiasis | 0.007146 | 0.004046 | 0 |
| 125 | Vibrio cholerae infection | 0.003176 | 0.002248 | 0.005681 |
| 126 | Prodigiosin biosynthesis | 0.003176 | 0.001798 | 0.005681 |
| 127 | Thermogenesis | 0.003176 | 0.002248 | 0.004261 |
| 128 | Signaling pathways regulating pluripotency of stem cells | 0.003176 | 0.001798 | 0.004261 |
| 129 | Viral carcinogenesis | 0.003176 | 0.003147 | 0.00284 |
| 130 | Carbon fixation in photosynthetic organisms | 0.002382 | 0.001349 | 0.004261 |
| 131 | Styrene degradation | 0.002382 | 0.001349 | 0.004261 |
| 132 | Lipoarabinomannan (LAM) biosynthesis | 0.002382 | 0.002697 | 0.00284 |
| 133 | Regulation of lipolysis in adipocytes | 0.002382 | 0.001349 | 0.00284 |
| 134 | Endocytosis | 0.003176 | 0.001798 | 0.00142 |
| 135 | Fluorobenzoate degradation | 0.001588 | 0.001798 | 0.00284 |
| 136 | mTOR signaling pathway | 0.002382 | 0.002248 | 0.00142 |
| 137 | Phagosome | 0.001588 | 0.001349 | 0.00284 |
| 138 | Protein digestion and absorption | 0.001588 | 0.001349 | 0.00284 |
| 139 | Steroid biosynthesis | 0.001588 | 0.002697 | 0.00142 |
| 140 | p53 signaling pathway | 0.001588 | 0.000899 | 0.00284 |
| 141 | Ras signaling pathway | 0.001588 | 0.000899 | 0.00284 |
| 142 | Staurosporine biosynthesis | 0.001588 | 0.000899 | 0.00284 |
| 143 | Chlorocyclohexane and chlorobenzene degradation | 0.001588 | 0.000899 | 0.00284 |
| 144 | Lysosome | 0.002382 | 0.001349 | 0.00142 |
| 145 | Proteasome | 0.001588 | 0.001798 | 0.00142 |
| 146 | Cellular senescence | 0 | 0.004496 | 0 |
| 147 | Acarbose and validamycin biosynthesis | 0.000794 | 0.001349 | 0.00142 |
| 148 | Phenylpropanoid biosynthesis | 0.000794 | 0.001349 | 0.00142 |
| 149 | Bisphenol degradation | 0.000794 | 0.001349 | 0.00142 |
| 150 | Polycyclic aromatic hydrocarbon degradation | 0.000794 | 0.001349 | 0.00142 |
| 151 | Sesquiterpenoid and triterpenoid biosynthesis | 0.000794 | 0.000899 | 0.00142 |
| 152 | Circadian rhythm - plant | 0.000794 | 0.000899 | 0.00142 |
| 153 | Glutamatergic synapse | 0.000794 | 0.000899 | 0.00142 |
| 154 | Thyroid hormone synthesis | 0.001588 | 0.001349 | 0 |
| 155 | MAPK signaling pathway - yeast | 0.000794 | 0.00045 | 0.00142 |
| 156 | Wnt signaling pathway | 0.000794 | 0.00045 | 0.00142 |
| 157 | Pathogenic Escherichia coli infection | 0.000794 | 0.00045 | 0.00142 |
| 158 | African trypanosomiasis | 0.000794 | 0.00045 | 0.00142 |
| 159 | Mannose type O-glycan biosynthesis | 0.000794 | 0.00045 | 0.00142 |
| 160 | Other types of O-glycan biosynthesis | 0.000794 | 0.00045 | 0.00142 |
| 161 | Biosynthesis of vancomycin group antibiotics | 0.000794 | 0.00045 | 0.00142 |
| 162 | Fatty acid elongation | 0.000794 | 0.000899 | 0 |
| 163 | Biosynthesis of ansamycins | 0.000794 | 0.000899 | 0 |
| 164 | Plant-pathogen interaction | 0.000794 | 0.000899 | 0 |
| 165 | ErbB signaling pathway | 0.000794 | 0.00045 | 0 |
| 166 | Hippo signaling pathway | 0.000794 | 0.00045 | 0 |
| 167 | PI3K-Akt signaling pathway | 0.000794 | 0.00045 | 0 |
| 168 | Ubiquitin mediated proteolysis | 0.000794 | 0.00045 | 0 |
| 169 | Transcriptional misregulation in cancer | 0.000794 | 0.00045 | 0 |
| 170 | Flavonoid biosynthesis | 0.000794 | 0.00045 | 0 |
| 171 | Glycosaminoglycan biosynthesis - heparan sulfate / heparin | 0.000794 | 0.00045 | 0 |
| 172 | Various types of N-glycan biosynthesis | 0.000794 | 0.00045 | 0 |
| 173 | Atrazine degradation | 0.000794 | 0.00045 | 0 |
| 174 | AMPK signaling pathway | 0 | 0.00045 | 0 |
| 175 | Calcium signaling pathway | 0 | 0.00045 | 0 |
| 176 | FoxO signaling pathway | 0 | 0.00045 | 0 |
| 177 | mRNA surveillance pathway | 0 | 0.00045 | 0 |
| 178 | Glycosphingolipid biosynthesis - globo and isoglobo series | 0 | 0.00045 | 0 |
| 179 | Furfural degradation | 0 | 0.00045 | 0 |
| 180 | RIG-I-like receptor signaling pathway | 0 | 0.00045 | 0 |
