# Supplementary material for: Metagenomic-Metabolomic Mining of Kinema, a Naturally Fermented Soybean Food of the Eastern Himalayas
Source: Front Microbiol. 2022 Apr 29;13:868383. doi: 10.3389/fmicb.2022.868383 (PMC9106393; doi:10.3389/fmicb.2022.868383)
Supplement: Supplementary file 9 [file Table_9.DOCX]

| **Supplementary Table 16. Predictive functional analysis of enzyme classification involved in various biochemical pathways** | | | | |
| --- | --- | --- | --- | --- |
| KO ID | Function | Relative Abundance (%) | | |
|  |  | Kinema (India) | Kinema (Nepal) | Kinema (Bhutan) |
| **Proteases** | | | | |
| K12536 | ATP-binding cassette, subfamily C, bacterial exporter for protease/lipase | 0.001805 | 0.001013 | 0.003267 |
| K06891 | ATP-dependent Clp protease adaptor protein ClpS | 0.001805 | 0.001013 | 0.001633 |
| K03694 | ATP-dependent Clp protease ATP-binding subunit ClpA | 0.004963 | 0.002786 | 0.006533 |
| K03695 | ATP-dependent Clp protease ATP-binding subunit ClpB | 0.043312 | 0.040523 | 0.050631 |
| K03696 | ATP-dependent Clp protease ATP-binding subunit ClpC | 0.072186 | 0.072688 | 0.081663 |
| K03697 | ATP-dependent Clp protease ATP-binding subunit ClpE | 0.04647 | 0.052933 | 0.031032 |
| K04086 | ATP-dependent Clp protease ATP-binding subunit ClpL | 0.004512 | 0.002786 | 0.004083 |
| K03544 | ATP-dependent Clp protease ATP-binding subunit ClpX | 0.053688 | 0.053439 | 0.061247 |
| K01358 | ATP-dependent Clp protease, protease subunit [EC:3.4.21.92] | 0.126777 | 0.117009 | 0.118412 |
| K03667 | ATP-dependent HslUV protease ATP-binding subunit HslU | 0.051884 | 0.053693 | 0.051448 |
| K01419 | ATP-dependent HslUV protease, peptidase subunit HslV [EC:3.4.25.2] | 0.031581 | 0.032165 | 0.028582 |
| K01338 | ATP-dependent Lon protease [EC:3.4.21.53] | 0.065419 | 0.066609 | 0.060431 |
| K04076 | ATP-dependent Lon protease [EC:3.4.21.53] | 0.046921 | 0.0504 | 0.044098 |
| K03797 | carboxyl-terminal processing protease [EC:3.4.21.102] | 0.077149 | 0.092443 | 0.069414 |
| K03798 | cell division protease FtsH [EC:3.4.24.-] | 0.090233 | 0.099027 | 0.081663 |
| K13274 | cell wall-associated protease [EC:3.4.21.-] | 0.032484 | 0.030139 | 0.029399 |
| K08315 | hydrogenase 3 maturation protease [EC:3.4.23.51] | 0.001353 | 0.00076 | 0.000817 |
| K03605 | hydrogenase maturation protease [EC:3.4.23.-] | 0.000451 | 0.000253 | 0.000817 |
| K20486 | lantibiotic leader peptide-processing serine protease [EC:3.4.21.-] | 0.002707 | 0.002786 | 0.000817 |
| K08642 | LasA protease [EC:3.4.24.-] | 0 | 0.002026 | 0 |
| K04770 | Lon-like ATP-dependent protease [EC:3.4.21.-] | 0.003609 | 0.002279 | 0.005716 |
| K07177 | Lon-like protease | 0.031581 | 0.034951 | 0.032665 |
| K13275 | major intracellular serine protease [EC:3.4.21.-] | 0.021656 | 0.019248 | 0.020416 |
| K12537 | membrane fusion protein, protease secretion system | 0.001353 | 0.00076 | 0.00245 |
| K04087 | membrane protease subunit HflC [EC:3.4.-.-] | 0.013986 | 0.015196 | 0.00735 |
| K04088 | membrane protease subunit HflK [EC:3.4.-.-] | 0.011279 | 0.017222 | 0.0049 |
| K07403 | membrane-bound serine protease (ClpP class) | 0.042409 | 0.044322 | 0.034299 |
| K07387 | metalloprotease [EC:3.4.24.-] | 0.002707 | 0.00152 | 0.004083 |
| K13277 | minor extracellular protease Epr [EC:3.4.21.-] | 0.02707 | 0.02406 | 0.030215 |
| K14647 | minor extracellular serine protease Vpr [EC:3.4.21.-] | 0.063163 | 0.059771 | 0.064514 |
| K19416 | modulator of FtsH protease | 0.001805 | 0.00152 | 0.001633 |
| K12538 | outer membrane protein, protease secretion system | 0.002256 | 0.001266 | 0.004083 |
| K06972 | presequence protease [EC:3.4.24.-] | 0.002256 | 0.001266 | 0.000817 |
| K05520 | protease I [EC:3.5.1.124] | 0.064065 | 0.069395 | 0.051448 |
| K01407 | protease III [EC:3.4.24.55] | 0.002707 | 0.00152 | 0.004083 |
| K04773 | protease IV [EC:3.4.21.-] | 0.041507 | 0.041789 | 0.038382 |
| K16922 | putative peptide zinc metalloprotease protein | 0.00767 | 0.011397 | 0.003267 |
| K08303 | putative protease [EC:3.4.-.-] | 0.084368 | 0.085604 | 0.09228 |
| K07395 | putative proteasome-type protease | 0.000451 | 0.000253 | 0.000817 |
| K11749 | regulator of sigma E protease [EC:3.4.24.-] | 0.046921 | 0.044575 | 0.042465 |
| K19225 | rhomboid protease GluP [EC:3.4.21.105] | 0.05053 | 0.061544 | 0.037565 |
| K14645 | serine protease [EC:3.4.21.-] | 0.008572 | 0.01089 | 0.00245 |
| K17734 | serine protease AprX [EC:3.4.21.-] | 0.020302 | 0.020515 | 0.015516 |
| K04772 | serine protease DegQ [EC:3.4.21.-] | 0.003158 | 0.001773 | 0.005716 |
| K04691 | serine protease DegS [EC:3.4.21.-] | 0.00406 | 0.002533 | 0.0049 |
| K04771 | serine protease Do [EC:3.4.21.107] | 0.100158 | 0.107892 | 0.093096 |
| K04774 | serine protease SohB [EC:3.4.21.-] | 0.004512 | 0.002533 | 0.00735 |
| K06012 | spore protease [EC:3.4.24.78] | 0.037898 | 0.041536 | 0.034299 |
| K12685 | subtilase-type serine protease [EC:3.4.21.-] | 0.004512 | 0.002786 | 0.003267 |
| K01342 | subtilisin [EC:3.4.21.62] | 0.026619 | 0.023047 | 0.030215 |
| **Glucosidases** | | | | |
| K05349 | beta-glucosidase [EC:3.2.1.21] | 0.041958 | 0.046095 | 0.024499 |
| K01223 | 6-phospho-beta-glucosidase [EC:3.2.1.86] | 0.193098 | 0.189444 | 0.189459 |
| K01187 | alpha-glucosidase [EC:3.2.1.20] | 0.068126 | 0.070155 | 0.065331 |
| K01210 | glucan 1,3-beta-glucosidase [EC:3.2.1.58] | 0.000451 | 0.000507 | 0.000817 |
| K21574 | glucan 1,4-alpha-glucosidase [EC:3.2.1.3] | 0.000451 | 0.000253 | 0.000817 |
| K01215 | glucan 1,6-alpha-glucosidase [EC:3.2.1.70] | 0.002707 | 0.00152 | 0.004083 |
| K01232 | maltose-6'-phosphate glucosidase [EC:3.2.1.122] | 0.0194 | 0.020008 | 0.017966 |
| K01182 | oligo-1,6-glucosidase [EC:3.2.1.10] | 0.062712 | 0.065849 | 0.057164 |
| **Galactosidases** | | | | |
| K12308 | beta-galactosidase [EC:3.2.1.23] | 0.060907 | 0.058505 | 0.054714 |
| K12111 | evolved beta-galactosidase subunit alpha [EC:3.2.1.23] | 0.001353 | 0.00076 | 0.00245 |
| K12112 | evolved beta-galactosidase subunit beta | 0.000451 | 0.000253 | 0.000817 |
| K01220 | 6-phospho-beta-galactosidase [EC:3.2.1.85] | 0.001353 | 0.001013 | 0.001633 |
| K07407 | alpha-galactosidase [EC:3.2.1.22] | 0.064516 | 0.085604 | 0.039198 |
| K01224 | arabinogalactan endo-1,4-beta-galactosidase [EC:3.2.1.89] | 0.032484 | 0.034951 | 0.018783 |
| K07407 | alpha-galactosidase [EC:3.2.1.22] | 0.064516 | 0.085604 | 0.039198 |
| **Amylases** | | | | |
| K01176 | alpha-amylase [EC:3.2.1.1] | 0.03113 | 0.035964 | 0.031849 |
| K01208 | cyclomaltodextrinase / maltogenic alpha-amylase / neopullulanase [EC:3.2.1.54 3.2.1.133 3.2.1.135] | 0.042861 | 0.041029 | 0.048998 |
| K05343 | maltose alpha-D-glucosyltransferase / alpha-amylase [EC:5.4.99.16 3.2.1.1] | 0.000451 | 0.000253 | 0.000817 |
| **Lipases** | | | | |
| K01054 | acylglycerol lipase [EC:3.1.1.23] | 0.001805 | 0.001773 | 0.000817 |
| K12536 | ATP-binding cassette, subfamily C, bacterial exporter for protease/lipase | 0.001805 | 0.001013 | 0.003267 |
| K13535 | cardiolipin-specific phospholipase [EC:3.1.1.-] | 0.000451 | 0.000253 | 0.000817 |
| K01048 | lysophospholipase [EC:3.1.1.5] | 0.02707 | 0.033938 | 0.030215 |
| K13985 | N-acyl-phosphatidylethanolamine-hydrolysing phospholipase D [EC:3.1.4.54] | 0.00767 | 0.004306 | 0.008983 |
| K01058 | phospholipase A1/A2 [EC:3.1.1.32 3.1.1.4] | 0.000902 | 0.000507 | 0.001633 |
| K01114 | phospholipase C [EC:3.1.4.3] | 0.008121 | 0.004812 | 0.013883 |
| K06999 | phospholipase/carboxylesterase | 0.041056 | 0.036724 | 0.041648 |
| K01047 | secretory phospholipase A2 [EC:3.1.1.4] | 0.000451 | 0.000253 | 0.000817 |
| K01046 | triacylglycerol lipase [EC:3.1.1.3] | 0.035642 | 0.042042 | 0.032665 |
| **gamma-polyglutamic acid proteins and enzymes** | | | | |
| K07282 | gamma-polyglutamate biosynthesis protein CapA | 0.025716 | 0.026593 | 0.026949 |
| K22116 | gamma-polyglutamate biosynthesis protein CapC | 0.012633 | 0.01317 | 0.008166 |
| K01932 | gamma-polyglutamate synthase [EC:6.3.2.-] | 0.015791 | 0.018235 | 0.012249 |
| K07680 | two-component system, NarL family, sensor histidine kinase ComP [EC:2.7.13.3] | 0.050982 | 0.057745 | 0.037565 |
| K07777 | two-component system, NarL family, sensor histidine kinase DegS [EC:2.7.13.3] | 0.037898 | 0.045335 | 0.024499 |
| K07778 | two-component system, NarL family, sensor histidine kinase DesK [EC:2.7.13.3] | 0.069479 | 0.074714 | 0.067781 |
| K02250 | competence protein ComK | 0.033837 | 0.03723 | 0.032665 |
| K02251 | competence protein ComQ | 0.044214 | 0.0504 | 0.026132 |
| K02253 | competence protein ComX | 0.001805 | 0.003039 | 0 |
| K02254 | competence protein ComZ | 0.008121 | 0.007598 | 0.011433 |
| K00284 | glutamate synthase (ferredoxin) [EC:1.4.7.1] | 0.021656 | 0.018995 | 0.029399 |
| K00264 | glutamate synthase (NADH) [EC:1.4.1.14] | 0.001353 | 0.001266 | 0.000817 |
| K00265 | glutamate synthase (NADPH) large chain [EC:1.4.1.13] | 0.095196 | 0.092443 | 0.105346 |
| K00266 | glutamate synthase (NADPH) small chain [EC:1.4.1.13] | 0.033386 | 0.035204 | 0.035932 |
