# Supplementary material for: Metagenomic-Metabolomic Mining of Kinema, a Naturally Fermented Soybean Food of the Eastern Himalayas
Source: Front Microbiol. 2022 Apr 29;13:868383. doi: 10.3389/fmicb.2022.868383 (PMC9106393; doi:10.3389/fmicb.2022.868383)
Supplement: Supplementary file 10 [file Table_10.DOCX]

| **Supplementary Table 17. Predictive aminoacyl-tRNA biosynthesis inferred by the KEGG database.** | | | | |
| --- | --- | --- | --- | --- |
| KO ID | Function | Relative Abundance (%) | | |
|  |  | *Kinema* (India) | *Kinema* (Nepal) | *Kinema* (Bhutan) |
| K01885 | glutamyl-tRNA synthetase [EC:6.1.1.17] | 0.009926 | 0.005825 | 0.013066 |
| K09698 | nondiscriminating glutamyl-tRNA synthetase [EC:6.1.1.24] | 0.05053 | 0.055719 | 0.051448 |
| K01886 | glutaminyl-tRNA synthetase [EC:6.1.1.18] | 0.014888 | 0.009118 | 0.017966 |
| K02433 | aspartyl-tRNA(Asn)/glutamyl-tRNA(Gln) amidotransferase subunit A [EC:6.3.5.6 6.3.5.7] | 0.06587 | 0.062304 | 0.063697 |
| K01872 | alanyl-tRNA synthetase [EC:6.1.1.7] | 0.087526 | 0.082818 | 0.100446 |
| K01893 | asparaginyl-tRNA synthetase [EC:6.1.1.22] | 0.044214 | 0.044828 | 0.039198 |
| K01873 | valyl-tRNA synthetase [EC:6.1.1.9] | 0.078502 | 0.070408 | 0.086563 |
| K01869 | leucyl-tRNA synthetase [EC:6.1.1.4] | 0.066772 | 0.069902 | 0.071047 |
| K01870 | isoleucyl-tRNA synthetase [EC:6.1.1.5] | 0.091135 | 0.084591 | 0.107796 |
| K04566 | lysyl-tRNA synthetase, class I [EC:6.1.1.6] | 0.000451 | 0.000253 | 0.000817 |
| K04567 | lysyl-tRNA synthetase, class II [EC:6.1.1.6] | 0.067675 | 0.068889 | 0.07268 |
| K01887 | arginyl-tRNA synthetase [EC:6.1.1.19] | 0.082112 | 0.08763 | 0.086563 |
| K01889 | phenylalanyl-tRNA synthetase alpha chain [EC:6.1.1.20] | 0.049628 | 0.048881 | 0.049815 |
| K01890 | phenylalanyl-tRNA synthetase beta chain [EC:6.1.1.20] | 0.075344 | 0.071168 | 0.078397 |
| K01866 | tyrosyl-tRNA synthetase [EC:6.1.1.1] | 0.067675 | 0.066103 | 0.07513 |
| K01867 | tryptophanyl-tRNA synthetase [EC:6.1.1.2] | 0.052786 | 0.050907 | 0.062064 |
| K09759 | nondiscriminating aspartyl-tRNA synthetase [EC:6.1.1.23] | 0.009474 | 0.005319 | 0.017149 |
| K01876 | aspartyl-tRNA synthetase [EC:6.1.1.12] | 0.059102 | 0.057238 | 0.062064 |
| K01878 | glycyl-tRNA synthetase alpha chain [EC:6.1.1.14] | 0.032033 | 0.03951 | 0.022866 |
| K01868 | threonyl-tRNA synthetase [EC:6.1.1.3] | 0.086172 | 0.088897 | 0.088196 |
| K01875 | seryl-tRNA synthetase [EC:6.1.1.11] | 0.074442 | 0.079019 | 0.062064 |
| K01042 | L-seryl-tRNA(Ser) seleniumtransferase [EC:2.9.1.1] | 0.010828 | 0.006838 | 0.015516 |
| K01883 | cysteinyl-tRNA synthetase [EC:6.1.1.16] | 0.051433 | 0.050653 | 0.051448 |
| K01874 | methionyl-tRNA synthetase [EC:6.1.1.10] | 0.103317 | 0.093709 | 0.121678 |
| K00604 | methionyl-tRNA formyltransferase [EC:2.1.2.9] | 0.029326 | 0.028366 | 0.034299 |
| K01881 | prolyl-tRNA synthetase [EC:6.1.1.15] | 0.064065 | 0.062557 | 0.07513 |
| K01892 | histidyl-tRNA synthetase [EC:6.1.1.21] | 0.05414 | 0.050147 | 0.058798 |
