# Supplementary material for: Metagenomic-Metabolomic Mining of Kinema, a Naturally Fermented Soybean Food of the Eastern Himalayas
Source: Front Microbiol. 2022 Apr 29;13:868383. doi: 10.3389/fmicb.2022.868383 (PMC9106393; doi:10.3389/fmicb.2022.868383)
Supplement: Supplementary file 11 [file Table_11.DOCX]

| **Supplementary Table 18. Predictive enzymes involved in phosphotransferase system (PTS) pathways inferred by the KEGG database** | | | | |
| --- | --- | --- | --- | --- |
| KO ID | Function | Relative Abundance (%) | | |
|  |  | *Kinema* (India) | *Kinema* (Nepal) | *Kinema* (Bhutan) |
| K08483 | phosphotransferase system, enzyme I, PtsI [EC:2.7.3.9] | 0.060456 | 0.060531 | 0.065331 |
| K02784 | phosphocarrier protein HPr | 0.001353 | 0.00076 | 0.00245 |
| K02777 | PTS system, sugar-specific IIA component [EC:2.7.1.-] | 0.041958 | 0.052426 | 0.031849 |
| K02798 | PTS system, mannitol-specific IIA component [EC:2.7.1.197] | 0.010828 | 0.008105 | 0.0098 |
| K20114 | PTS system, galactose-specific IIC component | 0.001353 | 0.00076 | 0.00245 |
| K20112 | PTS system, galactose-specific IIA component [EC:2.7.1.204] | 0.000451 | 0.000253 | 0.000817 |
| K02775 | PTS system, galactitol-specific IIC component | 0.009474 | 0.009877 | 0.013066 |
| K02773 | PTS system, galactitol-specific IIA component [EC:2.7.1.200] | 0.003609 | 0.002026 | 0.003267 |
| K02774 | PTS system, galactitol-specific IIB component [EC:2.7.1.200] | 0.004512 | 0.003292 | 0.0049 |
| K02783 | PTS system, glucitol/sorbitol-specific IIC component | 0.007219 | 0.005319 | 0.008166 |
| K02781 | PTS system, glucitol/sorbitol-specific IIA component [EC:2.7.1.198] | 0.004512 | 0.002786 | 0.00245 |
| K11196 | PTS system, fructose-specific IIC component | 0.008121 | 0.009624 | 0.00735 |
| K02771 | PTS system, fructose-specific IID component | 0.012633 | 0.01165 | 0.008166 |
| K11194 | PTS system, fructose-specific IIA component [EC:2.7.1.202] | 0.010828 | 0.012663 | 0.008166 |
| K11195 | PTS system, fructose-specific IIB component [EC:2.7.1.202] | 0.01173 | 0.012157 | 0.008983 |
| K02793 | PTS system, mannose-specific IIA component [EC:2.7.1.191] | 0.002256 | 0.001266 | 0.003267 |
| K02794 | PTS system, mannose-specific IIB component [EC:2.7.1.191] | 0.00406 | 0.002533 | 0.005716 |
| K02795 | PTS system, mannose-specific IIC component | 0.011279 | 0.006838 | 0.015516 |
| K02796 | PTS system, mannose-specific IID component | 0.0194 | 0.026087 | 0.017966 |
| K02770 | PTS system, fructose-specific IIC component | 0.004963 | 0.005825 | 0.005716 |
| K02768 | PTS system, fructose-specific IIA component [EC:2.7.1.202] | 0.009023 | 0.012157 | 0.0098 |
| K02769 | PTS system, fructose-specific IIB component [EC:2.7.1.202] | 0.001353 | 0.003039 | 0.001633 |
| K00882 | 1-phosphofructokinase [EC:2.7.1.56] | 0.043312 | 0.044068 | 0.040832 |
| K08484 | phosphotransferase system, enzyme I, PtsP [EC:2.7.3.9] | 0.004512 | 0.002533 | 0.005716 |
