# Supplementary material for: Metagenomic-Metabolomic Mining of Kinema, a Naturally Fermented Soybean Food of the Eastern Himalayas
Source: Front Microbiol. 2022 Apr 29;13:868383. doi: 10.3389/fmicb.2022.868383 (PMC9106393; doi:10.3389/fmicb.2022.868383)
Supplement: Supplementary file 12 [file Table_12.DOCX]

**Supplementary Table 19.** Metabolites in *kinema* samples identified by LC-MS associated with predictive KEGG pathways referring Human Database (HMDB) and PlantCyc database.

| **KEGG Pathways** | **Metabolites profile by LC-MS** |
| --- | --- |
| 1. **Metabolism** |  |
| D-Glutamine and D-glutamate metabolism | Glutamine, glutamate |
| Alanine, aspartate and glutamate metabolism | Asparagine, alanine, glutamate, GABA, citrate, N-acetyl-L-aspartate |
| Valine, leucine and isoleucine biosynthesis | Valine, leucine, isoleucine |
| Valine, leucine and isoleucine degradation | Valine, leucine, isoleucine |
| Glutathione metabolism | Glutamate, ornithine |
| D-Alanine metabolism | Alanine |
| Cysteine and methionine metabolism | Alanine |
| Arginine biosynthesis | Arginine, glutamate, ornithine |
| Arginine and proline metabolism | Arginine, glutamate, ornithine, GABA |
| Glycine, serine and threonine metabolism | Sarcosine |
| D-Arginine and D-ornithine metabolism | Arginine, ornithine |
| Lysine biosynthesis | Lysine |
| Lysine degradation | Lysine, L-aminoadipate |
| Phenylalanine metabolism | Phenylalanine, phenylacetaldehyde, tyrosine |
| Phenylalanine, tyrosine and tryptophan biosynthesis | Phenylalanine, tyrosine, tryptophan |
| Tyrosine metabolism | Tyrosine |
| Tryptophan metabolism | Tryptophan, serotonin, 3-hydroxy-L-kynurenine, 3-hydroxyanthranilate, melatonin |
| Histidine metabolism | Glutamate |
| Butanoate metabolism | Glutamate, GABA |
| Galactose Metabolism | Galactitol, D-sorbitol, galactose, myo-inositol |
| Fructose and mannose metabolism | D-sorbitol, fructose, D-mannose |
| Pentose phosphate pathway | Ribose |
| Pentose and glucuronate interconversions | xylose |
| Amino sugar and nucleotide sugar metabolism | Fructose, galactose |
| Starch and sucrose metabolism | Fructose, D-mannose |
| Glycolysis / Gluconeogenesis | glucose |
| Ascorbate and aldarate metabolism | D-glucarate, myo-inositol |
| Inositol phosphate metabolism | scyllo-inositol, myo-inositol |
| Citrate cycle (TCA cycle) | Citrate, isocitrate |
| Glyoxylate and dicarboxylate metabolism | Citrate, isocitrate, glutamate |
| Biotin Metabolism | Biotin, lysine |
| Nicotinate and nicotinamide metabolism | Nicotinamide |
| Vitamin B6 metabolism | Pyridoxamine, pyridoxine |
| Pantothenate and CoA biosynthesis | Pantothenate, valine |
| Riboflavin metabolism | Riboflavin |
| Porphyrin and chlorophyll metabolism | Glutamate |
| Lipoic acid metabolism | (R)-lipoate |
| Ubiquinone and other terpenoid-quinone biosynthesis | Tyrosine |
| Linoleic acid metabolism | Linoleate |
| Biosynthesis of unsaturated fatty acids | Linoleate, linolenate, oleate |
| Fatty acid biosynthesis | Palmitoleate, oleate |
| alpha-Linolenic acid metabolism | Linolenate, jasmonate |
| Arachidonic acid metabolism | 19(R)-Hydroxy-prostaglandin e2 |
| Purine metabolism | Adenosine, adenine, guanine |
| Pyrimidine metabolism | Thymine |
| beta-Alanine metabolism | Pantothenate, GABA |
| Selenocompound metabolism | Alanine |
| Glutathione metabolism | Glutamate, ornithine |
| Styrene degradation | Phenylacetaldehyde, acrylamide |
| Benzoate degradation | Benzoate |
| Dioxin degradation | Benzoate, salicylate |
| Aminobenzoate degradation | Benzoate, terephthalate, aniline, 3-hydroxyanthranilate |
| Polycyclic aromatic hydrocarbon degradation | Salicylate, terephthalate |
| Isoflavonoid biosynthesis | Daidzein, genistein, isovitexin, genistin, maackiain, biochanin-A, (+)-pisatin |
| Flavonoid biosynthesis | Apigenin, chrysin, chrysophanol |
| Tropane, piperidine and pyridine alkaloid biosynthesis | Swainsonine |
| Indole alkaloid biosynthesis | Sarpagine, catharanthine |
| Sesquiterpenoid and triterpenoid biosynthesis | Solavetivone |
| Peptidoglycan biosynthesis | Alanine |
| Nitrogen metabolism | Glutamate |
| 1. **Genetic Information Processing** |  |
| Aminoacyl-tRNA biosynthesis | Alanine, arginine, asparagine, glutamate, glutamine, leucine, isoleucine, lysine, ornithine, phenylalanine, tryptophan, tyrosine, valine |
| 1. **Environmental Information Processing** |  |
| ABC transporters | D-mannitol, terephthalate |
| Phosphotransferase system (PTS) | Galactitol, D-sorbitol, D-mannitol, fructose, D-mannose, glucose |
| Two-component system | Citrate, isocitrate |
| cAMP signaling pathway | Serotonin |
| Neuroactive ligand-receptor interaction | Serotonin, melatonin, GABA |
