# Supplementary material for: Metagenomic-Metabolomic Mining of Kinema, a Naturally Fermented Soybean Food of the Eastern Himalayas
Source: Front Microbiol. 2022 Apr 29;13:868383. doi: 10.3389/fmicb.2022.868383 (PMC9106393; doi:10.3389/fmicb.2022.868383)
Supplement: Supplementary file 13 [file Table_13.DOCX]

**Supplementary Table 20a.** **Significant primary metabolites (*p*<0.05) in *kinema* analysed by LC-MS represented by the average transformed log abundance (log x_i_ + 1) with ±SD.**

| **Sl. No.** | **Primary metabolites** | **Average log (x_i_ + 1) abundance with standard deviation (Avg ± SD)** | | |
| --- | --- | --- | --- | --- |
|  |  | ***Kinema* (India)** | ***Kinema* (Nepal)** | ***Kinema* (Bhutan)** |
|  | 1. **Amino acids** |  |  |  |
| 1 | Alanine | 8.30±0.11 | 8.37±0.07 | 8.46±0.01 |
| 2 | Arginine | 7.79±0.10 | 8.11±0.03 | 7.66±0.20 |
| 3 | Asparagine | 7.51±0.03 | 7.49±0.08 | 7.73±0.20 |
| 4 | Glutamate | 8.70±0.07 | 8.63±0.02 | 8.81±0.01 |
| 5 | Glutamine | 7.20±0.09 | 7.14±0.01 | 7.09±0.09 |
| 6 | Leucine/Isoleucine | 8.48±0.12 | 8.20±0.11 | 8.41±0.16 |
| 7 | Lysine | 8.57±0.05 | 8.64±0.11 | 8.77±0.16 |
| 8 | Ornithine | 7.72±0.10 | 7.61±0.09 | 7.85±0.15 |
| 9 | Phenylalanine | 8.38±0.07 | 8.28±0.03 | 8.34±0.17 |
| 10 | Tryptophan | 9.06±0.04 | 9.24±0.02 | 9.04±0.19 |
| 11 | Tyrosine | 7.78±0.16 | 7.90±0.002 | 7.79±0.01 |
| 12 | Valine | 9.48±0.10 | 9.53±0.02 | 9.62±0.08 |
| 13 | 4-aminobutanoate | 7.41±0.09 | 7.30±0.06 | 7.59±0.04 |
|  | 1. **Fatty acids** |  |  |  |
| 14 | Linoleate | 10.55±0.01 | 10.62±0.002 | 10.52±0.03 |
| 15 | Palmitoleate | 8.11±0.04 | 8.11±0.01 | 8.16±0.05 |
| 16 | Linolenate | 9.77±0.003 | 9.85±0.01 | 9.75±0.04 |
| 17 | Oleate | 10.06±0.05 | 10.15±0.03 | 10.06±0.04 |
|  | 1. **Sugars** |  |  |  |
| 18 | Galactitol, sorbitol, mannitol | 7.66±0.13 | 7.59±0.07 | 7.62±0.03 |
| 19 | Ribose, xylulose, arabinose, xylose | 7.37±0.16 | 6.90±0.04 | 7.21±0.07 |
| 20 | Fructose, mannose, galactose, glucose, scyllo-inositol, myo-inositol | 8.88±0.12 | 8.95±0.04 | 8.73±0.04 |
|  | 1. **Organic acids** |  |  |  |
| 21 | Citrate, Isocitrate | 10.20±0.02 | 10.25±0.05 | 10.02±0.15 |
| 22 | Benzoate | 7.10±0.11 | 7.33±0.10 | 7.54±0.09 |
| 23 | Cinnamate | 6.64±0.06 | 6.50±0.03 | 6.72±0.15 |
| 24 | Salicylate | 8.25±0.03 | 8.06±0.07 | 8.15±0.04 |
| 25 | Terephthalate | 7.26±0.01 | 7.21±0.06 | 7.29±0.16 |
| 26 | D-glucarate | 8.53±0.21 | 8.88±0.02 | 8.67±0.01 |
|  | 1. **Vitamins** |  |  |  |
| 27 | Biotin (Vitamin B7) | 8.38±0.01 | 8.50±0.10 | 8.22±0.12 |
| 28 | Nicotinamide (Vitamin B3) | 7.86±0.11 | 8.19±0.03 | 7.52±0.05 |
| 29 | Pyridoxamine (Vitamin B6) | 8.37±0.15 | 8.36±0.06 | 8.23±0.14 |
| 30 | Pyridoxine (Vitamin B6) | 7.36±0.15 | 7.44±0.07 | 7.47±0.05 |
| 31 | (R)-Pantothenate (Vitamin B5) | 8.87±0.02 | 8.81±0.02 | 8.50±0.14 |
| 32 | Riboflavin (Vitamin B2) | 7.48±0.04 | 7.44±0.10 | 7.55±0.23 |
|  | 1. **Miscellaneous** |  |  |  |
| 33 | Adenine | 8.61±0.07 | 8.63±0.01 | 8.60±0.10 |
| 34 | Adenosine | 8.19±0.06 | 8.31±0.03 | 8.03±0.05 |
| 35 | Guanine | 7.72±0.17 | 8.08±0.06 | 7.83±0.06 |
| 36 | Thymine | 7.59±0.04 | 7.48±0.03 | 7.57±0.11 |

**Supplementary Table 20b:** **Significant secondary metabolites (*p*<0.05) in *kinema* analysed by LC-MS represented by the average transformed log abundance (log x_i_ + 1) with ±SD.**

| Sl. No. | Secondary Metabolites and other bioactive compounds | Average log (x_i_ + 1) abundance with standard deviation (Avg ± SD) | | |
| --- | --- | --- | --- | --- |
|  |  | *Kinema* (India) | *Kinema* (Nepal) | *Kinema* (Bhutan) |
| 1 | Daidzein, chrysin, chrysophanol | 9.32±0.06 | 9.44±0.10 | 9.26±0.10 |
| 2 | Genistein, Apigenin | 8.72±0.0008 | 8.76±0.03 | 8.71±0.15 |
| 3 | Isovitexin, Genistin | 8.17±0.10 | 8.28±0.003 | 8.16±0.02 |
| 4 | Maackiain, Biochanin-A | 8.64±0.07 | 8.51±0.05 | 8.66±0.05 |
| 5 | (+)-pisatin | 6.39±0.03 | 6.49±0.11 | 6.53±0.05 |
| 6 | Soyasaponin III | 7.37±0.06 | 7.60±0.02 | 7.51±0.01 |
| 7 | Swainsonine | 7.73±0.13 | 7.59±0.14 | 7.84±0.13 |
| 8 | Serotonin | 8.00±0.01 | 7.87±0.09 | 8.02±0.08 |
| 9 | 19(R)-Hydroxy-prostaglandin e2 | 7.22±0.03 | 6.20±0.04 | 6.80±0.05 |
| 10 | Solavetivone | 6.68±0.10 | 6.62±0.16 | 6.87±0.18 |
| 11 | 3-hydroxy-L-kynurenine | 7.60±0.08 | 7.47±0.08 | 7.52±0.001 |
| 12 | Benzimidazole | 6.56±0.05 | 6.71±0.04 | 6.78±0.10 |
| 13 | Sarpagine | 7.07±0.08 | 6.96±0.08 | 7.24±0.15 |
| 14 | Catharanthine | 7.54±0.11 | 6.83±0.0007 | 7.40±0.02 |
| 15 | Phenylacetaldehyde | 7.43±0.08 | 7.16±0.08 | 7.54±0.10 |
| 16 | Jasmonate | 7.23±0.0006 | 7.30±0.01 | 7.44±0.18 |
| 17 | Methyl Jasmonate | 7.28±0.05 | 7.18±0.06 | 7.00±0.13 |
| 18 | N-feruloyltyramine | 7.12±0.11 | 7.02±0.06 | 7.43±0.10 |
| 19 | γ-glutamyl-ethylamide | 8.28±0.03 | 8.22±0.001 | 8.56±0.01 |
| 20 | 4α-formyl-4β-methyl-5α-cholesta-8,24-dien-3β-ol | 6.83±0.15 | 7.02±0.07 | 7.18±0.11 |
| 21 | 1-18:0-2-18:2-phosphatidylethanolamine | 7.45±0.09 | 7.45±0.07 | 7.18±0.06 |
| 22 | Demethylphylloquinone (Vitamin K) | 6.57±0.04 | 6.63±0.06 | 7.25±0.12 |
| 23 | Harmalol | 8.44±0.07 | 8.28±0.08 | 8.35±0.17 |
| 24 | p-coumaroyltyramine | 7.78±0.02 | 7.09±0.09 | 7.26±0.03 |
| 25 | Aniline | 6.62±0.11 | 6.73±0.12 | 6.78±0.01 |
| 26 | 3-hydroxyanthranilate | 7.62±0.09 | 7.48±0.10 | 7.63±0.05 |
| 27 | Acrylamide | 8.01±0.05 | 8.19±0.03 | 7.97±0.14 |
| 28 | Deferoxamine mesylate | 7.45±0.14 | 7.14±0.06 | 7.53±0.07 |
| 29 | 4-O-oxalyl-L-threonate | 7.03±0.002 | 6.67±0.01 | 6.96±0.09 |
| 30 | L-quinate | 8.04±0.11 | 8.15±0.01 | 8.32±0.09 |
| 31 | 4-methylpyrazole | 7.75±0.08 | 7.76±0.05 | 7.99±0.09 |
| 32 | Melatonin | 7.70±0.04 | 7.44±0.01 | 7.56±0.06 |
| 33 | (R)-lipoate | 8.95±0.05 | 8.98±0.06 | 8.99±0.05 |
| 34 | L,L-diaminopimelate | 6.95±0.11 | 7.06±0.02 | 7.05±0.11 |
| 35 | L-2-aminoadipate | 8.87±0.07 | 8.76±0.07 | 9.10±0.13 |
| 36 | L-2,3-dihydrodipicolinate | 7.17±0.18 | 7.00±0.07 | 7.16±0.06 |
| 37 | N-Acetyl-L-aspartic acid | 8.73±0.06 | 8.58±0.04 | 9.05±0.02 |
