# Supplementary material for: Metagenomic-Metabolomic Mining of Kinema, a Naturally Fermented Soybean Food of the Eastern Himalayas
Source: Front Microbiol. 2022 Apr 29;13:868383. doi: 10.3389/fmicb.2022.868383 (PMC9106393; doi:10.3389/fmicb.2022.868383)
Supplement: Supplementary file 14 [file Table_14.DOCX]

**Supplementary Table** 21. The log transformed fold change (FC) representing the abundance variation of metabolites among *kinema* samples from different locations.

| **Metabolites** | **Log FC *kinema*** | | |  |
| --- | --- | --- | --- | --- |
|  | **India/Nepal** | **India/Bhutan** | **Nepal/Bhutan** | **p-Value** |
| Daidzein, chrysin, chrysophanol | -0.41156 | 0.209889 | 0.621448 | 0.014651 |
| (+)-pisatin | -0.36862 | -0.47008 | -0.10146 | 0.00757 |
| Genistein, apigenin | -0.13725 | -0.00129 | 0.13596 | 0.049658 |
| Biochanin-A, maackiain | 0.236012 | 0.098325 | -0.13769 | 0.028414 |
| Isovitexin, Genistin | -0.90984 | 1.20998 | 2.119818 | 1.6E-06 |
| Benzimidazole | -0.4859 | -0.73126 | -0.24536 | 0.000476 |
| Serotonin | 0.424462 | -0.09608 | -0.52054 | 0.006865 |
| Solavetivone | 0.1931 | -0.65117 | -0.84427 | 0.049075 |
| p-coumaroyltyramine | 2.250853 | 1.718889 | -0.53196 | 1.31E-09 |
| Swainsonine | 0.471286 | -0.11959 | -0.59087 | 0.000033 |
| Sarpagine | 0.423454 | -0.88182 | -1.30527 | 0.003364 |
| Soyasaponin III | -0.76208 | -0.46247 | 0.299619 | 3.96E-05 |
| 4-methylpyrazole | -0.0177 | -0.79917 | -0.78147 | 0.000469 |
| Melatonin | 0.507191 | 0.222649 | -0.28454 | 0.043166 |
| 3-hydroxyanthranilate | 0.482492 | -0.01277 | -0.49526 | 0.03253 |
| 3-hydroxy-L-kynurenine | 0.443754 | 0.291686 | -0.15207 | 0.017558 |
| Catharanthine | 2.386164 | 0.483658 | -1.90251 | 1.99E-09 |
| Salicylate | 0.605232 | 0.329044 | -0.27619 | 0.000401 |
| N-acetylaspartic acid | 0.514603 | -1.03566 | -1.55026 | 6.38E-09 |
| 19(R)-hydroxy Prostaglandin E2 | 3.372818 | 1.363318 | -2.0095 | 3.53E-13 |
| Phenylacetaldehyde | 2.931468 | -0.19006 | -3.12152 | 1.17E-07 |
| Methyl jasmonate | 0.366207 | 1.207088 | 0.840881 | 3.2E-05 |
| Jasmonate | -0.23164 | -0.75197 | -0.52032 | 0.019578 |
| Riboflavin | 0.122644 | -0.33278 | -0.45543 | 0.53513 |
| Biotin | -0.43337 | 0.50708 | 0.940451 | 0.001056 |
| Pyridoxamine | 0.377276 | 0.485188 | 0.107912 | 0.12573 |
| Pyridoxine | -0.21742 | -0.31128 | -0.09386 | 0.29452 |
| (R)-pantothenate | 0.201943 | 1.198164 | 0.996221 | 2.5E-05 |
| Nicotinamide | -1.09111 | 1.135871 | 2.226978 | 1.74E-08 |
| Demethylphylloquinone | -0.46035 | -1.48895 | -1.0286 | 8.24E-07 |
